# Visible-Light-Driven Carboxylative 1,2-Difunctionalization of C=C Bonds with Tetrabutylammonium Oxalate

**DOI:** 10.1021/acscentsci.4c01464

**Published:** 2024-11-15

**Authors:** Sai Wang, Pei Xu, Zhi-Tao Liu, Yi-Qin Liu, Hao-Qiang Jiang, Tian-Zi Hao, Hui-Xian Jiang, Hui Xu, Xu-Dong Cao, Dong Guo, Xu Zhu

**Affiliations:** † Jiangsu Key Laboratory of New Drug Research and Clinical Pharmacy, School of Pharmacy, Xuzhou Medical University, 209 Tongshan Road, Xuzhou 221004, China; ‡ Key Laboratory of Organic Synthesis of Jiangsu Province, College of Chemistry, Chemical Engineering and Materials Science, Soochow University, Suzhou 215123, China

## Abstract

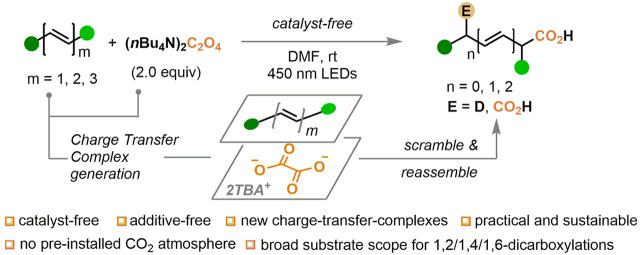

Herein, we report a visible-light-induced charge-transfer-complex-enabled dicarboxylation and deuterocarboxylation of C=C bonds with oxalate as a masked CO_2_ source under catalyst-free conditions. In this reaction, we disclosed the first example that the tetrabutylammonium oxalate could be able to aggregate with aryl substrates via π–cation interactions to form the charge transfer complexes, which subsequently triggers the single electron transfer from the oxalic dianion to the ammonium countercation under irradiation of 450 nm bule LEDs, releasing CO_2_ and CO_2_ radical anions. Diverse alkenes, dienes, trienes, and indoles, including challenging trisubstituted olefins, underwent dicarboxylation and anti-Markovnikov deuterocarboxylation with high selectivity to access valuable 1,2- and 1,4-dicarboxylic acids as well as indoline-derived diacids and β-deuterocarboxylic acids under mild conditions. The *in situ* generated CO_2_^•–^ and CO_2_ molecules from oxalic radical anions could both add to the C=C bond without assistance of any photocatalyst or additives, which made this reaction sustainable, clean, and efficient.

## Abstract 2

Tetrabutylammonium oxalate (TBAO) works as a carboxylation reagent for 1,2-, 1,4-, and 1,6-difunctionalization of alkene, diene, and triene substrates in the absence of photocatalyst or any additives.

The solar-driven photocatalytic carbon dioxide (CO_2_) reduction reaction has gained significant attention due to its dual capability to generate high-value fuels while mitigating CO_2_ pollution.^1,2^ In organic chemistry fundamental research and the medicine industry, CO_2_ was widely used as the carbonyl (C1) synthon for alkene dicarboxylation under photocatalytic or electrocatalytic conditions^3−11^ to forge thermodynamically and kinetically stable C—C bonds, producing value-added dicarboxylic acids (Figure 1).^12−14^ Since the 1990s, electrocatalysis for alkene dicarboxylation with CO_2_ was first reported by Duñach and co-workers^15^ and then developed by several groups employing a sacrificial anode system.^16−21^ The deep reduction potential generated on the cathode usually caused poor functional group tolerance and therefore limited its applications in organic synthesis (Scheme 1a).^22^ Recently, photoredox catalysis has emerged as a sustainable alternative to generate highly reductive systems with an exogenous electron donor allowing access to more energy demanding substrates.^23−25^ In 2021, Yu and co-workers disclosed an elegant reductive photocatalytic system for alkene dicarboxylation with CO_2_ as the carbonyl source and DIPEA (*N*,*N*-diisopropylethylamine) as the electron donor (Scheme 1b).^26^ In these reactions, excess amounts of CO_2_ (1 atm of atmosphere, hard to quantify) as the carbonyl source were necessary. The requirement of either sacrificial anodes or stoichiometric electron donors leaves space for improvement of such transformations. A catalyst-free strategy to access diacids from alkenes is still under developed.

**Figure 1:**
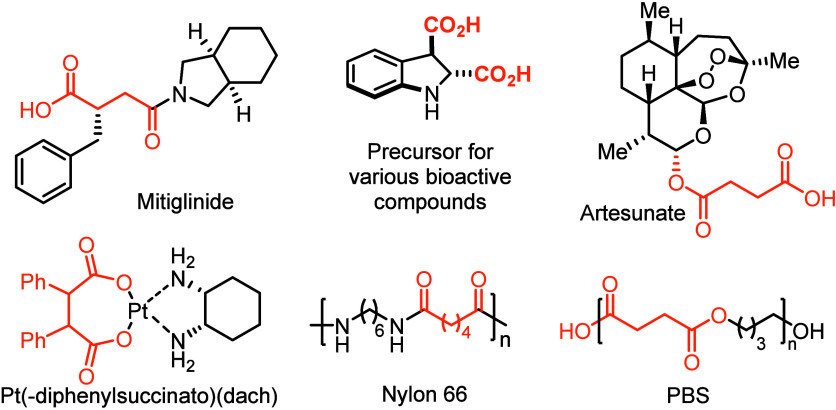
Representative pharmaceutical drug molecules, synthetic intermediates, and polymers containing 1,2-, 1,4-, or 1,6-dicarboxylic backbones.

**Scheme 1:**
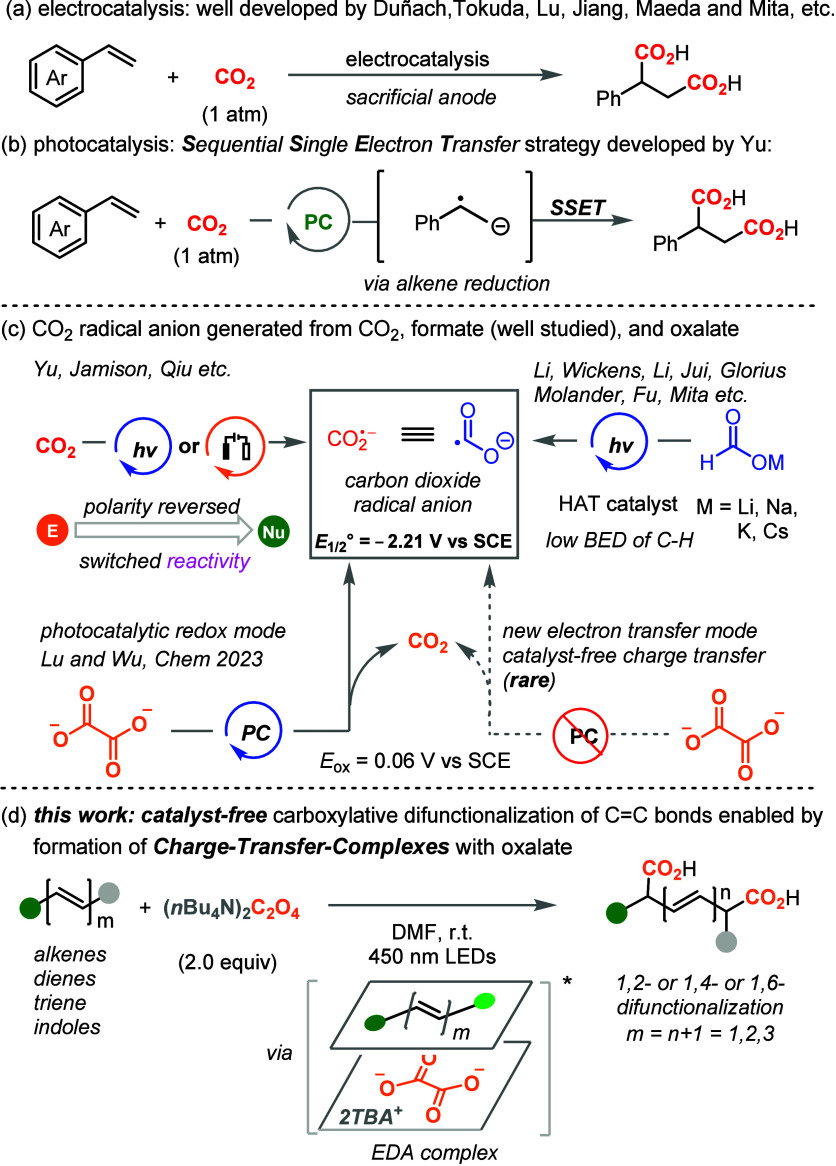
(a) Electrocatalysis and (b) Photocatalysis to Access Diacids from Alkenes with CO_2_ As the C1 Source, (c) the Model of CO_2_ Radical Anion Generation, and (d) Photoinduced Additive-Free Protocol with Oxalate (This Work)

Recently, the carbon dioxide radical anion (CO_2_^•–^) was exploited as the masked CO_2_ (C1 source) for carboxylation reactions, as shown in Scheme 1c.^27−31^ In addition, the polarity of CO_2_^•–^ is reversed compared with CO_2_; thus, new reactivities could be expected for this radical anion species.^32−40^ For example, in 2022, Yu and co-workers reported the first carbo–carboxylation reaction of unactivated alkenes involving formation of the CO_2_^•–^ species from CO_2_.^41^ In such reaction, the CO_2_^•–^ was generated from CO_2_ via single electron transfer (SET) under highly reducing conditions (*E*_red_ = −2.21 V vs SCE).^42^ Therefore, certain photocatalysts and excess amounts of DIPEA as the sacrificial electron donor were necessary. From 2021, Li,^43−45^ Wickens,^46−50^ Li,^51−53^ Jui,^54,55^ Molander,^56,57^ Glorius,^58^ Fu,^59,60^ and Mita^61,62^ developed a series of carboxylation or reduction reactions with formate, a masked quantitative CO_2_ reagent, which could generate CO_2_^•–^ species via hydrogen atom transfer (HAT). However, in the presence of the HAT catalyst, dicarboxylation of alkenes with formate as the sole carbonyl source is still challenging. Development of a new CO_2_^•–^ precursor/masked CO_2_ reagent for dicarboxylation of alkenes under mild, clean, and sustainable manner is highly desired.

Light-induced intermolecular charge transfer (CT), or electron (E)–donor (D)–acceptor (A) complexes formed through assembly of two substrates that do not absorb at the desired wavelength individually, was recently applied as a principle to realize a photoredox cycle for synthetic organic chemistry in a photocatalyst-free manner.^63−68^ Recently, we found that tetrabutylammonium oxalate (TBAO) could act as an electron donor to generate the EDA complexes with electron deficient substrates, such as *N*-Bz imines, to access amino acids under catalyst-free conditions.^69,70^ During our further investigation on TBAO, herein, we disclose our recent development on the alkene dicarboxylation reaction with TBAO as the masked CO_2_ source via a unique electron transfer manner, where the noninnocent countercation played a significant role during the electron transformation process (Scheme 1d).

On the basis of the literature^71−74^ and our previous studies on CO_2_^•–^ species,^75−81^ we envisioned that oxalic dianions could interact with aryl alkenes via anion−π interactions and be excited by photoirradiation to undergo SET and generate CO_2_^•–^ and CO_2_. As expected, after careful screening of the reaction parameters (see more screening details in the Supporting Information), we realized the diacid product **2a** in 73% isolated yield when substrate **1a** was treated with tetrabutylammonium oxalate (TBAO) in DMF under 450 nm LED irradiation (Table 1, entry 1). The countercation of the oxalic salt is crucial as the Na^+^, NH_4_^+^, or Et_4_N^+^ could not convert the alkene to diacid at all (Table 1, entries 2–4). Two equivalents of TBAO were optimal to give the best yields (Table 1, entries 5 and 6). The wavelength of the light source was also screened, and 450 nm LEDs are the best to give the corresponding product **2a** (Table 1, entries 7–9). Without light irradiation, no conversion of **1a** was observed (Table 1, entry 10). Interestingly, a similar yield of **2a** was obtained when 3DPAFIPN (2,4,6-tris(diphenylamino)-5-fluoroisophthalonitrile) was used as the photocatalyst.

**Table 1:**
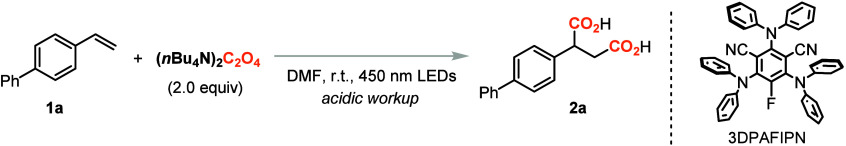
Optimization of the Reaction Conditions^a^

entry	variation of the standard conditions	yield (%)^b^
1	none	76(73)^c^
2	Na_2_C_2_O_4_ instead of TBAO	0
3	(NH_4_)_2_C_2_O_4_ instead of TBAO	0
4	(NEt_4_)_2_C_2_O_4_ instead of TBAO	0
5	1.2 equiv of TBAO	46
6	1.5 equiv of TBAO	60
7	410 nm LEDs	40
8	430 nm LEDs	41
9	460 nm LEDs	52
10	no light	0
11	with 3DPAFIPN and CsF^d^	76

a Reaction conditions: **1a** (0.2 mmol) and oxalate (2.0 equiv) in DMF (0.13 M) at r.t. for 12 h under N_2_ atmosphere.

b Crude ^1^H NMR yield with dichloroethane as the internal standard.

c Isolated yield.

d 3DPAFIPN (2.0 mol %) and CsF (5.0 equiv) were used.

This is encouraging as there was no literature precedent showing that alkenes react with oxalate without any photocatalyst or additive to form the succinic acids. To further understand the reaction mechanism and test the substrate scope, we next treated α-cyclohexyl styrene (**1b**) with the standard reaction conditions. However, no desired diacid **2b** was observed. Interestingly, when the reaction was conducted under a CO_2_ atmosphere, the desired diacid **2b** was isolated in 36% yield. Afterward, the styrene **1c** was investigated and the desired diacid **2c** was isolated in 62% yield when a catalytic amount of 3DPAFIPN was added in the reaction. For *para*-MeO or *para*-CN substituted styrenes (**1d** and **1e**), both the photocatalyst and the CO_2_ atmosphere were necessary for the dicarboxylation process. It seems that the biaryl structure on substrate **1a** is crucial to initiate the reaction. Afterward, some other 1,1-disubstituted monoaryl styrenes **1f**–**1h** were tested, and the corresponding desired diacids could also be obtained in the presence of photocatalyst. With these results in hand, we envisioned that the highly conjugated alkenyl substrate might be crucial to realize the diacids. Therefore, the 2-phenyl aryl alkene **1i** was tested and the desired product **2i** was isolated in 95% yield in the absence of any additive. The 3-phenoxyl aryl alkene **1j** was also a good substrate for the dicarboxylation process to give **2j** in 62% yield. When the 4-benzyl aryl alkene **1k** was examined, the product **2k** was formed, although the yield is only 34%.

The heteroarene pyrrole was also tolerated in this reaction (**2n**). Afterward, the substituents on the aryl ring of substrates were tested to verify the functional group tolerance and electron density effluences. The methoxy (**2o** and **2p**), cyano (**2q**), ester (**2r**), and fluoro (**2s**) groups were well tolerated and provided the desired diacids in satisfied yields. The vulnerable trimethylsilyl (TMS) group was also retained after the transformation to give product **2t** in 78% yield. Moreover, the disubstituted alkene **1u** was investigated and the corresponding diacid **2u** was obtained in good yield.

To further elaborate the utility of this protocol for dicarboxylation, various 1,1-diaryl alkenes were examined under the optimized reaction conditions. The electron density on the aryl ring did not affect the reaction efficiency, and products **2aa**–**2ae** tethering fluoro or methyl groups were obtained in good yields. The naphthyl and heteroaryl substituents were also tolerated well to give the desired product in up to 89% yield (**2af** and **2ag**). The fluoro and methoxy groups tethered on the aryl rings were also amenable (**2ah** and **2ai**). To our delight, the steric hindered trisubstituted alkenes also worked well to give the diacids in moderate to good yields (Table 2C, **2ba**–**2bf**). In most cases, elevated temperature was required to realize satisfied yields. Then, the acrylate derivative **1ca** as well as the d-menthol derived acrylate **1cb** were treated with the standard conditions to give the diacids **2ca** and **2cb** in 56% and synthetically useful yield, respectively, without degradation of the ester motif. The 1,1-diaryl alkene **1cc** derived from d-menthol could also be converted to diacid **2cc** in 60% yield. Interestingly, the perillyl alcohol derived substrate **1cd**, which has multiple alkenyl moieties, could give the chemoselective dicarboxylation product **2cd** in synthetically useful yield in the presence of photocatalyst. Overall, various biaryl styrenes and 1,1-diaryl alkenes could be smoothly converted to the corresponding diacids in good yields without a photocatalyst or additives. However, the monoaryl alkenes were inert for this transformation, and addition of photocatalyst is required. The highly conjugated π-system seems crucial in initiating the photocatalyst-free charge transfer process of the reaction. As the CO_2_ radical anion was generated as a strong reductant during the transformation, dehalogenation or reduction of the carbonyl groups happened and no desired products were isolated from the halo- or carbonyl substrates.

**Table 2:**
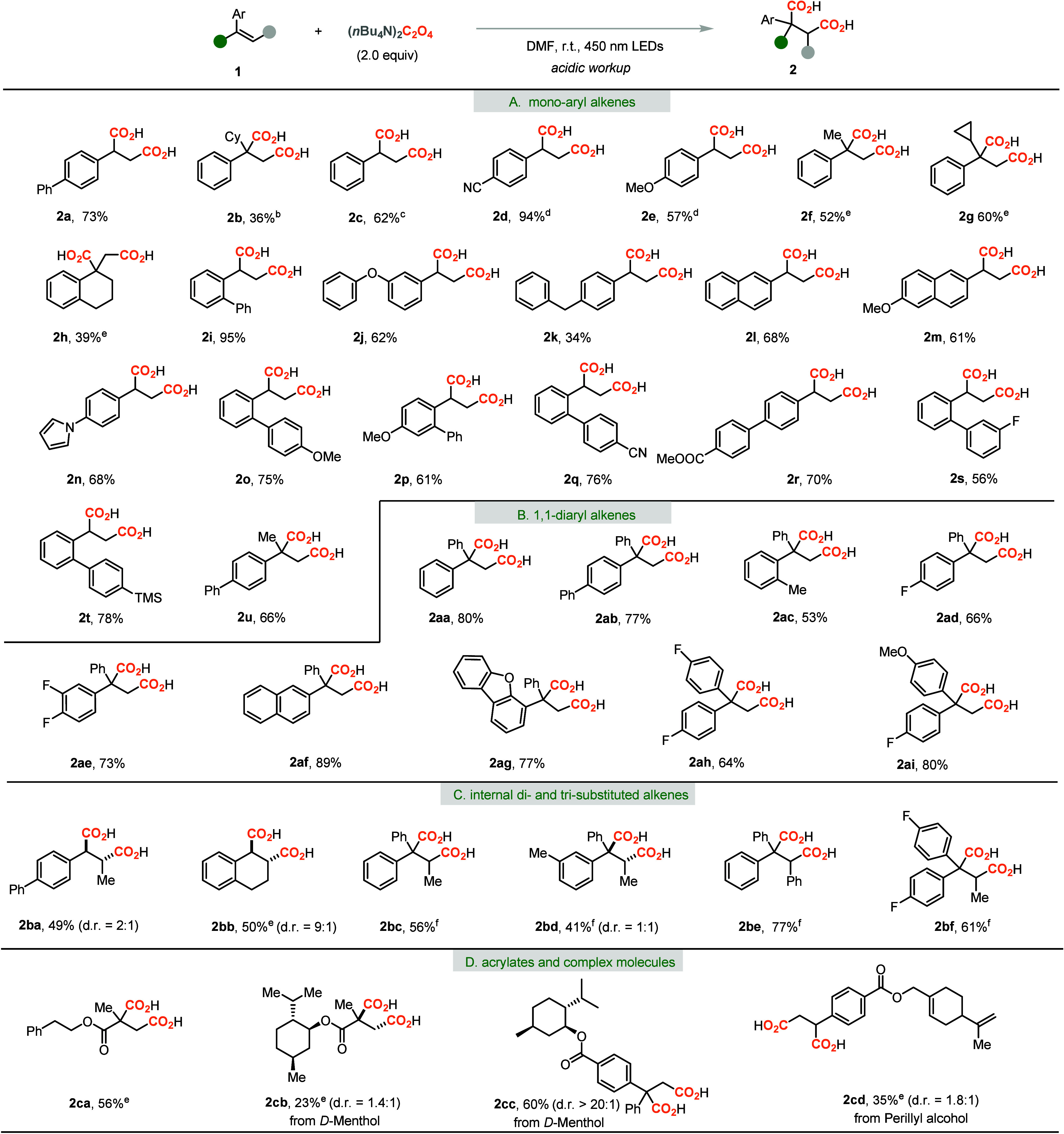
Scope of Alkenes and Later Stage Modification of Natural Products^a^

a Reaction conditions: **1** (0.2 mmol) and (*n*Bu_4_N)_2_C_2_O_4_ (2.0 equiv) in DMF (0.13 M) at r.t. for 12–48 h under N_2_ atmosphere and 450 nm LED irradiation.

b CO_2_ atmosphere.

c 3DPAFIPN (2.5 mol %) and CsF (8.0 equiv) were used.

d 3DPAFIPN (2.5 mol %), CsF (5.0 equiv), and CO_2_ atmosphere were used.

e 3DPAFIPN (2.5 mol %) and CsF (5.0 equiv) were used.

f 50 °C instead of r.t.

Aryl dienes also own a highly conjugated π-system that is probably suitable for the catalyst-free dicarboxylation process. As we know, dicarboxylation of dienes would provide unsaturated diacids with diverse functional groups as the modification handles. However, such reactions were rarely disclosed with limited examples and relatively low yields.^8^ Therefore, various aryl dienes were exploited, as shown in Table 3. When 1,1-diphenyl 1,3-butadiene (**3a**) was treated with the standard reaction conditions, to our delight, unsaturated dicarboxylic acid **4a** was obtained in 90% yield. The substrates bearing a methyl, phenyl, or fluoro group could also react smoothly and gave the 1,3-dicarboxylated products in good yields as the *E*/*Z* mixtures (**4b**–**4f**). A relatively higher temperature of 50 °C was required to get good regioselectivity, and formation of the 1,4-dicarboxylation product was completely prohibited. When the monoaryl substituted diene **3g** was investigated, the 1,4-dicarboxylation product **5g** was obtained in 29% yield with excellent regioselectivity and *E*/*Z* ratio. When diene **3h** was utilized, the desired unsaturated diacid **5h** was obtained in 62% yield. The naphthyl substituted diene **3i** was also a good candidate for the 1,4-dicarboxylation process. The disubstituted and internal dienes were also tested, and the unsaturated diacids **5k**–**5l** were obtained in good yields. When 1,3-dienes were treated with the standard reaction conditions, the allyl radicals would prefer the isomerization and form the less steric hindered radicals or more stabilized benzylic radicals. Therefore, 1,4-dicarboxylation was observed for dienes **5g**–**5l**. Interestingly, the dicarboxylation process can be applied for the triene system **3m**, and the 1,6-dicarboxylation product **5m** with a dienyl functionality was isolated in 37% yield with excellent regioselectivity.

**Table 3:**
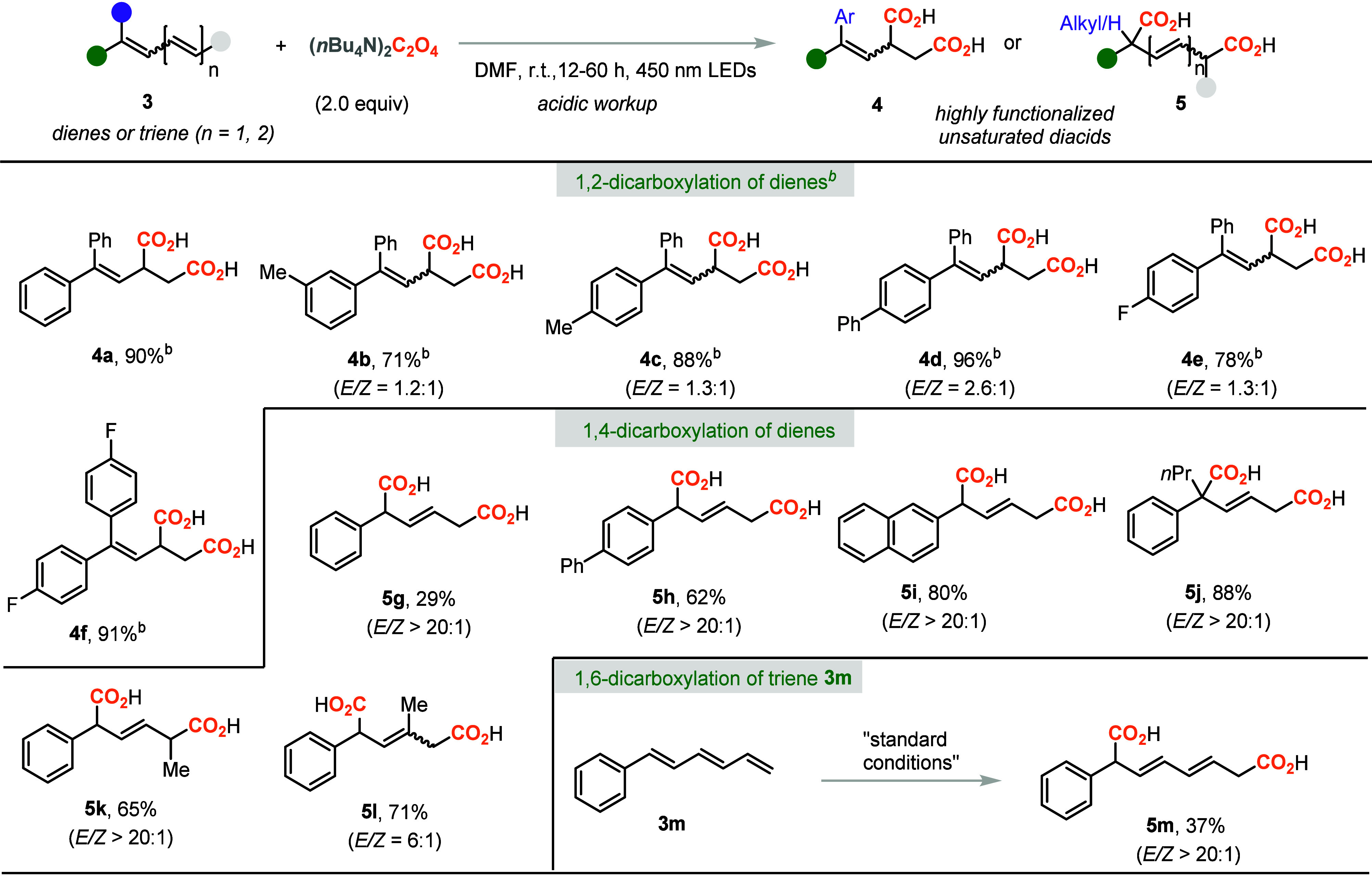
Scope of Dienes for Selective 1,2- or 1,4-Dicarboxylation^a^

a Reaction conditions: **1** (0.2 mmol) and (*n*Bu_4_N)_2_C_2_O_4_ (2.0 equiv) were irradiated with 450 nm LEDs in DMF (0.13 M) at r.t. for 12–48 h under N_2_ atmosphere.

b 50 °C was used instead of r.t.

As shown in Table 4, *N*-Boc protected indole (**6a**) was then investigated and dearomatization of the indole ring happened in the presence of photocatalyst, providing the dicarboxylated indoline product **6a** in 60% yield. Afterward, the effects of the fluoro group on the 4, 5, and 7 positions were examined, and all of them could be converted to the corresponding diacids in moderate to good yields (**7b**–**7d**). 6-Methylindole **6e** and ester **6f** could also be converted to indoline diacids **7e** and **7f**, respectively, in good yields. The 3-methyl substituted indole **6g**, which is sterically hindered, was also amenable to give **7g** in 69% yield, although excess amounts of base were required. Interestingly, 2-phenyl indole **6h** could provide diacid **7h** in 36% yield even in the absence of the photocatalyst. This result suggested again that a highly conjugated π-system could trigger single electron transfer under photoirradiation in the absence of photocatalyst.

**Table 4:**
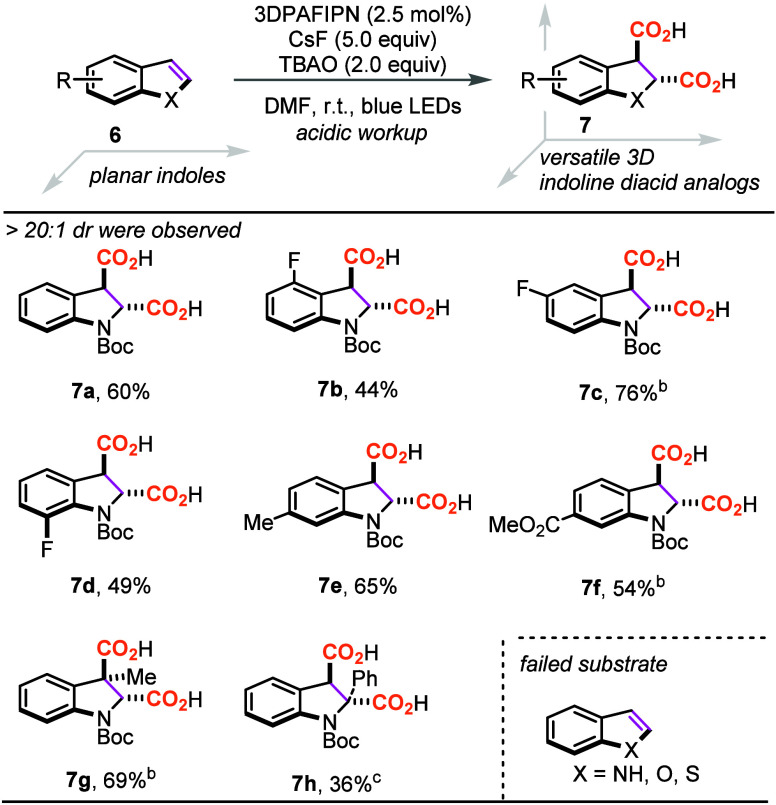
Scope of Indoles for 1,2-Dicarboxylation^a^

a Reaction conditions: **6** (0.2 mmol), 3DPAFIPN (2.5 mol %), (*n*Bu_4_N)_2_C_2_O_4_ (2.0 equiv), and CsF (5.0 equiv) in DMF (0.13 M) at r.t. for 12–48 h under N_2_ atmosphere and 450 nm LED irradiation.

b CsF (8.0 equiv) was used.

c Without 3DPAFIPN.

To further elaborate the synthetic utility of this difunctionalization reaction and probe the formation of carbon anion intermediates during the transformation, the deuterocarboxylation of the representative alkenes was investigated, as shown in Table 5. The 1,1-diaryl alkenes were good substrates for deuterocarboxylation to give product **8a** in 69% yield with a 90% deuteration ratio. The steric hindered trisubstituted alkene was also amenable to give **8b** in good yield and deuteration ratio. It is not surprising that the diene substrate could also be converted to the corresponding deuterocarboxylic acid **8c** in a moderate yield and deuteration ratio. When the simple styrene substrates were examined, the deuterocarboxylation products (**8d** and **8e**) could also be obtained. However, the addition of photocatalyst to the reaction system is required.

**Table 5:**
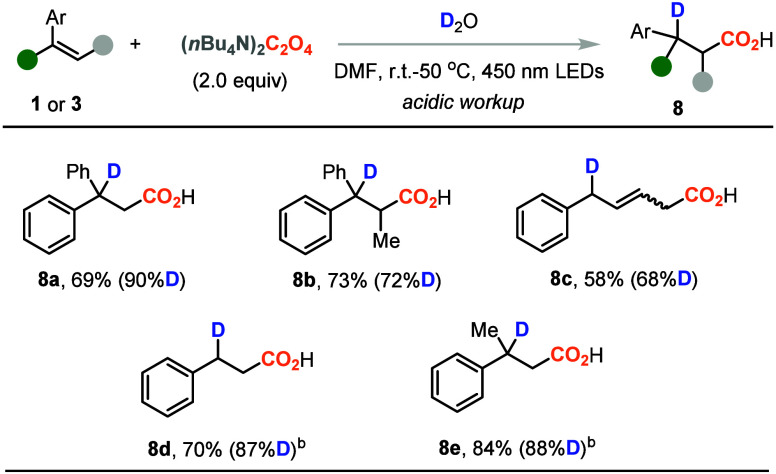
Representative Examples for Deuterocarboxylation^a^

a Reaction conditions: **1** or **3** (0.2 mmol), (*n*Bu_4_N)_2_C_2_O_4_ (2.0 equiv), and D_2_O (2.0–10.0 equiv as indicated in the SI) in DMF (0.13 M) at r.t. or 50 °C under N_2_ atmosphere and 450 nm LED irradiation.

b 3DPAFIPN (2.5 mol %) was used.

Moreover, the application of the diacid products obtained in this reaction were conducted as shown in Scheme 2. First, the large-scale reaction of substrate **1a** was investigated and the desired diacid **2a** was obtained in 56% yield. Afterward, the succinic acid **2aa** could be easily converted to the pyrrolidine-2,5-dione **9**, which is the precursor for anticonvulsant drug derivatives.^82^ The unsaturated diacid **4a** was first methylated to give diester **10** and the alkenyl moiety could be further converted to functionalized six-membered lactone **11** in the presence of NBS in DMSO. The indoline **7a** was methylated and then the Boc group was deprotected under acidic conditions. The rearomatization of the indole ring was realized by treating it with DDQ in dioxane. Indole **14** could be further converted into various biologically active drug molecules.^83,84^

**Scheme 2:**
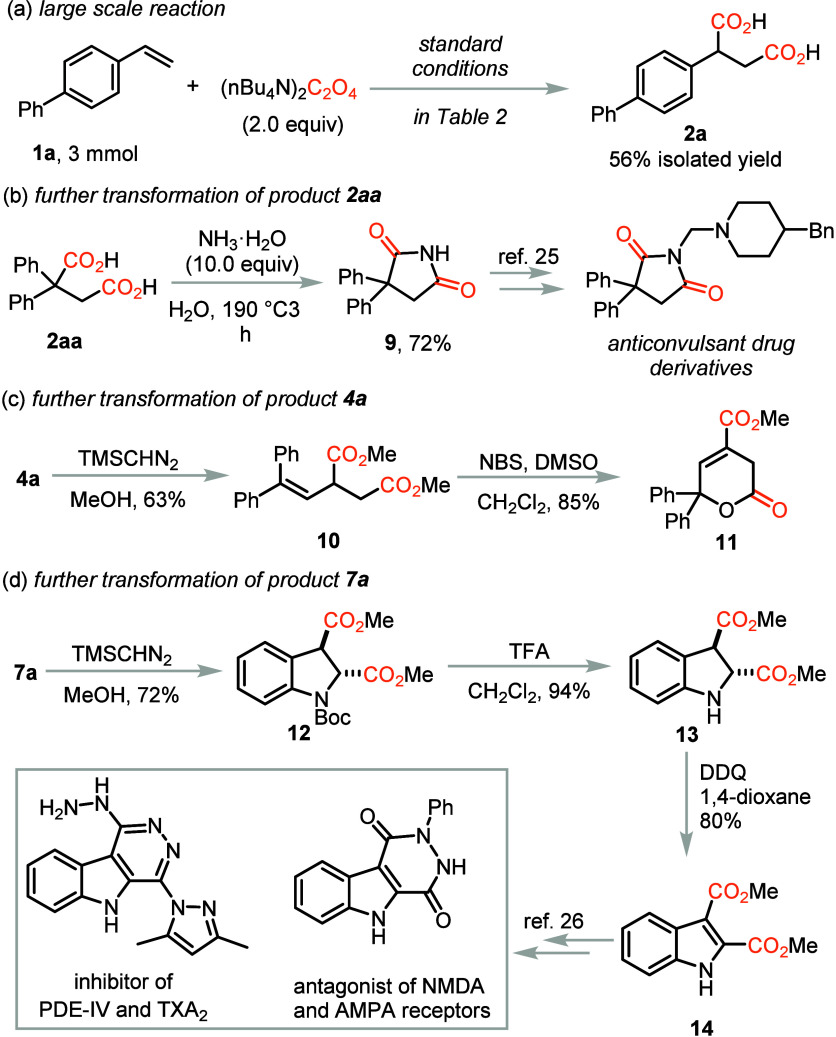
Applications of the Reaction

To gain further insights into the reaction mechanism, several control experiments were conducted, as shown in Scheme 3. To see if the reaction proceeded via the generation of radical species, TEMPO (2,2,6,6-tetramethylpiperidinyloxy) was added to the reaction with **1aa**. The dicarboxylation process was prohibited, and only a trace amount of **2aa** was detected. The TEMPO trapped adduct **15** was not detected, which might be due to the steric hindrance. To see if the electron was transferred directly from the oxalic dianion to the alkene substrate, the deuterium labeling experiment was conducted with D_2_O, as shown in Scheme 3b. The monocarboxylation product **8a** was obtained in 64% yield and 90% deuterium labeling ratio. Only deuteration on the benzyl position was observed, indicating formation of the benzyl anion intermediate during the transformation. It is worth noting that only 1.0 equiv of TBAO was utilized in this reaction, which means both of two electrons from the oxalic dianion were transferred to the final product and no electron was wasted during the transformation.

**Scheme 3:**
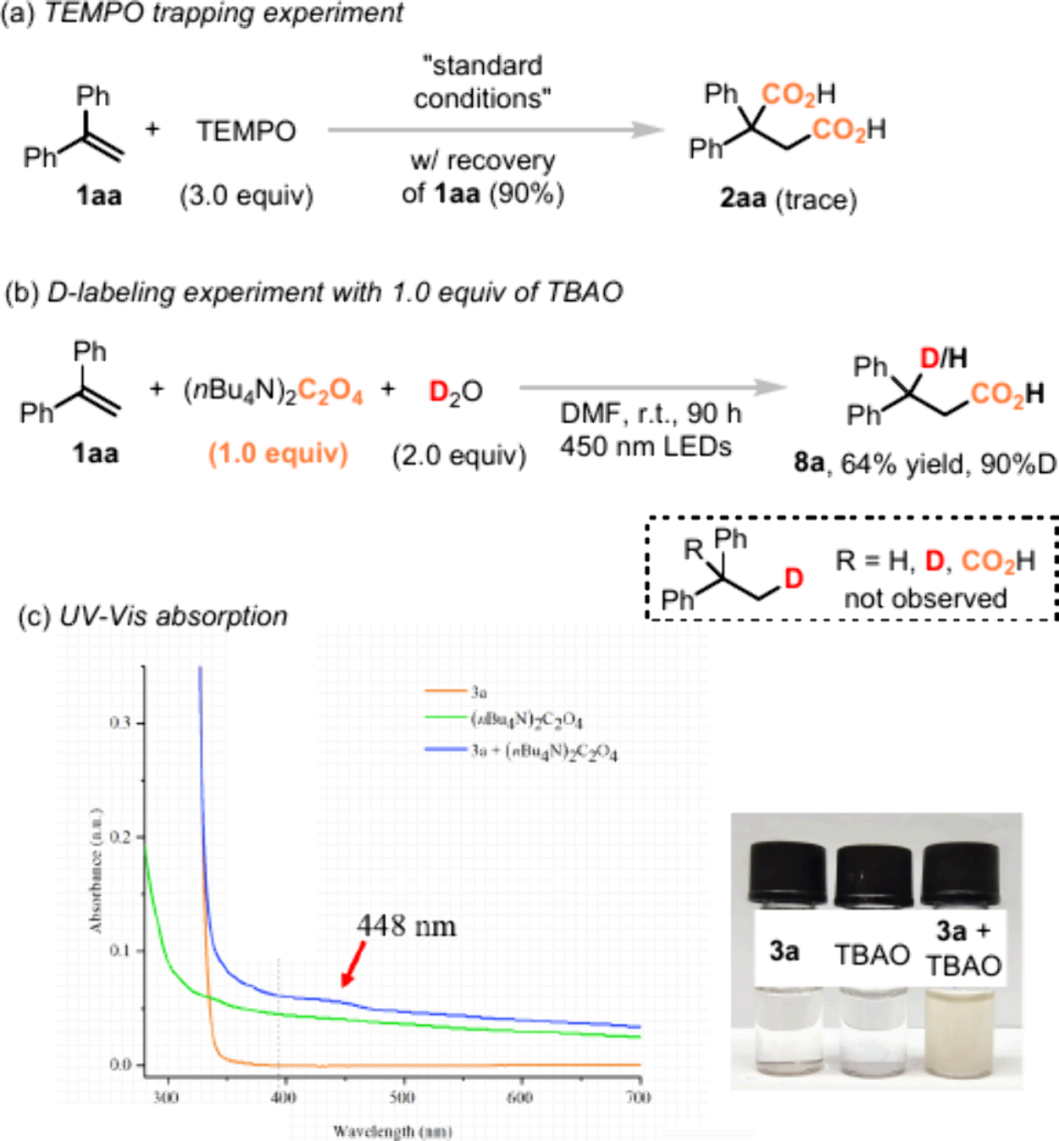
Mechanistic Studies

Combined with the phenomenon that simple monoaryl styrene provided no diacid product in the absence of photocatalyst, we could get the conclusion that the highly conjugated π-system is crucial to trigger the electron transfer process and cause the homolysis of the oxalic radical anion. A new CT or EDA complex was probably formed in this reaction. Indeed, the UV–vis absorption experiments showed that a bathochromic shift of the absorption band was observed (Scheme 3c, blue line). A new absorption peak at a wavelength of 448 nm was detected, meaning that a new complex was formed between the substrate and oxalate, which could be excited at a wavelength of 450 nm LEDs. In the meantime, the color change from colorless to yellow for the mixture of substrate **3a** and TBAO was also observed (Scheme 3c).

As shown in Scheme 4, on the basis of the above results, we proposed that the reaction was first initiated by photoexcitation of the EDA complex formed between alkene substrate and TBAO. The C—C bond of the oxalic dianion was fixed in the presence of highly conjugated substrate and not able to freely rotate, which decreased the energy barrier for single electron transfer. Afterward, one electron transfer from the oxalic dianion to the alkene substrate occurred to give the oxalic radical anion and the radical anion form of the alkene substrate (intermediate **I**). Subsequently, the homolysis of the oxalate radical anion generated CO_2_ and CO_2_^•–^, which underwent radical recombination to the intermediate **I** within the aggregate and established the first carboxy group, generating the benzylic anion intermediate **II**. Afterward, the benzyl anion intermediate **II** could trap the CO_2_ released from the oxalate and install the second carboxy group to give the diacid product **2aa**. In the presence of D_2_O, deuteration is more favorable than the second carboxylation process, producing deuterated carboxylic acid **8a** as the sole product.

**Scheme 4:**
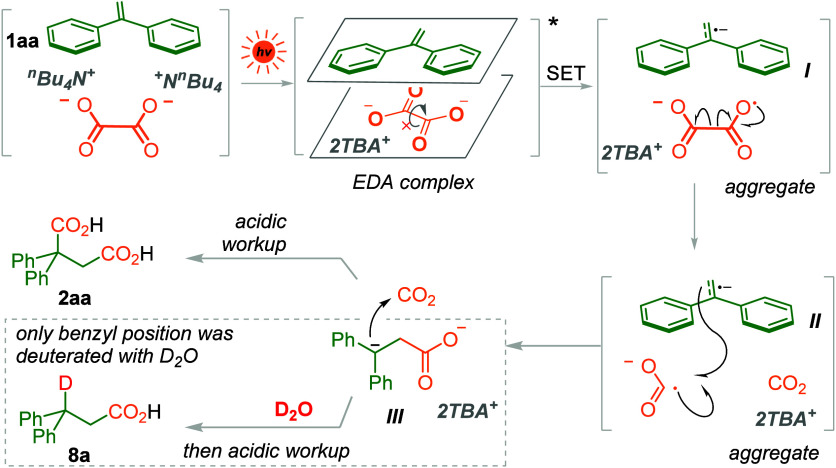
Proposed Mechanism for Dicarboxylation of Alkene **1aa**

In summary, a visible-light-induced additive-free alkene dicarboxylation reaction with TBAO was developed. Formation of the EDA complex between the oxalic dianion and the alkene substrate is crucial to triggering the intramolecular single electron transfer. The radical recombination of CO_2_^•–^ with the carbon-centered radical showcased a new way to establish the carboxyl group. This is the first example that oxalate acts as both the C1 source and reductant for alkene dicarboxylation via formation of an EDA complex in the absence of any additives. Further applications of TBAO in synthetic organic chemistry under photocatalytic conditions are currently underway in our laboratory.

## Data Availability

The data that support the findings of this study are available in the Supporting Information of this article.

## Supporting Information Available

Experimental procedures including optimization of reaction conditions, synthesis of substrates, acid derivatives, application of the reaction, control experiments, ^1^H and ^13^C NMR spectra, and mass data (PDF)
