# Supplementary material for: Visible-Light-Driven Carboxylative 1,2-Difunctionalization of C=C Bonds with Tetrabutylammonium Oxalate
Source: ACS Cent Sci. 2024 Nov 15;11(1):46–56. doi: 10.1021/acscentsci.4c01464 (PMC11758224; doi:10.1021/acscentsci.4c01464)

Supporting Information

Visible-Light-Driven Carboxylative 1,2-Difunctionalization of C=C Bonds with Tetrabutylammonium Oxalate

Sai Wang,[a,‡] Pei Xu,[a,‡] Zhi-Tao Liu,[a] Yi-Qin Liu,[a] Hao-Qiang Jiang,[a] Tian-Zi Hao,[a] Hui-Xian Jiang,[a] Hui Xu,[a] Xu-Dong Cao,[a,*] Dong Guo,[a,*] Xu Zhu[a,b,*]

[a]Jiangsu Key Laboratory of New Drug Research and Clinical Pharmacy, School of Pharmacy, Xuzhou Medical University 209 Tongshan Road, Xuzhou 221004, China

[b]Key Laboratory of Organic Synthesis of Jiangsu Province, College of Chemistry, Chemical Engineering and Materials Science, Soochow University, Suzhou, 215123, China

Email: ixudongcao@163.com; guo@xzhmu.edu.cn; xuzhu@xzhmu.edu.cn

Contents

1. General information S2

2. Optimization of the reaction conditions S3

    2.1 General procedure S3

    2.2 Procedure for large scale reaction: S4

3. Synthesis of substrates S6

4. Dicarboxylic acid derivatives S27

5. The application of the reaction S52

    6.1 TEMPO trapping S57

    6.2 D-labeling experiment S58

    6.3 Biaryl ethane with 1.0 equiv of TBAO S59

    6.4 UV-Vis absorption experiment S61

7. References S62

8. NMR spectra S63

1. General information

^{1}H NMR (400 MHz) spectra, ^{13}C NMR (100 MHz) spectra, and ^{19}F NMR (376 MHz) spectra were recorded on a JEOL ECZ400 (400 MHz) spectrometers in $CDCl_3$, CD_3OD or DMSO-d_6. The following abbreviations were used to explain the multiplicities: s = singlet, d = doublet, t = triplet, q = quartet, dd = doublet of doublet, td = triplet of doublet, ddd = doublet of doublet of doublet, sep = septet, m = multiplet, br = broad. Flash column chromatography was performed from Nuo Tai silica gel (Size: 200-300) with distilled solvents. High Resolution mass spectra were obtained from the Xuzhou Medical University Mass Spectral facility: Agilent G6550A Q-TOF (ESI).

All reactions were set up on the bench top and conducted under nitrogen atmosphere while subject to irradiation from blue LEDs (Xuzhou Aijia Electronic Technology Co., Ltd, AC220V, 45 W, λ_{max} = 450 nm). The material of the irradiation vessel is borosilicate glass. The distance from the light source to the irradiation vessel is 2 cm. Reagents, solvents, and photocatalysts were purchased from various vendors and used as received, unless stated otherwise. Thin-layer chromatography (TLC) was performed on 0.2-0.3 mm SiliCycle silica gel F-254 plates.

2. Optimization of the reaction conditions

2.1 General procedure:

An oven-dried tube (2 mL) containing a stirring bar was charged with the substrate (0.2 mmol, if solid) and then introduced in a glovebox, where it was charged with $(n\mathrm{Bu_4N})_2\mathrm{C_2O_4}$ (229.2 mg, 0.4 mmol, 2.0 equiv). The tube was taken out of the glovebox and connected to a Schlenk line where it was evacuated and back-filled with N_2 for 3 times. Then the substrate (0.2 mmol, if liquid) and dry DMF (1.5 mL) were added under nitrogen protection. Finally, the Schlenk tube was placed at the distance of 2 cm from two 45 W blue LEDs lamps (wavelength: 450 nm) and stirred at room temperature or 50 °C for 12-60 hours. After completion of the reaction, the reaction mixture was quenched with 4 mL of HCl (1 N), diluted with water (30 mL), and extracted with EtOAc (3 x 30 mL). The combined organic layers were washed with water and brine before dried over Na_2SO_4 and concentrated under reduced pressure. The residue was purified by silica gel flash chromatography (EtOAc/PE = 2/1 ~ 5/1) to give the pure desired product.

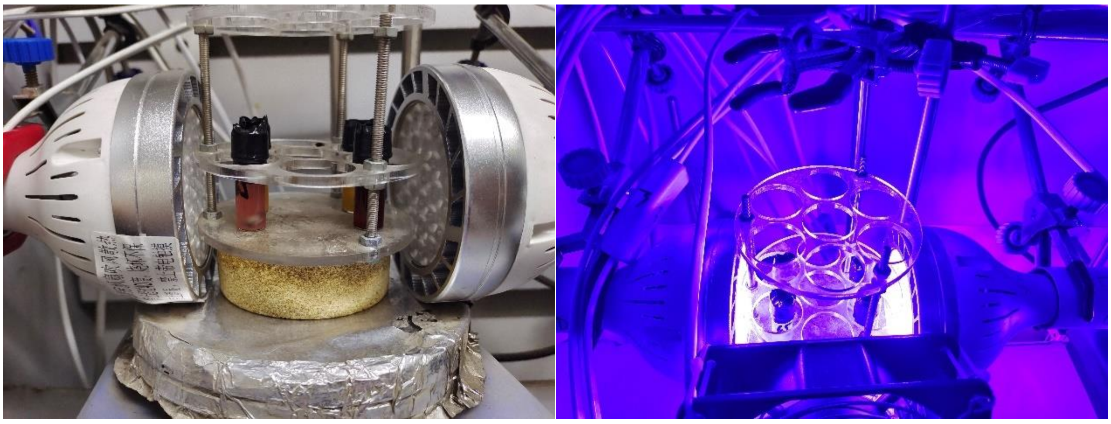

Figure S1. Standard setup for reactions at room temperature.

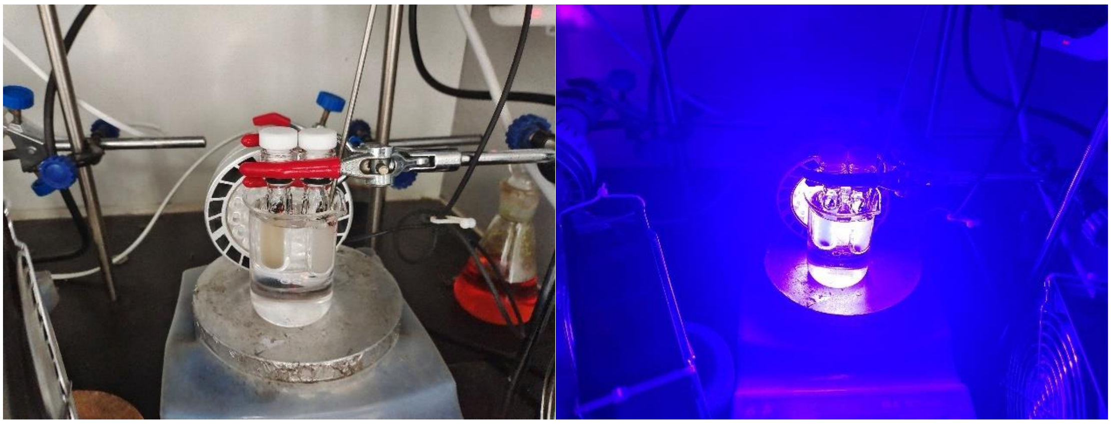

Figure S2. Standard setup for reactions at 50 °C.

Table S1. Screening table of the dicarboxylation of 4-vinylbiphenyl with oxalate.

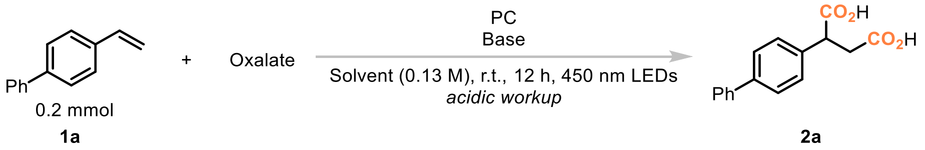

Entry	PC (mol%)	Oxalate (equiv)	Base (equiv)	Solvent (mL)	Wavelength (nm)	Yield (%)[a]
1	4DPAIPN (2)	$H_2C_2O_4$ (2.0)	TMG (5)	DMF (1.5)	450	0
2	4DPAIPN (2)	$Na_2C_2O_4$ (2.0)	-	DMF (1.5)	450	0
3	4DPAIPN (2)	$(NH_4)_2C_2O_4$ (2.0)	-	DMF (1.5)	450	0
4	4DPAIPN (2)	$(NMe_4)_2C_2O_4$ (2.0)	-	DMF (1.5)	450	0
5	4DPAIPN (2)	$(NEt_4)_2C_2O_4$ (2.0)	-	DMF (1.5)	450	0
6	4DPAIPN (2)	$(n\text{Bu}_4N)_2C_2O_4$ (2.0)	-	DMF (1.5)	450	55
7	4DPAIPN (2)	$(n\text{Bu}_4N)_2C_2O_4$ (2.0)	Cs_2CO_3 (5)	DMF (1.5)	450	42
8	4DPAIPN (2)	$(n\text{Bu}_4N)_2C_2O_4$ (2.0)	CsF (5)	DMF (1.5)	450	74
9	3DPAFIPN (2)	$(n\text{Bu}_4N)_2C_2O_4$ (2.0)	CsF (5)	DMF (1.5)	450	76
10	4CzIPN (2)	$(n\text{Bu}_4N)_2C_2O_4$ (2.0)	CsF (5)	DMF (1.5)	450	75
11	*fac*-Ir(ppy)$_3$ (2)	$(n\text{Bu}_4N)_2C_2O_4$ (2.0)	CsF (5)	DMF (1.5)	450	70
12	-	$(n\text{Bu}_4N)_2C_2O_4$ (2.0)	-	DMF (1.5)	450	76(73)[b]
13	-	$(n\text{Bu}_4N)_2C_2O_4$ (1.2)	-	DMF (1.5)	450	46
14	-	$(n\text{Bu}_4N)_2C_2O_4$ (1.5)	-	DMF (1.5)	450	60
15	-	$(n\text{Bu}_4N)_2C_2O_4$ (2.0)	-	DMA (1.5)	450	19
16	-	$(n\text{Bu}_4N)_2C_2O_4$ (2.0)	-	DMSO (1.5)	450	53
17	-	$(n\text{Bu}_4N)_2C_2O_4$ (2.0)	-	DMF (1.5)	410	40
18	-	$(n\text{Bu}_4N)_2C_2O_4$ (2.0)	-	DMF (1.5)	430	41
19	-	$(n\text{Bu}_4N)_2C_2O_4$ (2.0)	-	DMF (1.5)	460	52
20	-	$(n\text{Bu}_4N)_2C_2O_4$ (2.0)	-	DMF (1.5)	450	0[c]

[a]Yields were determined by ^{1}HNMR using 1, 2-dichloroethane as an internal standard. [b]Isolated yield. [c]No light.

2.2 Procedure for large scale reaction:

An oven-dried tube (50 mL) containing a stirring bar was charged with substrate **1a** (0.54g, 3.0 mmol) and then introduced into a glovebox, where it was charged with $(n\text{Bu}_4N)_2C_2O_4$ (3.44 g, 6.0 mmol, 2.0 equiv). The tube was taken out of the glovebox and connected to a Schlenk line where it was evacuated and back-filled with N_2 for 3

times. Then DMF (25 mL) were added via syringe under N_2 protection. Finally, the Schlenk tube was placed at a distance of 2 cm from two 45 W blue LEDs lamps (wavelength: λ_{max} = 450 nm) and stirred at room temperature for 48 hours. After completion of the reaction, the reaction mixture was quenched with 20 mL of HCl (1 N), diluted with water (30 mL) and extracted with EtOAc (3 x 50 mL). The combined organic layers were washed with water and brine before dried over Na_2SO_4 and concentrated under reduced pressure. The residue was purified by silica gel flash chromatography (EtOAc/PE = 5/1) to give the pure desired product in 56% yield (0.45 g).

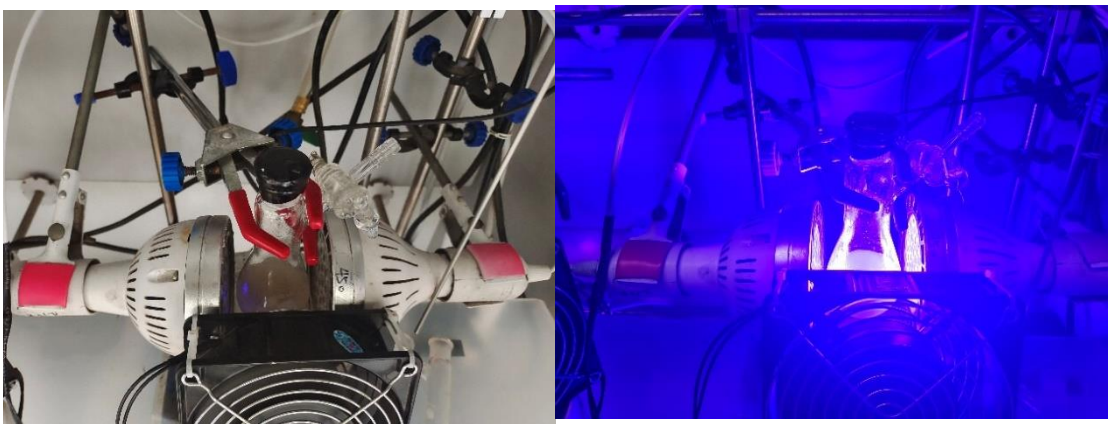

Figure S3. Standard setup for large reactions.

3. Synthesis of substrates

Scheme S1. The scope of various aryl alkenes and dienes.

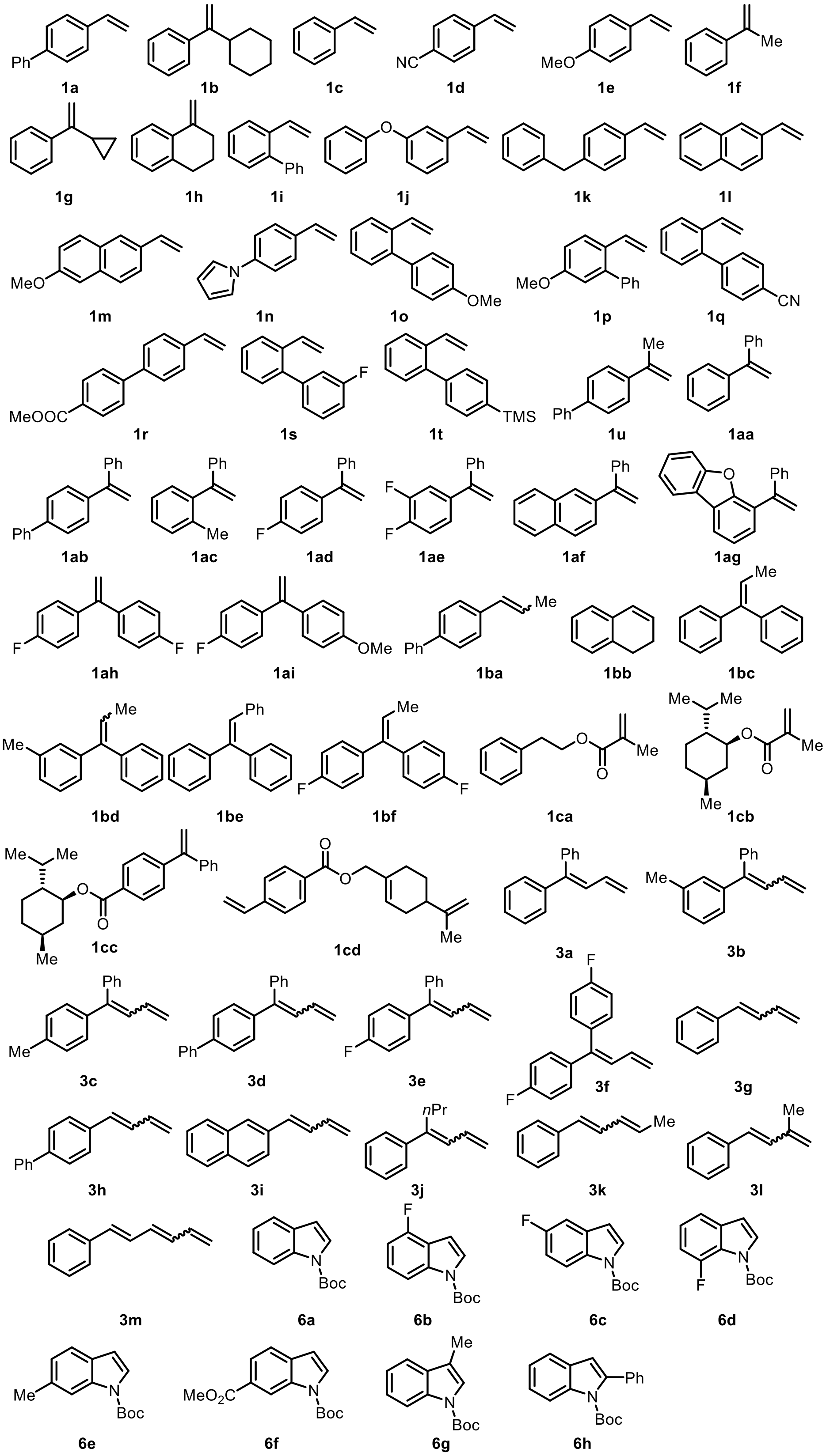

Substrates **1a, 1c, 1d, 1e, 1f, 1aa,** and **1bb** were purchased from commercial sources.

General procedure A: substrates **1b, 1g, 1h, 1j, 1l, 1m, 1n, 1u, 1ab, 1ac, 1ad, 1ae, 1af, 1ah, 1ai, 1ba, 1bc, 1bd, 1be, 1bf, 3c, 3d, 3g, 3h, 3i, 3j, 3k, 3l,** and **3m** were synthesized following the reported procedures in the reported literatures.[1]

To a stirred solution of the Witting reagents (10.0 mmol, 2.0 equiv) in THF (20 mL) at 0 °C was added NaHMDS (1.0 M in THF, 10 mmol, 2.0 equiv) or *t*BuOK (10.0 mmol, 2.0 equiv) dropwise. The reaction mixture was then stirred for 2 hours at room temperature. Ketone or aldehyde (5.0 mmol, 1.0 equiv) in THF (5 mL) was added dropwise to the cooled reaction mixture at 0 °C. The reaction was then stirred at room temperature for 12 hours, quenched with saturated NH_4Cl (20 mL), diluted with water (30 mL), and extracted with EtOAc (3 x 50 mL). The combined organic layers were washed with brine before dried over anhydrous Na_2SO_4 and concentrated under reduced pressure. The product was purified by flash column chromatography (eluent: 100% PE).

General procedure B: **1i, 1k, 1o, 1q, 1r, 1s,** and **1t** were synthesized following the reported procedures in the literature.[2]

A Schlenk tube was charged with phenylboronic acid derivatives (6.0 mmol, 1.2 equiv), K_2CO_3 (15.0 mmol, 3.0 equiv) and $Pd(PPh_3)_4$ (0.25 mmol, 5.0 mol%). The flask was next evacuated and backfilled with N_2 for 3 times, followed by addition of the corresponding bromide (5.0 mmol, 1.0 equiv), toluene (10 mL), EtOH (2.5 mL), and H_2O (10 mL). The reaction mixture was stirred at 80 °C for 12 hours until the starting material was fully consumed. It was cooled to room temperature, quenched with water (20 mL) and extracted with EtOAc (3 x 50 mL). The combined organic layers were washed with brine before dried over anhydrous Na_2SO_4 and concentrated under reduced pressure. The product was isolated by flash column chromatography.

General procedure C: **1p** was synthesized following the reported procedure in the literature.[1]

A Schlenk tube was charged with methyltriphenylphosphonium bromide (10.0 mmol, 2.0 equiv) and introduced into a glovebox, where it was charged with *t*BuOK (10.0 mmol, 2.0 equiv). The tube was taken out of the glovebox and THF (25 mL) were

added via syringe. The reaction mixture was then stirred for 2 hours at room temperature. 2-bromo-4-methoxybenzaldehyde (5.0 mmol, 1.0 equiv) was added dropwise to the cooled reaction mixture at 0 °C. The reaction was then stirred at room temperature for 12 hours, quenched with saturated NH_4Cl (20 mL), and extracted with EtOAc (3 x 50 mL). The combined organic layers were washed with brine before dried over anhydrous Na_2SO_4 and concentrated under reduced pressure.

A Schlenk tube was charged with phenylboronic acid (4.8 mmol, 1.2 equiv), K_2CO_3 (12.0 mmol, 3.0 equiv), and $Pd(PPh_3)_4$ (0.2 mmol, 5.0 mol%). The flask was next evacuated and backfilled with N_2 for 3 times, followed by addition of the 2-bromo-4-methoxy-1-vinylbenzene (4.0 mmol, 1.0 equiv), toluene (10 mL), EtOH (2.5 mL), and H_2O (10 mL). The reaction mixture was then stirred at 80 °C for 12 hours. It was cooled to room temperature, quenched with water (20 mL) and extracted with EtOAc (3 x 50 mL). The combined organic layers were washed with brine before dried over anhydrous Na_2SO_4 and concentrated under reduced pressure. The product was purified by flash column chromatography.

General procedure D: **1ag** was synthesized following the reported procedure in the literature.[1]

A Schlenk tube was charged with dibenzo[*b*,*d*]furan-4-ylboronic acid (5.0 mmol, 1.0 equiv) and $Pd(PPh_3)_2Cl_2$ (0.1 mmol, 2.0 mol%). The flask was next evacuated and backfilled with N_2 for 3 times. K_2CO_3 (10 mL, 2 M), α-bromostyrene (6.0 mmol, 1.5 equiv), and THF (15 mL) were added to the reaction mixture, which was stirred at 60 °C for 24 hours before was cooled to room temperature, quenched with water (20 mL) and extracted with EtOAc (3 x 50 mL). The combined organic layers were washed with brine before dried over anhydrous Na_2SO_4 and concentrated under reduced pressure. The product was purified by flash column chromatography (eluent: 100% PE).

General procedure E: **1ca** and **1cb** was synthesized following the reported procedures in the literature.[1]

The corresponding alcohol (5.0 mmol, 1.0 equiv) was dissolved in 20 mL dry

DCM. TEA (7.5 mmol, 1.5 equiv) was added to the reaction mixture which was stirred at room temperature for 15 mins. Methacryloyl chloride (6.0 mmol, 1.2 equiv) was added dropwise into the solution on an ice bath. After the addition was completed, the reaction was warmed to room temperature and stirred for 24 hours. Afterward, the reaction was quenched with water (20 mL) and extracted with EtOAc (3 x 50 mL). The combined organic layers were washed with brine before dried over anhydrous Na_2SO_4 and concentrated under reduced pressure. The product was isolated by flash column chromatography (eluent: EtOAc/PE = 1/50).

General procedure F: **1cc** and **1cd** was synthesized following the reported procedure in the literature. [1]

Acid (1.0 mmol, 1.0 equiv), DCC (1.5 mmol, 1.2 equiv) and DMAP (*N*,*N*-dimethylpyridin-4-amine, 0.2 mmol, 0.2 equiv) were dissolved in DCM (10 mL) in a 25 mL round-bottom flask. Afterward, the corresponding alcohol (1.2 mmol, 1.2 equiv) was added and the resulting reaction mixture was stirred for 12 hours at room temperature. The reaction was monitored by TLC to ensure full conversion of the starting material. Afterward, the reaction was extracted with DCM (3 x 50 mL). The combined organic layers were washed with brine before dried over anhydrous Na_2SO_4 and concentrated under reduced pressure. The product was purified by flash column chromatography (eluent: EtOAc/PE = 1/50).

General procedure G: **3a, 3b, 3e, 3f,** and **3j** were synthesized following the reported procedures in the literatures. [3]

To a solution of carbonyl compounds (5.0 mmol, 1.0 equiv) in dry THF (20 mL) was added allylmagnesium bromide (1 M in THF, 11.0 mmol, 2.2 equiv) under a nitrogen atmosphere at room temperature. The mixture was next stirred for about 1 hour until full consumption of the carbonyl compounds (monitored by TLC). Then diphenyl phosphite (6.0 mmol, 1.2 equiv) was added. To the reaction mixture, which was stirred for 2 hours and then quenched with water. The resulting crude mixture was extracted with dichloromethane (3 x 50 mL), and washed with brine before dried over anhydrous

Na_2SO_4 and concentrated under reduced pressure.. The product was purified by flash column chromatography (eluent: 100% PE).

General procedure H: **6a, 6b, 6c, 6d, 6e, 6f, 6g,** and **6h** was synthesized following the reported procedure in the literature. [1]

To the THF solution of indole derivatives (2.0 mmol, 1.0 equiv), DMAP (0.2 mmol, 10.0 mol%) and Boc anhydride (2.2 mmol, 1.1 equiv) were added and the solution was stirred under room temperature until fully conversion of the starting material. The reaction mixture was then quenched by saturated sodium bicarbonate solution (20 mL) and extracted by ethyl acetate (3 x 50 mL). The combined organic layers were washed with brine before dried over anhydrous Na_2SO_4 and concentrated under reduced pressure. The product was purified by flash column chromatography (eluent: EtOAc/PE 1/20).

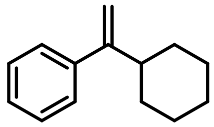

(1-cyclohexylvinyl)benzene (1b): Prepared according to general procedure A using cyclohexyl(phenyl)methanone and methyltriphenylphosphonium bromide. The spectra matched with reported literature.[1]
^{1}H NMR (400 MHz, $CDCl_3$) δ 7.36 – 7.28 (m, 5H), 5.13 (s, 1H), 5.00 (s, 1H), 2.42 (t, J = 11.2 Hz, 1H), 1.88 – 1.74 (m, 4H), 1.74 – 1.66 (m, 1H), 1.31 (m, 2H), 1.22 – 1.11 (m, 3H).
^{13}C NMR (100 MHz, $CDCl_3$) δ 155.2, 143.1, 128.3, 127.1, 126.8, 110.5, 42.7, 32.8, 27.0, 26.6.

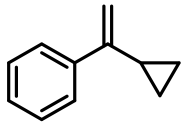

(1-cyclopropylvinyl)benzene (1g): Prepared according to general procedure A using cyclopropyl(phenyl)methanone and methyltriphenylphosphonium bromide. The spectra matched with reported literature.[1]
^{1}H NMR (400 MHz, $CDCl_3$) δ 7.63 – 7.57 (m, 2H), 7.38 – 7.27 (m, 3H), 5.28 (s, 1H), 4.94 (s, 1H), 1.70 – 1.60 (m, 1H), 0.86 – 0.83 (m, 2H), 0.63 – 0.57 (m, 2H).
^{13}C NMR (100 MHz, $CDCl_3$) δ 149.5, 141.8, 128.3, 127.6, 126.3, 109.2, 15.8, 6.8.

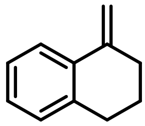

1-methylene-1,2,3,4-tetrahydronaphthalene (1h): Prepared according to general procedure A using 3,4-dihydronaphthalen-1(2*H*)-one and methyltriphenylphosphonium bromide. The spectra matched with reported literature.[4]

^{1}H NMR (400 MHz, $CDCl_3$) δ 7.83 – 7.57 (m, 1H), 7.22 – 7.03 (m, 3H), 5.47 (d, *J* = 1.6 Hz, 1H), 4.95 (d, *J* = 1.6 Hz, 1H), 2.84 (t, *J* = 6.4 Hz, 2H), 2.61 – 2.43 (m, 2H), 1.92 – 1.83 (m, 2H).

^{13}C NMR (100 MHz, $CDCl_3$) δ 143.6, 137.5, 134.8, 129.3, 127.7, 126.0, 124.3, 108.0, 33.4, 30.6, 23.9, 18.7, 14.9.

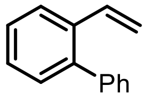

2-vinyl-1,1'-biphenyl (1i): Prepared according to general procedure B from 2-bromostyrene and phenylboronic acid. The spectra matched with reported literature. [5]

^{1}H NMR (400 MHz, $CDCl_3$) δ 7.69 – 7.54 (m, 1H), 7.49 – 7.27 (m, 8H), 6.78 – 6.66 (dd, *J* = 17.2, 10.8 Hz, 1H), 5.70 (d, *J* = 17.6 Hz, 1H), 5.19 (d, *J* = 10.8 Hz, 1H).

^{13}C NMR (100 MHz, $CDCl_3$) δ 141.0, 136.0, 130.2, 129.9, 128.9, 128.1, 127.8, 127.6, 127.2, 127.1, 125.8, 114.7.

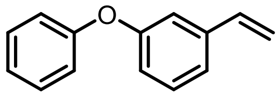

1-phenoxy-3-vinylbenzene (1j): Prepared according to general procedure A from 3-phenoxybenzaldehyde and methyltriphenylphosphonium bromide. The spectra matched with reported literature. [6]

^{1}H NMR (400 MHz, $CDCl_3$) δ 7.47 – 6.84 (m, 9H), 6.66 (dd, *J* = 17.6, 10.8 Hz, 1H), 5.71 (d, *J* = 17.6 Hz, 1H), 5.25 (d, *J* = 10.8 Hz, 1H).

^{13}C NMR (100 MHz, $CDCl_3$) δ 157.6, 157.3, 139.6, 136.4, 129.9(2), 129.8(9), 123.4, 121.5, 119.0, 118.4, 116.6, 114.8.

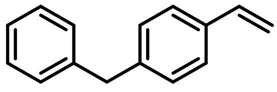

1-benzyl-4-vinylbenzene (1k): Prepared according to general procedure B from bromobenzene and 4-vinylphenylboronic acid. The spectra matched with reported liter ature. [7]

^{1}H NMR (400 MHz, $CDCl_3$) δ 7.32 – 7.27 (m, 4H), 7.23 – 7.10 (m, 5H), 6.67 (dd, *J* = 18.0, 10.8 Hz, 1H), 5.68 (d, *J* = 17.6 Hz, 1H), 5.17 (d, *J* = 11.2 Hz, 1H), 3.94 (s, 2H).

^{13}C NMR (100 MHz, CDCl$_3$) δ 141.1, 140.9, 136.7, 135.6, 129.2, 129.0,128.5, 126.5, 126.2, 113.3, 41.8.

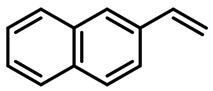

2-vinylnaphthalene (1l): Prepared according to general procedure A from 2-naphthaldehyde and methyltriphenylphosphonium bromide. The spectra matched with reported literature. [8]
^{1}H NMR (400 MHz, CDCl$_3$) δ 7.82 – 7.68 (m, 4H), 7.66 – 7.57 (m, 1H), 7.48 – 7.36 (m, 2H), 6.86 (dd, J = 17.6, 10.8 Hz, 1H), 5.86 (d, J = 17.6 Hz, 1H), 5.32 (d, J = 11.2 Hz, 1H).
^{13}C NMR (100 MHz, CDCl$_3$) δ 137.1, 135.1, 133.7, 133.3, 128.3, 128.2, 127.8, 126.5, 126.4, 126.1, 123.3, 114.3.

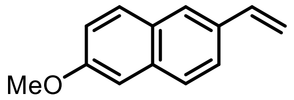

2-methoxy-6-vinylnaphthalene (1m): Prepared according to general procedure A from 6-methoxy-2-naphthaldehyde and methyltriphenylphosphonium bromide. The spectra matched with reported literature. [9]
^{1}H NMR (400 MHz, CDCl$_3$) δ 7.69 – 7.60 (m, 3H), 7.60 – 7.51 (m, 1H), 7.14 – 7.06 m, 2H), 6.80 (dd, J = 17.6, 13.2 Hz, 1H), 5.79 (d, J = 17.6 Hz, 1H), 5.25 (d, J = 11.2 Hz, 1H), 3.85 (s, 3H).
^{13}C NMR (100 MHz, CDCl$_3$) δ 157.9, 137.1, 134.4, 133.0, 129.7, 129.0, 127.1, 126.3, 123.8, 119.1, 113.2, 105.9, 55.3.

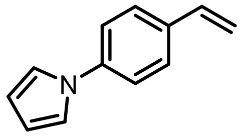

1-(4-vinylphenyl)-1*H*-pyrrole (1n): Prepared according to general procedure A from 4-(1*H*-pyrrol-1-yl)benzaldehyde and methyltriphenylphosphonium bromide.
^{1}H NMR (400 MHz, CDCl$_3$) δ 7.50 – 7.35 (m, 2H), 7.33 – 7.24 (m, 2H), 7.07 – 7.04 (m, 2H), 6.68 (dd, J = 17.6, 11.2 Hz, 1H), 6.36 – 6.32 (m, 2H), 5.70 (d, J = 17.6 Hz, 1H), 5.23 (d, J = 11.2 Hz, 1H).
^{13}C NMR (100 MHz, CDCl$_3$) δ 140.2, 135.9, 135.0, 127.4, 120.4, 119.2, 113.8, 110.6.

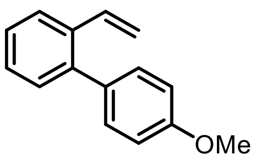

4'-methoxy-2-vinyl-1,1'-biphenyl (1o): Prepared according to general procedure B from 2-bromostyrene and 4-methoxyphenylboronic acid.

^{1}H NMR (400 MHz, CDCl$_3$) δ 7.65 – 7.59 (m, 1H), 7.36 – 7.23 (m, 5H), 6.97 – 6.92 (m, 2H), 6.73 (dd, J = 17.6, 10.8 Hz, 1H), 5.69 (d, J = 17.6 Hz, 1H), 5.18 (d, J = 11.2 Hz, 1H), 3.86 (s, 3H)
^{13}C NMR (100 MHz, CDCl$_3$) δ 158.9, 140.6, 136.2, 136.0, 133.3, 131.0, 130.3, 127.8, 127.3, 125.9, 114.6, 113.6, 55.4.
HRMS (ESI-TOF): *m/z* Calcd. For $C_{15}H_{14}O$: $(M+H)^+$ 211.117 Found: 211.1117.

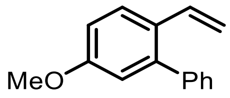

5-methoxy-2-vinyl-1,1'-biphenyl (1p): Prepared according to general procedure C.
^{1}H NMR (400 MHz, CDCl$_3$) δ 7.61 – 7.54 (m, 1H), 7.42 – 7.28 (m, 5H), 6.92 – 6.78 (m, 2H), 6.63 (dd, J = 17.6, 10.8 Hz, 1H), 5.58 (m, J = 17.6 Hz, 1H), 5.08 (d, J = 10.4 Hz, 1H), 3.83 (s, 3H).
^{13}C NMR (100 MHz, CDCl$_3$) δ 159.1, 142.2, 140.9, 135.3, 129.8, 128.6, 128.1, 127.2, 127.0, 114.9, 113.7, 112.7, 55.3.
HRMS (ESI-TOF): *m/z* Calcd. For $C_{15}H_{14}O$: $(M+H)^+$ 211.1117 Found: 211.1117.

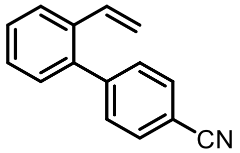

2'-vinyl-[1,1'-biphenyl]-4-carbonitrile (1q): Prepared according to general procedure B from 2-bromostyrene and (4-cyanophenyl)boronic acid.
^{1}H NMR (400 MHz, CDCl$_3$) δ 7.73 – 7.62 (m, 3H), 7.49 – 7.33 (m, 4H), 7.28 – 7.25 (m, 1H), 6.60 (dd, J = 17.2, 10.8 Hz, 1H), 5.72 (d, J = 17.6 Hz, 1H), 5.25 (d, J = 10.8 Hz, 1H).
^{13}C NMR (100 MHz, CDCl$_3$) δ 145.8, 138.9, 135.9, 135.2, 132.0, 130.7, 129.8, 128.7, 128.1, 126.3, 119.1, 116.2, 111.0.
HRMS (ESI-TOF): *m/z* Calcd. For $C_{15}H_{11}N$: $(M+Na)^+$ 228.0784 Found: 228.0784.

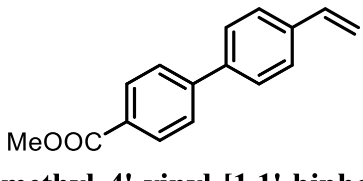

methyl 4'-vinyl-[1,1'-biphenyl]-4-carboxylate (1r): Prepared according to general procedure B from 4-bromostyrene and (4-(methoxycarbonyl)phenyl)boronic acid.
^{1}H NMR (400 MHz, CDCl$_3$) δ 8.09 (d, J = 8.4 Hz, 2H), 7.71 – 7.42 (m, 6H), 6.75 (dd, J = 20.4, 11.2 Hz, 1H), 5.81 (d, J = 17.6 Hz, 1H), 5.30 (d, J = 11.2 Hz, 1H), 3.93 (s, 3H).
^{13}C NMR (100 MHz, CDCl$_3$) δ 167.1, 145.1, 139.3, 137.5, 136.2, 130.2, 128.9, 127.5, 126.9, 114.6, 52.2.
HRMS (ESI-TOF): *m/z* Calcd. For $C_{16}H_{14}O_2$: $(M+H)^+$ 239.1067 Found: 239.1065.

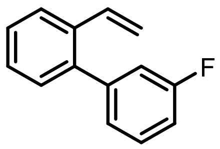

3'-fluoro-2-vinyl-1,1'-biphenyl (1s): Prepared according to general procedure B from 2-bromostyrene and (3-fluorophenyl)boronic acid.
^{1}H NMR (400 MHz, CDCl$_3$) δ 7.62 (dd, J = 7.6, 1.6 Hz, 1H), 7.40 – 7.20 (m, 4H), 7.15 – 6.97 (m, 3H), 6.68 (dd, J = 17.6, 11.2 Hz, 1H), 5.69 (dd, J = 17.6, 1.2 Hz, 1H), 5.19 (dd, J = 11.2, 1.2 Hz, 1H).
^{13}C NMR (100 MHz, CDCl$_3$) δ 162.6 (d, J_{FC} = 244.8 Hz), 143.2 (d, J_{FC} = 7.6 Hz), 139.6, 135.9, 135.7, 130.0, 129.6 (d, J_{FC} = 8.4 Hz), 128.0, 127.9, 126.0, 125.7 (d, J_{FC} = 2.8 Hz), 116.9 (d, J_{FC} = 21.3 Hz), 115.3, 114.0 (d, J_{FC} = 20.8 Hz).
^{19}F NMR (376 MHz, CDCl$_3$) δ -114.9.

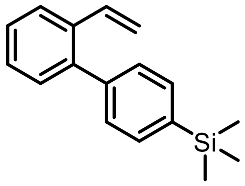

trimethyl(2'-vinyl-[1,1'-biphenyl]-4-yl)silane (1t): Prepared according to general procedure B from 2-bromostyrene and (4-(trimethylsilyl)phenyl)boronic acid.
^{1}H NMR (400 MHz, CDCl$_3$) δ 7.39 – 7.47 (m, 3H), 7.25 – 7.06 (m, 5H), 6.60 (dd, J = 17.6, 11.2 Hz, 1H), 5.52 (d, J = 17.6 Hz, 1H), 4.99 (d, J = 10.8 Hz, 1H), 0.15 (s, 9H).
^{13}C NMR (100 MHz, CDCl$_3$) δ 141.4, 140.9, 139.0, 136.1, 135.9, 133.2, 130.3, 129.3, 127.8, 127.6, 125.9, 114.7.-0.9.

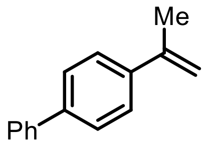

4-(prop-1-en-2-yl)-1,1'-biphenyl (1u): Prepared according to general procedure A from 1-([1,1'-biphenyl]-4-yl)ethan-1-one and methyltriphenylphosphonium bromide. The spectra matched with reported literature. [10]
^{1}H NMR (400 MHz, CDCl$_3$) δ 7.64 – 7.52 (m, 6H), 7.47 – 7.40 (m, 2H), 7.38 – 7.30 (m, 1H), 5.43 (s, 1H), 5.11 (s, 1H), 2.19 (s, 3H).
^{13}C NMR (100 MHz, CDCl$_3$) δ 142.9, 140.9, 140.3, 140.2, 128.9, 127.4, 127.1(3), 127.0(7), 126.0, 112.6, 21.9.

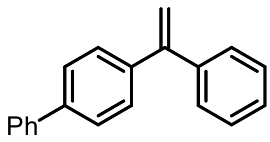

4-(1-phenylvinyl)-1,1'-biphenyl (1ab): Prepared according to general procedure A from [1,1'-biphenyl]-4-yl(phenyl)methanone and methyltriphenylphosphonium

bromide. The spectra matched with reported literature. [1]

^{1}H NMR (400 MHz, CDCl$_3$) δ 7.71 – 7.52 (m, 4H), 7.51 – 7.29 (m, 10H), 5.52 (s, 1H), 5.47 (s, 1H).

^{13}C NMR (100 MHz, CDCl$_3$) δ 149.8, 141.6, 140.9, 140.7, 140.6, 128.9, 128.8, 128.5, 128.4, 127.9, 127.5, 127.2, 127.0, 114.5.

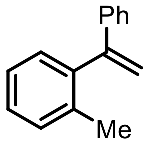

1-methyl-2-(1-phenylvinyl)benzene (1ac): Prepared according to general procedure A from phenyl(*o*-tolyl)methanone and methyltriphenylphosphonium bromide. The spectra matched with reported literature. [1]

^{1}H NMR (400 MHz, CDCl$_3$) δ 7.97 – 6.83 (m, 9H), 5.77 (d, J = 1.2 Hz, 1H), 5.20 (d, J = 1.2 Hz, 1H), 2.05 (s, 3H).

^{13}C NMR (100 MHz, CDCl$_3$) δ 149.6, 141.8, 140.7, 136.2, 130.2(1), 130.1(5), 128.5, 127.7(0), 127.6(7), 126.6, 125.8, 115.0, 20.3.

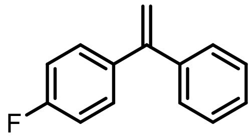

1-fluoro-4-(1-phenylvinyl)benzene (1ad): Prepared according to general procedure A from (4-fluorophenyl)(phenyl)methanone and methyltriphenylphosphonium bromide. The spectra matched with reported literature. [1]

^{1}H NMR (400 MHz, CDCl$_3$) δ 7.39 – 7.28 (m, 7H), 7.02 (t, J = 8.8 Hz, 2H), 5.42 (s, 1H) 5.40 (s, 1H).

^{13}C NMR (100 MHz, CDCl$_3$) δ 162.6 (d, J_{FC} = 245.6 Hz), 149.1, 141.4, 137.6 (d, J_{FC} = 3.3 Hz), 130.0 (d, J_{FC} = 8.0 Hz), 128.4, 128.3, 128.0, 115.1 (d, J_{FC} = 21.3 Hz), 114.3.

^{19}F NMR (376 MHz, CDCl$_3$) δ -114.6.

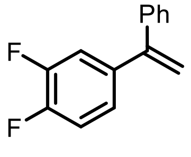

1,2-difluoro-4-(1-phenylvinyl)benzene (1ae): Prepared according to general procedure A from (3,4-difluorophenyl)(phenyl)methanone and methyltriphenylphosphonium bromide. The spectra matched with reported literature. [1]

^{1}H NMR (400 MHz, CDCl$_3$) δ 7.47 – 7.27 (m, 5H), 7.23 – 6.93 (m, 3H), 5.46 (s, 1H), 5.43 (s, 1H).

^{13}C NMR (100 MHz, CDCl$_3$) δ 151.4 (d, J_{FC} = 12.2 Hz), 149.0 (dd, J_{FC} = 12.7, 3.2 Hz), 148.4, 140.8, 138.7 (t, J_{FC} = 4.9 Hz), 128.5, 128.3, 128.2, 124.4 (q, J_{FC} = 3.4 Hz), 117.3 (d, J_{FC} = 17.6 Hz), 117.1 (d, J_{FC} =17.0 Hz), 115.2.

^{19}F NMR (376 MHz, CDCl$_3$) δ -137.9, -139.0.

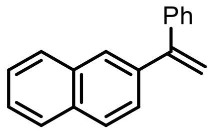

2-(1-phenylvinyl)naphthalene (1af): Prepared according to general procedure A from naphthalen-2-yl(phenyl)methanone and methyltriphenylphosphonium bromide. The spectra matched with reported literature. [1]

^{1}H NMR (400 MHz, CDCl$_3$) δ 7.37– 7.11 (m, 8H), 7.13 (d, J = 17.6 Hz, 2H), 6.96 – 6.59 (m, 2H), 5.80 (s, 1H), 5.36 (s, 1H).

^{13}C NMR (100 MHz, CDCl$_3$) δ 150.2, 141.6, 139.0, 133.4, 133.1, 128.5, 128.4, 128.3, 128.0, 127.8, 127.7, 127.4, 126.5, 126.3, 126.2, 115.0.

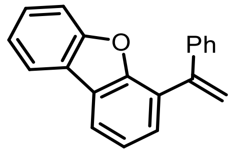

4-(1-phenylvinyl)dibenzo[*b*,*d*]furan (1ag): Prepared according to general procedure D from dibenzo[*b*,*d*]furan-4-ylboronic acid and α-bromostyrene.

^{1}H NMR (400 MHz, CDCl$_3$) δ 7.99 – 7.85 (m, 2H), 7.50 (d, J = 8.4 Hz, 1H), 7.44 – 7.28 (m, 9H), 5.88 (s, 2H).

^{13}C NMR (100 MHz, CDCl$_3$) δ 156.2, 154.1, 144.7, 141.1, 128.4(3), 128.3(8), 127.9, 127.8, 127.3, 126.1, 124.7, 124.3, 122.9, 122.8, 120.7, 120.1, 117.8, 112.0.

HRMS (ESI-TOF): *m/z* Calcd. For $C_{20}H_{14}O$: 271.1117. Found: 271.1126.

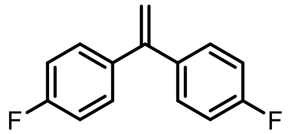

4,4'-(ethene-1,1-diyl)bis(fluorobenzene) (1ah): Prepared according to general procedure A from bis(4-fluorophenyl)methanone and methyltriphenylphosphonium bromide. The spectra matched with reported literature. [1]

^{1}H NMR (400 MHz, CDCl$_3$) δ 7.32 – 7.26 (m, 4H), 7.07 – 6.98 (m, 4H), 5.39 (s, 2H).

^{13}C NMR (100 MHz, CDCl$_3$) δ 162.7 (d, J_{FC} = 245.8 Hz), 148.2, 137.5 (d, J_{FC} = 3.2 Hz), 129.9 (d, J_{FC} = 8.0 Hz), 115.2 (d, J_{FC} = 21.2 Hz), 114.2.

^{19}F NMR (376 MHz, CDCl$_3$) δ -113.9.

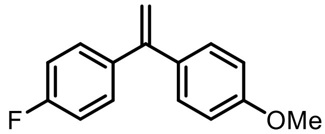

1-fluoro-4-(1-(4-methoxyphenyl)vinyl)benzene (1ai): Prepared according to general procedure A from (4-fluorophenyl)(4-methoxyphenyl)methanone and methyltriphenylphosphonium bromide. The spectra matched with reported literature. [1]

^{1}H NMR (400 MHz, CDCl$_3$) δ 7.33 – 7.28 (m, 2H), 7.27 – 7.23 (m, 2H), 7.01 (t, J = 8.8 Hz, 2H), 6.87 (d, J = 8.8 Hz, 2H), 5.37 (s, 1H), 5.31 (s, 1H), 3.83 (s, 3H).
^{13}C NMR (100 MHz, CDCl$_3$) δ 162.6 (d, J_{FC} = 244.6 Hz), 159.5, 148.6, 138.0 (d, J_{FC} = 3.6 Hz), 133.9, 130.0 (d, J_{FC} = 8.0 Hz), 129.4, 115.1 (d, J_{FC} = 21.1 Hz), 113.7, 112.9, 55.3.
^{19}F NMR (376 MHz, CDCl$_3$) δ -114.8.

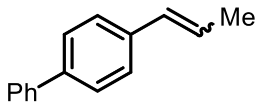

4-(prop-1-en-1-yl)-1,1'-biphenyl (1ba, mixture of isomers, *E/Z* = 1:1): Prepared according to general procedure A from [1,1'-biphenyl]-4-carbaldehyde and ethyltriphenylphosphonium bromide. The spectra matched with reported literature. [11]
^{1}H NMR (400 MHz, CDCl$_3$) δ 6.48 – 6.38 (m, 3H), 6.38 – 6.33 (m, 1H), 6.29 – 6.19 (m, 4H), 6.19 – 6.11 (m, 1H), 5.35 – 5.21 (m, 1H), 5.15 – 5.06 (m, 0.50H), 4.69 – 4.61 (m, 0.50H), 0.78 (dd, J = 7.2, 2.0 Hz, 1.50H), 0.73 (dd, J = 6.4, 1.6 Hz, 1.50H).
^{13}C NMR (100 MHz, CDCl$_3$) δ 141.0, 139.6, 139.3, 137.1, 136.8, 130.7, 129.6, 129.4, 128.9(1), 128.8(8), 127.3, 127.3(3), 127.3(1), 127.2, 127.1, 127.0(1), 126.9(5), 126.4, 126.0, 18.7, 14.9.

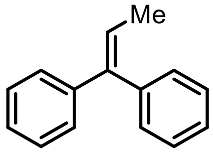

prop-1-ene-1,1-diyldibenzene (1bc): Prepared according to general procedure A from benzophenone and ethyltriphenylphosphonium bromide. The spectra matched with reported literature.[1]
^{1}H NMR (400 MHz, CDCl$_3$) δ 7.37 (tt, J = 7.6, 1.2 Hz, 2H), 7.32 – 7.16 (m, 8H), 6.17 (q, J = 7.2 Hz, 1H), 1.76 (d, J = 7.2 Hz, 3H).
^{13}C NMR (100 MHz, CDCl$_3$) δ 143.1, 142.6, 140.2, 130.2, 128.3, 128.2, 127.3, 127.0, 126.9, 124.3, 15.9.

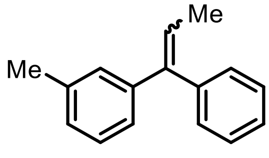

1-methyl-4-(1-phenylvinyl)benzene (1bd): Prepared according to general procedure A from phenyl(*m*-tolyl)methanone and ethyltriphenylphosphonium bromide. The spectra matched with reported literature. [1]
^{1}H NMR (400 MHz, CDCl$_3$) δ 7.42 – 7.08 (m, 7H), 7.03 – 6.97 (m, 2H), 6.15 (q, J = 7.2 Hz, 1H), 2.32 (d, J = 22.4 Hz, 3H), 1.75 (dd, J = 6.8, 2.4 Hz, 3H).
^{13}C NMR (100 MHz, CDCl$_3$) δ 143.2, 143.1, 142.7, 140.2, 140.1, 137.8, 137.7, 130.8, 130.2, 128.2(3), 128.1(7), 128.1(3), 128.0(9), 128.0, 127.7(0), 127.6(5), 127.3(2), 127.2(8), 126.9, 126.8, 124.6, 124.1, 21.6, 15.9, 15.8.

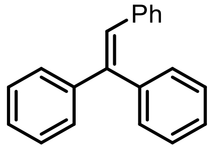

ethene-1,1,2-triyltribenzene (1be): Prepared according to general procedure A from benzophenone and benzyltriphenylphosphonium bromide. The spectra matched with reported literature. [1]

^{1}H NMR (400 MHz, CDCl$_3$) δ 7.37 – 7.26 (m, 8H), 7.23 – 7.08 (m, 5H), 7.06 – 6.99 (m, 2H), 6.97 (s, 1H).

^{13}C NMR (100 MHz, CDCl$_3$) δ 143.6, 142.8, 140.5, 137.6, 130.6, 129.7, 128.8, 128.4, 128.3, 128.1, 127.8, 127.7, 127.6, 126.9.

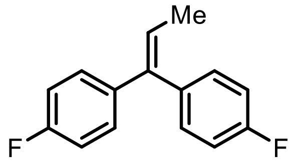

4,4'-(prop-1-ene-1,1-diyl)bis(fluorobenzene) (1bf): Prepared according to general procedure A from bis(4-fluorophenyl)methanone and ethyltriphenylphosphonium bromide. The spectra matched with reported literature. [1]

^{1}H NMR (400 MHz, CDCl$_3$) δ 7.24 – 7.02 (m, 6H), 6.93 (t, J = 6.4 Hz, 2H), 6.09 (q, J = 7.2 Hz, 1H), 1.73 (d, J = 7.2 Hz, 3H).

^{13}C NMR (100 MHz, CDCl$_3$) δ 162.1 (d, J_{FC} = 244.5 Hz), 162.0 (d, J_{FC} = 244.6 Hz), 140.6, 139.1 (d, J_{FC} = 3.1 Hz), 135.8 (d, J_{FC} = 3.4 Hz), 131.7 (d, J_{FC} = 7.9 Hz), 128.8 (d, J_{FC} = 7.9 Hz), 124.5, 115.3 (d, J_{FC} = 21.1 Hz), 115.1 (d, J_{FC} = 21.1 Hz), 15.8.

^{19}F NMR (376 MHz, CDCl$_3$) δ -115.3, -116.0.

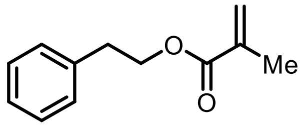

phenethyl methacrylate (1ca): Prepared according to general procedure E using phenylethyl alcohol. The spectra matched with reported literature.[1]

^{1}H NMR (400 MHz, CDCl$_3$) δ 7.35 – 7.18 (m, 5H), 6.07 (s, 1H), 5.53 (s, 1H), 4.34 (t, J = 7.2 Hz, 2H), 2.98 (t, J = 7.2 Hz, 2H), 1.91 (t, J = 1.2 Hz, 3H).

^{13}C NMR (100 MHz, CDCl$_3$) δ 167.2, 137.9, 136.3, 128.9, 128.4, 126.5, 125.39, 65.1, 35.1, 18.2.

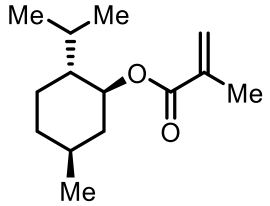

(1*S*,2*R*,5*S*)-2-isopropyl-5-methylcyclohexyl methacrylate (1cb): Prepared according to general procedure E using *L*-menthol. The spectra matched with reported literature.[12]
^{1}H NMR (400 MHz, CDCl$_3$) δ 6.08 (s, 1H), 5.52 (s, 1H), 4.74 (td, J = 10.8, 4.4 Hz, 1H), 2.04 (dd, J = 7.6, 3.6 Hz, 1H), 1.94 (s, 3H), 1.88 (ddd, J = 14.0, 7.2, 2.4 Hz, 1H), 1.69 (d, J = 12.4 Hz, 2H), 1.57 – 1.40 (m, 2H), 1.15 – 0.96 (m, 2H), 0.92 – 0.88 (m, 7H), 0.77 (d, J = 6.8 Hz, 3H).
^{13}C NMR (100 MHz, CDCl$_3$) δ 166.9, 136.9, 124.9, 74.4, 47.2, 40.9, 34.4, 31.5, 26.5, 23.7, 22.1, 20.8, 18.4, 16.5.

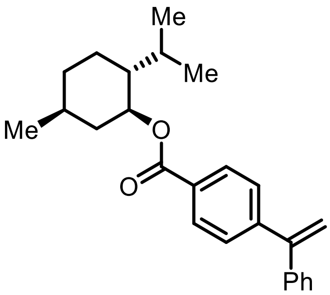

(1*S*,2*R*,5*S*)-2-isopropyl-5-methylcyclohexyl 4-(1-phenylvinyl)benzoate (1cd): Prepared according to general procedure F from 4-(1-phenylvinyl)benzoic acid and menthol.
^{1}H NMR (400 MHz, CDCl$_3$) δ 8.04 – 7.99 (m, 2H), 7.42 – 7.38 (m, 2H), 7.35 – 7.28 (m, 5H), 5.53 (d, J = 7.6 Hz, 2H), 4.95 (td, J = 10.8, 4.4 Hz, 1H), 2.19 – 2.09 (m, 1H), 1.97 (ddt, J = 14.0, 7.2, 3.6 Hz, 1H), 1.72 (dt, J = 15.2, 3.2 Hz, 2H), 1.60 – 1.52 (m, 2H), 1.17 – 1.06 (m, 2H), 0.92 (dd, J = 6.8, 4.0 Hz, 6H), 0.89 – 0.84 (m, 1H), 0.81 (d, J = 6.8 Hz, 3H).
^{13}C NMR (100 MHz, CDCl$_3$) δ 166.0, 149. 4, 146.0, 140.9, 130.1, 129.6, 128.4, 128.3, 128.1, 115.8, 74.9, 47.4, 41.1, 34.4, 31.5, 26.6, 23.7, 22.2, 20.9, 16.6.
HRMS (ESI-TOF): *m/z* Calcd. For $C_{25}H_{30}O_2$: $(M+Na)^+$ 385.2138 Found: 385.2129.

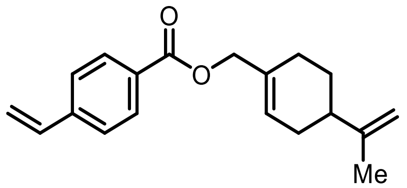

(4-(prop-1-en-2-yl)cyclohex-1-en-1-yl)methyl 4-vinylbenzoate (1ce): Prepared according to general procedure F using (4-(prop-1-en-2-yl)cyclohex-1-en-1-yl)methanol.
^{1}H NMR (400 MHz, CDCl$_3$) δ 7.87 (d, J = 8.0 Hz, 2H), 7.28 (d, J = 8.0 Hz, 2H), 6.57 (dd, J = 17.6, 10.8 Hz, 1H), 5.76 – 5.63 (m, 2H), 5.20 (d, J = 10.8 Hz, 1H), 4.61 – 4.54 (m, 4H), 2.01 (s, 4H), 1.89 – 1.78 (m, 1H), 1.71 (d, J = 12.0 Hz, 1H), 1.59 (s, 3H), 1.36 (ddd, J = 20.0, 12.0, 8.8 Hz, 1H).
^{13}C NMR (100 MHz, CDCl$_3$) δ 165.8, 149.2, 141.7, 135.9, 132.5, 129.8, 129.4, 125.9, 125.3, 116.2, 108.8, 68.6, 40.7, 30.3, 27.2, 26.3, 20.6.
HRMS (ESI-TOF): *m/z* Calcd. For $C_{19}H_{22}O_2$: $(M+Na)^+$ 305.1512. Found: 305.1514.

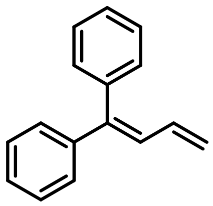

buta-1,3-diene-1,1-diyldibenzene (3a): Prepared according to general procedure G from benzophenone. The spectra matched with reported literature. [13]

^{1}H NMR (400 MHz, CDCl$_3$) δ 7.37 – 7.15 (m, 10H), 6.70 (dd, *J* = 11.2, 2.0 Hz, 1H), 6.52 – 6.38 (m, 1H), 5.36 (d, *J* = 16.8 Hz, 1H), 5.10 (d, *J* = 10.0 Hz, 1H).

^{13}C NMR (100 MHz, CDCl$_3$) δ 143.3, 142.2, 139.8, 135.1, 130.5, 128.6, 128.2(9), 128.2(7), 127.7, 127.6, 127.5, 118.7.

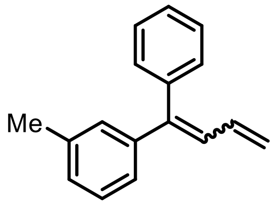

1-methyl-3-(1-phenylbut-1-en-1-yl)benzene (3b, mixture of isomers, *E/Z* = 1.1:1): Prepared according to general procedure G from phenyl(*o*-tolyl)methanone. The spectra matched with reported literature. [3]

^{1}H NMR (400 MHz, CDCl$_3$) δ 7.33 – 6.86 (m, 9H), 6.60 (d, *J* = 11.2 Hz, 1H), 6.43 – 6.27 (m, 1H), 5.30 (d, *J* = 1.2 Hz, 0.51H), 5.26 (d, *J* = 1.2 Hz, 0.48H), 5.03 (s, 0.51H), 5.00 (s, 0.48H), 2.25 (s, 1.40H), 2.20 (s, 1.58H).

^{13}C NMR (100 MHz, CDCl$_3$) δ 143.4(3), 143.4(0), 142.3, 142.2, 139.9, 139.7, 137.9, 137.8, 135.2, 135.1, 131.1, 130.5, 128.6, 128.5, 128.4, 128.3(3), 128.2(9), 128.2(4), 128.2(1), 128.1(5), 127.6(9), 127.6(5), 127.5(7), 127.5, 125.0, 118.6, 118.5, 21.5(9), 21.5(5).

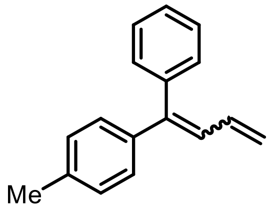

1-methyl-4-(1-phenylbuta-1,3-dien-1-yl)benzene (3c, mixture of isomers, *E/Z* = 1.1:1): Prepared according to general procedure A from phenyl(*p*-tolyl)methanone and allyltriphenylphosphonium bromide. The spectra matched with reported literature. [14]

^{1}H NMR (400 MHz, CDCl$_3$) δ 7.40 – 7.06 (m, 9H), 6.69 (dd, *J* = 11.2, 2.8 Hz, 1H), 6.53 – 6.37 (m, 1H), 5.37 (ddd, *J* = 16.8, 4.4, 2.0 Hz, 1H), 5.10 (ddd, *J* = 9.6, 6.8, 2.0 Hz, 1H), 2.39 (s, 1.44H), 2.33 (s, 1.55H).

^{13}C NMR (100 MHz, CDCl$_3$) δ 143.3, 143.2, 142.4, 139.9, 139.4, 137.5, 137.2, 136.8, 135.2(2), 135.1(6), 130.5(2), 130.4(5), 129.0(4), 128.9(7), 128.5, 128.3, 128.2, 127.9, 127.8, 127.6, 127.4, 118.4, 118.3, 21.4, 21.3.

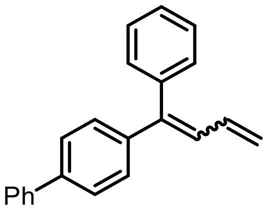

4-(1-phenylbuta-1,3-dien-1-yl)-1,1'-biphenyl (3d, mixture of isomers, *E/Z* = 1:1): Prepared according to general procedure A from 4-phenylbenzophenone and allyltriphenylphosphonium bromide. The spectra matched with reported literature.[14]

^{1}H NMR (400 MHz, CDCl$_3$) δ 7.68 – 7.21 (m, 14H), 6.76 (dd, J = 20.0, 11.2 Hz, 1H), 6.59 – 6.39 (m, 1H), 5.44 (t, J = 1.6 Hz, 0.50H), 5.39 (t, J = 1.6 Hz, 0.50H), 5.19 – 5.11 (m, 1H).

^{13}C NMR (100 MHz, CDCl$_3$) δ 142.9, 142.8, 142.3, 141.1, 140.9, 140.8, 140.4, 140.3, 139.7, 138.8, 135.1, 131.0, 130.6, 129.0, 128.9(2), 128.8(7), 128.6, 128.4, 128.1, 127.8, 127.7, 127.6, 127.5(0), 127.4(6), 127.2, 127.1, 127.0(2), 126.9(6), 118.9, 118.8.

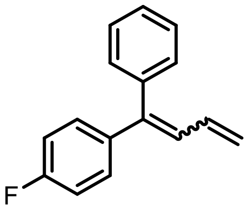

1-fluoro-4-(1-phenylbuta-1,3-dien-1-yl)benzene (3e, mixture of isomers, *E/Z* = 1.2:1): Prepared according to general procedure G from (4-fluorophenyl)(phenyl)methanone. The spectra matched with reported literature.[15]

^{1}H NMR (400 MHz, CDCl$_3$) δ 7.24 – 7.00 (m, 7H), 6.89 (td, J = 8.8, 2.0 Hz, 1H), 6.78 (td, J = 8.8, 2.0 Hz, 1H), 6.56 (d, J = 11.2 Hz, 0.45H), 6.49 (d, J = 11.2 Hz, 0.55H), 6.35 – 6.20 (m, 1H), 5.25 (d, J = 6.8 Hz, 0.53H), 5.21 (d, J = 6.8 Hz, 0.48H), 4.98 (t, J = 9.6 Hz, 1H).

^{13}C NMR (100 MHz, CDCl$_3$) δ 161.3 (d, J_{FC} = 246.1 Hz), 161.1 (d, J_{FC} = 245.4 Hz), 141.0(5), 141.0(0), 140.9, 138.5, 137.2 (d, J_{FC} = 3.0 Hz), 134.5 (d, J_{FC} = 3.1 Hz), 133.8, 133.6, 131.0 (d, J_{FC} = 7.8 Hz), 129.3, 128.2 (d, J_{FC} = 7.8 Hz), 127.8, 127.4, 127.2, 126.6, 126.5, 118.0, 117.7, 114.1 (d, J_{FC} = 21.1 Hz), 114.0 (d, J_{FC} = 21.3 Hz).

^{19}F NMR (376 MHz, CDCl$_3$) δ -115.4, -115.5.

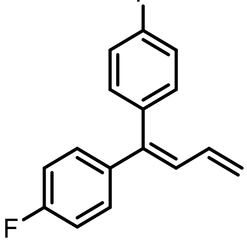

4,4'-(buta-1,3-diene-1,1-diyl)bis(fluorobenzene) (3f): Prepared according to general

procedure G from bis(4-fluorophenyl)methanone. The spectra matched with reported literature.[14]

^{1}H NMR (400 MHz, CDCl$_3$) δ 6.19 – 6.06 (m, 4H), 5.99 (t, J = 8.8 Hz, 2H), 5.88 (t, J = 8.8 Hz, 2H), 5.59 (d, J = 11.2 Hz, 1H), 5.36 (dt, J = 16.8, 10.4 Hz, 1H), 4.34 (d, J = 16.8 Hz, 1H), 4.09 (d, J = 10.4 Hz, 1H).

^{13}C NMR (100 MHz, CDCl$_3$) δ 162.5 (d, J_{FC} = 246.4 Hz), 162.3 (d, J_{FC} = 245.7 Hz), 141.1, 138.2 (d, J_{FC} = 3.1 Hz), 135.4 (d, J_{FC} = 3.1 Hz), 134.6, 132.1 (d, J_{FC} = 7.8 Hz), 129.2 (d, J_{FC} = 7.8 Hz), 128.8, 119.2, 115.4 (d, J_{FC} = 21.2 Hz), 115.2 (d, J_{FC} = 21.3 Hz).

^{19}F NMR (376 MHz, CDCl$_3$) δ -114.9, -115.1.

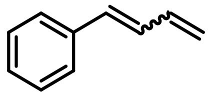

buta-1,3-dien-1-ylbenzene (3g, mixture of isomers, *E/Z* = 2:1): Prepared according to general procedure A from benzaldehyde and allyltriphenylphosphonium bromide. The spectra matched with reported literature.[16]

^{1}H NMR (400 MHz, CDCl$_3$) δ 7.40 – 7.15 (m, 5H), 6.95 – 6.82 (m, 0.64H), 6.75 (ddd, J = 12.4, 10.4, 5.2 Hz, 0.36H), 6.55 – 6.40 (m, 1.37H), 6.28 – 6.19 (m, 0.65H), 5.40 – 5.24 (m, 1H), 5.23 – 5.10 (m, 1H).

^{13}C NMR (100 MHz, CDCl$_3$) δ 137.4, 137.3, 137.2, 133.3, 133.0, 130.9, 130.5, 129.7, 129.1, 128.7, 128.3, 127.7, 127.1, 126.5, 119.7, 117.7.

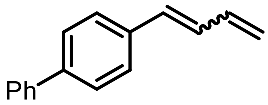

4-(buta-1,3-dien-1-yl)-1,1'-biphenyl (3h, mixture of isomers, *E/Z* = 1.1:1): Prepared according to general procedure A from *p*-diphenylaldehyde and allyltriphenylphosphonium bromide. The spectra matched with reported literature.[17]

^{1}H NMR (400 MHz, CDCl$_3$) δ 7.60 – 7.51 (m, 4H), 7.46 – 7.37 (m, 4H), 7.34 – 7.28 (m, 1H), 7.01 – 6.75 (m, 1H), 6.62 – 6.19 (m, 2H), 5.43 – 5.29 (m, 1H), 5.23 (d, J = 10.4 Hz, 0.52H), 5.17 (d, J = 10.2 Hz, 0.47H).

^{13}C NMR (100 MHz, CDCl$_3$) δ 140.8, 140.7, 140.4, 139.9, 137.3, 136.5, 136.3, 133.4, 132.5, 131.0, 130.1, 129.8, 129.6, 128.9, 127.4(4), 127.3(8), 127.1, 127.0(2), 127.0(0), 119.9, 117.8.

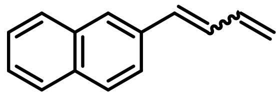

2-(buta-1,3-dien-1-yl)naphthalene (3i, mixture of isomers): Prepared according to general procedure A from 2-naphthaldehyde and allyltriphenylphosphonium bromide. The spectra matched with reported literature.[18]

^{1}H NMR (400 MHz, CDCl$_3$) δ 7.84 – 7.73 (m, 4H), 7.50 – 7.42 (m, 3H), 7.04 – 6.80 (m, 1H), 6.76 – 6.52 (m, 1H), 6.36 (t, J = 11.2 Hz, 1H), 5.50 – 5.34 (m, 1H), 5.25 (dd, J = 23.2, 10.8 Hz, 1H).

^{13}C NMR (100 MHz, CDCl$_3$) δ 137.4, 135.0, 134.8, 133.8, 133.4, 133.1, 132.5, 131.3, 130.6, 130.1, 128.4, 128.1, 128.0, 127.9, 127.8(1), 127.7(5), 127.3, 126.7, 126.4, 126.3, 126.1, 126.0(4), 123.5(9), 120.1, 118.0.

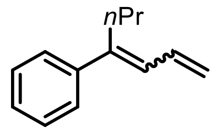

hepta-1,3-dien-4-ylbenzene (3j, mixture of isomers): Prepared according to general procedure G from propyl(phenyl)methanone. **Note**: This compound contains impurities.
^{1}H NMR (400 MHz, CDCl$_3$) δ 7.40 – 7.17 (m, 4H), 6.82 – 5.76 (m, 2H), 5.34 – 4.87 (m, 2H), 3.26 – 2.13 (m, 2H), 1.41 (tq, J = 14.8, 7.6 Hz, 2H), 1.08 – 0.87 (m, 3H).
^{13}C NMR (100 MHz, CDCl$_3$) δ 142.5, 142.3, 136.2, 134.7, 133.6, 132.1, 128.7, 128.4, 128.3, 127.2, 126.6, 126.4, 126.2, 117.7, 116.1, 115.3, 34.4, 32.0, 22.4, 22.1, 14.3, 14.1.

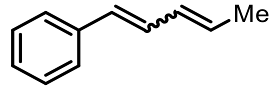

penta-1,3-dien-1-ylbenzene (3k, mixture of isomers, *E/Z* = 3.3:1): Prepared according to general procedure A from *trans*-3-phenylacrylaldehyde and ethyltriphenylphosphonium bromide. The spectra matched with reported literature.[16]
^{1}H NMR (400 MHz, CDCl$_3$) δ 7.37 (dd, J = 20.8, 7.6 Hz, 2H), 7.27 (q, J = 8.0 Hz, 2H), 7.17 (q, J = 8.0, 7.6 Hz, 1H), 7.08 (dd, J = 15.6, 11.2 Hz, 0.23H), 6.73 (dd, J = 15.6, 10.4 Hz, 0.76H), 6.50 (d, J = 15.6 Hz, 0.23H), 6.40 (d, J = 15.6 Hz, 0.74H), 6.25 – 6.11 (m, 1H), 5.79 (dq, J = 13.6, 6.8 Hz, 0.70H), 5.57 (dq, J = 14.4, 7.2 Hz, 0.19H), 1.84 (d, J = 7.2 Hz, 0.73H), 1.79 (d, J = 6.8 Hz, 2.26H).
^{13}C NMR (100 MHz, CDCl$_3$) δ 137.8, 132.0, 130.4, 129.9, 129.8, 129.4, 128.7, 128.6, 127.4, 127.2(2), 127.1(5), 126.4, 126.2, 124.2, 18.5, 13.7.

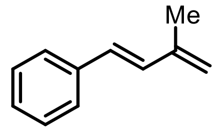

(3-methylbuta-1,3-dien-1-yl)benzene (3l): Prepared according to general procedure A from *trans*-benzylideneacetone and methyltriphenylphosphonium bromide. The spectra matched with reported literature.[13]
^{1}H NMR (400 MHz, CDCl$_3$) δ 7.46 – 7.40 (m, 2H), 7.32 (t, J = 7.6 Hz, 2H), 7.25 – 7.20 (m, 1H), 6.88 (d, J = 16.0 Hz, 1H), 6.53 (d, J = 16.0 Hz, 1H), 5.09 (d, J = 16.8 Hz, 2H), 1.98 (s, 3H).
^{13}C NMR (100 MHz, CDCl$_3$) δ 143.7, 136.7, 134.5, 130.7, 129.1, 128.4, 127.3, 126.7, 20.9.

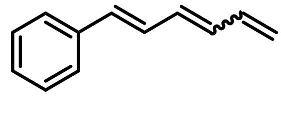

((1*E*,3*E*)-hexa-1,3,5-trien-1-yl)benzene (3m, mixture of isomers, *E/Z* = 1:1): Prepared according to general procedure A from *trans*-3-phenylacrylaldehyde and allyltriphenylphosphonium bromide. The spectra matched with reported literature.[16]
^{1}H NMR (400 MHz, CDCl$_3$) δ 7.42-7.33 (m, 2H), 7.32-7.25 (m, 2H), 7.24 – 7.14 (m, 1H), 6.83 – 6.73 (m, 1H), 6.56 (d, J = 7.2 Hz, 0.5H), 6.52 (d, J = 7.2 Hz, 0.5H), 6.46 – 6.27 (m, 2H), 6.15 (t, J = 10.8 Hz, 0.5H), 6.05 (t, J = 10.8 Hz, 0.5H), 5.35 – 5.06 (m, 2H).
^{13}C NMR (100 MHz, CDCl$_3$) δ 137.4, 137.2, 133.9, 133.7, 133.6, 133.1, 132.3, 130.5, 130.2, 128.9(1), 128.8(8), 128.8, 127.8, 127.7, 126.6, 126.5, 124.1, 118.5, 117.6.

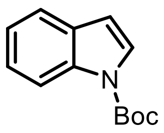

***tert*-butyl 1*H*-indole-1-carboxylate (6a):** Prepared according to general procedure H using 1*H*-indole. The spectra matched with reported literature.[1]
^{1}H NMR (400 MHz, CDCl$_3$) δ 8.14 (d, J = 8.4 Hz, 1H), 7.62 – 7.50 (m, 2H), 7.27 – 7.34 (m, 1H), 7.18 – 7.26 (m, 1H), 6.56 (d, J = 3.6 Hz, 1H), 1.67 (s, 9H).
^{13}C NMR (100 MHz, CDCl$_3$) δ 149.9, 135.3, 130.7, 126.0, 124.3, 122.7, 121.0, 115.2, 107.4, 83.7, 28.3.

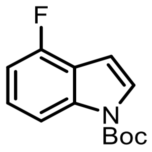

***tert*-butyl 4-fluoro-1*H*-indole-1-carboxylate (6b):** Prepared according to general procedure H using 4-fluoro-1*H*-indole. The spectra matched with reported literature.[19]
^{1}H NMR (400 MHz, CDCl$_3$) δ 7.93 (d, J = 8.4 Hz, 1H), 7.56 (d, J = 3.6 Hz, 1H), 7.23 (td, J = 8.4, 5.2 Hz, 1H), 6.91 (ddd, J = 9.6, 8.0, 0.8 Hz, 1H), 6.66 (dd, J = 3.6, 0.8 Hz, 1H), 1.67 (s, 9H).
^{13}C NMR (100 MHz, CDCl$_3$) δ 155.9 (d, J_{FC} = 246.1 Hz), 149.7, 137.5 (d, J_{FC} = 9.2 Hz), 126.0, 125.0 (d, J_{FC} =7.5 Hz), 119.5 (d, J_{FC} =22.0 Hz), 111.4 (d, J_{FC} =3.6 Hz), 108.0 (d, J_{FC} =18.4 Hz), 102.9, 84.3, 28.3.
^{19}F NMR (376 MHz, CDCl$_3$) δ -123.2.

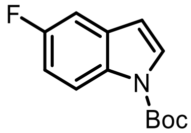

***tert*-butyl 5-fluoro-1*H*-indole-1-carboxylate (6c):** Prepared according to general procedure H using 5-fluoro-1*H*-indole. The spectra matched with reported literature.[1]
^{1}H NMR (400 MHz, CDCl$_3$) δ 8.08 (s, 1H), 7.62 (d, J = 3.6 Hz, 1H), 7.17 – 7.23 (m, 1H), 6.98 – 7.07 (m, 1H), 6.52 (d, J = 3.6 Hz, 1H), 1.67 (s, 9H).
^{13}C NMR (100 MHz, CDCl$_3$) δ 159.3(d, J_{FC} = 237.4 Hz), 149.6, 131.7, 131.5(d, J_{FC} =

9.8 Hz), 127.5, 116.1(d, J_{FC} = 9.1 Hz), 112.0(d, J_{FC} = 24.9 Hz), 107.1(d, J_{FC} = 3.9 Hz), 106.4(d, J_{FC} = 23.6 Hz), 84.0, 28.2.
^{19}F NMR (376 MHz, $CDCl_3$) δ -121.1.

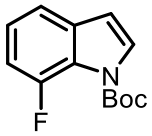

***tert*-butyl 7-fluoro-1*H*-indole-1-carboxylate (6d):** Prepared according to general procedure H using 7-fluoro-1*H*-indole. The spectra matched with reported literature.[20]
^{1}H NMR (400 MHz, $CDCl_3$) δ 7.62 (d, *J* = 3.6 Hz, 1H), 7.30 (dd, *J* = 7.6, 1.2 Hz, 1H), 7.13 (td, *J* = 8.0, 4.0 Hz, 1H), 7.04 – 6.97 (m, 1H), 6.56 (dd, *J* = 3.6, 2.0 Hz, 1H), 1.64 (s, 9H).
^{13}C NMR (100 MHz, $CDCl_3$) δ 150.1 (d, J_{FC} = 251.2 Hz), 149.0, 135.0 (d, J_{FC} = 3.6 Hz), 128.3, 123.6 (d, J_{FC} = 7.1 Hz), 122.0 (d, J_{FC} = 10.0 Hz), 116.8 (d, J_{FC} = 3.6 Hz), 111.6 (d, J_{FC} = 23.1 Hz), 107.4, 84.2, 28.0.
^{19}F NMR (376 MHz, $CDCl_3$) δ -117.0.

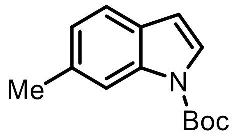

tert-butyl 6-methyl-1*H*-indole-1-carboxylate (6e): Prepared according to general procedure H using methyl 6-methyl-1*H*-indole. The spectra matched with reported literature.[21]
^{1}H NMR (400 MHz, $CDCl_3$) δ 8.02 (s, 1H), 7.49 (d, *J* = 3.6 Hz, 1H), 7.42 (d, *J* = 8.0 Hz, 1H), 7.08 – 6.99 (m, 1H), 6.49 (d, *J* = 3.6 Hz, 1H), 2.48 (s, 3H), 1.65 (s, 9H).
^{13}C NMR (100 MHz, $CDCl_3$) δ149.9, 135.7, 134.2, 128.3, 125.3, 124.3, 120.5, 115.5, 107.3, 83.5, 28.2, 22.0.

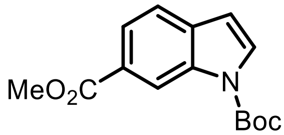

1-(*tert*-butyl) 6-methyl 1*H*-indole-1,6-dicarboxylate (6f): Prepared according to general procedure H using methyl 1*H*-indole-6-carboxylate. The spectra matched with reported literature.[1]
^{1}H NMR (400 MHz, $CDCl_3$) δ 8.87 (s, 1H), 7.89 – 7.94 (m, 1H), 7.70 – 7.75 (m 1H), 7.63 – 7.56 (m, 1H), 6.61 (d, *J* = 3.6 Hz, 1H), 3.94 (s, 3H), 1.70 (s, 9H).
^{13}C NMR (100 MHz, $CDCl_3$) δ 167.8, 149.5, 134.7, 134.4, 128.9, 126.0, 124.0, 120.7, 117.2, 107.3, 84.5, 52.2, 28.2.

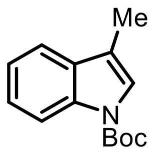

***tert*-butyl 3-methyl-1*H*-indole-1-carboxylate (6g):** Prepared according to general procedure H using 3-methyl-1*H*-indole. The spectra matched with reported literature.[22]
^{1}H NMR (400 MHz, CDCl$_3$) δ 8.12 (s, 1H), 7.50 – 7.45 (m, 1H), 7.39 – 7.27 (m, 2H), 7.23 (td, J = 7.6, 1.2 Hz, 1H), 2.25 (d, J = 1.2 Hz, 3H), 1.65 (s, 9H).
^{13}C NMR (100 MHz, CDCl$_3$) δ 149.9, 135.5, 131.5, 124.3, 122.8, 122.4, 119.0, 116.4, 115.2, 83.2, 28.3, 9.7.

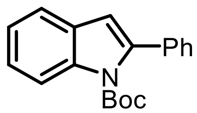

***tert*-butyl 2-phenyl-1*H*-indole-1-carboxylate (6h):** Prepared according to general procedure H from 2-phenyl-1*H*-indole. The spectra matched with reported literature.[19]
^{1}H NMR (400 MHz, CDCl$_3$) δ 8.22 (d, J = 8.4 Hz, 1H), 7.58 – 7.52 (m, 1H), 7.44 – 7.30 (m, 6H), 7.28 – 7.20 (m, 1H), 6.57 – 6.53 (m, 1H), 1.30 (s, 9H).
^{13}C NMR (100 MHz, CDCl$_3$) δ 150.3, 140.6, 137.6, 135.1, 129.3, 128.8, 127.9, 127.7, 124.4, 123.0, 120.6, 115.3, 110.0, 83.5, 27.7.

4. Dicarboxylic acid derivatives

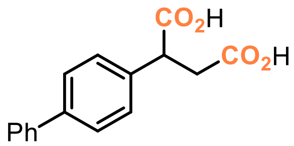

2-([1,1'-biphenyl]-4-yl)succinic acid (2a): Prepared according to general procedure from 4-vinylbiphenyl (0.2 mmol, 36.1 mg, 1.0 equiv). After 12 hours, the product was purified by flash column chromatography (EtOAc/PE 2/1 as the eluent) and obtained as white solid (39.3 mg, 73% yield).
^{1}H NMR (400 MHz, CD_3OD) δ 7.62 – 7.55 (m, 4H), 7.45 – 7.37 (m, 4H), 7.32 (t, *J* = 7.6 Hz, 1H), 4.07 (dd, *J* = 10.0, 5.2 Hz, 1H), 3.14 (dd, *J* = 17.2, 10.0 Hz, 1H), 2.67 (dd, *J* = 17.2, 5.2 Hz, 1H).
^{13}C NMR (100 MHz, CD_3OD) δ 176.5, 175.2, 141.9, 141.7, 138.9, 129.9, 129.4, 128.4, 128.3, 127.9, 48.3, 38.7.
HRMS (ESI-TOF): *m/z* Calcd. For $C_{16}H_{14}O_4$: $(M+Na)^+$ 293.0784 Found: 293.0779.

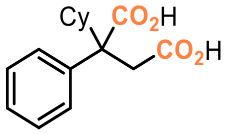

2-cyclohexyl-2-phenylsuccinic acid (2b): Prepared according to general procedure using (1-cyclohexylvinyl)benzene (0.2 mmol, 37.3 mg, 1.0 equiv). After 72 hours under CO_2 atmosphere, the product was purified by flash column chromatography (EtOAc/PE 2/1 as the eluent) and obtained as white solid (20.0 mg, 36% yield).
^{1}H NMR (400 MHz, CD_3OD) δ 7.38 (d, *J* = 8.0 Hz, 2H), 7.31 – 7.24 (m, 2H), 7.23 – 7.17 (m, 1H), 3.21 (d, *J* = 16.8 Hz, 1H), 3.06 (d, *J* = 16.8 Hz, 1H), 2.13 (t, *J* = 11.6 Hz, 1H), 1.68 (dt, *J* = 38.8, 13.2 Hz, 5H), 1.23 (dt, *J* = 26.0, 10.8 Hz, 2H), 1.08 – 0.77 (m, 3H).
^{13}C NMR (100 MHz, CD_3OD) δ 177.9, 175.6, 142.0, 128.8, 128.7, 127.5, 57.3, 47.1, 40.1, 30.0, 29.9, 28.2, 28.1, 27.6.
HRMS (ESI-TOF): *m/z* Calcd. For $C_{16}H_{20}O_4$: $(M+Na)^+$ 299.1254. Found: 299.1244.

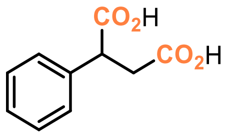

2-phenylsuccinic acid (2c): Prepared according to general procedure (3DPAFIPN (0.005 mmol, 3.2 mg, 2.5 mol%) and CsF (1.6 mmol, 243.0 mg, 8.0 equiv)) using styrene (0.2 mmol, 23.0 μL, 1.0 equiv). After 8 hours, the product was purified by flash column chromatography (EtOAc/PE 2/1 as the eluent) and obtained as white solid (24.0 mg, 62% yield).
^{1}H NMR (400 MHz, CD_3OD) δ 7.33 – 7.23 (m, 5H), 4.01 (dd, *J* = 10.0, 5.2 Hz, 1H), 3.10 (dd, *J* = 17.2, 10.0 Hz, 1H), 2.62 (dd, *J* = 17.2, 5.2 Hz, 1H).

^{13}C NMR (100 MHz, CD_3OD) δ 176.7, 175.2, 139.9, 129.8, 128.9, 128.5, 48.7, 38.8.
HRMS (ESI-TOF): *m/z* Calcd. For $C_{10}H_{10}O_4$: $(M+Na)^+$ 217.0471. Found: 217.0468.

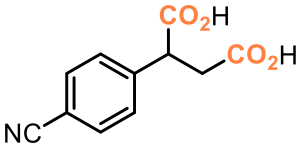

2-(4-cyanophenyl)succinic acid (2d): Prepared according to general procedure (3DPAFIPN (0.005 mmol, 3.2 mg, 2.5 mol%) and CsF (1.0 mmol, 151.9 mg, 5.0 equiv)) using 4-vinylbenzonitrile (0.2 mmol, 24.0 µL, 1.0 equiv). After 12 hours under CO_2 atmosphere, the product was purified by flash column chromatography (EtOAc/PE 2/1 as the eluent) and obtained as white solid (41.0 mg, 94% yield).
^{1}H NMR (400 MHz, CD_3OD) δ 7.70 (dd, J = 8.0, 1.6 Hz, 2H), 7.53 (dd, J = 8.0, 1.6 Hz, 2H), 4.14 (dd, J = 9.6, 5.6 Hz, 1H), 3.13 (ddd, J = 17.2, 9.6, 1.6 Hz, 1H), 2.70 (ddd, J = 17.2, 5.6, 1.6 Hz, 1H).
^{13}C NMR (100 MHz, CD_3OD) δ 175.3, 174.6, 145.5, 133.6, 130.2, 119.5, 112.3, 48.7, 38.1.
HRMS (ESI-TOF): *m/z* Calcd. For $C_{11}H_9NO_4$: $(M+Na)^+$ 242.0424. Found: 242.0420.

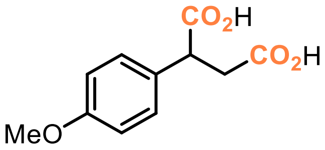

2-(4-methoxyphenyl)succinic acid (2e): Prepared according to general procedure (3DPAFIPN (0.005 mmol, 3.2 mg, 2.5 mol%) and CsF (1.0 mmol, 151.9 mg, 5.0 equiv)) using (0.2 mmol, 26.6 µL, 1.0 equiv). After 12 hours under CO_2 atmosphere, the product was purified by flash column chromatography (EtOAc/PE 2/1 as the eluent) and obtained as white solid (25.6 mg, 57% yield).
^{1}H NMR (400 MHz, CD_3OD) δ 7.23 – 7.20 (m, 2H), 6.89 – 6.84 (m, 2H), 3.94 (dd, J = 10.0, 5.2 Hz, 1H), 3.75 (s, 3H), 3.06 (dd, J = 17.2, 10.0 Hz, 1H), 2.58 (dd, J = 17.2, 5.2 Hz, 1H).
^{13}C NMR (100 MHz, CD_3OD) δ 176.9, 175.2, 160.5, 131.7, 129.9, 115.1, 55.7, 47.8, 38.8.
HRMS (ESI-TOF): *m/z* Calcd. For $C_{11}H_{12}O_5$: $(M+Na)^+$ 247.0577. Found: 247.0572.

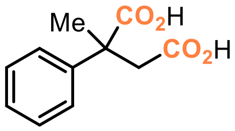

2-methyl-2-phenylsuccinic acid (2f): Prepared according to general procedure (3DPAFIPN (0.005 mmol, 3.2 mg, 2.5 mol%) and CsF (1.0 mmol, 151.9 mg, 5.0 equiv)) using prop-1-en-2-ylbenzene (0.2 mmol, 26.0 µL, 1.0 equiv). After 8 hours, the product was purified by flash column chromatography (EtOAc/PE 2/1 as the eluent) and obtained as white solid (21.7 mg, 52% yield).

^{1}H NMR (400 MHz, CD_3OD) δ 7.46 – 7.36 (m, 2H), 7.36 – 7.27 (m, 2H), 7.27 – 7.19 (m, 1H), 3.21 (d, J = 16.8 Hz, 1H), 2.81 (d, J = 16.8 Hz, 1H), 1.68 (s, 3H).
^{13}C NMR (100 MHz, CD_3OD) δ 178.8, 174.7, 144.7, 129.5, 128.0, 126.8, 49.2, 44.3, 24.0.
HRMS (ESI-TOF): *m/z* Calcd. For $C_{11}H_{12}O_4$: $(M+Na)^+$ 231.0628. Found: 231.0625.

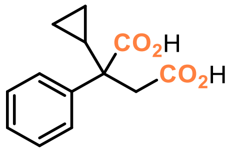

2-cyclopropyl-2-phenylsuccinic acid (2g): Prepared according to general procedure (3DPAFIPN (0.005 mmol, 3.2 mg, 2.5 mol%) and CsF (1.0 mmol, 151.9 mg, 5.0 equiv)) using (1-cyclopropylvinyl)benzene (0.2 mmol, 28.8 mg, 1.0 equiv). After 8 hours, the product was purified by flash column chromatography (EtOAc/PE 2/1 as the eluent) and obtained as white solid (27.9mg, 60% yield).
^{1}H NMR (400 MHz, CD_3OD) δ 7.34 – 7.26 (m, 4H), 7.23 (dt, J = 6.8, 3.2 Hz, 1H), 3.11 (s, 2H), 1.72 (ddd, J = 14.4, 8.4, 6.0 Hz, 1H), 0.50 (dt, J = 13.2, 6.8 Hz, 1H), 0.43 – 0.30 (m, 1H), 0.05 (p, J = 5.2 Hz, 2H).
^{13}C NMR (100 MHz, CD_3OD) δ 178.4, 174.9, 140.7, 128.9, 128.7, 128.1, 53.6, 42.9, 18.3, 3.7, 1.1.
HRMS (ESI-TOF): *m/z* Calcd. For $C_{13}H_{16}O_4$: $(M+Na)^+$ 259.0941. Found: 259.0931.

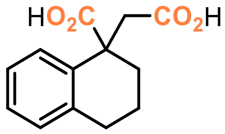

1-(carboxymethyl)-1,2,3,4-tetrahydronaphthalene-1-carboxylic acid (2h): Prepared according to general procedure (3DPAFIPN (0.005 mmol, 3.2 mg, 2.5 mol%) and CsF (1.0 mmol, 151.9 mg, 5.0 equiv)) using 1-methylene-1,2,3,4-tetrahydronaphthalene (0.2 mmol, 28.8 mg, 1.0 equiv). After 8 hours, the product was purified by flash column chromatography (EtOAc/PE 2/1 as the eluent) and obtained as white solid (18.1 mg, 39% yield).
^{1}H NMR (400 MHz, CD_3OD) δ 7.37 – 7.27 (m, 1H), 7.00 (ddd, J = 10.8, 6.4, 3.6 Hz, 3H), 3.04 (d, J = 16.8 Hz, 1H), 2.69 (t, J = 6.4 Hz, 2H), 2.61 (d, J = 16.8 Hz, 1H), 2.37 (ddd, J = 12.4, 9.2, 3.2 Hz, 1H), 2.08 (ddd, J = 13.6, 8.8, 3.2 Hz, 1H), 1.88 (ddq, J = 12.8, 6.4, 3.2 Hz, 1H), 1.69 (dtq, J = 12.8, 6.4, 3.2 Hz, 1H).
^{13}C NMR (100 MHz, CD_3OD) δ 179.0, 174.7, 138.7, 138.2, 130.6, 128.6, 127.9, 127.1, 45.2, 32.3, 30.9, 20.8.
HRMS (ESI-TOF): *m/z* Calcd. For $C_{13}H_{14}O_4$: $(M+Na)^+$ 257.0784. Found: 257.0784.

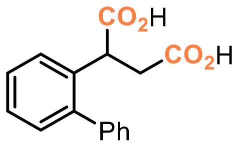

2-([1,1'-biphenyl]-2-yl)succinic acid (2i): Prepared according to general procedure from 2-vinyl-1,1'-biphenyl (0.2 mmol, 36.1 mg, 1.0 equiv). After 24 hours, the product was purified by flash column chromatography (EtOAc/PE 2/1 as the eluent) and obtained as white solid (51.2 mg, 95% yield).
^{1}H NMR (400 MHz, CD$_3$OD) δ 7.47 – 7.26 (m, 8H), 7.25 – 7.19 (m, 1H), 4.27 – 4.18 (dd, J = 8.0, 4.8 Hz, 1H), 3.05 – 2.93 (dd, J = 17.2, 10.8 Hz, 1H), 2.44 (dd, J = 17.2, 4.0 Hz, 1H),
^{13}C NMR (100 MHz, CD$_3$OD) δ 176.9, 175.0, 143.7, 142.4, 137.6, 131.6, 130.6, 129.3, 128.9, 128.3, 128.2, 127.9, 44.2, 39.0.
HRMS (ESI-TOF): m/z Calcd. For $C_{16}H_{14}O_4$: $(M+Na)^+$ 293.0784 Found: 293.0772.

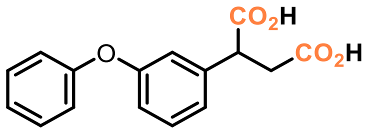

2-(3-phenoxyphenyl)succinic acid (2j): Prepared according to general procedure from 1-phenoxy-3-vinylbenzene (0.2 mmol, 39.3 mg, 1.0 equiv). After 24 hours, the product was purified by flash column chromatography (EtOAc/PE 2/1 as the eluent) and obtained as white solid (35.5 mg, 62% yield).
^{1}H NMR (400 MHz, CD$_3$OD) δ 7.36 – 7.27 (m, 3H), 7.13 – 7.03 (m, 2H), 7.01 – 6.95 (m, 3H), 6.89 – 6.83 (m, 1H), 3.99 (dd, J = 10.0, 5.6 Hz, 1H), 3.05 (dd, J = 16.8, 10.0 Hz, 1H), 2.62 (dd, J = 16.8, 5.6 Hz, 1H).
^{13}C NMR (100 MHz, DMSO-d_6) δ 173.8, 172.6, 156.8, 156.3, 140.9, 130.3, 130.2, 123.7, 122.8, 118.8, 117.9, 117.1, 46.7, 37.4.
HRMS (ESI-TOF): m/z Calcd. For $C_{16}H_{14}O_5$: $(M+Na)^+$ 309.0733 Found: 309.0720.

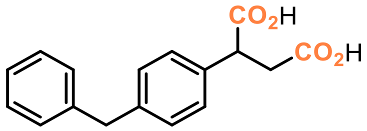

2-(4-benzylphenyl)succinic acid (2k): Prepared according to general procedure from 1-benzyl-4-vinylbenzene (0.2 mmol, 38.9 mg, 1.0 equiv). After 24 hours, the product was purified by flash column chromatography (EtOAc/PE 2/1 as the eluent) and obtained as white solid (19.3 mg, 34% yield).
^{1}H NMR (400 MHz, DMSO-d_6) δ 12.25 (brs, 2H), 7.30 – 7.13 (m, 9H), 3.94 – 3.83 (m, 3H), 2.92 (dd, J = 16.8, 10.4 Hz, 1H), 2.58 – 2.49 (m, 1H).
^{13}C NMR (100 MHz, CD$_3$OD) δ 174.1, 172.7, 141.2, 140.3, 136.3, 128.9, 128.7, 128.5, 127.8, 126.0, 46.5, 40.7, 37.4.
HRMS (ESI-TOF): m/z Calcd. For $C_{17}H_{16}O_4$: $(M+Na)^+$ 307.0941 Found: 307.0938.

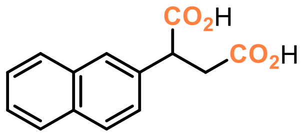

2-([1,1'-biphenyl]-2-yl)succinic acid (2l): Prepared according to general procedure from 2-vinylnaphthalene (0.2 mmol, 30.2 mg, 1.0 equiv). After 24 hours, the product was purified by flash column chromatography (EtOAc/PE 2/1 as the eluent) and obtained as white solid (33.2 mg, 68% yield).

^{1}H NMR (400 MHz, CD_3OD) δ 7.88 – 7.74 (m, 4H), 7.52 – 7.37 (m, 3H), 4.21 (dd, J = 9.6, 5.2 Hz, 1H), 3.22 (dd, J = 17.2, 10.0 Hz, 1H), 2.74 (dd, J = 17.2, 5.2 Hz, 1H).

^{13}C NMR (100 MHz, CD_3OD) δ 176.6, 175.2, 137.2, 135.0, 134.2, 129.5, 128.8, 128.6, 127.7, 127.3, 127.0, 126.8, 38.7.

HRMS (ESI-TOF): *m/z* Calcd. For $C_{14}H_{12}O_4$: $(M+Na)^+$ 267.0628 Found: 267.0621.

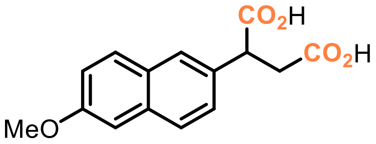

2-(6-methoxynaphthalen-2-yl)succinic acid (2m): Prepared according to general procedure from 2-methoxy-6-vinylnaphthalene (0.2 mmol, 36.8 mg, 1.0 equiv). After 24 hours, the product was purified by flash column chromatography (EtOAc/PE 2/1 as the eluent) and obtained as white solid (33.7 mg, 61% yield).

^{1}H NMR (400 MHz, CD_3OD) δ 7.76 – 7.69 (m, 3H), 7.40 (dd, J = 8.4, 2.0 Hz, 1H), 7.12 (d, J = 2.8 Hz, 1H), 7.12 (dd, J = 9.2, 2.8 Hz, 1H), 4.14 (dd, J = 10.0, 5.2 Hz, 1H), 3.89 (s, 3H), 3.19 (dd, J = 17.2, 10.4 Hz, 1H), 2.70 (dd, J = 17.2, 5.6 Hz, 1H).

^{13}C NMR (100 MHz, CD_3OD) δ176.8, 175.3, 159.3, 135.4, 134.8, 130.4, 130.2, 128.4, 127.5, 127.2, 120.1, 106.5, 55.7, 38.8.

HRMS (ESI-TOF): *m/z* Calcd. For $C_{15}H_{14}O_5$: $(M-H)^-$ 273.0768. Found: 273.0775.

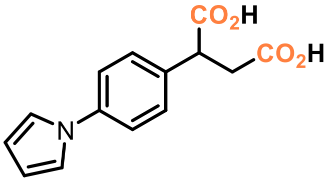

2-(4-(1*H*-pyrrol-1-yl)phenyl)succinic acid (2n): Prepared according to general procedure from 1-(4-vinylphenyl)-1*H*-pyrrole (0.2 mmol, 33.8 mg, 1.0 equiv). After 24 hours, the product was purified by flash column chromatography (EtOAc/PE 2/1 as the eluent) and obtained as white solid (35.2 mg, 68% yield).

^{1}H NMR (400 MHz, CD_3OD) δ 7.47 – 7.35 (m, 4H), 7.19 – 7.13 (m, 2H), 6.28 – 6.24 (m, 2H), 4.05 (dd, J = 9.6, 5.2 Hz, 1H), 3.12 (dd, J = 16.8, 10.0 Hz, 1H), 2.66 (dd, J = 16.8, 5.6 Hz, 1H).

^{13}C NMR (100 MHz, CD_3OD) δ 176.5, 175.1, 141.4, 137.0, 130.2, 121.2, 120.0, 111.4, 48.1, 38.6.

HRMS (ESI-TOF): *m/z* Calcd. For $C_{14}H_{13}NO_4$: $(M+Na)^+$ 282.0737. Found: 282.0724.

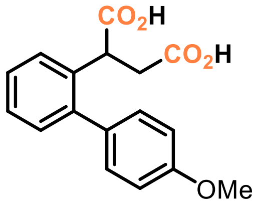

2-(4'-methoxy-[1,1'-biphenyl]-2-yl)succinic acid (2o): Prepared according to general procedure from 4'-methoxy-2-vinyl-1,1'-biphenyl (0.2 mmol, 42.1 mg, 1.0 equiv). After 24 hours, the product was purified by flash column chromatography (EtOAc/PE 2/1 as the eluent) and obtained as white solid (45.1 mg, 75% yield).

^{1}H NMR (400 MHz, CD_3OD) δ 7.39 – 7.23 (m, 5H), 7.24 – 7.18 (m, 1H), 7.02 – 6.95 (m, 2H), 4.27 (dd, J = 10.8, 2.4 Hz, 1H), 3.83 (s, 3H), 2.98 (dd, J = 16.8, 10.4 Hz, 1H), 2.43 (dd, J = 16.8, 2.8 Hz, 1H),

^{13}C NMR (100 MHz, CD_3OD) δ 177.0, 175.1, 160.4, 143.3, 137.7, 134.6, 131.8, 131.6, 128.6, 128.2, 127.8, 114.7, 55.7, 44.2, 39.1.

HRMS (ESI-TOF): m/z Calcd. For $C_{17}H_{16}O_5$: $(M+Na)^+$ 323.0890. Found: 323.0902.

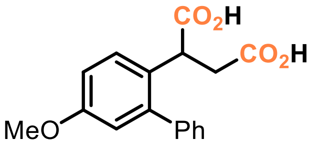

2-(5-methoxy-[1,1'-biphenyl]-2-yl)succinic acid (2p): Prepared according to general procedure from 5-methoxy-2-vinyl-1,1'-biphenyl (0.2 mmol, 42.1 mg, 1.0 equiv). After 24 hours, the product was purified by flash column chromatography (EtOAc/PE 2/1 as the eluent) and obtained as white solid (36.7 mg, 61% yield).

^{1}H NMR (400 MHz, CD_3OD) δ 7.51 – 7.33 (m, 5H), 7.32 – 7.26 (m, 1H), 6.95 – 6.87 (m, 1H), 6.80 – 6.71 (m, 1H), 4.12 (dd, J = 12.0, 4.8 Hz, 1H), 3.79 (s, 3H), 3.01 – 2.89 (dd, J = 16.4, 10.8 Hz, 1H), 2.41 (dd, J = 17.2, 4.0 Hz, 1H).

^{13}C NMR (100 MHz, CD_3OD) δ 177.3, 175.2, 159.9, 144.9, 142.4, 130.5, 129.7, 129.4, 129.1, 128.4, 116.7, 114.6, 55.8, 43.6, 39.2.

HRMS (ESI-TOF): m/z Calcd. For $C_{17}H_{16}O_5$: $(M+Na)^+$ 3230890. Found: 323.0883.

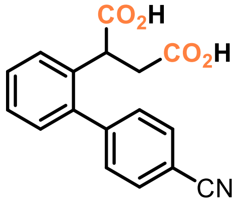

2-(4'-cyano-[1,1'-biphenyl]-2-yl)succinic acid (2q): Prepared according to general procedure from 2'-vinyl-[1,1'-biphenyl]-4-carbonitrile (0.2 mmol, 41.1 mg, 1.0 equiv). After 24 hours, the product was purified by flash column chromatography (EtOAc/PE 2/1 as the eluent) and obtained as white solid (45.0 mg, 76% yield).

^{1}H NMR (400 MHz, CD_3OD) δ 7.84 – 7.78 (m, 2H), 7.64 – 7.58 (m, 2H), 7.45 – 7.32 (m, 3H), 7.28 – 7.22 (m, 1H), 4.10 (dd, J = 9.6, 5.6 Hz, 1H), 3.02 (dd, J = 17.2, 9.6 Hz, 1H), 2.54 (dd, J = 16.8, 5.6 Hz, 1H).

^{13}C NMR (100 MHz, CD_3OD) δ 176.3, 174.9, 147.4, 141.9, 137.4, 133.2, 131.8, 131.2,

129.9, 128.5, 128.2, 119.7, 112.2, 44.2, 38.8.
HRMS (ESI-TOF): *m/z* Calcd. For $C_{17}H_{13}NO_4$: $(M+Na)^+$ 318.0737. Found: 318.0735.

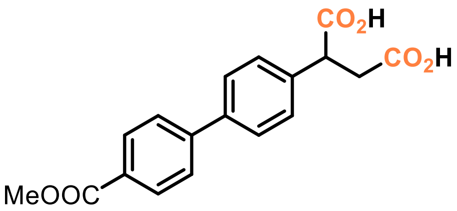

2-(4'-(methoxycarbonyl)-[1,1'-biphenyl]-4-yl)succinic acid (2f): Prepared according to general procedure from methyl 4'-vinyl-[1,1'-biphenyl]-4-carboxylate (0.2 mmol, 47.7 mg, 1.0 equiv). After 24 hours, the product was purified by flash column chromatography (EtOAc/PE 2/1 as the eluent) and obtained as white solid (46.0 mg, 70% yield).
^{1}H NMR (400 MHz, DMSO-*d*$_6$) δ 12.43 (brs, 2H), 8.04 (d, J = 7.2 Hz, 2H), 7.82 (d, J = 8.0 Hz, 2H), 7.71 (d, J = 7.6 Hz, 2H), 7.43 (d, J = 7.6 Hz, 2H), 3.97 (dd, J = 10.0, 5.2 Hz, 1H), 3.88 (s, 3H), 3.00 (dd, J = 17.2, 10.4 Hz, 1H), 2.60 (dd, J = 16.8, 5.2 Hz, 1H).
^{13}C NMR (100 MHz, DMSO-*d*$_6$) δ 174.0, 172.8, 166.1, 144.3, 139.1, 137.7, 129.9, 128.6, 128.5, 127.3, 126.9, 52.2, 46.7, 37.5.
HRMS (ESI-TOF): *m/z* Calcd. For $C_{18}H_{16}O_6$: $(M+Na)^+$ 351.0839. Found: 351.0822.

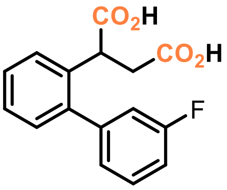

2-(3'-fluoro-[1,1'-biphenyl]-2-yl)succinic acid (2s): Prepared according to general procedure from 3'-fluoro-2-vinyl-1,1'-biphenyl (0.2 mmol, 39.6 mg, 1.0 equiv). After 24 hours, the product was purified by flash column chromatography (EtOAc/PE 2/1 as the eluent) and obtained as white solid (32.5 mg, 56% yield).
^{1}H NMR (400 MHz, CD_3OD) δ 7.49 – 7.28 (m, 4H)7.26 – 7.07 (m, 4H), 4.19 (dd, J = 10.0, 5.2 Hz, 1H), 3.01 (dd, J = 17.2, 10.0 Hz, 1H), 2.49 (dd, J = 17.2, 5.2 Hz, 1H).
^{13}C NMR (100 MHz, CD_3OD) δ 176.5, 174.9, 164.0 (d, J_{FC} = 243.8 Hz), 144.7 (d, J_{FC} = 7.6 Hz), 142.4, 137.5, 131.4, 131.0 (d, J_{FC} = 8.5 Hz), 129.4, 128.4, 128.0, 126.6 (d, J_{FC} = 2.8 Hz), 117.5 (d, J_{FC} = 21.8 Hz), 115.1 (d, J_{FC} = 21.1 Hz), 44.2, 38.9.
^{19}F NMR (376 MHz, CD_3OD) δ -115.9.
HRMS (ESI-TOF): *m/z* Calcd. For $C_{16}H_{13}FO_4$: $(M+Na)^+$ 311.0690. Found: 311.0685.

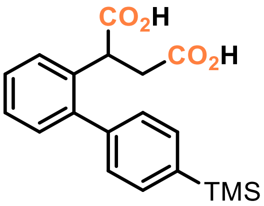

2-(4'-(trimethylsilyl)-[1,1'-biphenyl]-2-yl)succinic acid (2t): Prepared according to

general procedure from trimethyl(2'-vinyl-[1,1'-biphenyl]-4-yl)silane (0.2 mmol, 50.1 mg, 1.0 equiv). After 24 hours, the product was purified by flash column chromatography (EtOAc/PE 2/1 as the eluent) and obtained as white solid (53.1 mg, 78% yield).
^{1}H NMR (400 MHz, CD$_3$OD) δ 7.62 – 7.56 (m, 2H), 7.39 (d, *J* = 8.0 Hz, 3H), 7.35 – 7.27 (m, 2H), 7.24 – 7.16 (m, 1H), 4.25 (dd, *J* = 10.4, 4.8 Hz, 1H), 3.00 (dd, *J* = 17.2, 10.4 Hz, 1H), 2.46 (dd, *J* = 16.8, 4.8 Hz, 1H), 0.30 (s, 9H).
^{13}C NMR (100 MHz, CD$_3$OD) 177.0, 175.2, 143.6, 142.8, 140.1, 137.5, 134.3, 131.6, 129.9, 128.9, 128.2, 127.9, 44.3, 39.1, -1.0.
HRMS (ESI-TOF): *m/z* Calcd. For $C_{19}H_{22}O_4Si$: $(M+Na)^+$ 365.1180. Found: 365.1183.

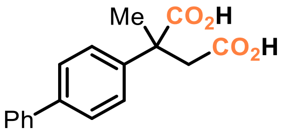

2-([1,1'-biphenyl]-4-yl)-2-methylsuccinic acid (2u): Prepared according to general procedure from 4-(prop-1-en-2-yl)-1,1'-biphenyl (0.2 mmol, 38.9 mg, 1.0 equiv). After 24 hours, the product was purified by flash column chromatography (EtOAc/PE 2/1 as the eluent) and obtained as white solid (37.6 mg, 66% yield).
^{1}H NMR (400 MHz, CD$_3$OD) *δ* 7.63 – 7.52 (m, 4H), 7.52 – 7.46 (m, 2H), 7.46 – 7.37 (m, 2H), 7.36 – 7.27 (m, 1H), 3.24 (d, *J* = 16.4 Hz, 1H), 2.86 (d, *J* = 16.4 Hz, 1H), 1.72 (s, 3H).
^{13}C NMR (100 MHz, CD$_3$OD) *δ* 178.9, 174.8, 143.8, 141.8, 141.0, 129.8, 128.4, 128.0, 127.9, 127.4, 49.1, 44.4, 24.1.
HRMS (ESI-TOF): *m/z* Calcd. For $C_{17}H_{16}O_4$: $(M+Na)^+$ 307.0941. Found: 307.0942.

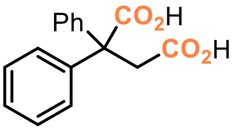

2,2-diphenylsuccinic acid (2aa): Prepared according to general procedure from ethene-1,1-diyldibenzene (0.2 mmol, 36.0 μL, 1.0 equiv). After 12 hours, the product was purified by flash column chromatography (EtOAc/PE 2/1 as the eluent) and obtained as white solid (43.2 mg, 80% yield).
^{1}H NMR (400 MHz, CD$_3$OD) *δ* 7.40 – 7.15 (m, 10H), 3.60 – 3.41 (m, 2H).
^{13}C NMR (100 MHz, CD$_3$OD) *δ* 176.8, 174.4, 144.5, 129.9, 128.8, 127.8, 58.6, 44.8.
HRMS (ESI-TOF): *m/z* Calcd. For $C_{16}H_{14}O_4$: $(M+Na)^+$ 293.0784. Found: 293.0776.

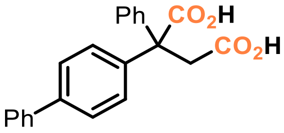

2-([1,1'-biphenyl]-4-yl)-2-phenylsuccinic acid (2ab): Prepared according to general procedure from 4-(1-phenylvinyl)-1,1'-biphenyl (0.2 mmol, 51.3 mg, 1.0 equiv). After

24 hours, the product was purified by flash column chromatography (EtOAc/PE 2/1 as the eluent) and obtained as white solid (53.6mg, 77%yield).
^{1}H NMR (400 MHz, CD$_3$OD) δ 7.58 (d, J = 7.2 Hz, 2H), 7.50 (d, J = 8.0 Hz, 2H), 7.44 – 7.34 (m, 6H), 7.33 – 7.17 (m, 4H), 3.53 (s, 2H).
^{13}C NMR (100 MHz, CD$_3$OD) δ 176.6, 174.3, 144.5, 143.6, 141.8, 140.8, 130.5, 129.9, 129.8, 128.9, 128.3, 127.9, 127.2, 58.3, 44.7.
HRMS (ESI-TOF): *m/z* Calcd. For $C_{22}H_{18}O_4$: $(M+Na)^+$ 369.1097. Found: 369.1091.

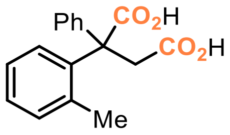

2-phenyl-2-(*o*-tolyl)succinic acid (2ac): Prepared according to general procedure from 1-methyl-2-(1-phenylvinyl)benzene (0.2 mmol, 38.9 mg, 1.0 equiv). After 24 hours, the product was purified by flash column chromatography (EtOAc/PE 2/1 as the eluent) and obtained as white solid (29.9 mg, 53% yield).
^{1}H NMR (400 MHz, CD$_3$OD) δ 7.41 – 7.07 (m, 9H), 3.61 (d, J = 15.6 Hz, 1H), 3.40 (d, J = 15.6 Hz, 1H), 1.90 (s, 3H).
^{13}C NMR (100 MHz, CD$_3$OD) δ 176.8, 174.9, 143.8, 142.0, 138.8, 133.5, 130.0, 129.6, 128.8, 128.3, 127.7, 126.4, 59.2, 45.1, 22.0.
HRMS (ESI-TOF): *m/z* Calcd. For $C_{17}H_{16}O_4$: $(M+Na)^+$ 307.0941. Found: 307.0939.

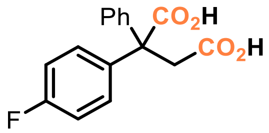

2-(4-fluorophenyl)-2-phenylsuccinic acid (2ad): Prepared according to general procedure from 1-fluoro-4-(1-phenylvinyl)benzene (0.2 mmol, 39.6 mg, 1.0 equiv). After 24 hours, the product was purified by flash column chromatography (EtOAc/PE 2/1 as the eluent) and obtained as white solid (38.0 mg, 66%yield).
^{1}H NMR (400 MHz, CD$_3$OD) δ 7.41 – 7.16 (m, 7H), 6.95 (t, J = 8.4 Hz, 2H), 3.53 (d, J = 16.4 Hz, 1H), 3.40 (d, J = 16.4 Hz, 1H).
^{13}C NMR (100 MHz, CD$_3$OD) δ 176.9, 174.5, 162.9 (d, J_{FC} = 243.2 Hz), 144.5, 140.6 (d, J_{FC} = 3.0 Hz), 132.1 (d, J_{FC} = 7.9 Hz), 129.6, 129.0, 128.0, 115.2 (d, J_{FC} = 21.2 Hz), 58.2, 45.0.
^{19}F NMR (376 MHz, CD$_3$OD) δ -118.9.
HRMS (ESI-TOF): *m/z* Calcd. For $C_{16}H_{13}FO_4$: $(M+Na)^+$ 311.0690. Found: 311.0690.

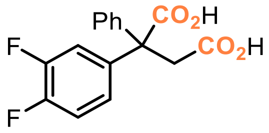

2-(3,4-difluorophenyl)-2-phenylsuccinic acid (2ae): Prepared according to general procedure from 1,2-difluoro-4-(1-phenylvinyl)benzene (0.2 mmol, 43.2 mg, 1.0 equiv).

After 24 hours, the product was purified by flash column chromatography (EtOAc/PE 2/1 as the eluent) and obtained as white solid (44.9 mg, 73% yield).

^{1}H NMR (400 MHz, CD$_3$OD) δ 7.36 – 7.23 (m, 6H), 7.15 – 7.02 (m, 2H), 3.60 (d, J = 16.8 Hz, 1H), 3.37 (d, J = 16.8 Hz, 1H).

^{13}C NMR (100 MHz, CD$_3$OD) δ 176.1, 174.2, 150.5 (dd, J_{FC} = 243.3, 12.5 Hz), 150.2 (dd, J_{FC} = 245.4, 12.7 Hz), 143.9, 142.0 (t, J_{FC} = 4.6 Hz), 129.2(8), 129.2(5), 128.3, 126.7 (dd, J_{FC} = 5.9, 3.5 Hz), 119.9 (d, J_{FC} = 19.1 Hz), 117.1 (d, J_{FC} = 17.1 Hz), 58.0, 44.7.

^{19}F NMR (376 MHz, CD$_3$OD) δ -140.7, -142.9.

HRMS (ESI-TOF): *m/z* Calcd. For $C_{16}H_{12}F_2O_4$: $(M+Na)^+$ 329.0596. Found: 329.0587.

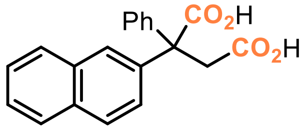

2-(naphthalen-2-yl)-2-phenylsuccinic acid (2af): Prepared according to general procedure from 2-(1-phenylvinyl)naphthalene (0.2 mmol, 46.1 mg, 1.0 equiv). After 24 hours, the product was purified by flash column chromatography (EtOAc/PE 2/1 as the eluent) and obtained as white solid (57.0 mg, 89% yield).

^{1}H NMR (400 MHz, CD$_3$OD) δ 7.85 (d, J = 2.0 Hz, 1H), 7.79 – 7.72 (m, 2H), 7.69 (d, J = 8.8 Hz, 1H), 7.46 – 7.38 (m, 2H), 7.37 – 7.29 (m, 3H), 7.28 – 7.17 (m, 3H), 3.60 (d, J = 8.4 Hz, 2H)

^{13}C NMR (100 MHz, CD$_3$OD) δ 176.5, 174.3, 144.4, 141.7, 134.3, 133.6, 130.0, 129.3, 128.9, 128.5, 128.3, 128.2, 127.9, 127.2, 127.1, 58.6, 44.7.

HRMS (ESI-TOF): *m/z* Calcd. For $C_{20}H_{16}O_4$: $(M+Na)^+$ 343.0941. Found: 343.0934.

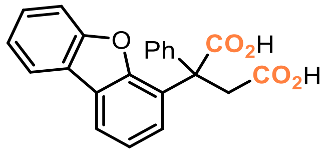

2-(dibenzo[*b*,*d*]furan-4-yl)-2-phenylsuccinic acid (2ag): Prepared according to general procedure from 4-(1-phenylvinyl)dibenzo[*b*,*d*]furan (0.2 mmol, 54.1 mg, 1.0 equiv). After 24 hours, the product was purified by flash column chromatography (EtOAc/PE 2/1 as the eluent) and obtained as white solid (55.7 mg, 77% yield).

^{1}H NMR (400 MHz, CD$_3$OD) δ 7.98 (d, J = 7.6 Hz, 1H), 7.94 (dd, J = 7.6, 1.2 Hz, 1H), 7.50 – 7.45 (m, 3H), 7.43 – 7.40 (m, 2H), 7.34 – 7.22 (m, 5H), 3.87 (s, 2H).

^{13}C NMR (100 MHz, CD$_3$OD) δ 176.8, 175.0, 156.9, 155.1, 142.0, 129.5, 129.2, 129.0(4), 129.0(1), 128.2, 125.8, 125.2, 123.9, 123.3, 121.6, 120.8, 112.4, 57.7, 42.4.

HRMS (ESI-TOF): *m/z* Calcd. For $C_{22}H_{16}O_5$: $(M+Na)^+$ 383.0890. Found: 383.0878.

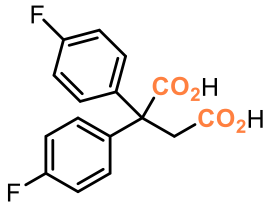

2,2-bis(4-fluorophenyl)succinic acid (2ah): Prepared according to general procedure from 4,4'-(ethene-1,1-diyl)bis(fluorobenzene) (0.2 mmol, 43.2 mg, 1.0 equiv). After 8 hours, the product was purified by flash column chromatography (EtOAc/PE 2/1 as the eluent) and obtained as white solid (39.0 mg, 64% yield).

^{1}H NMR (400 MHz, CD$_3$OD) δ 7.40 – 7.26 (m, 4H), 7.07 – 6.92 (m, 4H), 3.46 (d, J = 3.6 Hz, 2H).

^{13}C NMR (100 MHz, CD$_3$OD) δ 176.5, 174.2, 164.3 (d, J_{FC} = 243.8 Hz), 140.5 (d, J_{FC} = 2.8 Hz), 131.9 (d, J_{FC} = 7.9 Hz), 115.4 (d, J_{FC} = 21.5 Hz), 57.7, 45.0.

^{19}F NMR (376 MHz, CD$_3$OD) δ -117.7.

HRMS (ESI-TOF): *m/z* Calcd. For $C_{16}H_{12}F_2O_4$: $(M+Na)^+$ 329.0596. Found: 329.0585.

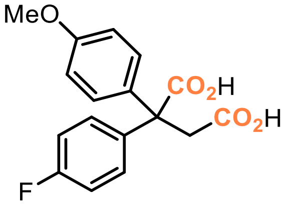

2-(4-fluorophenyl)-2-(4-methoxyphenyl)succinic acid (2ai): Prepared according to general procedure from 1-fluoro-4-(1-(4-methoxyphenyl)vinyl)benzene (0.2 mmol, 45.7 mg, 1.0 equiv). After 24 hours, the product was purified by flash column chromatography (EtOAc/PE 2/1 as the eluent) and obtained as white solid (51.0 mg, 80% yield).

^{1}H NMR (400 MHz, CD$_3$OD) δ 7.18 (dd, J = 8.8, 5.2 Hz, 2H), 7.10 (d, J = 8.8 Hz, 2H), 6.86 (t, J = 8.8 Hz, 2H), 6.73 (d, J = 8.8 Hz, 2H), 3.66 (s, 3H), 3.33 (q, J = 16.0 Hz, 2H).

^{13}C NMR (100 MHz, CD$_3$OD) δ 177.3, 174.7, 162.8 (d, J_{FC} = 243.4 Hz), 159.9, 140.9 (d, J_{FC} = 2.8 Hz), 136.3, 131.9 (d, J_{FC} = 8.0 Hz), 130.7, 115.1 (d, J_{FC} = 21.2 Hz), 114.3, 57.7, 55.7, 45.3.

^{19}F NMR (376 MHz, CD$_3$OD) δ -118.0.

HRMS (ESI-TOF): *m/z* Calcd. For $C_{17}H_{15}FO_5$: $(M+Na)^+$ 341.0796. Found: 341.0798.

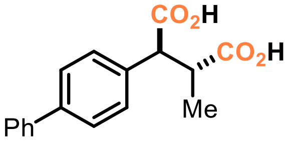

2-([1,1'-biphenyl]-4-yl)-3-methylsuccinic acid (2ba, mixture of isomers, d.r. = 2:1): Prepared according to general procedure from prop-1-en-1-ylbenzene (0.2 mmol, 38.9 mg, 1.0 equiv). After 24 hours, the product was purified by flash column

chromatography (EtOAc/PE 2/1 as the eluent) and obtained as white solid (27.6 mg, 49% yield).

^{1}H NMR (400 MHz, CD$_3$OD) δ 7.65 – 7.50 (m, 4H), 7.47 – 7.35 (m, 4H), 7.35 – 7.26 (m, 1H), 3.77 (t, J = 11.2 Hz, 1H), 3.27 – 3.07 (m, 1H), 1.34 (d, J = 6.8, Hz, 2H), 1.00 (d, J = 7.2 Hz, 1H).

^{13}C NMR (100 MHz, CD$_3$OD) δ 179.5, 178.0, 176.8, 175.7, 141.9, 141.8, 141.7, 137.9, 137.4, 130.0(5), 130.0(1), 129.9, 129.8, 128.4(4), 128.3(8), 128.3(6), 128.0, 127.9, 56.0, 55.4, 44.7, 43.7, 17.1, 16.0.

HRMS (ESI-TOF): *m/z* Calcd. For $C_{17}H_{16}O_4$: $(M+Na)^+$ 307.0941. Found: 307.0941.

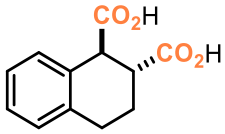

1,2,3,4-tetrahydronaphthalene-1,2-dicarboxylic acid (2bb, mixture of isomers, d.r. = 9:1): Prepared according to general procedure (3DPAFIPN (0.005 mmol, 3.2 mg, 2.5 mol%) and CsF (1.0 mmol, 151.9 mg, 5.0 equiv)) using 1,2-dihydronaphthalene (0.2 mmol, 25.9 µL, 1.0 equiv). After 8 hours, the product was purified by flash column chromatography (EtOAc/PE 2/1 as the eluent) and obtained as white solid (22.2 mg, 50% yield,).

^{1}H NMR (400 MHz, CD$_3$OD) δ 7.38 – 7.34 (m, 0.11H), 7.30 – 7.25 (m, 0.91H), 7.09 – 7.03 (m, 3H), 4.11 (d, J = 5.2 Hz, 0.11H), 4.01 (d, J = 8.4 Hz, 0.90H), 3.09 (ddd, J = 10.4, 8.4, 3.6 Hz, 0.91H), 2.86 – 2.73 (m, 2.10H), 2.35 (ddt, J = 18.8, 12.4, 6.0 Hz, 0.10H), 2.26 – 2.17 (m, 1H), 1.81 (dddd, J = 13.2, 10.0, 9.2, 6.0 Hz, 0.91H).

^{13}C NMR (100 MHz, CD$_3$OD) δ 177.7, 177.4, 137.4, 133.5, 130.1, 129.3, 127.9, 127.1, 48.5, 44.2, 29.3, 25.6.

HRMS (ESI-TOF): *m/z* Calcd. For $C_{12}H_{12}O_4$: $(M+Na)^+$ 243.0628. Found: 243.0628.

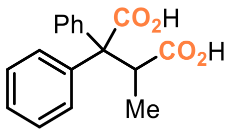

3-methyl-2,2-diphenylsuccinic acid (2bc): Prepared according to general procedure from prop-1-ene-1,1-diyldibenzene (0.2 mmol, 38.9 mg, 1.0 equiv). After 24 hours at 50 °C, the product was purified by flash column chromatography (EtOAc/PE 2/1 as the eluent) and obtained as white solid (32.0 mg, 56% yield).

^{1}H NMR (400 MHz, CD$_3$OD) δ 7.41 – 7.18 (m, 10H), 4.09 (qd, J = 6.8, 2.4 Hz, 1H), 1.22 – 1.10 (m, 3H).

^{13}C NMR (100 MHz, CD$_3$OD) δ 178.3, 177.4, 143.3, 142.1, 132.3, 130.5, 128.8, 128.0, 127.9, 127.6, 63.7, 45.8, 15.3.

HRMS (ESI-TOF): *m/z* Calcd. For $C_{17}H_{16}O_4$: $(M+Na)^+$ 307.0941. Found: 307.0932.

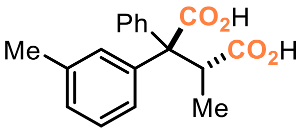

3-methyl-2-phenyl-2-(*m*-tolyl)succinic acid (2bd, mixture of isomers, d.r. = 1:1): Prepared according to general procedure from 1-methyl-3-(1-phenylprop-1-en-1-yl)benzene (0.2 mmol, 41.7 mg, 1.0 equiv). After 24 hours at 50 °C, the product was purified by flash column chromatography (EtOAc/PE 2/1 as the eluent) and obtained as white solid (24.2 mg, 41% yield).
^{1}H NMR (400 MHz, CD_3OD) δ 7.38 (dd, J = 8.4, 1.2 Hz, 1H), 7.31 – 6.99 (m, 8H), 3.98 (qd, J = 7.2, 4.0 Hz, 1H), 2.28 (s, 1.51H), 2.25 (s, 1.50H), 1.22 (s, 1.49H), 1.22 – 1.19 (m, 1.49H).
^{13}C NMR (100 MHz, CD_3OD) δ 179.2, 179.1, 177.8(0), 177.7(7), 143.7, 143.6, 142.7, 142.5, 138.6, 137.4, 132.6, 132.1, 130.9, 130.4, 128.9, 128.8, 128.7, 128.2, 127.9(2), 127.8(5), 127.5, 127.2, 63.9(9), 63.9(5), 46.8(4), 46.7(9), 21.7, 21.6, 15.6, 15.5.
HRMS (ESI-TOF): *m/z* Calcd. For $C_{18}H_{18}O_4$: $(M+Na)^+$ 321.1097. Found: 321.1089.

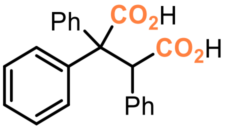

2,2,3-triphenylsuccinic acid (2be): Prepared according to general procedure from ethene-1,1,2-triyltribenzene (0.2 mmol, 51.3 mg, 1.0 equiv). After 24 hours at 50 °C, the product was purified by flash column chromatography (EtOAc/PE 2/1 as the eluent) and obtained as white solid (53.4 mg, 77% yield).
^{1}H NMR (400 MHz, CD_3OD) δ 7.33 – 7.29 (m, 2H), 7.28 – 7.24 (m, 2H), 7.23 – 7.09 (m, 7H), 7.01 (t, J = 8.0 Hz, 2H), 6.84 – 6.79 (m, 2H), 5.48 (s, 1H).
^{13}C NMR (100 MHz, CD_3OD) δ 177.2, 176.9, 142.6, 141.8, 137.5, 132.1, 131.9, 131.5, 128.6, 128.3, 128.2, 128.1, 127.6, 66.1, 58.9.
HRMS (ESI-TOF): *m/z* Calcd. For $C_{22}H_{18}O_4$: $(M+Na)^+$ 321.1097. Found: 321.1095.

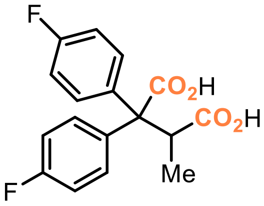

2,2-bis(4-fluorophenyl)-3-methylsuccinic acid (2bf): Prepared according to general procedure from 4,4'-(prop-1-ene-1,1-diyl)bis(fluorobenzene) (0.2 mmol, 46.1 mg, 1.0 equiv). After 24 hours at 50 °C, the product was purified by flash column chromatography (EtOAc/PE 2/1 as the eluent) and obtained as white solid (38.8 mg, 61% yield).
^{1}H NMR (400 MHz, CD_3OD) δ 7.43 – 7.30 (m, 4H), 7.03 (t, J = 8.0 Hz, 2H), 6.96 (t, J = 8.0 Hz, 2H), 4.06 (q, J = 6.8 Hz, 1H), 1.16 (d, J = 6.8 Hz, 3H).

^{13}C NMR (100 MHz, CD_3OD) δ 178.0, 177.1, 163.1 (d, J_{FC} = 244.2 Hz), 162.9 (d, J_{FC} = 243.7 Hz), 139.1, 137.9, 134.1 (d, J_{FC} = 7.8 Hz), 132.4 (d, J_{FC} = 7.9 Hz), 115.5 (d, J_{FC} = 21.4 Hz), 114.5 (d, J_{FC} = 22.1 Hz), 62.7, 45.9, 15.2.
^{19}F NMR (376 MHz, CD_3OD) δ -117.8, -118.5.
HRMS (ESI-TOF): *m/z* Calcd. For $C_{17}H_{14}F_2O_4$: $(M+Na)^+$ 343.0952. Found: 343.0945.

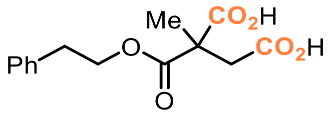

2-methyl-2-(phenethoxycarbonyl)succinic acid (2ca): Prepared according to general procedure (3DPAFIPN (0.005 mmol, 3.2 mg, 2.5 mol%) and CsF (1.0 mmol, 151.9 mg, 5.0 equiv)) using phenethyl methacrylate (0.2 mmol, 38.0 mg, 1.0 equiv). After 8 hours, the product was purified by flash column chromatography (EtOAc/PE 2/1 as the eluent) and obtained as white solid (31.2 mg, 56% yield).
^{1}H NMR (400 MHz, CD_3OD) δ 7.30 – 7.17 (m, 5H), 4.31 (t, J = 6.8 Hz, 2H), 2.99 – 2.88 (m, 3H), 2.81 (d, J = 17.2 Hz, 1H), 1.48 (s, 3H).
^{13}C NMR (100 MHz, CD_3OD) δ 174.3, 173.8, 172.9, 139.2, 130.0, 129.5, 127.5, 67.2, 52.7, 40.9, 35.8, 20.8.
HRMS (ESI-TOF): *m/z* Calcd. For $C_{14}H_{16}O_6$: $(M+Na)^+$ 303.0839. Found: 303.0836.

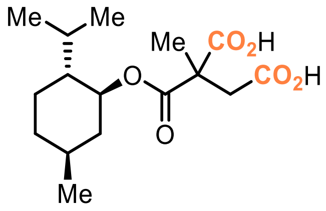

2-((((1*S*,2*R*,5*S*)-2-isopropyl-5-methylcyclohexyl)oxy)carbonyl)-2-methylsuccinic acid (2cb, mixture of isomers, d.r. = 1.4:1): Prepared according to general procedure (3DPAFIPN (0.005 mmol, 3.2 mg, 2.5 mol%) and CsF (1.0 mmol, 151.9 mg, 5.0 equiv)) using (1*S*,2*R*,5*S*)-2-isopropyl-5-methylcyclohexyl methacrylate (0.2 mmol, 44.9 mg, 1.0 equiv). After 8 hours, the product was purified by flash column chromatography (EtOAc/PE 2/1 as the eluent) and obtained as white solid (14.6 mg, 23% yield).
^{1}H NMR (400 MHz, CD_3OD) δ 4.67 (qd, J = 10.4, 4.4 Hz, 1H), 3.02 – 2.74 (m, 2H), 2.02 – 1.91 (m, 2H), 1.70 (d, J = 11.2 Hz, 2H), 1.51 (d, J = 2.8 Hz, 3H), 1.48 – 1.34 (m, 2H), 1.08 (q, J = 12.8 Hz, 1H), 1.00 – 0.86 (m, 8H), 0.74 (dd, J = 6.8, 5.2 Hz, 3H).
^{13}C NMR (100 MHz, CD_3OD) δ 174.6, 174.0, 172.6, 172.5, 76.8, 76.7, 52.9, 52.8, 48.4, 41.3, 41.0, 35.4, 32.6, 27.0, 26.9, 24.3, 24.2, 22.4, 21.2, 21.1, 20.8(4), 20.7(9), 16.4, 16.3.
HRMS (ESI-TOF): *m/z* Calcd. For $C_{16}H_{26}O_6$: $(M+Na)^+$ 337.1622. Found: 337.1626.

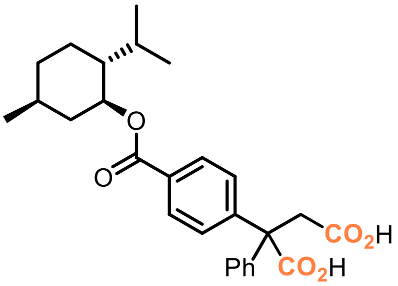

2-(4-((((1*S*,2*R*,5*S*)-2-isopropyl-5-methylcyclohexyl)oxy)carbonyl)phenyl)-2-phenylsuccinic acid (2cc, d.r. > 20:1): Prepared according to general procedure from (1*S*,2*R*,5*S*)-2-isopropyl-5-methylcyclohexyl 4-(1-phenylvinyl)benzoate (0.2 mmol, 72.5 mg, 1.0 equiv). After 24 hours, the product was purified by flash column chromatography (EtOAc/PE 2/1 as the eluent) and obtained as white solid (53.9 mg, 60% yield).

^{1}H NMR (400 MHz, CD$_3$OD) δ 7.88 (d, J = 8.4 Hz, 2H), 7.47 (d, J = 8.4 Hz, 2H), 7.36 – 7.13 (m, 5H), 4.98 – 4.90 (m, 1H), 3.60 (d, J = 16.4 Hz, 1H), 3.44 (d, J = 16.8 Hz, 1H), 2.11 – 2.00 (m, 1H), 2.00 – 1.87 (m, 1H), 1.78 – 1.71 (m, 2H), 1.68 – 1.49 (m, 2H), 1.23 – 1.06 (m, 2H), 1.01 – 0.88 (m, 7H), 0.80 (d, J = 6.8 Hz, 3H).

^{13}C NMR (100 MHz, CD$_3$OD) δ 176.0, 174.1, 167.4, 145.0, 144.1, 130.6, 130.1, 129.7, 129.1, 128.2, 76.1, 58.7, 44.5, 42.1, 35.4, 32.8, 27.8, 24.7, 22.5, 21.0, 16.9.

HRMS (ESI-TOF): *m/z* Calcd. For $C_{27}H_{32}O_6$: $(M+Na)^+$ 475.2091. Found: 475.2088.

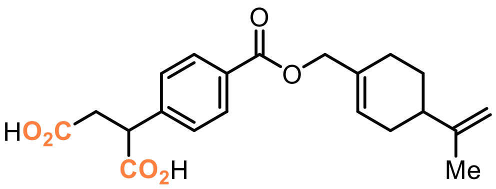

2-(4-(((4-(prop-1-en-2-yl)cyclohex-1-en-1-yl)methoxy)carbonyl)phenyl)succinic acid (2cd, mixture of isomers, d.r. = 1.8:1): Prepared according to general procedure (3DPAFIPN (0.005 mmol, 3.2 mg, 2.5 mol%) and CsF (1.0 mmol, 151.9 mg, 5.0 equiv)) using (4-(prop-1-en-2-yl)cyclohex-1-en-1-yl)methyl 4-vinylbenzoate (0.2 mmol, 56.5 mg, 1.0 equiv). After 8 hours, the product was purified by flash column chromatography (EtOAc/PE 2/1 as the eluent) and obtained as white solid (26.2 mg, 35% yield).

^{1}H NMR (400 MHz, CD$_3$OD) δ 7.87 (t, J = 9.2 Hz, 2H), 7.34 (t, J = 9.2 Hz, 2H), 5.75 (s, 1H), 4.60 (t, J = 11.2 Hz, 4H), 4.02 (dd, J = 9.6, 5.2 Hz, 1H), 3.03 (dt, J = 21.2, 10.8 Hz, 1H), 2.57 (dd, J = 17.2, 5.2 Hz, 1H), 2.14 – 1.99 (m, 4H), 1.90 (dd, J = 17.2, 11.2 Hz, 1H), 1.77 (d, J = 11.2 Hz, 1H), 1.63 (d, J = 10.8 Hz, 3H), 1.48 – 1.36 (m, 1H).

^{13}C NMR (100 MHz, CD$_3$OD) δ 175.8, 174.8, 167.5, 150.7, 145.3, 134.0, 130.9, 130.7, 129.3, 126.7, 109.4, 69.9, 42.2, 38.4, 31.6, 28.6, 27.4, 21.0.

HRMS (ESI-TOF): *m/z* Calcd. For $C_{21}H_{24}O_6$: $(M+Na)^+$ 395.1465. Found: 395.1450.

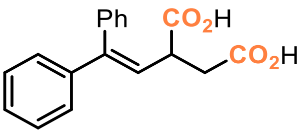

2-(2,2-diphenylvinyl)succinic acid (4a): Prepared according to general procedure from buta-1,3-diene-1,1-diyldibenzene (0.2 mmol, 41.3 mg, 1.0 equiv). After 12 hours at 50 °C, the product was purified by flash column chromatography (EtOAc/PE 2/1 as the eluent) and obtained as white solid (53.1 mg, 90% yield).
^{1}H NMR (400 MHz, CD_3OD) δ 7.42 – 7.32 (m, 3H), 7.26 – 7.17 (m, 7H), 6.04 (d, J = 10.4 Hz, 1H), 3.57 (ddd, J = 10.4, 8.4, 5.6 Hz, 1H), 2.78 (dd, J = 16.4, 8.4 Hz, 1H), 2.51 (dd, J = 16.4, 5.6 Hz, 1H).
^{13}C NMR (100 MHz, CD_3OD) δ 176.7, 174.9, 146.4, 143.2, 140.5, 130.8, 129.4, 129.3, 129.2, 128.6, 128.4, 126.0, 43.6, 37.8.
HRMS (ESI-TOF): *m/z* Calcd. For $C_{18}H_{16}O_4$: $(M+Na)^+$ 319.0941. Found: 319.0941.

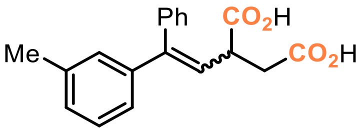

2-(2-phenyl-2-(*m*-tolyl)vinyl)succinic acid (4b, mixture of isomers, *E/Z* = 1.2:1): Prepared according to general procedure from 1-methyl-3-(1-phenylbuta-1,3-dien-1-yl)benzene (0.2 mmol, 44.1 mg, 1.0 equiv). After 12 hours at 50 °C, the product was purified by flash column chromatography (EtOAc/PE 2/1 as the eluent) and obtained as white solid (44.0 mg, 71% yield).
^{1}H NMR (400 MHz, CD_3OD) δ 7.41 – 7.11 (m, 7H), 7.01 (dd, J = 22.4, 8.0 Hz, 2H), 6.02 (ddd, J = 10.4, 5.2, 2.0 Hz, 1H), 3.62 – 3.51 (m, 1H), 2.78 (ddd, J = 16.0, 8.0, 5.2 Hz, 1H), 2.53 (t, J = 4.8 Hz, 0.55H), 2.49 (t, J = 4.8 Hz, 0.45H), 2.35 (d, J = 4.8 Hz, 1.38H), 2.26 (d, J = 4.8 Hz, 1.62H).
^{13}C NMR (100 MHz, CD_3OD) δ 176.7(9), 176.7(6), 174.9, 146.5, 143.3, 143.2, 140.7, 140.5, 139.2, 138.9, 131.4, 130.8, 129.4, 129.3(2), 129.2(6), 129.2, 129.1, 129.0, 128.6, 128.4, 127.9, 125.9, 125.7, 43.6(2), 43.6(1), 37.8(9), 37.8(7), 21.5(0), 21.4(5).
HRMS (ESI-TOF): *m/z* Calcd. For $C_{19}H_{18}O_4$: $(M+Na)^+$ 333.1097. Found: 333.1093.

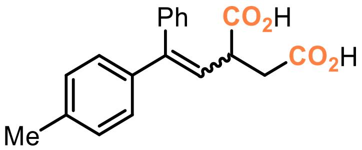

2-(2-phenyl-2-(*p*-tolyl)vinyl)succinic acid (4c, mixture of isomers, *E/Z* = 1.3:1): Prepared according to general procedure from 1-methyl-4-(1-phenylbuta-1,3-dien-1-yl)benzene (0.2 mmol, 44.1 mg, 1.0 equiv). After 12 hours at 50 °C, the product was purified by flash column chromatography (EtOAc/PE 2/1 as the eluent) and obtained as white solid (54.5 mg, 88% yield).
^{1}H NMR (400 MHz, CD_3OD) δ 7.43 – 7.33 (m, 1H), 7.30 – 7.05 (m, 8H), 6.00 (d, J = 10.6 Hz, 1H), 3.61 – 3.50 (m, 1H), 2.77 (ddd, J = 16.4, 8.4, 2.0 Hz, 1H), 2.51 (dd, J = 5.6, 2.4 Hz, 0.56H), 2.47 (dd, J = 5.6, 2.4 Hz, 0.42H), 2.37 (s, 1.71H), 2.29 (s, 1.27H).
^{13}C NMR (100 MHz, CD_3OD) δ 176.8, 175.0, 146.4, 146.3, 143.4, 140.7, 140.4, 138.5(4), 138.4(6), 137.6, 130.9, 130.8, 130.0, 129.8, 129.4, 129.2, 128.6, 128.5, 128.3, 125.9, 125.2, 43.7, 37.9, 21.3, 21.1.

HRMS (ESI-TOF): *m/z* Calcd. For $C_{19}H_{18}O_4$: $(M+Na)^+$ 333.1097. Found: 333.1093.

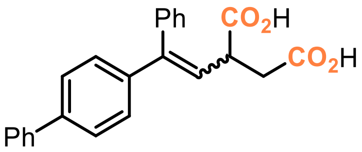

2-(2-([1,1'-biphenyl]-4-yl)-2-phenylvinyl)succinic acid (4d, mixture of isomers, *E/Z* = 2.6:1): Prepared according to general procedure from 4-(1-phenylbuta-1,3-dien-1-yl)-1,1'-biphenyl (0.2 mmol, 56.5 mg, 1.0 equiv). After 24 hours at 50 °C, the product was purified by flash column chromatography (EtOAc/PE 2/1 as the eluent) and obtained as white solid (71.2 mg, 96% yield).

^{1}H NMR (400 MHz, CD_3OD) δ 7.67 – 7.51 (m, 4H), 7.45 – 7.24 (m, 10H), 6.12 (d, *J* = 10.4 Hz, 0.28H), 6.06 (d, *J* = 10.4 Hz, 0.72H), 3.70 – 3.63 (m, 0.71H), 3.62 – 3.54 (m, 0.29H), 2.85 – 2.76 (m, 1H), 2.58 – 2.49 (m, 1H).

^{13}C NMR (100 MHz, CD_3OD) δ 176.7, 175.0, 174.9, 146.1, 145.9, 143.2, 142.0, 141.9, 141.7, 141.6, 141.5, 140.4, 139.5, 131.4, 130.9, 129.9, 129.8, 129.5, 129.3, 128.9, 128.7, 128.6, 128.4(3), 128.3(8), 128.0, 127.8, 127.7, 126.2, 125.9, 43.7, 37.8.

HRMS (ESI-TOF): *m/z* Calcd. For $C_{24}H_{20}O_4$: $(M+Na)^+$ 395.1254. Found: 395.1251.

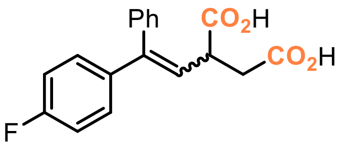

2-(2-(4-fluorophenyl)-2-phenylvinyl)succinic acid (4e, mixture of isomers, *E/Z* = 1.3:1): Prepared according to general procedure from 1-fluoro-4-(1-phenylbuta-1,3-dien-1-yl)benzene (0.2 mmol, 44.9 mg, 1.0 equiv). After 12 hours at 50 °C, the product was purified by flash column chromatography (EtOAc/PE 2/1 as the eluent) and obtained as white solid (48.9 mg, 78% yield).

^{1}H NMR (400 MHz, CD_3OD) δ 7.42 – 7.36 (m, 1H), 7.28 – 7.11 (m, 7H), 7.00 – 6.96 (m, 1H), 6.05 – 6.00 (m, 1H), 3.58 – 3.53 (m, 1H), 2.79 (dt, *J* = 15.6, 7.2 Hz, 1H), 2.83 – 2.75 (m, 0.56H), 2.54 – 2.49 (m, 0.42H).

^{13}C NMR (100 MHz, CD_3OD) δ 176.6, 176.5, 174.9, 163.8 (d, J_{FC} = 244.5 Hz), 163.6 (d, J_{FC} = 244.1 Hz), 145.4, 145.3, 143.1, 140.3, 139.5 (d, J_{FC} = 3.1 Hz), 136.6 (d, J_{FC} = 3.1 Hz), 132.8 (d, J_{FC} = 8.1 Hz), 130.9, 130.8, 130.2 (d, J_{FC} = 8.1 Hz), 129.5, 129.3, 128.8, 128.4, 126.6, 126.2, 116.2 (d, J_{FC} = 23.4 Hz), 115.9 (d, J_{FC} = 21.7 Hz), 43.6(6), 43.6(5), 37.8(1), 37.7(6).

^{19}F NMR (376 MHz, CD_3OD) δ -116.1, -116.5.

HRMS (ESI-TOF): *m/z* Calcd. For $C_{18}H_{15}FO_4$: $(M+Na)^+$ 337.0847. Found: 337.0846.

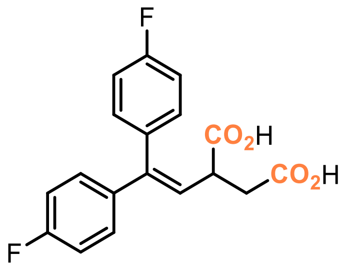

2-(2,2-bis(4-fluorophenyl)vinyl)succinic acid (4f): Prepared according to general procedure from 4,4'-(buta-1,3-diene-1,1-diyl)bis(fluorobenzene) (0.2 mmol, 48.5 mg, 1.0 equiv). After 12 hours at 50 °C, the product was purified by flash column chromatography (EtOAc/PE 2/1 as the eluent) and obtained as white solid (60.6 mg, 91% yield).

^{1}H NMR (400 MHz, CD_3OD) δ 7.21 – 7.00 (m, 6H), 6.90 (t, J = 8.4 Hz, 2H), 5.91 (d, J = 10.4 Hz, 1H), 3.43 (dt, J = 10.4, 6.8 Hz, 1H), 2.68 (dd, J = 16.4, 8.0 Hz, 1H), 2.42 (dd, J = 16.4, 5.6 Hz, 1H).

^{13}C NMR (100 MHz, CD_3OD) δ 176.5, 174.9, 163.8 (d, J_{FC} = 244.6 Hz), 163.7(d, J_{FC} = 244.2 Hz), 144.3, 139.4 (d, J_{FC} = 2.8 Hz), 136.4 (d, J_{FC} = 3.0 Hz), 132.8 (d, J_{FC} = 7.9 Hz), 130.2 (d, J_{FC} = 8.1 Hz), 126.7, 116.3 (d, J_{FC} = 31.5 Hz), 116.0 (d, J_{FC} = 31.5 Hz), 43.7, 37.7.

^{19}F NMR (376 MHz, CD_3OD) δ -116.2, -116.6.

HRMS (ESI-TOF): *m/z* Calcd. For $C_{18}H_{14}F_2O_4$: $(M+Na)^+$ 55.0752. Found: 355.0751.

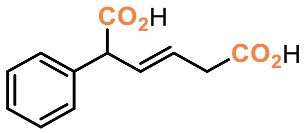

2-phenylhex-3-enedioic acid (5g, *E/Z* > 20:1): Prepared according to general procedure from buta-1,3-dien-1-ylbenzene (0.2 mmol, 26.0 mg, 1.0 equiv). After 60 hours, the product was purified by flash column chromatography (EtOAc/PE 2/1 as the eluent) and obtained as colourless liquid (12.7 mg, 29% yield).

^{1}H NMR (400 MHz, CD_3OD) δ 7.31 (d, J = 4.4 Hz, 4H), 7.26 – 7.22 (m, 1H), 5.99 (dd, J = 15.6, 8.4 Hz, 1H), 5.69 (dt, J = 14.8, 7.2 Hz, 1H), 4.30 (d, J = 8.4 Hz, 1H), 3.08 (d, J = 7.2 Hz, 2H).

^{13}C NMR (100 MHz, CD_3OD) δ 176.1, 175.3, 140.2, 132.8, 129.6, 129.0, 128.2, 126.4, 55.9, 38.4.

HRMS (ESI-TOF): *m/z* Calcd. For $C_{12}H_{12}O_4$: $(M+Na)^+$ 243.0628. Found: 243.0629.

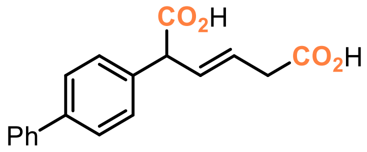

2-([1,1'-biphenyl]-4-yl)hex-3-enedioic acid (5h, *E/Z* > 20:1): Prepared according to general procedure from 4-(buta-1,3-dien-1-yl)-1,1'-biphenyl (0.2 mmol, 41.3 mg, 1.0 equiv). After 60 hours, the product was purified by flash column chromatography

(EtOAc/PE 2/1 as the eluent) and obtained as white solid (36.5 mg, 62% yield).

^{1}H NMR (400 MHz, CD$_3$OD) δ 7.58 (t, J = 8.0 Hz, 4H), 7.40 (dd, J = 8.0, 5.6 Hz, 4H), 7.31 (t, J = 7.2 Hz, 1H), 6.03 (dd, J = 15.6, 8.4 Hz, 1H), 5.73 (dt, J = 14.8, 6.8 Hz, 1H), 4.36 (d, J = 8.4 Hz, 1H), 3.10 (d, J = 7.2 Hz, 2H).

^{13}C NMR (100 MHz, CD$_3$OD) δ 176.1, 175.4, 142.0, 141.4, 139.3, 132.7, 129.8, 129.5, 128.3, 128.2, 127.9, 126.5, 55.6, 38.5.

HRMS (ESI-TOF): *m/z* Calcd. For $C_{18}H_{16}O_4$: $(M+Na)^+$ 319.0941. Found: 319.0931.

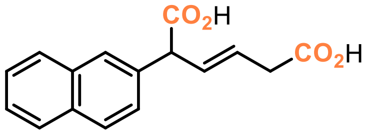

2-(naphthalen-2-yl)hex-3-enedioic acid (5i, *E/Z* > 20:1): Prepared according to general procedure from **2-(buta-1,3-dien-1-yl)naphthalene** (0.2 mmol, 36.1 mg, 1.0 equiv). After 60 hours, the product was purified by flash column chromatography (EtOAc/PE 2/1 as the eluent) and obtained as white solid (43.1 mg, 80% yield).

^{1}H NMR (400 MHz, CD$_3$OD) δ 7.89 – 7.74 (m, 6H), 7.51 – 7.42 (m, 4H), 6.09 (ddt, J = 15.2, 8.4, 1.6 Hz, 1H), 5.81 – 5.71 (m, 1H), 4.49 (d, J = 8.4 Hz, 1H), 3.12 (d, J = 6.8 Hz, 2H).

^{13}C NMR (100 MHz, CD$_3$OD) δ 176.1, 175.4, 137.6, 135.0, 134.0, 132.7, 129.2, 128.8, 128.6, 127.7, 127.2, 127.1, 126.9, 126.7, 56.0, 38.5.

HRMS (ESI-TOF): *m/z* Calcd. For $C_{16}H_{14}O_4$: $(M+Na)^+$ 293.0784. Found: 293.0779

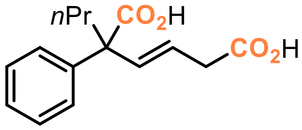

2-phenyl-2-propylhex-3-enedioic acid (5j, *E/Z* > 20:1): Prepared according to general procedure from hepta-1,3-dien-4-ylbenzene (0.2 mmol, 34.5 mg, 1.0 equiv). After 60 hours, the product was purified by flash column chromatography (EtOAc/PE 2/1 as the eluent) and obtained as colourless liquid (46.2 mg, 88% yield).

^{1}H NMR (400 MHz, CD$_3$OD) δ 7.31 – 7.20 (m, 5H), 6.15 (d, J = 16.0 Hz, 1H), 5.53 (dt, J = 16.0, 7.2 Hz, 1H), 3.11 (d, J = 7.2 Hz, 2H), 2.15 – 1.98 (m, 2H), 1.30 – 1.18 (m, 2H), 0.90 (t, J = 7.2 Hz, 3H).

^{13}C NMR (100 MHz, CD$_3$OD) δ 178.0, 175.5, 144.0, 137.1, 129.2, 128.4, 127.7, 125.0, 58.1, 40.6, 38.9, 19.3, 14.9.

HRMS (ESI-TOF): *m/z* Calcd. For $C_{15}H_{18}O_4$: $(M+Na)^+$ 285.1097. Found: 285.1099.

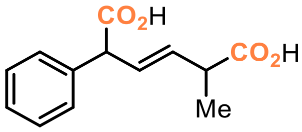

2-methyl-5-phenylhex-3-enedioic acid (5k, *E/Z* > 20:1): Prepared according to general procedure from penta-1,3-dien-1-ylbenzene (0.2 mmol, 28.8 mg, 1.0 equiv).

After 60 hours, the product was purified by flash column chromatography (EtOAc/PE 2/1 as the eluent) and obtained as white solid (30.4 mg, 65% yield).

^{1}H NMR (400 MHz, CD$_3$OD) δ 7.27 (dt, J = 23.6, 2.4 Hz, 5H), 5.99 (dd, J = 15.6, 8.4 Hz, 1H), 5.69 (dd, J = 15.6, 7.6 Hz, 1H), 4.34 – 4.25 (m, 1H), 3.15 (p, J = 6.8 Hz, 1H), 1.27 – 1.19 (m, 3H).

^{13}C NMR (100 MHz, CD$_3$OD) δ 178.2, 176.1, 140.2, 133.2, 130.7, 129.6, 129.0, 128.2, 55.8, 43.8, 17.6.

HRMS (ESI-TOF): *m/z* Calcd. For $C_{13}H_{14}O_4$: $(M+Na)^+$ 257.0784. Found: 257.0784.

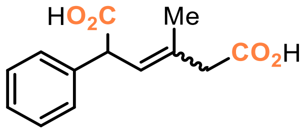

4-methyl-2-phenylhex-3-enedioic acid (5l, mixture of isomers, *E/Z* = 6:1): Prepared according to general procedure from 3-methylbuta-1,3-dien-1-ylbenzene (0.2 mmol, 28.8 mg, 1.0 equiv). After 60 hours, the product was purified by flash column chromatography (EtOAc/PE 2/1 as the eluent) and obtained as white solid (33.3mg, 71% yield).

^{1}H NMR (400 MHz, CD$_3$OD) δ 7.38 – 7.26 (m, 4H), 7.25 – 7.15 (m, 1H), 5.84 – 5.79 (m, 1H), 4.55 – 4.48 (m, 1H), 3.10 (s, 0.24H), 3.06 (s, 1.76H), 1.85 (d, J = 1.6 Hz, 0.33H), 1.75 (d, J = 1.2 Hz, 2.67H).

^{13}C NMR (100 MHz, CD$_3$OD) δ 176.5, 175.3, 140.7, 133.1, 129.6, 129.4, 128.9, 128.0, 127.8, 127.6, 51.6, 45.5, 16.8.

HRMS (ESI-TOF): *m/z* Calcd. For $C_{13}H_{14}O_4$: $(M+Na)^+$ 257.0784. Found: 257.0782.

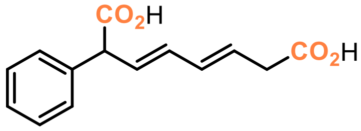

2-phenylocta-3,5-dienedioic acid (5m, *E/Z* > 20:1): Prepared according to general procedure from hexa-1,3,5-trien-1-ylbenzene (0.2 mmol, 31.2 mg, 1.0 equiv). After 60 hours, the product was purified by flash column chromatography (EtOAc/PE 2/1 as the eluent) and obtained as white solid (18.3 mg, 37% yield).

^{1}H NMR (400 MHz, CD$_3$OD) δ 7.28 (dt, J = 20.0, 4.0 Hz, 5H), 6.23 – 6.09 (m, 2H), 6.02 (dd, J = 14.0, 8.0 Hz, 1H), 5.73 (dt, J = 14.0, 7.2 Hz, 1H), 4.32 (d, J = 8.0 Hz, 1H), 3.09 (d, J = 7.2 Hz, 2H).

^{13}C NMR (100 MHz, CD$_3$OD) δ 176.1, 175.4, 140.3, 134.0, 133.0, 132.0, 129.7, 129.0, 128.2, 127.0, 55.9, 38.5.

HRMS (ESI-TOF): *m/z* Calcd. For $C_{14}H_{14}O_4$: $(M+Na)^+$ 269.0784. Found: 269.0774.

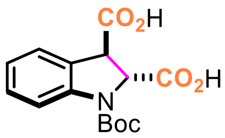

1-(*tert*-butoxycarbonyl)indoline-2,3-dicarboxylic acid (7a, d.r. > 20:1): Prepared according to general procedure (3DPAFIPN (0.005 mmol, 3.2 mg, 2.5 mol%) and CsF (1.0 mmol, 151.9 mg, 5.0 equiv)) using *tert*-butyl 1*H*-indole-1-carboxylate (0.2 mmol, 43.5 mg, 1.0 equiv). After 8 hours, the product was purified by flash column chromatography (EtOAc/PE 2/1 as the eluent) and obtained as white solid (36.7 mg, 60% yield).

^{1}H NMR (400 MHz, CD_3OD) δ 7.83 (brs, 1H), 7.40 (dd, J = 7.2, 4.0 Hz, 1H), 7.26 (q, J = 7.6 Hz, 1H), 7.01 (dp, J = 12.8, 5.2 Hz, 1H), 5.21 (s, 1H), 4.18 (s, 1H), 1.53 (s, 9H).

^{13}C NMR (100 MHz, CD_3OD) δ 174.2, 173.3, 153.0, 143.4, 130.1, 127.8, 126.3, 123.9, 115.5, 82.8, 64.3, 51.2, 28.5.

HRMS (ESI-TOF): *m/z* Calcd. For $C_{15}H_{17}NO_6$: $(M+Na)^+$ 330.0948. Found: 330.0944.

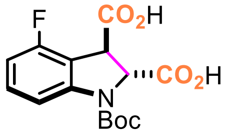

1-(*tert*-butoxycarbonyl)-4-fluoroindoline-2,3-dicarboxylic acid (7b, d.r. > 20:1): Prepared according to general procedure (3DPAFIPN (0.005 mmol, 3.2 mg, 2.5 mol%) and CsF (1.0 mmol, 151.9 mg, 5.0 equiv)) using *tert*-butyl 4-fluoro-1*H*-indole-1-carboxylate (0.2 mmol, 47.1 mg, 1.0 equiv). After 8 hours, the product was purified by flash column chromatography (EtOAc/PE 2/1 as the eluent) and obtained as white solid (28.7 mg, 44% yield).

^{1}H NMR (400 MHz, DMSO-d_6) δ 7.75 – 7.48 (m, 1H), 7.33 (q, J = 8.0 Hz, 1H), 6.85 (t, J = 8.8 Hz, 1H), 4.98 (s, 1H), 4.30 – 4.19 (m, 1H), 1.49 (brs, 9H).

^{13}C NMR (100 MHz, DMSO-d_6) δ 171.3, 171.1, 158.7 (d, J_{FC} = 245.4 Hz), 150.7, 144.8, 131.4 (d, J_{FC} = 8.1 Hz), 113.3 (d, J_{FC} = 20.7 Hz), 110.1, 109.7 (d, J_{FC} = 19.8 Hz), 81.4, 64.1, 46.6, 27.7.

^{19}F NMR (376 MHz, DMSO-d_6) δ -119.2.

HRMS (ESI-TOF): *m/z* Calcd. For $C_{15}H_{16}FNO_6$: $(M+Na)^+$ 348.0854. Found: 348.0851.

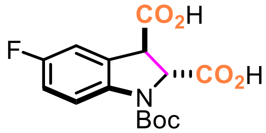

1-(*tert*-butoxycarbonyl)-5-fluoroindoline-2,3-dicarboxylic acid (7c, d.r. > 20:1): Prepared according to general procedure (3DPAFIPN (0.005 mmol, 3.2 mg, 2.5 mol%) and CsF (1.6 mmol, 243.0 mg, 8.0 equiv)) using *tert*-butyl 5-fluoro-1*H*-indole-1-carboxylate (0.2 mmol, 47.1 mg, 1.0 equiv). After 8 hours, the product was purified by flash column chromatography (EtOAc/PE 2/1 as the eluent) and obtained as white solid

(49.2 mg, 76% yield).
^{1}H NMR (400 MHz, CD$_3$OD) δ 7.80 (brs, 1H), 7.20 – 7.09 (m, 1H), 7.06 – 6.95 (m, 1H), 5.23 (s, 1H), 4.21 (s, 1H), 1.51 (brs, 9H).
^{13}C NMR (100 MHz, CD$_3$OD) δ 172.3, 171.0, 158.4 (d, J_{FC} = 238.7 Hz), 151.3, 138.1, 128.0 (d, J_{FC} = 7.4 Hz), 114.8, 114.6, 112.0 (d, J_{FC} = 24.7 Hz), 81.2, 62.9, 49.2, 26.8.
^{19}F NMR (376 MHz, CD$_3$OD) δ -123.0.
HRMS (ESI-TOF): *m/z* Calcd. For $C_{15}H_{16}FNO_6$: $(M+Na)^+$ 348.0854. Found: 348.0851.

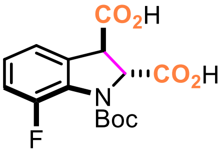

1-(*tert*-butoxycarbonyl)-7-fluoroindoline-2,3-dicarboxylic acid (7d, d.r. > 20:1): Prepared according to general procedure (3DPAFIPN (0.005 mmol, 3.2 mg, 2.5 mol%) and CsF (1.0 mmol, 151.9 mg, 5.0 equiv)) using (0.2 mmol, 47.1 mg, 1.0 equiv). After 8 hours, the product was purified by flash column chromatography (EtOAc/PE 2/1 as the eluent) and obtained as white solid (32.1 mg, 49% yield).
^{1}H NMR (400 MHz, CD$_3$OD) δ 7.24 (d, J = 5.2 Hz, 1H), 7.08 (tp, J = 8.0, 4.4 Hz, 2H), 5.37 (dd, J = 4.0, 2.4 Hz, 1H), 4.18 (s, 1H), 1.56 – 1.50 (m, 9H).
^{13}C NMR (100 MHz, CD$_3$OD) δ 173.7, 172.9, 153.9, 152.4 (d, J_{FC} = 250.6 Hz), 133.8, 129.9 (d, J_{FC} = 10.8 Hz), 126.6 (d, J_{FC} = 6.0 Hz), 122.3 (d, J_{FC} = 3.1 Hz), 118.2 (d, J_{FC} = 21.2 Hz), 83.4, 66.3, 51.8, 28.3.
^{19}F NMR (376 MHz, CD$_3$OD) δ -118.3.
HRMS (ESI-TOF): *m/z* Calcd. For $C_{15}H_{16}FNO_6$: $(M+Na)^+$ 348.0854. Found: 348.0854.

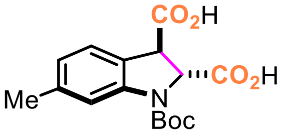

1-(tert-butoxycarbonyl)-6-methylindoline-2,3-dicarboxylic acid (7e, d.r. > 20:1): Prepared according to general procedure (3DPAFIPN (0.005 mmol, 3.2 mg, 2.5 mol%) and CsF (1.0 mmol, 151.9 mg, 5.0 equiv)) using 1-(*tert*-butyl) 6-methyl 1*H*-indole-1,6-dicarboxylate (0.2 mmol, 46.3 mg, 1.0 equiv). After 8 hours, the product was purified by flash column chromatography (EtOAc/PE 2/1 as the eluent) and obtained as white solid (42.0 mg, 65% yield).
^{1}H NMR (400 MHz, CD$_3$OD) δ 7.68 (brs, 1H), 7.26 (d, J = 7.6 Hz, 1H), 6.83 (d, J = 7.6 Hz, 1H), 5.19 (s, 1H), 4.14 – 4.07 (m, 1H), 2.33 (s, 3H), 1.52 (brs, 9H).
^{13}C NMR (100 MHz, CD$_3$OD) δ 174.3, 173.5, 153.0, 143.5, 140.4, 125.9, 125.0, 124.5, 116.2, 82.7, 64.6, 50.9, 28.5, 21.8.
HRMS (ESI-TOF): *m/z* Calcd. For $C_{17}H_{19}NO_8$: $(M+Na)^+$ 344.1105. Found: 344.1106.

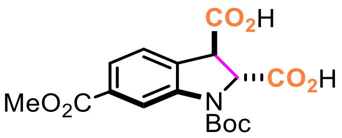

1-(*tert*-butoxycarbonyl)-6-(methoxycarbonyl)indoline-2,3-dicarboxylic acid (7f, d.r. > 20:1): Prepared according to general procedure(3DPAFIPN (0.005 mmol, 3.2 mg, 2.5 mol%) and CsF (1.6 mmol, 243.0 mg, 8.0 equiv)) using 1-(*tert*-butyl) 6-methyl 1*H*-indole-1,6-dicarboxylate (0.2 mmol, 55.1 mg, 1.0 equiv). After 8 hours, the product was purified by flash column chromatography (EtOAc/PE 2/1 as the eluent) and obtained as white solid (39.4 mg, 54% yield).

^{1}H NMR (400 MHz, CD_3OD) δ 8.35 (brs, 1H), 7.70 (q, J = 6.8 Hz, 1H), 7.51 (q, J = 6.8 Hz, 1H), 5.25 (s, 1H), 4.28 (s, 1H), 3.92 – 3.88 (s, 3H), 1.59 (brs, 9H).

^{13}C NMR (100 MHz, CD_3OD) δ 173.8, 172.5, 168.2, 152.9, 144.0, 133.2, 132.3, 126.5, 125.4, 116.2, 83.2, 64.5, 52.7, 51.2, 28.4.

HRMS (ESI-TOF): *m/z* Calcd. For $C_{17}H_{19}NO_8$: $(M+Na)^+$ 388.1003. Found:388.1002.

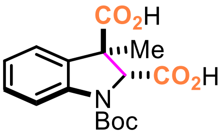

1-(*tert*-butoxycarbonyl)-3-methylindoline-2,3-dicarboxylic acid (7g, d.r. > 20:1): Prepared according to general procedure (3DPAFIPN (0.005 mmol, 3.2 mg, 2.5 mol%) and CsF (1.6 mmol, 243.0 mg, 8.0 equiv)) using *tert*-butyl 3-methyl-1*H*-indole-1-carboxylate (0.2 mmol, 46.3 mg, 1.0 equiv). After 8 hours, the product was purified by flash column chromatography (EtOAc/PE 2/1 as the eluent) and obtained as white solid (44.2 mg, 69% yield).

^{1}H NMR (400 MHz, CD_3OD) δ 7.87 – 7.43 (m, 1H), 7.32 (d, J = 7.6 Hz, 1H), 7.25 (t, J = 7.6 Hz, 1H), 7.05 – 6.96 (m, 1H), 5.37 (s, 1H), 1.61 (s, 3H), 1.52 (s, 9H).

^{13}C NMR (100 MHz, CD_3OD) δ 176.0, 172.9, 153.0, 143.2, 133.6, 130.1, 125.1, 123.9, 115.2, 82.9, 69.4, 53.6, 28.5, 20.9.

HRMS (ESI-TOF): *m/z* Calcd. For $C_{16}H_{19}NO_6$: $(M+Na)^+$ 344.1105. Found: 344.1106.

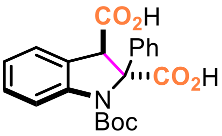

1-(*tert*-butoxycarbonyl)-2-phenylindoline-2,3-dicarboxylic acid (7f, d.r. > 20:1): Prepared according to general procedure from *tert*-butyl 2-phenyl-1*H*-indole-1-carboxylate (0.2 mmol, 58.7 mg, 1.0 equiv). After 12 hours, the product was purified by flash column chromatography (EtOAc/PE 2/1 as the eluent) and obtained as white solid (27.5 mg, 36% yield).

^{1}H NMR (400 MHz, CD_3OD) δ 7.95 (d, J = 7.6 Hz, 1H), 7.58 (dd, J = 6.4, 3.2 Hz, 2H), 7.29 (t, J = 7.6 Hz, 1H), 7.25 – 7.17 (m, 4H), 7.03 (t, J = 7.6 Hz, 1H), 4.83 (s, 1H), 1.24 (brs, 9H).

^{13}C NMR (100 MHz, CD$_3$OD) δ 174.4, 172.5, 153.4, 145.0, 138.8, 129.7, 128.9, 128.6, 128.3, 127.3, 127.2, 124.0, 115.5, 83.0, 76.6, 60.2, 28.2.
HRMS (ESI-TOF): *m/z* Calcd. For $C_{21}H_{21}NO_6$: $(M+Na)^+$ 406.1261. Found: 406.1255.

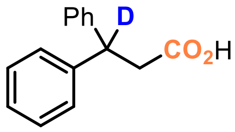

3,3-diphenylpropanoic-3-*d* acid (8a): Prepared according to general procedure using ethene-1,1-diyldibenzene (0.2 mmol, 36.0 µL, 1.0 equiv). After 89 hours, the product was purified by flash column chromatography (EtOAc/PE 1/5 as the eluent) and obtained as white solid (29.1 mg, 69% yield).
^{1}H NMR (400 MHz, CDCl$_3$) δ 7.41 – 7.09 (m, 10H), 4.51 (t, J = 8.0 Hz, 0.10H, **0.90D**), 3.07 (s, 2H).
^{13}C NMR (100 MHz, CDCl$_3$) δ 178.1, 143.3, 128.8, 127.7, 126.8, 46.7, 46.52 – 46.12 (m, CD), 40.5(3), 40.4(5).
HRMS (ESI-TOF): *m/z* Calcd. For $C_{15}H_{13}DO_4$: $(M+Na)^+$ 250.0949. Found: 250.0946.

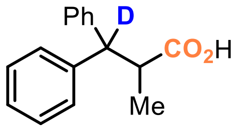

2-methyl-3,3-diphenylpropanoic-3-*d* acid (8b): Prepared according to general procedure using prop-1-ene-1,1-diyldibenzene (0.2 mmol, 38.9 mg, 1.0 equiv). After 120 hours at 50 °C, the product was purified by flash column chromatography (EtOAc/PE 1/5 as the eluent) and obtained as white solid (35.0 mg, 73% yield).
^{1}H NMR (400 MHz, CDCl$_3$) δ 7.31 – 7.09 (m, 10H), 4.05 (d, J = 11.6 Hz, 0.28H, **0.72D**), 3.40 – 3.13 (m, 1H), 1.12 (d, J = 6.8 Hz, 3H).
^{13}C NMR (100 MHz, CDCl$_3$) δ 180.4, 141.5, 141.4, 140.7, 140.6, 127.3, 127.1, 126.7(9), 126.7(6), 126.1, 125.3, 125.2, 53.2, 53.0 – 52.6 (m, CD), 42.9, 42.8, 15.8, 15.7.
HRMS (ESI-TOF): *m/z* Calcd. For $C_{16}H_{15}DO_2$: $(M-H)^-$ 240.1140. Found: 240.1152.

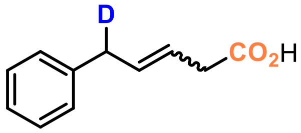

(*E*)-5-phenylpent-3-enoic-5-*d* acid (8c): Prepared according to general procedure using buta-1,3-dien-1-ylbenzene (0.2 mmol, 26.0 mg, 1.0 equiv). After 24 hours at 50 °C, the product was purified by flash column chromatography (EtOAc/PE 1/5 as the eluent) and obtained as colorless liquid (20.6 mg, 58% yield).
^{1}H NMR (400 MHz, CDCl$_3$) δ 7.32 – 7.25 (m, 2H), 7.23 – 7.15 (m, 3H), 5.83 – 5.69 (m, 1H), 5.69 – 5.50 (m, 1H), 3.40 – 3.34 (m, 1.32H, **0.68D**), 3.12 (d, J = 6.8 Hz, 2H).
^{13}C NMR (100 MHz, CDCl$_3$) δ 178.4, 140.0, 133.9, 128.6, 128.6, 126.3, 122.4, 39.0,

38.8 – 38.5 (m, CD), 37.7.
HRMS (ESI-TOF): *m/z* Calcd. For $C_{11}H_{11}DO_2$: $(M-H)^-$ 176.0827. Found: 176.0834.

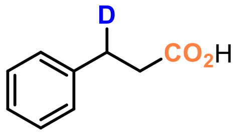

3-phenylpropanoic-3-*d* acid (8d): Prepared according to general procedure (3DPAFIPN (0.005 mmol, 3.2 mg, 2.5 mol%)) using styrene (0.2 mmol, 23.1 μL, 1.0 equiv). After 24 hours, the product was purified by flash column chromatography (EtOAc/PE 1/5 as the eluent) and obtained as yellow liquid (21.1 mg, 70% yield).
^{1}H NMR (400 MHz, CDCl$_3$) δ 7.40 – 7.10 (m, 5H), 2.95 – 2.91 (m, 1.13H, **0.87D**), 2.74 – 2.64 (m, 2H).
^{13}C NMR (100 MHz, CDCl$_3$) δ 179.4, 140.3, 128.7, 128.4, 126.5, 35.7(4), 35.6(7), 30.7, 30.6 – 30.2 (m, CD).
HRMS (ESI-TOF): *m/z* Calcd. For $C_9H_9DO_2$: $(M-H)^-$ 150.0671. Found: 150.0669.

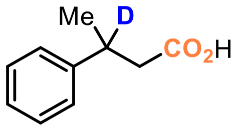

3-phenylbutanoic-3-*d* acid (8e): Prepared according to general procedure (3DPAFIPN (0.005 mmol, 3.2 mg, 2.5 mol%)) using prop-1-en-2-ylbenzene (0.2 mmol, 26.0 μL, 1.0 equiv). After 24 hours, the product was purified by flash column chromatography (EtOAc/PE 1/5 as the eluent) and obtained as yellow liquid (27.5 mg, 84% yield).
^{1}H NMR (400 MHz, CDCl$_3$) δ 7.33 – 7.27 (m, 2H), 7.25 – 7.18 (m, 3H), 3.32 – 3.20 (m, 0.12H, **0.88D**), 2.73 – 2.52 (m, 2H), 1.31 (s, 3H).
^{13}C NMR (100 MHz, CDCl$_3$) δ 178.9, 145.5, 128.7, 126.8, 126.7, 42.7, 42.6, 36.3, 36.1 – 35.7 (m, CD), 22.0, 21.9.
HRMS (ESI-TOF): *m/z* Calcd. For $C_{10}H_{11}DO_2$: $(M-H)^-$ 164.0827. Found: 164,0839.

5. The application of the reaction

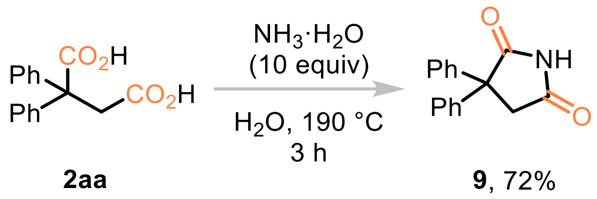

A total of 0.2 mmol of **2aa** was dissolved in 2 mL of water and 2.0 mmol of the 28% ammonia was gradually added. The mixture was heated in an oil bath at 190 °C with simultaneous distillation of water. After the water was completely removed, the reaction was maintained for 3 hours. The residue was purified by silica gel flash chromatography eluting with PE/EtOAc 5/1 afforded the desired product.

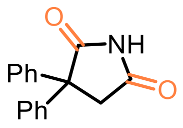

3,3-diphenylpyrrolidine-2,5-dione (9): Prepared according to general procedure from 2,2-diphenylsuccinic acid (0.2 mmol, 54.0 mg, 1.0 equiv). After 3 hours, the product was purified by flash column chromatography (eluent: EtOAc/PE 1/5) and obtained as white solid (36.3 mg, 72% yield).

^{1}H NMR (400 MHz, $CDCl_3$) δ 8.62 (s, 1H), 7.38 – 7.29 (m, 10H), 3.49 (s, 2H).

^{13}C NMR (100 MHz, $CDCl_3$) δ 179.1, 175.6, 141.4, 129.0, 127.9, 127.5, 58.5, 46.1.

HRMS (ESI-TOF): *m/z* Calcd. For $C_{16}H_{14}O_4$: $(M-H)^-$ 250.0874. Found: 250.0875.

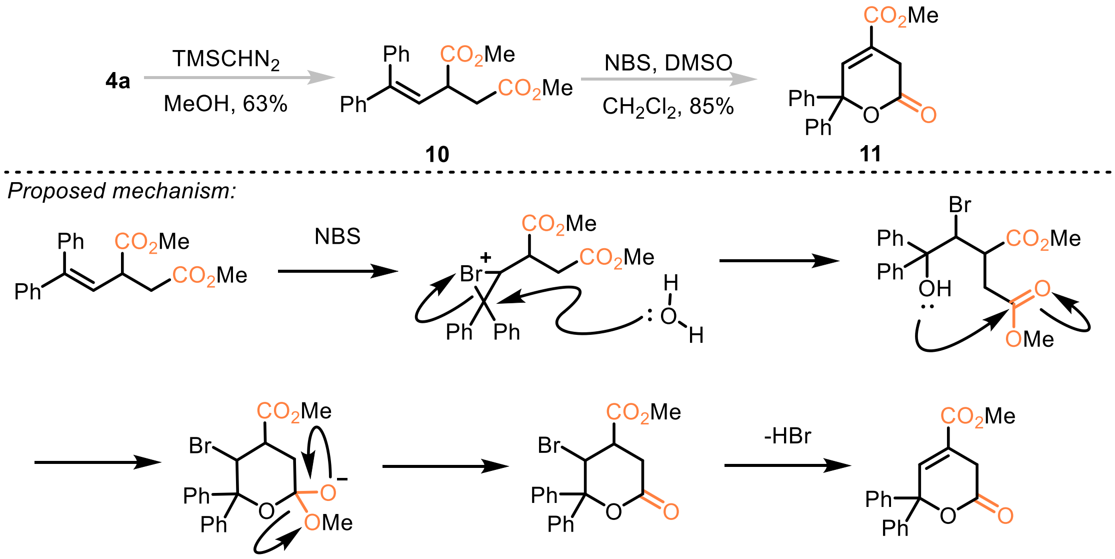

To a solution of the product **4a** (148.2 mg, 0.5 mmol) in MeOH (2 mL) was added trimethylsilyldiazomethane in hexane (6 mL, 2 M, 24.0 equiv) at 0 °C. After stirring at room temperature for 24 hours, the mixture was concentrated under high vacuum. The crude material was purified by column chromatography on silica gel (eluent: EtOAc / PE 1/10) to afford colorless oil **10** (102.6 mg, 63%).

A Schlenk tube charged with NBS (53.4 mg, 0.3 mmol, 3.0 equiv) was evacuated and backfilled with nitrogen for 3 times. DMSO (21.3 µL, 0.3 mmol, 3.0 equiv) and DCM (2 mL) were added via syringe. The reaction mixture was then stirred at room temperature for 20 mins. Compound **10** (32.4 mg, 0.1 mmol, 1.0 equiv) in DCM (1 mL) was added dropwise to the reaction mixture, which was then stirred at room temperature for 16 hours, diluted with DCM (3 x 20 mL) and brine (20 mL), dried over Na_2SO_4, filtered, concentrated under high vacuum. The residue was purified with flash chromatograph column on silica gel (eluent: EtOAc/PE 1/4) to afford brown solid **11** (26.2 mg, 85% yield).

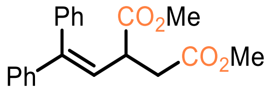

dimethyl 2-(2,2-diphenylvinyl)succinate (10)

^{1}H NMR (400 MHz, CDCl$_3$) δ 7.43 – 7.33 (m, 3H), 7.27 – 7.20 (m, 7H), 6.02 (d, J = 10.4 Hz, 1H), 3.72 (s, 3H), 3.70 – 3.63 (m, 1H), 3.61 (s, 3H), 2.84 (dd, J = 16.4, 8.4 Hz, 1H), 2.56 (dd, J = 16.4, 6.4 Hz, 1H).

^{13}C NMR (100 MHz, $CDCl_3$) δ 173.7, 171.6, 145.4, 141.6, 139.0, 129.8, 128.5, 128.3, 127.8, 127.7, 127.5, 124.3, 52.4, 51.9, 42.2, 36.9.

HRMS (ESI-TOF): *m/z* Calcd. For $C_{19}H_{18}O_4$: $(M+Na)^+$ 333.1097. Found: 333.1086.

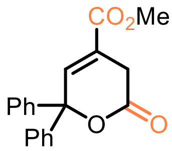

methyl 2-oxo-6,6-diphenyl-3,6-dihydro-2*H*-pyran-4-carboxylate (11)

^{1}H NMR (400 MHz, $CDCl_3$) δ 7.94 (t, J = 1.2 Hz, 1H), 7.41 – 7.29 (m, 10H), 3.75 (s, 3H), 3.43 (d, J = 1.2 Hz, 2H).

^{13}C NMR (100 MHz, $CDCl_3$) δ 172.3, 170.0, 154.5, 139.5, 128.8, 128.8, 126.8, 125.3, 90.9, 52.5, 30.3.

HRMS (ESI-TOF): *m/z* Calcd. For $C_{19}H_{16}O_4$: $(M+Na)^+$ 331.0941. Found: 331.0942.

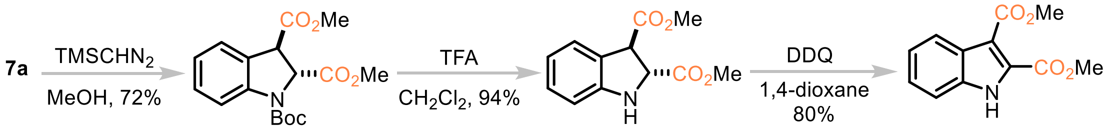

To a solution of the product **7a** (153.7 mg, 0.5 mmol) in MeOH (2 mL) was added trimethylsilyldiazomethane in hexane (3 mL, 2 M, 12.0 equiv) at 0 °C. After stirring at room temperature for 12 hours, the mixture was concentrated under high vacuum. The crude material was purified by column chromatography on silica gel (eluent: EtOAc / PE 1/5) to afford **12** (66.3 mg, 72%, d.r. > 20:1).

To a solution of compound **12** (93.9 mg, 0.28 mmol, 1.0 equiv) in CH_2Cl_2 (2 mL) was added TFA (2 mL) at 0 °C. The reaction mixture was allowed to warm to room temperature and stirred for another 5 hours. The solvent was evaporated under high vacuum. The residue was dissolved in CH_2Cl_2 (20 mL), washed successively with saturated $NaHCO_3$ (2 x 10 mL) and brine (10 mL), dried over Na_2SO_4, filtered, concentrated under high vacuum. The residue was purified with flash chromatograph column on silica gel (eluent: EtOAc/PE 1/4) to give **13** as a colorless oil (61.7 mg, 94% yield, d.r. >20:1).

To a solution of compound **13** (61.7 mg, 0.26 mmol, 1.0 equiv) in 1,4-dioxane (3

mL) was added DDQ (72.6 mg, 0.32 mmol, 1.2 equiv). The mixture was stirred at room temperature for 1 hour. The mixture was diluted with EA (20 mL), washed with saturated $NaHCO_3$ until the water phase became colorless and brine (20 mL), dried over Na_2SO_4, filtered, concentrated under high vacuum. The residue was purified with flash chromatograph column on silica gel (eluent: EtOAc/PE 1/4) to give **14** as a white solid (48.3 mg, 80% yield).

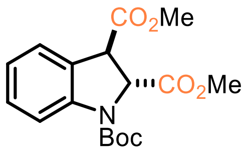

1-(*tert*-butyl) 2,3-dimethylindoline-1,2,3-tricarboxylate (12)

^{1}H NMR (400 MHz, $CDCl_3$) δ 7.92 (brs, 1H), 7.33 (d, J = 7.6 Hz, 1H), 7.27 (t, J = 7.6 Hz, 1H), 6.98 (t, J = 7.6 Hz, 1H), 5.35 (s, 1H), 4.17 (s, 1H), 3.79 (s, 3H), 3.76 (s, 3H), 1.51 (s, 9H).

^{13}C NMR (100 MHz, $CDCl_3$) δ 171.3, 170.4, 151.3, 142.3, 129.6, 125.1, 122.8, 115.02, 81.8, 62.7, 53.1, 52.7, 49.9, 28.3.

ESIHRMS: *m/z* Calcd. For $C_{17}H_{21}NO_6$: $(M+Na)^+$ 358.1261. Found: 358.1264.

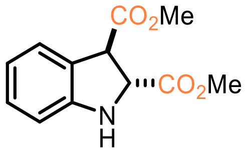

dimethylindoline-2,3-dicarboxylate (13)

^{1}H NMR (400 MHz, $CDCl_3$) δ 7.29 (d, J = 7.6 Hz, 1H), 7.11 (t, J = 7.6 Hz, 1H), 6.78 (t, J = 7.6 Hz, 1H), 6.72 (d, J = 7.6 Hz, 1H), 4.90 (d, J = 5.6 Hz, 1H), 4.47 (d, J = 5.6 Hz, 1H), 3.79 (s, 3H), 3.77 (s, 3H).

^{13}C NMR (100 MHz, $CDCl_3$) δ 173.3, 171.6, 149.5, 129.3, 125.2, 124.1, 119.7, 110.6, 62.1, 52.9, 52.8, 51.0.

ESIHRMS: *m/z* Calcd. For $C_{12}H_{13}NO_4$: $(M+Na)^+$ 258.0737. Found: 258.0739.

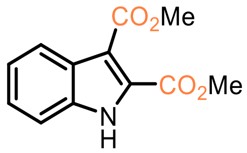

dimethyl 1*H*-indole-2,3-dicarboxylate (14)

^{1}H NMR (400 MHz, CDCl$_3$) δ 9.83 (s, 1H), 8.05 (d, J = 8.0 Hz, 1H), 7.45 (d, J = 8.4 Hz, 1H), 7.35 (ddd, J = 8.4, 7.2, 1.2 Hz, 1H), 7.26 (ddd, J = 8.0, 7.2, 1.2 Hz, 1H), 3.99 (s, 3H), 3.94 (s, 3H).

^{13}C NMR (100 MHz, CDCl$_3$) δ 164.9, 161.7, 135.0, 128.3, 126.8, 125.9, 122.7(3), 122.6(5), 112.2, 111.8, 52.8, 52.0.

ESIHRMS: *m/z* Calcd. For $C_{12}H_{11}NO_4$: $(M+Na)^+$ 256.0580. Found: 256.0583.

6.1 TEMPO trapping

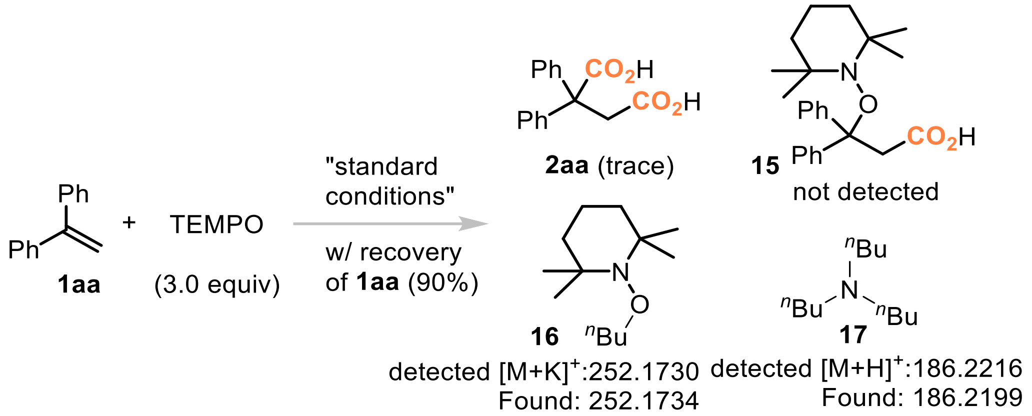

An oven-dried tube (2 mL) containing a stirring bar was charged with TEMPO (93.8 mg, 0.6 mmol, 3 equiv.), The Schlenk tube was then introduced in a glovebox, where it was charged with $(n\mathrm{Bu}_4\mathrm{N})_2\mathrm{C}_2\mathrm{O}_4$ (229.2 mg, 0.4 mmol, 2.0 equiv). The tube was taken out of the glovebox and connected to a vacuum line where it was evacuated and back-filled with N_2 for 3 times. Then the substrate **1aa** (36.0 µL, 0.2 mmol) and DMF (1.5 mL) were added. Finally, the Schlenk tube was placed at a distance of 2 cm from two 45 W blue LEDs lamps (wavelength: 450 nm) and stirred at room temperature for 24 hours. After the completion of the reaction, the mixture was quenched with 4 mL of HCl (1 N), diluted with water (30 mL) and extracted with EtOAc (3 x 30 mL). The combined organic layers were washed with water and brine before dried over sodium sulfate and concentrated under reduced pressure. The crude ^{1}H NMR of the material obtained showed only trace amount of desired product and 90% of starting material was recovered.

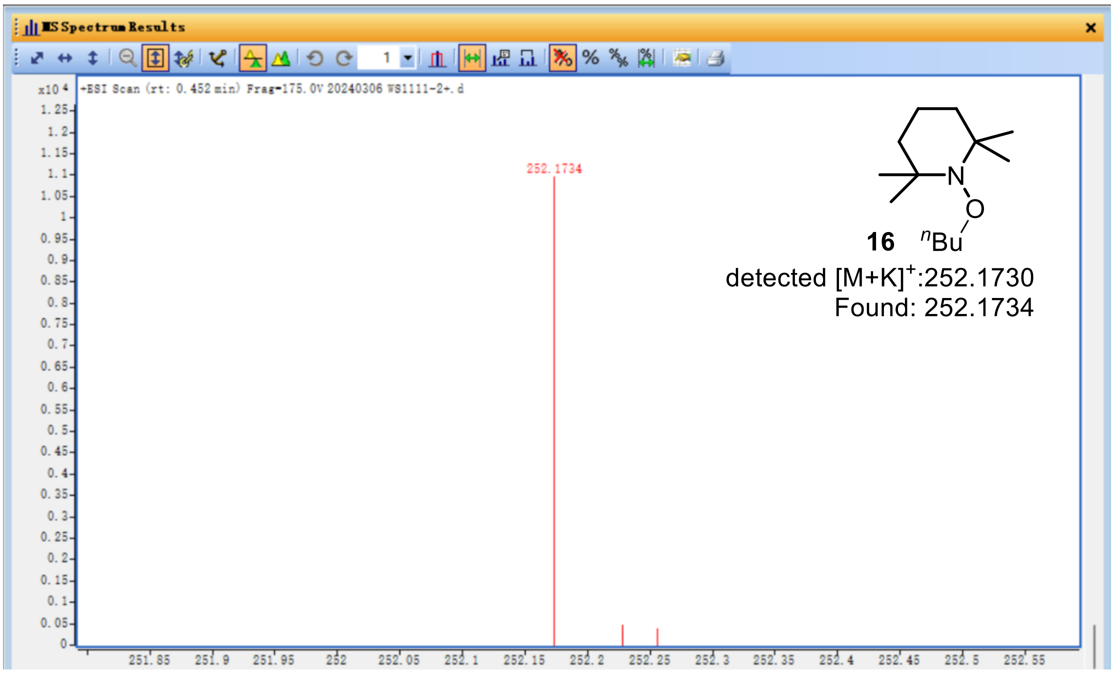

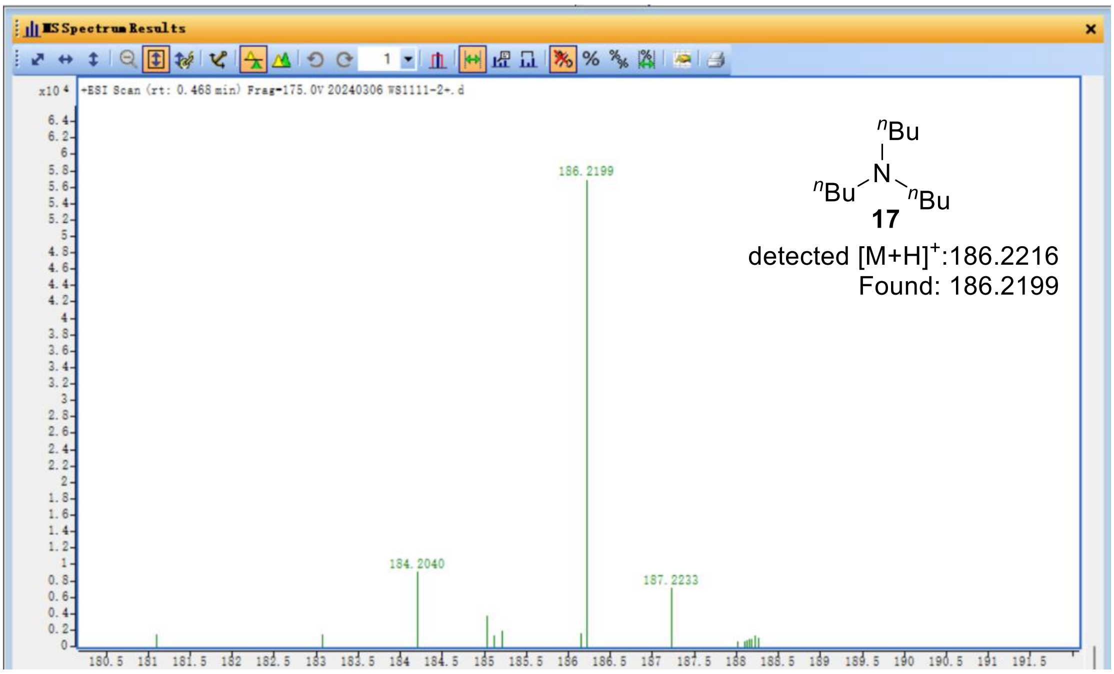

6.2 D-labeling experiment

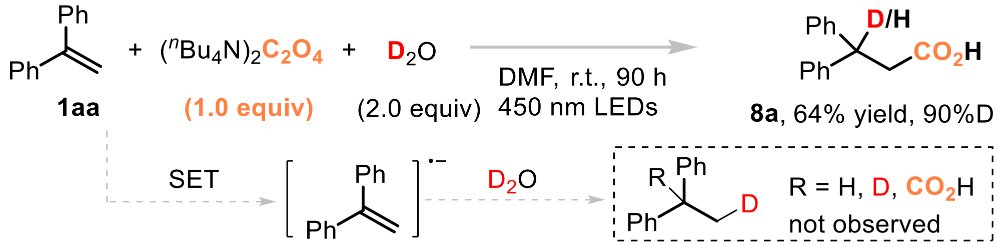

An oven-dried tube (2 mL) was introduced in a glovebox, where it was charged with $(n\mathrm{Bu_4N})_2\mathrm{C_2O_4}$ (114.6 mg, 0.2 mmol, 1.0 equiv). The tube was taken out of the glovebox and connected to a Schlenk line where it was evacuated and back-filled with N_2 for 3 times. Then the substrate **1aa** (36.0 µL, 0.2 mmol), D_2O (7.2 µL, 0.4 mmol, 2.0 equiv) and DMF (1.5 mL) were added under N_2 atmosphere. Finally, the Schlenk tube was placed at the distance of 2 cm from two 45 W blue LEDs lamps (wavelength: 450 nm) and stirred at room temperature for 24 hours. After the completion of the reaction, the mixture was quenched with 4 mL of HCl (1 N), diluted with water (30 mL), and extracted with EtOAc (3 x 30 mL). The combined organic layers were washed with water and brine before dried over anhydrous Na_2SO_4 and concentrated under reduced pressure. The yield of product **8a** was determined by crude ^{1}H NMR based on the 1,2-dichloroethane as the internal standard.

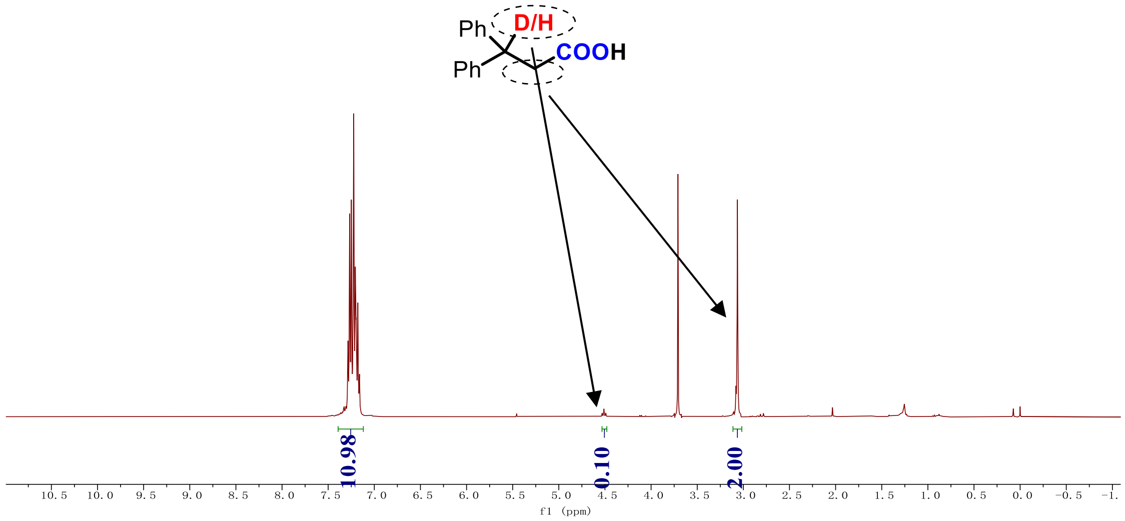

6.3 Biaryl ethane with 1.0 equiv of TBAO

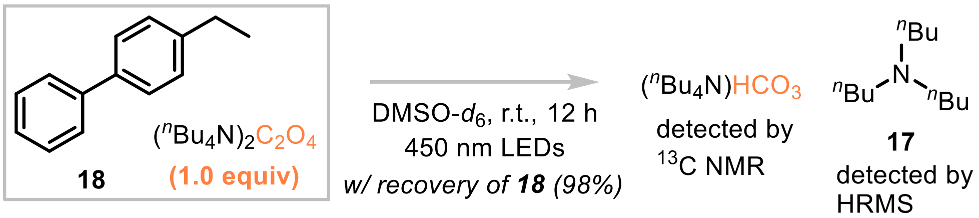

The Schlenk tube charged with **18** (18.2 mg, 0.1 mmol) was introduced in a glovebox, where it was charged with $(n\mathrm{Bu_4N})_2C_2O_4$ (229.2 mg, 0.4 mmol, 4.0 equiv). The tube was taken out of the glovebox and connected to a Schlenk line where it was evacuated and back-filled with N_2 for 3 times. Then DMSO-d_6 (2.5 mL) were added under nitrogen protection. Finally, the Schlenk tube was placed at the distance of 2 cm from two 45 W blue LEDs lamps (wavelength: 450 nm) and stirred at room temperature for 12 hours. ^{13}C NMR was tested at 0, 6, and 12 hours respectively, with the syringe to take 0.5 mL mixture.

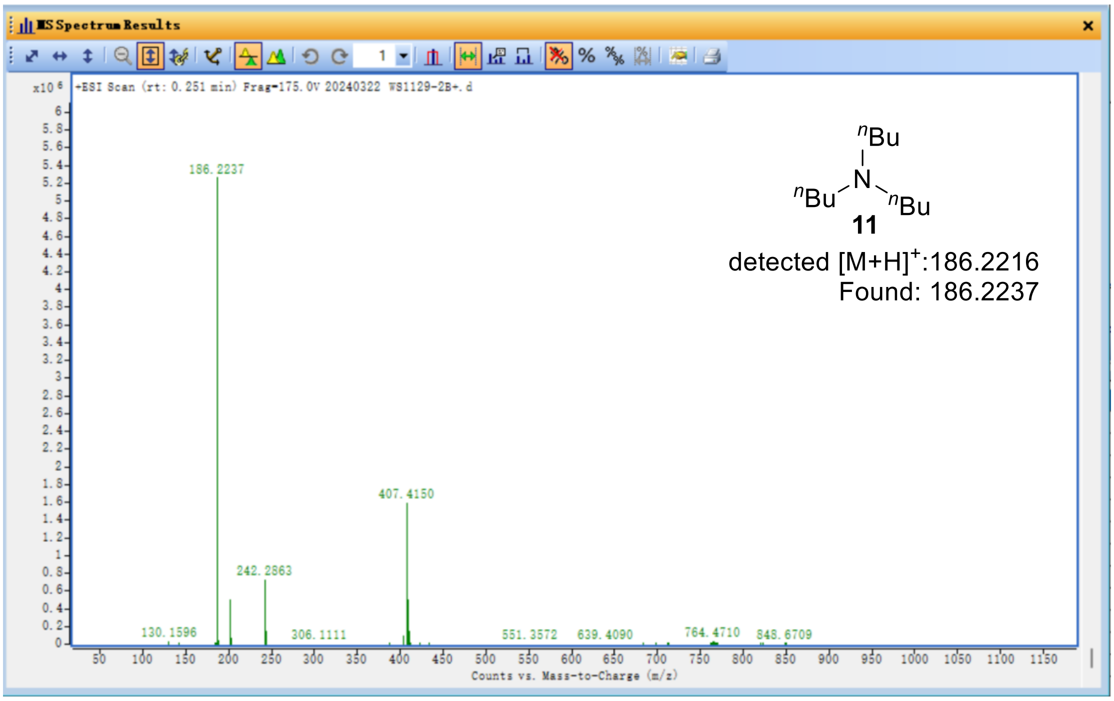
MS Spectrum Results
+ESI Scan (rt: 0.251 min) Frag=175.0V 20240322 WS1129-2B+.d
186.2237
407.4150
242.2863
130.1596
306.1111
551.3572
639.4090
764.4710
848.6709
Counts vs. Mass-to-Charge (m/z)
ⁿBu
N
ⁿBu
ⁿBu
11
detected [M+H]+:186.2216
Found: 186.2237

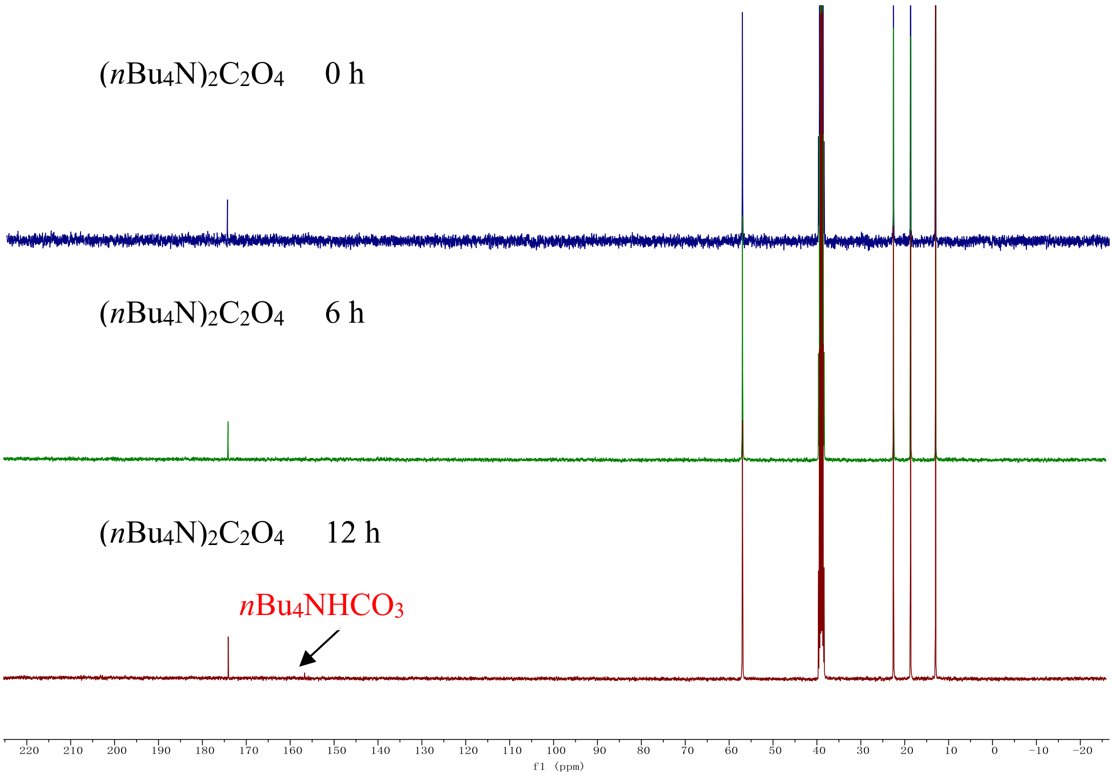
(nBu4N)2C2O4 0 h
(nBu4N)2C2O4 6 h
(nBu4N)2C2O4 12 h
nBu4NHCO3
f1 (ppm)

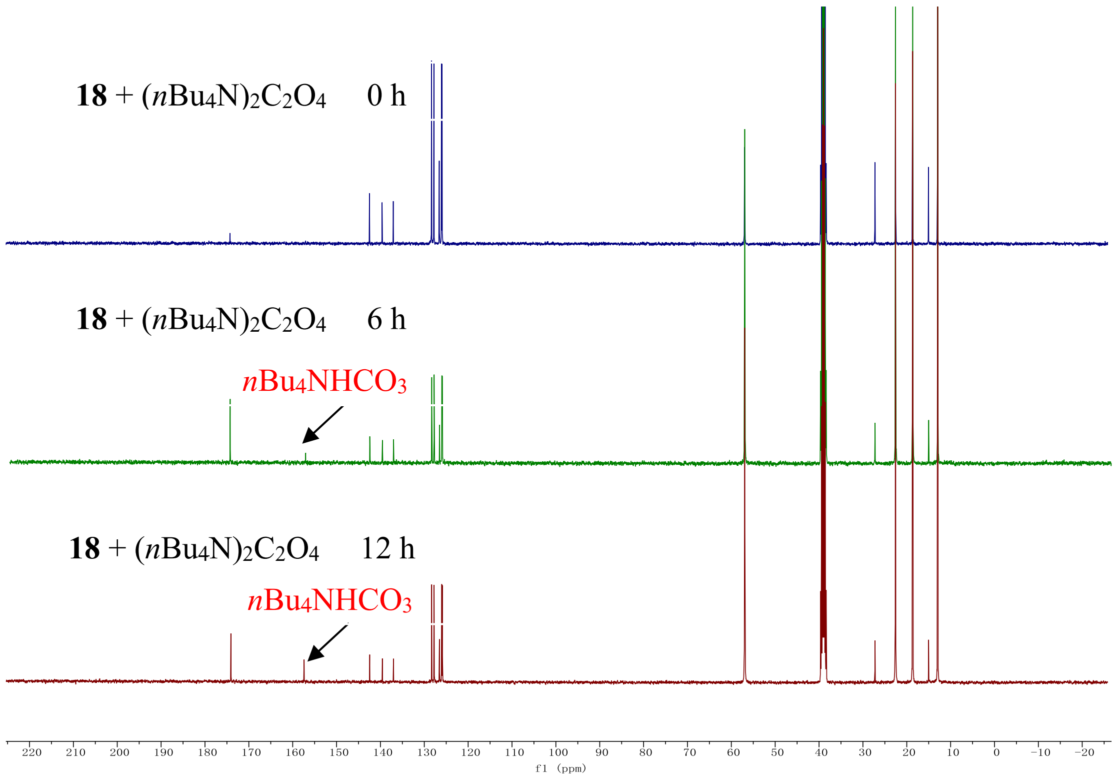

6.4 UV-Vis absorption experiment

The solutions of the following components were prepared, respectively. A pale-yellow solution was observed when the starting material **3a** was combined with oxalate in dry DMF.

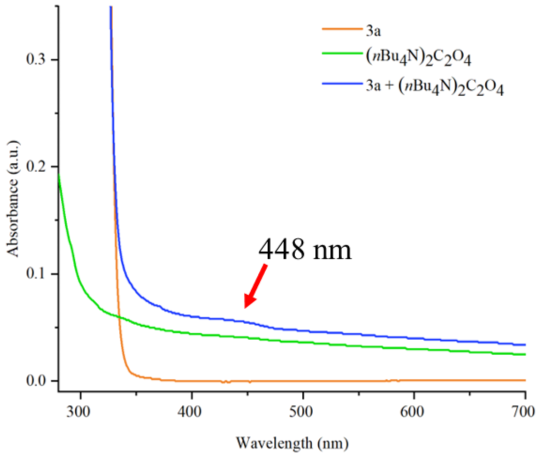

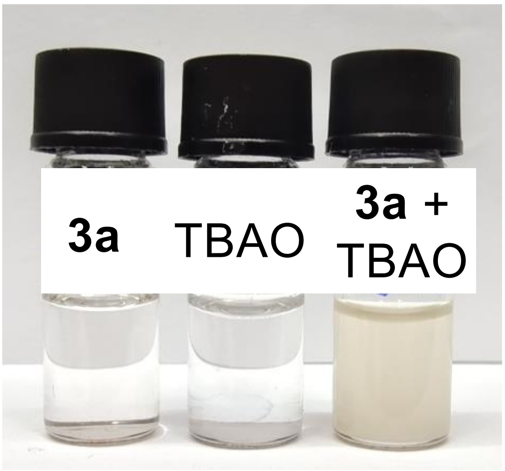

7. References

[1] P. Xu, S. Wang, H. Xu, Y.-Q. Liu, R.-B. Li, W.-W. Liu, X.-Y. Wang, M.-L. Zou, Y. Zhou, D. Guo, X. Zhu, *ACS Catal.* **2023**, *13*, 2149-2155.

[2] S. Ito, H. Fujimoto, M. Tobisu, *J. Am. Chem. Soc.* **2022**, *144*, 15, 6714-6718.

[3] H. Cui, Y. Li, S.-L. Zhang, *Org. Biomol. Chem.* **2012**, *10,* 2862-2869.

[4] V. C. Purohit, S. P. Allwein, R. P. Bakale, *Org. Lett.* **2013***, 15,* 1650-1653.

[5] S. K. Pagire, N. Kumagai, M. Shibasaki, *Org. Lett.* **2020**, *22*, 7853-7858.

[6] S. Xia, D. Cao, H. Zeng, L.-N. He, C.-J. Li, *JACS Au* **2022**, *2,* 1929-1934.

[7] X. Dan, Q. Yang, L. Xing, Y. Tang, W. Wang, Y. Cai, *Org. Let*. **2023**, *25*, 4124-4129.

[8] J.-M. Huang, Z.-Q. Lin, D.-S. Chen, *Org. Lett.* **2012**, *14*, 22-25.

[9] L.-F. Yang, Z.-Q. Xiong, X.-H. Ouyang, Q.-A. Wang, J.-H. Li, *Org. Lett.* **2024**, *26*, 1667-1671.

[10] J. Wang, B. Shao, H. Ge, Y. Li, H. Qi, L. Xiao, *Org. Lett.* **2023**, *25*, 5333-5338.

[11] H. Liu, M. Xu, C. Cai, J. Chen, Y. Gu, Y. Xia, *Org. Lett.* **2020**, *22*, 1193-1198.

[12] G.-Y. Tan, F. Paulus, Á. Rentería-Gómez, R. F. Lalisse, C. G. Daniliuc, O. Gutierrez, F. Glorius, *J. Am. Chem. Soc.* **2022**, *144*, 21664-21673.

[13] M. Yilmaz, A. U. Inal, *Tetrahedron* **2022**, *116*, 132806.

[14] L. Zhou, F. Ye, Y. Zhang, J.-B. Wang, *Org. Lett.* **2012**, *14*, 922-925.

[15] X.-K. Zhou, Y. Xu, G.-B. Dong, *J. Am. Chem. Soc.* **2021**, *143*, 20042-20048.

[16] A. M. Sheta, M. A. Mashaly, S. B. Said, S. S. Elmorsy, A.V. Malkov, B. R. Buckley, *Chem. Sci.* **2020**, *11*, 9109-9114.

[17] A. Bhowmik, R. A. Fernandes, *Org. Lett.* **2019**, *21*, 9203-9207.

[18] L. Yang, X. Wu, W. Lu, Y. Lu, Z. Zhang, *Org. Lett.* **2024**, *26*, 2287-2291.

[19] G. L. Trammel, R. Kuniyil, P. F. Crook, P. Liu, M. K. Brown, *J. Am. Chem. Soc.* **2021**, *143*, 16502-16511.

[20] S. Pandit, V. K. Pandey, A. S. Adhikari, S. Kumar, A. Maurya, K. R. Kant, N. Majumdar, *J. Org. Chem.* **2023**, *88*, 97-105.

[21] Y. Yi, Z. Fan, C. Xi, *Green Chem.* **2022**, *24*, 7894-7899.

[22] Y. You, W. Kanna, H. Takano, H. Hayashi, S. Maeda, T. Mita, *J. Am. Chem. Soc.* **2022**, *144*, 3685-3695.

8. NMR spectra

1-(4-vinylphenyl)-1*H*-pyrrole (1n)

^{1}H NMR (400 MHz, $CDCl_3$)

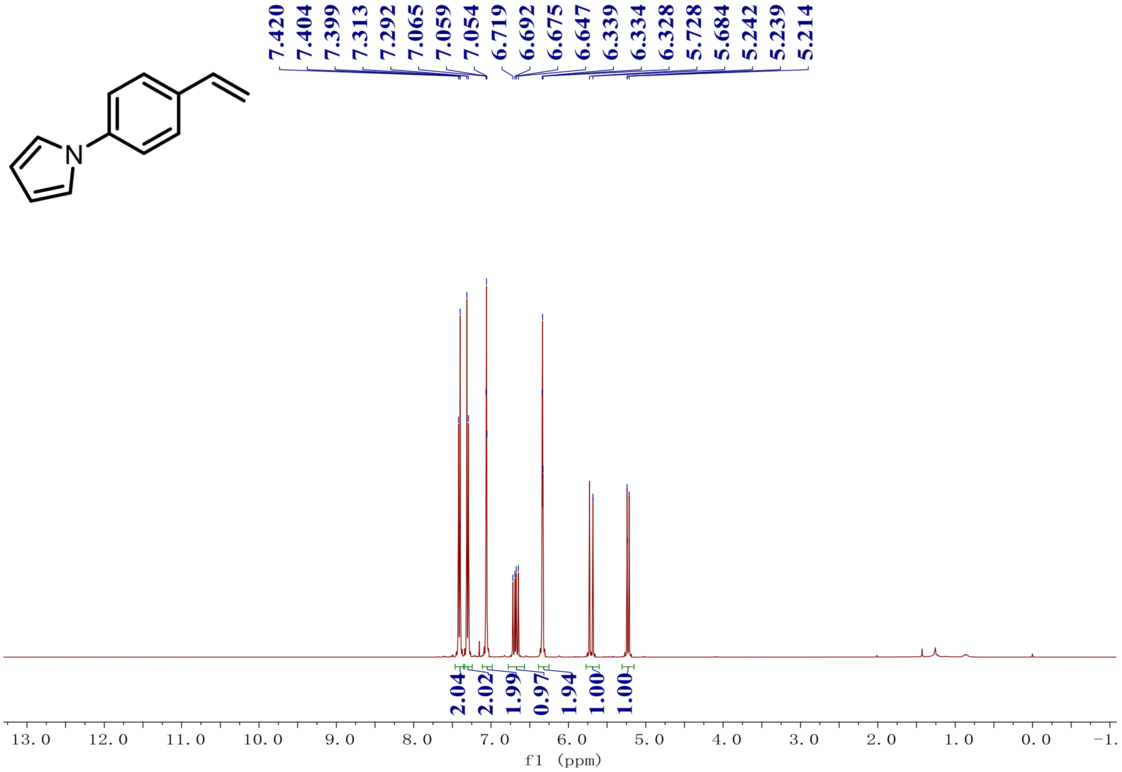

1-(4-vinylphenyl)-1*H*-pyrrole (1n)

^{13}C NMR (100 MHz, $CDCl_3$)

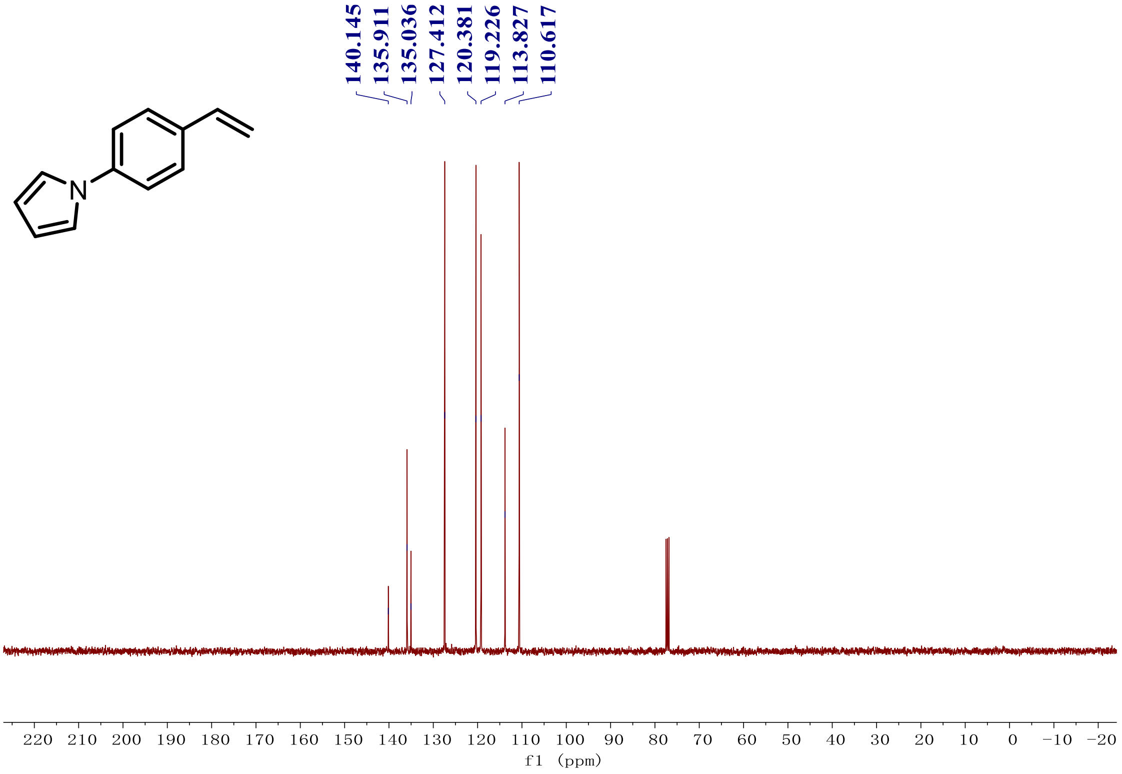

4'-methoxy-2-vinyl-1,1'-biphenyl (1o)
^{1}H NMR (400 MHz, $CDCl_3$)

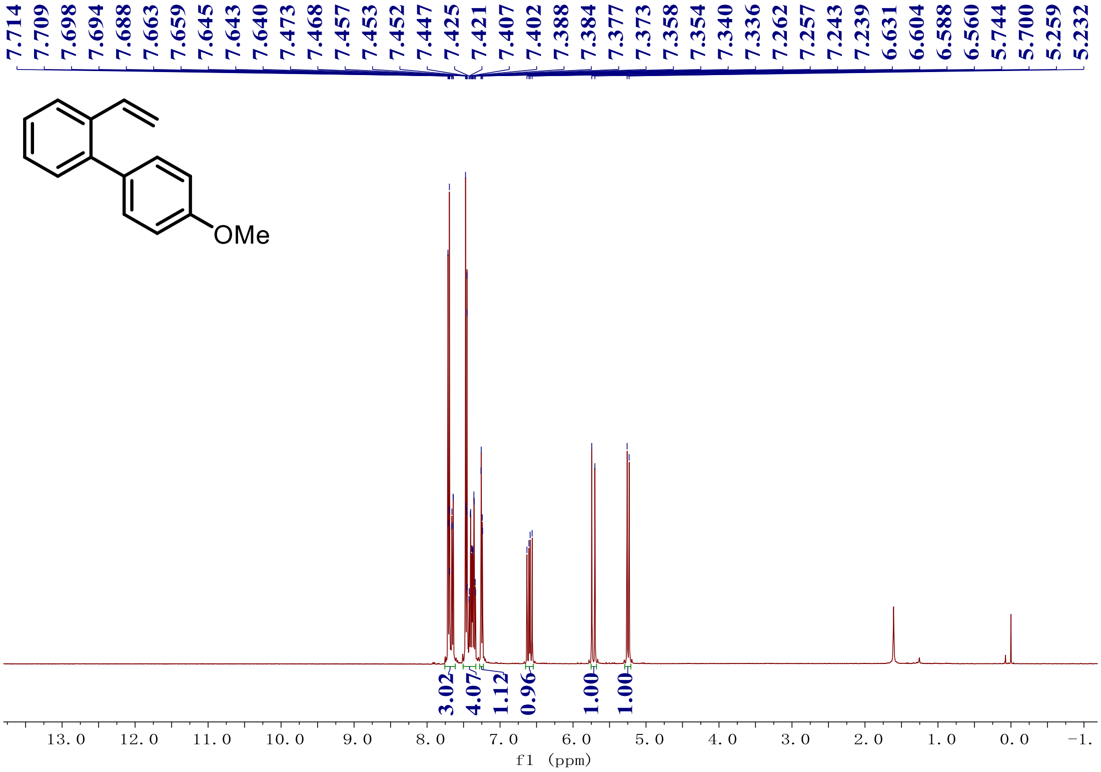

4'-methoxy-2-vinyl-1,1'-biphenyl (1o)
^{13}C NMR (100 MHz, $CDCl_3$)

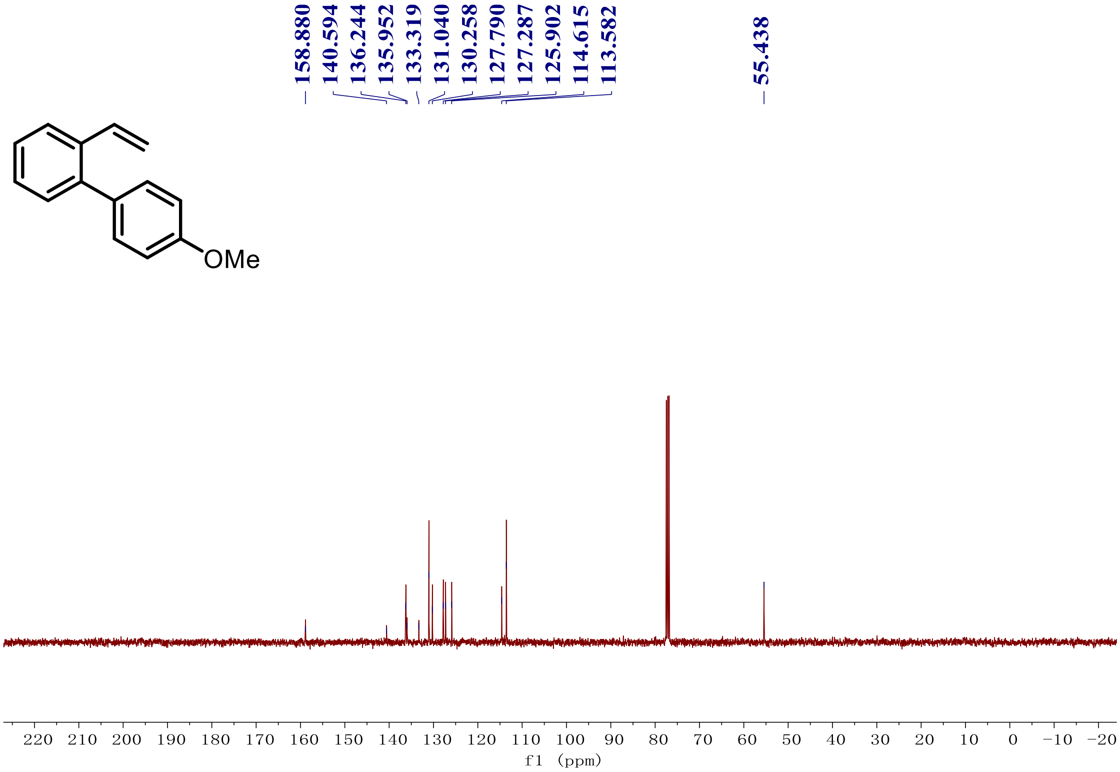

5-methoxy-2-vinyl-1,1'-biphenyl (1p)
^{1}H NMR (400 MHz, $CDCl_3$)

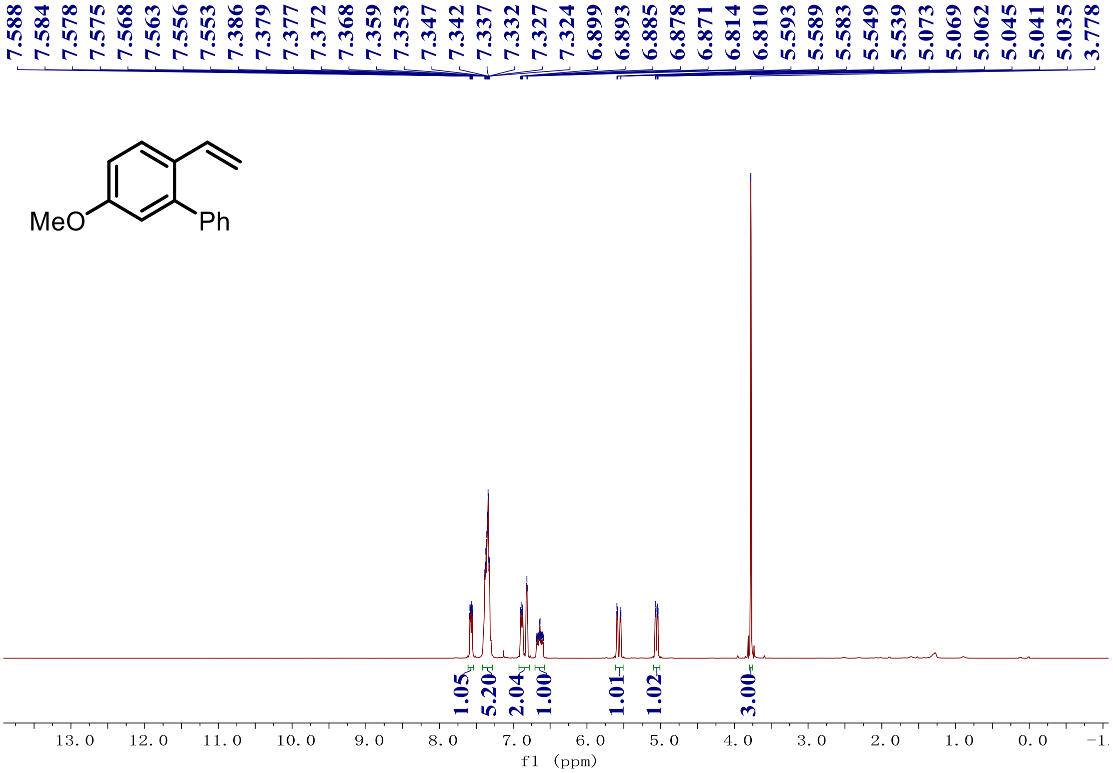

5-methoxy-2-vinyl-1,1'-biphenyl (1p)
^{13}C NMR (100 MHz, $CDCl_3$)

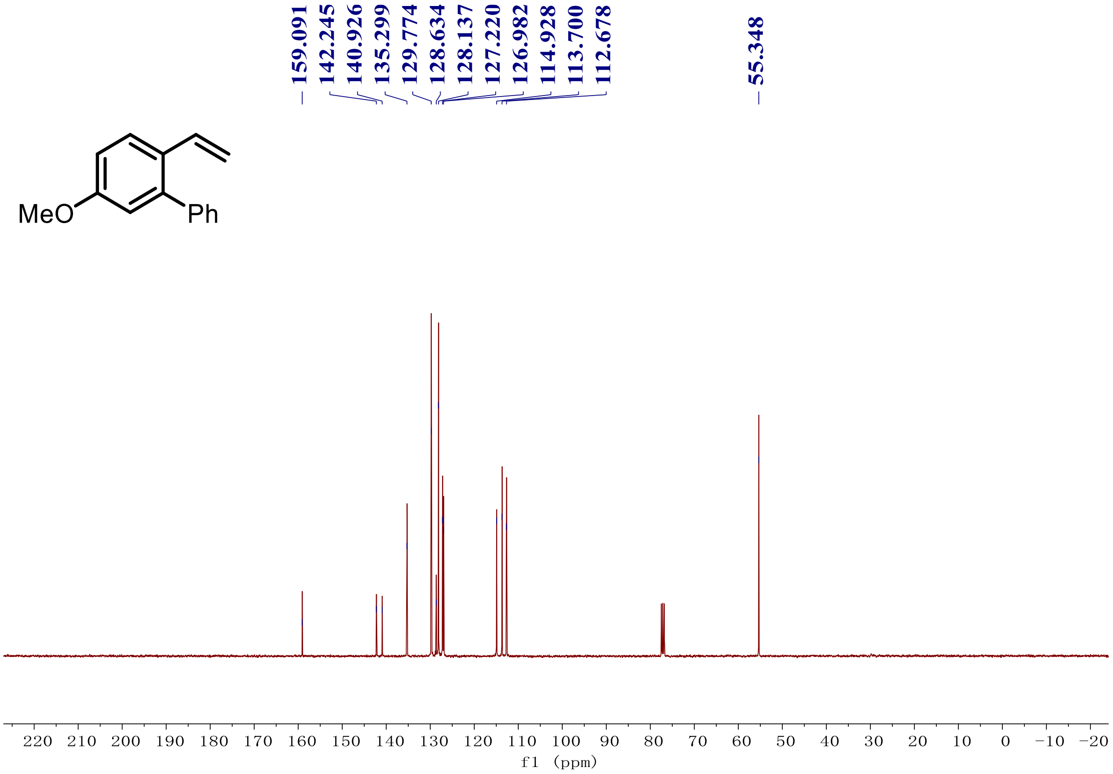

2'-vinyl-[1,1'-biphenyl]-4-carbonitrile (1q)
^{1}H NMR (400 MHz, $CDCl_3$)

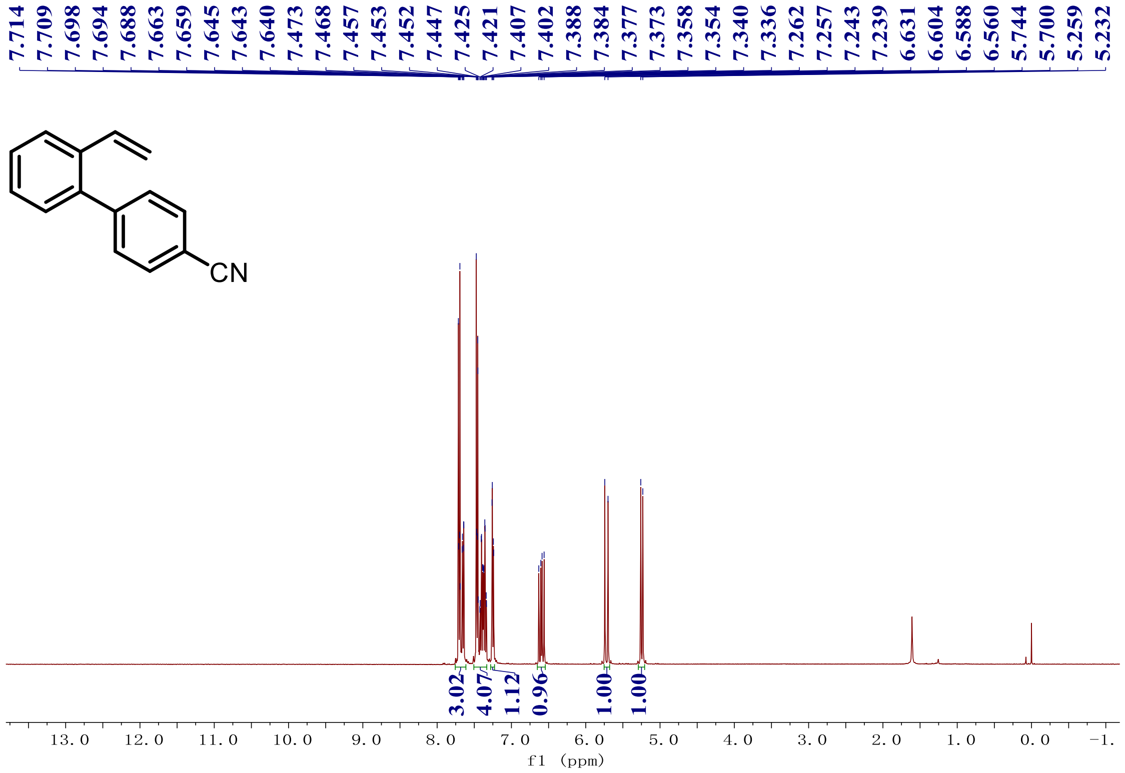

2'-vinyl-[1,1'-biphenyl]-4-carbonitrile (1q)
^{13}C NMR (100 MHz, $CDCl_3$)

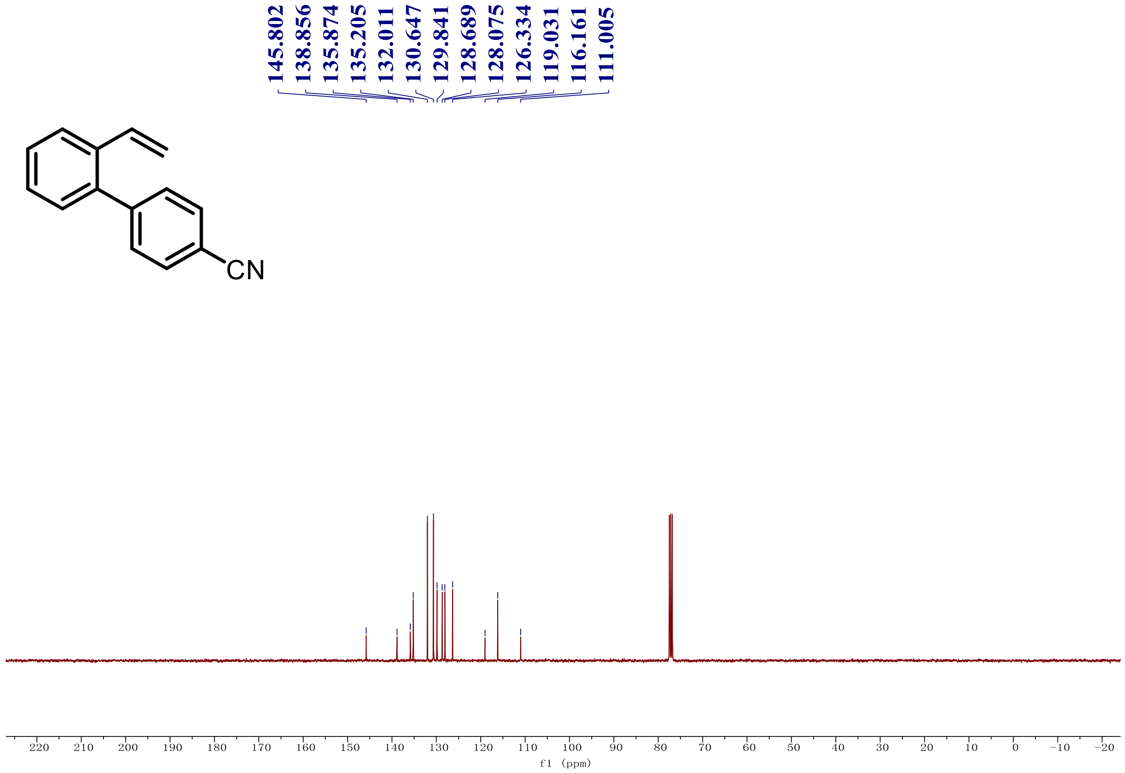

methyl 4'-vinyl-[1,1'-biphenyl]-4-carboxylate (1r)
^{1}H NMR (400 MHz, CDCl$_3$)

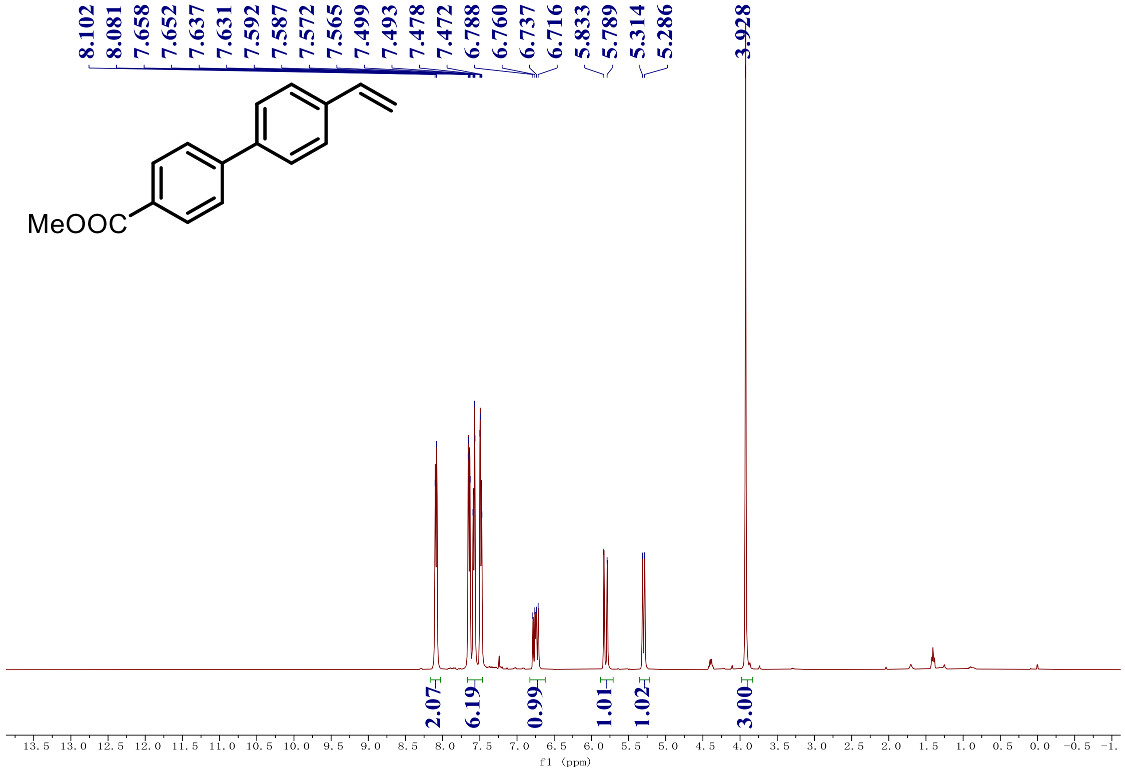

methyl 4'-vinyl-[1,1'-biphenyl]-4-carboxylate (1r)
^{13}C NMR (100 MHz, CDCl$_3$)

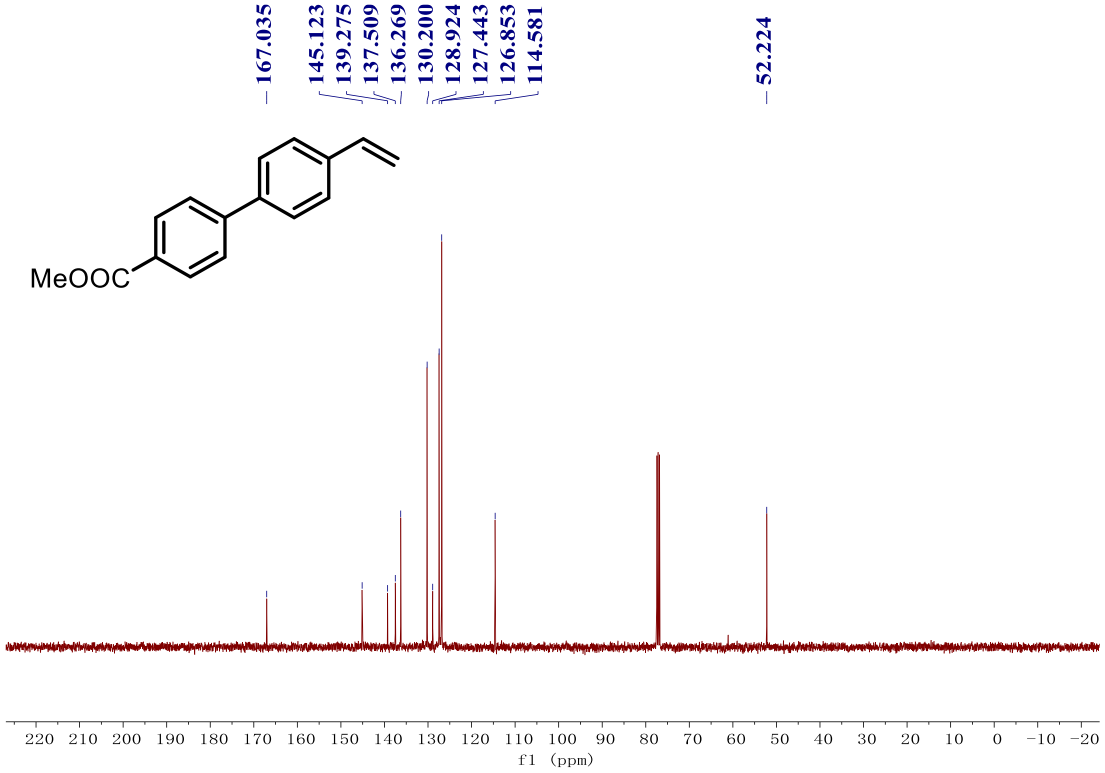

3'-fluoro-2-vinyl-1,1'-biphenyl (1s)

^{1}H NMR (400 MHz, $CDCl_3$)

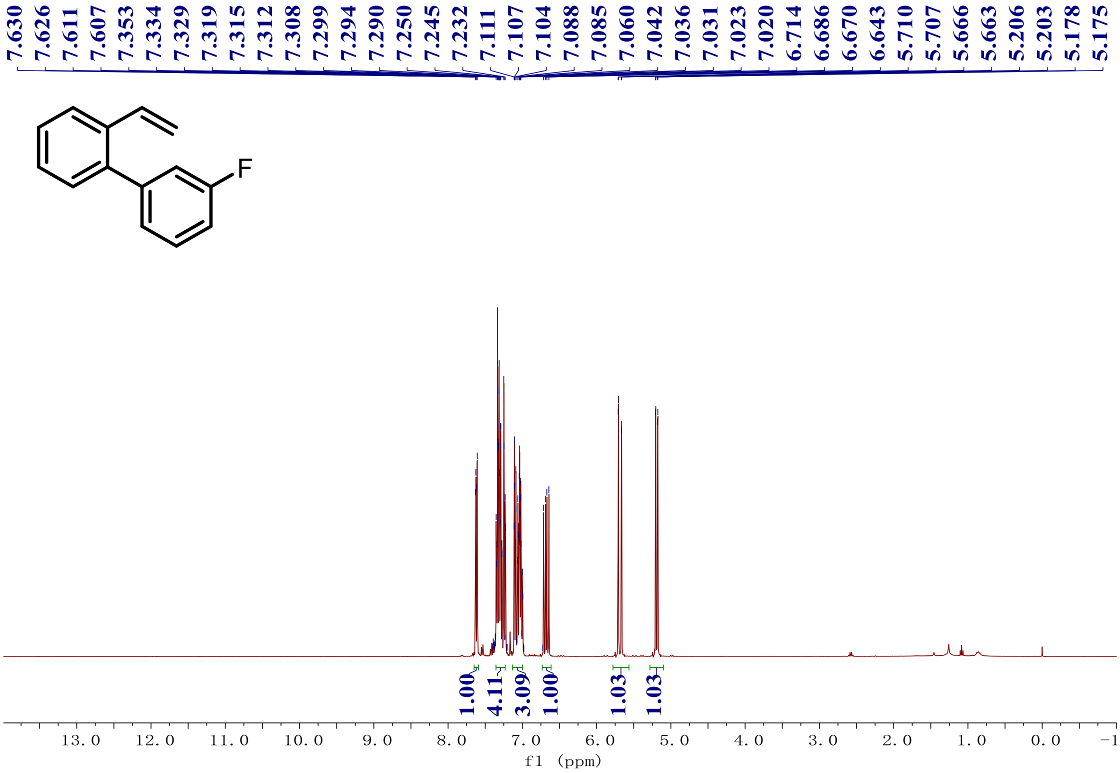

3'-fluoro-2-vinyl-1,1'-biphenyl (1s)

^{13}C NMR (100 MHz, $CDCl_3$)

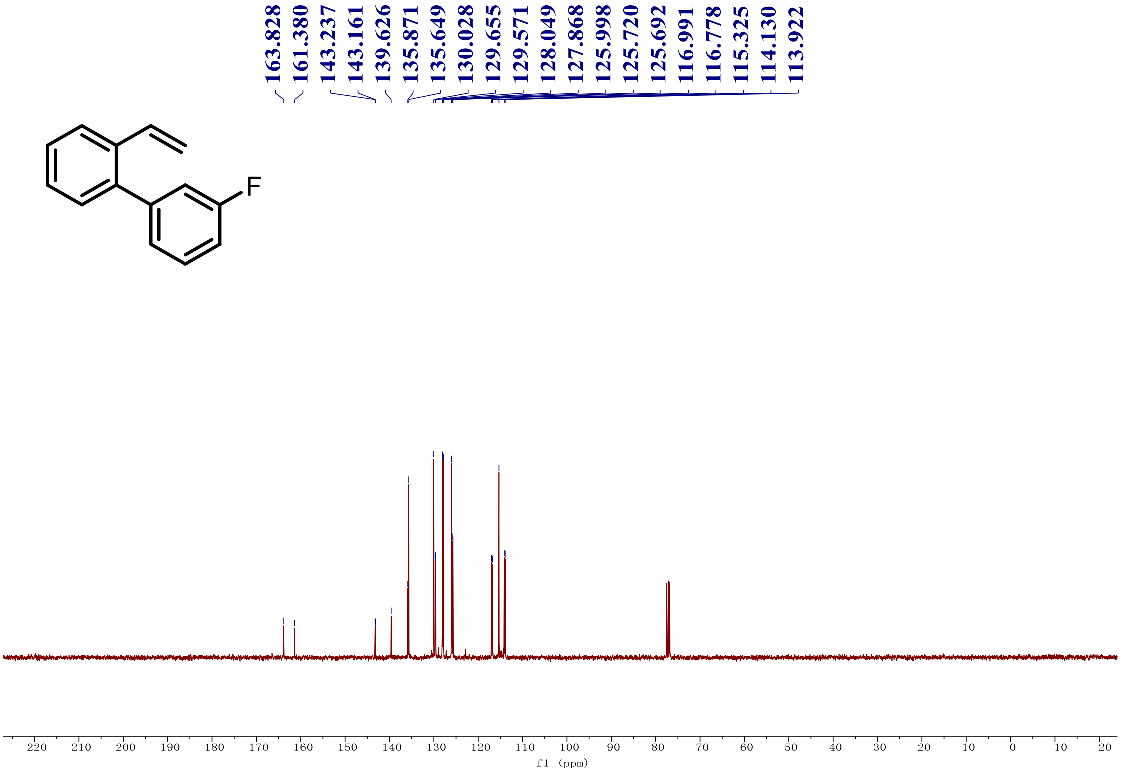

3'-fluoro-2-vinyl-1,1'-biphenyl (1s)
^{19}F NMR (376 MHz, $CDCl_3$)

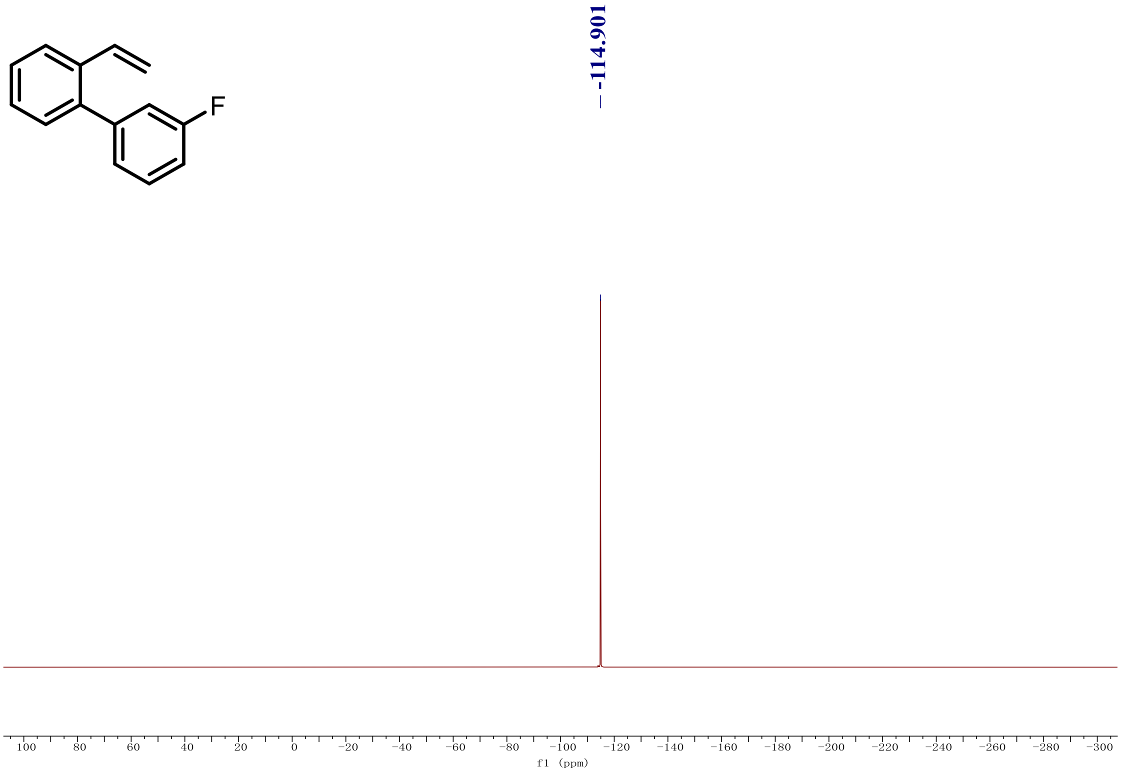

trimethyl(2'-vinyl-[1,1'-biphenyl]-4-yl)silane (1t)
^{1}H NMR (400 MHz, $CDCl_3$)

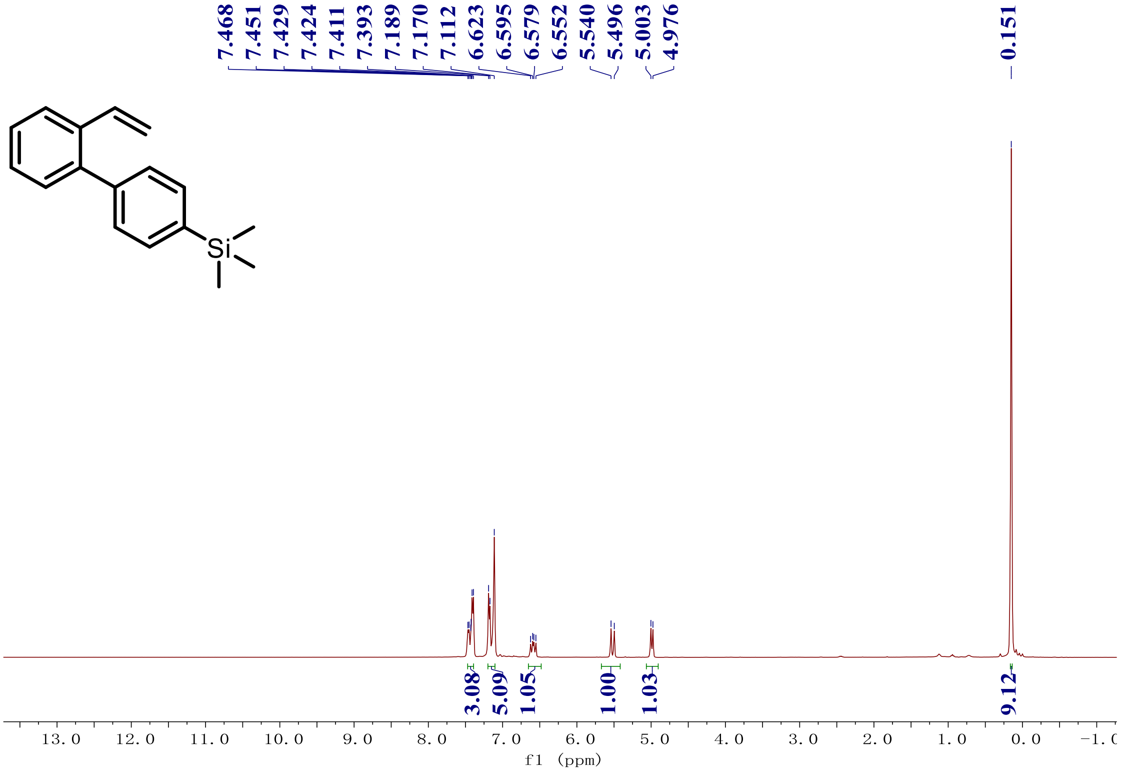

trimethyl(2'-vinyl-[1,1'-biphenyl]-4-yl)silane (1t)
^{13}C NMR (100 MHz, $CDCl_3$)

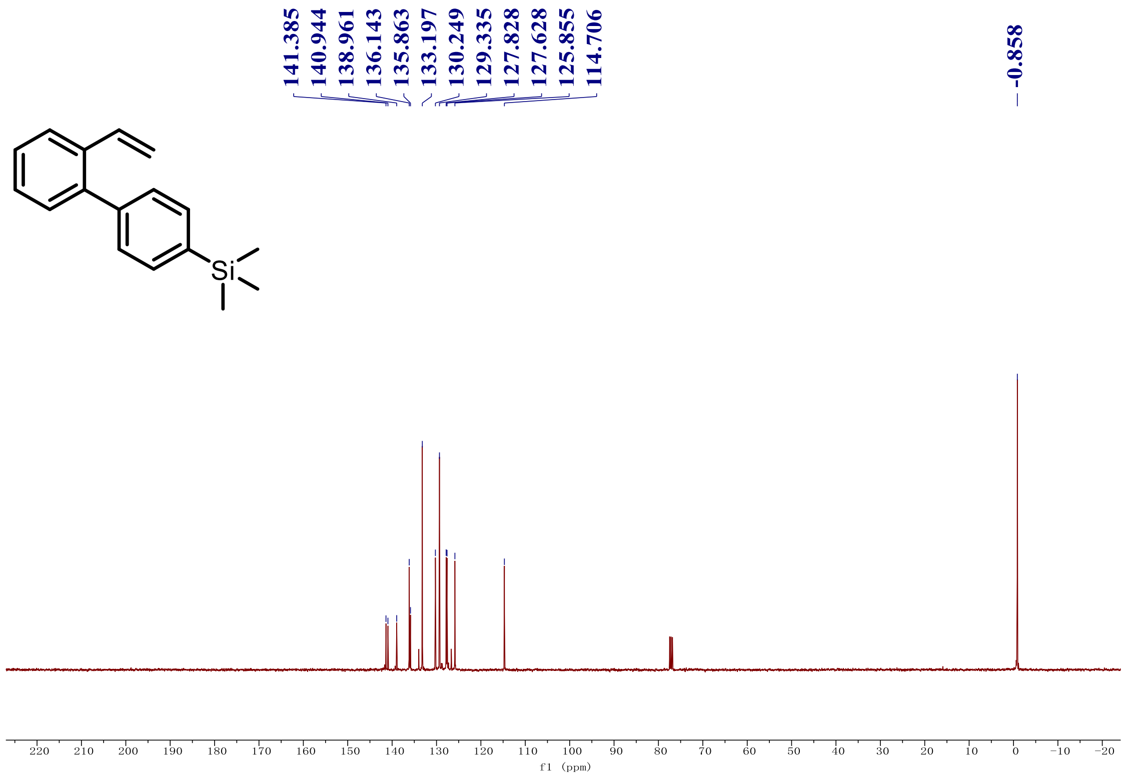

4-(1-phenylvinyl)dibenzo[*b*,*d*]furan (1ag)
^{1}H NMR (400 MHz, $CDCl_3$)

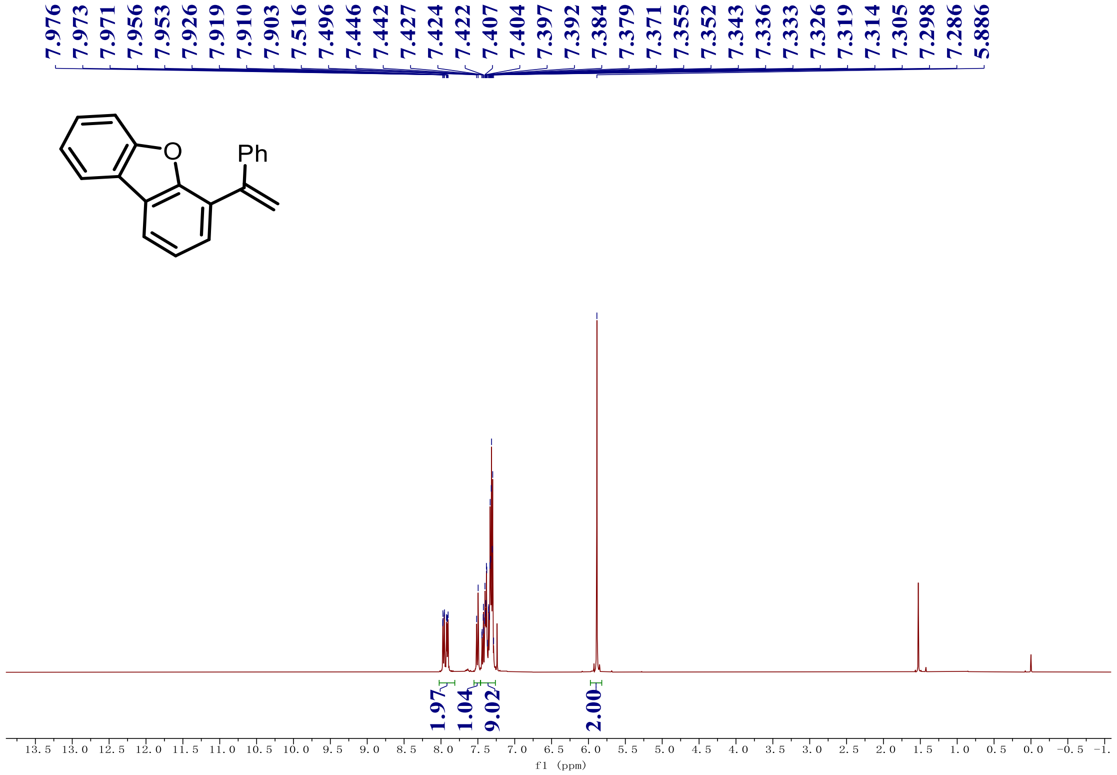

4-(1-phenylvinyl)dibenzo[*b*,*d*]furan (1ag)
^{13}C NMR (100 MHz, CDCl$_3$)

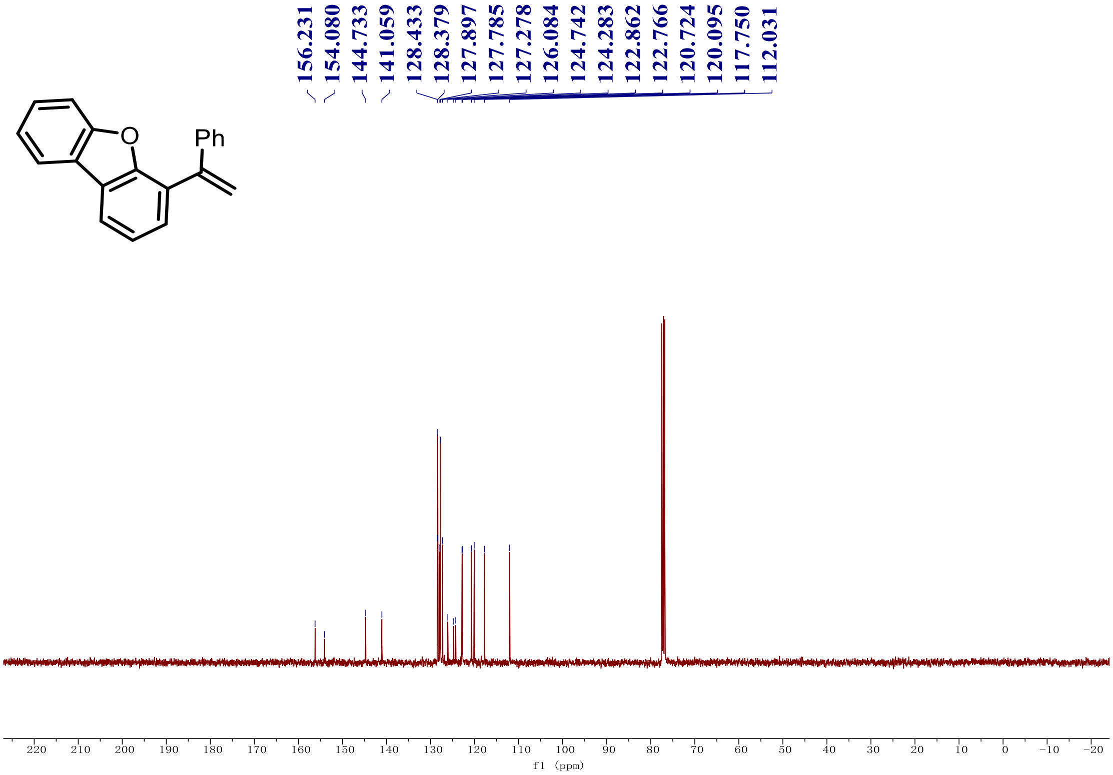

(1*S*,2*R*,5*S*)-2-isopropyl-5-methylcyclohexyl 4-(1-phenylvinyl)benzoate (1cd)
^{1}H NMR (400 MHz, CDCl$_3$)

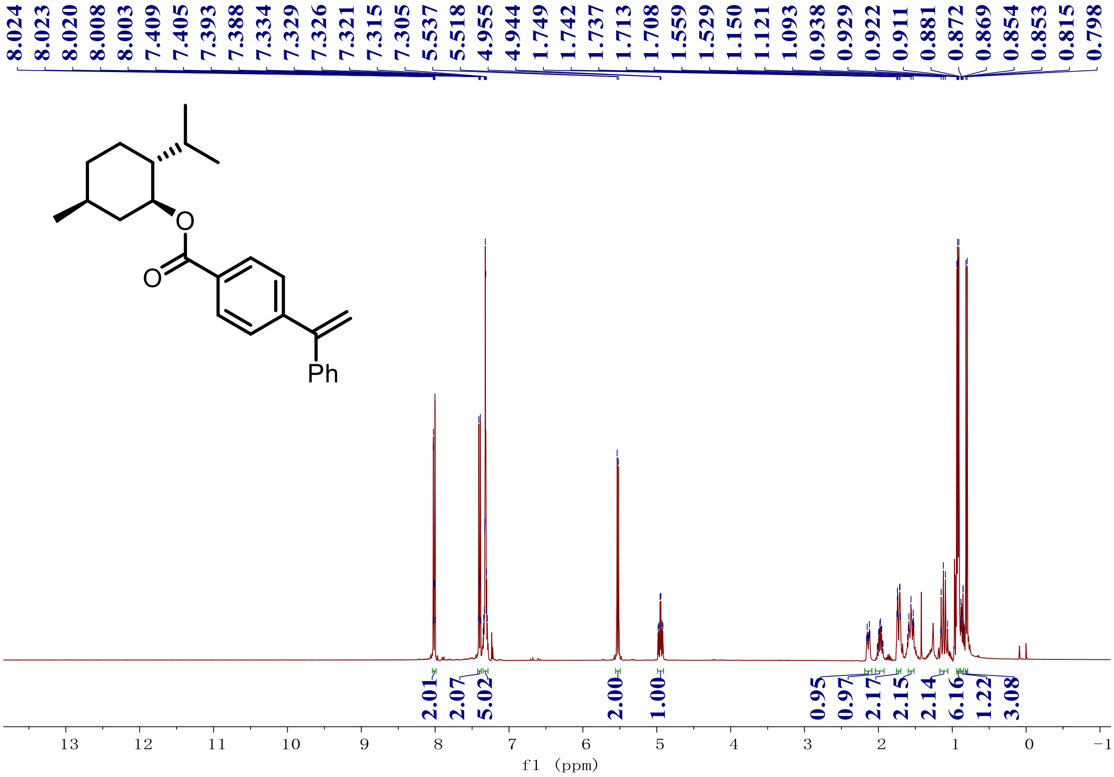

(1*S*,2*R*,5*S*)-2-isopropyl-5-methylcyclohexyl 4-(1-phenylvinyl)benzoate (1cd)
^{13}C NMR (100 MHz, CDCl$_3$)

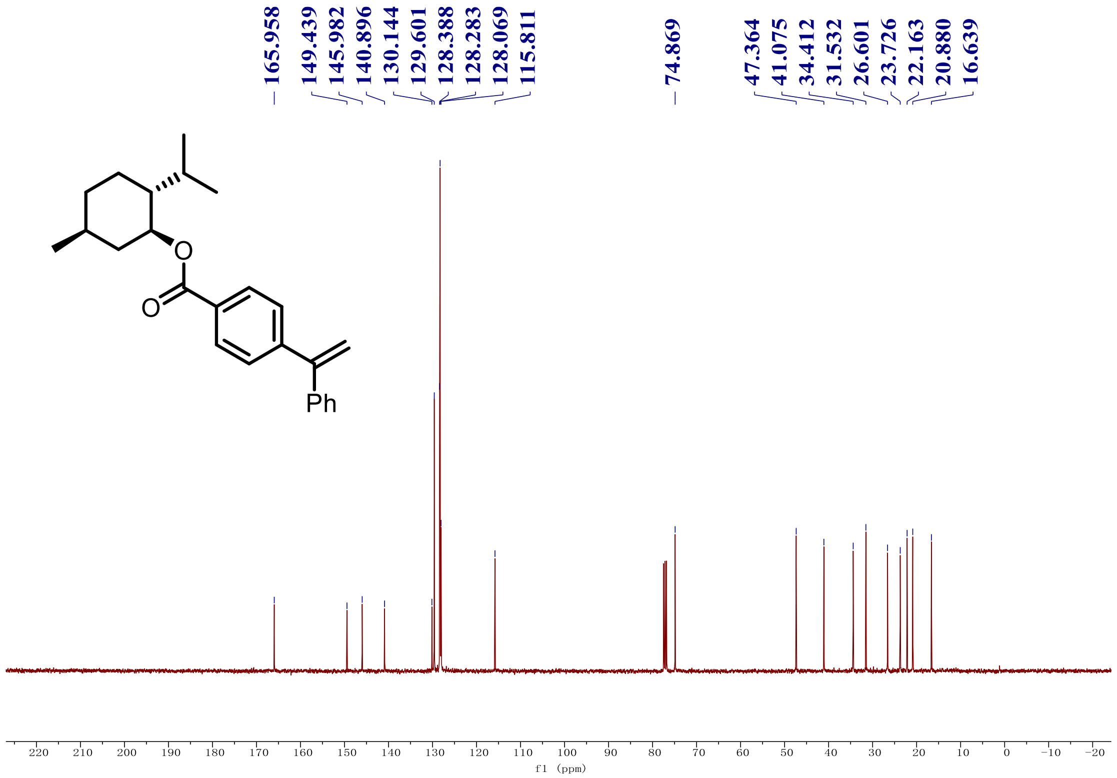

(4-(prop-1-en-2-yl)cyclohex-1-en-1-yl)methyl 4-vinylbenzoate (1ce)
^{1}H NMR (400 MHz, CDCl$_3$)

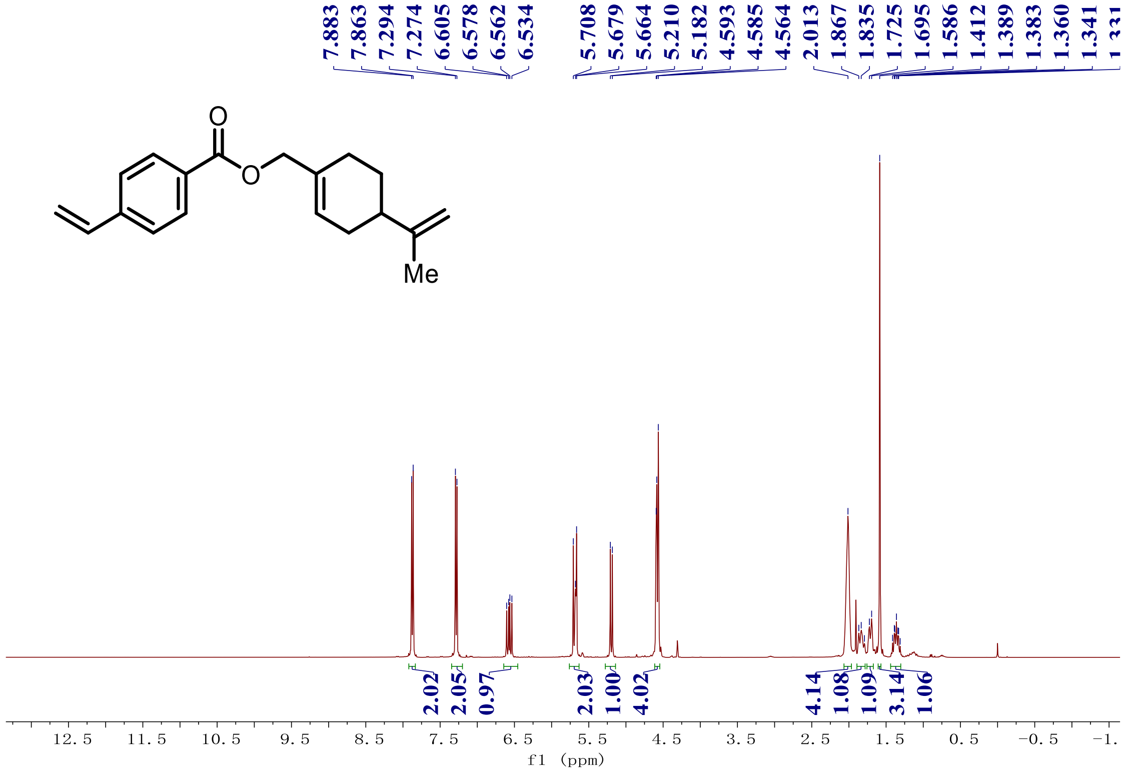

(4-(prop-1-en-2-yl)cyclohex-1-en-1-yl)methyl 4-vinylbenzoate (1ce)
^{13}C NMR (100 MHz, $CDCl_3$)

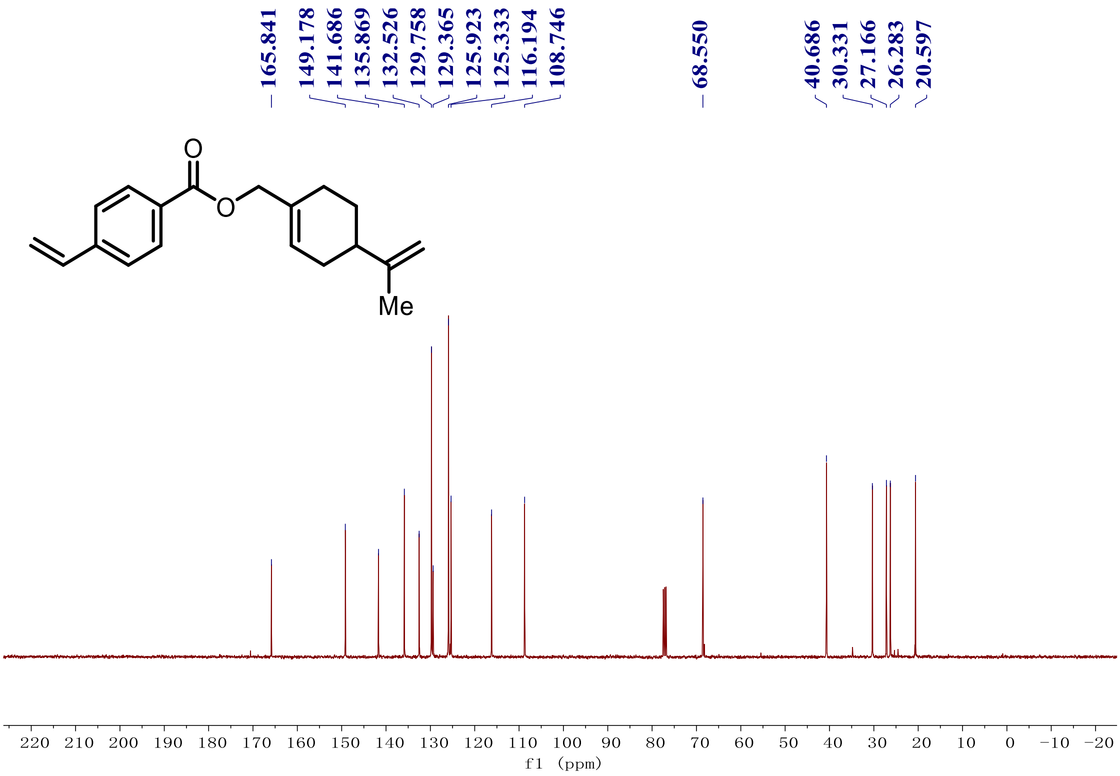

hepta-1,3-dien-4-ylbenzene (3j)
^{1}H NMR (400 MHz, $CDCl_3$)

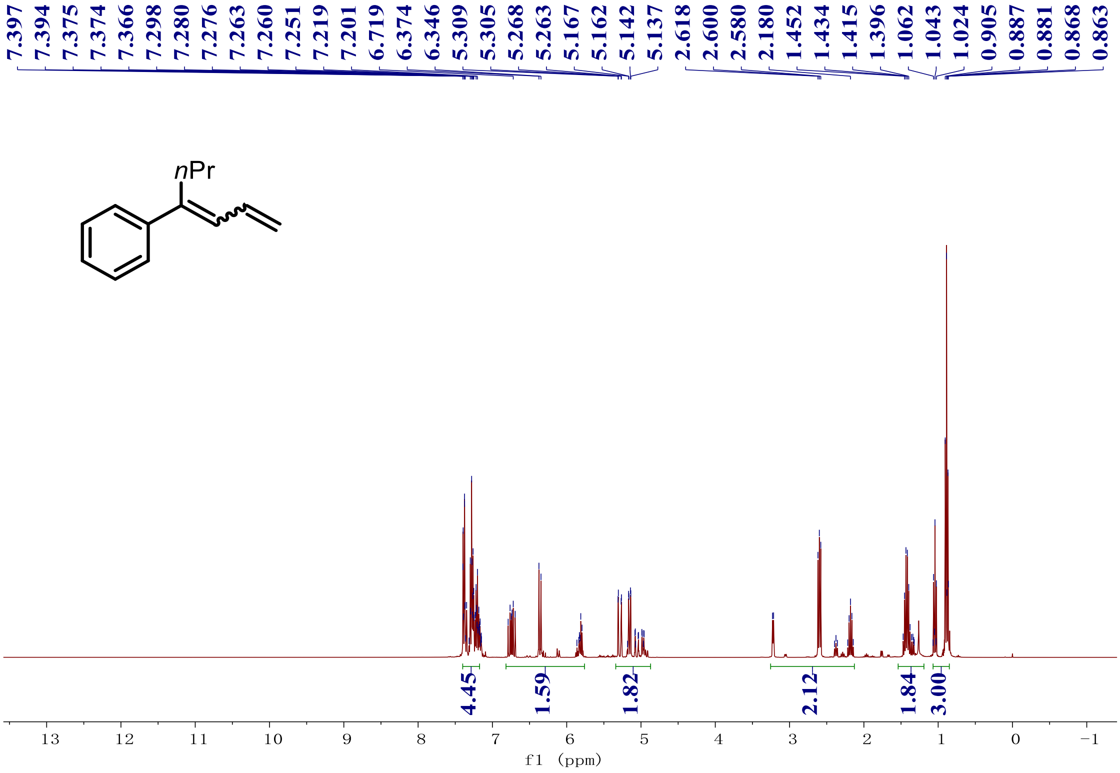

hepta-1,3-dien-4-ylbenzene (3j)

^{13}C NMR (100 MHz, $CDCl_3$)

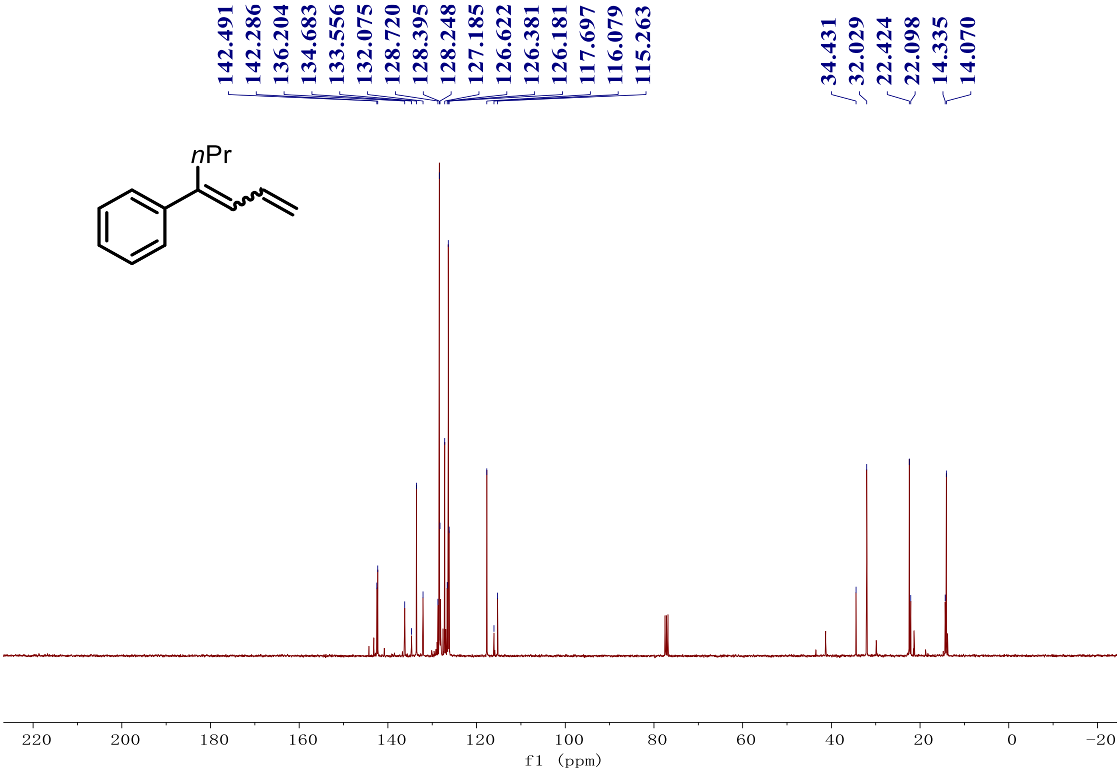

2-([1,1'-biphenyl]-4-yl)succinic acid (2a)

^{1}H NMR (400 MHz, CD_3OD)

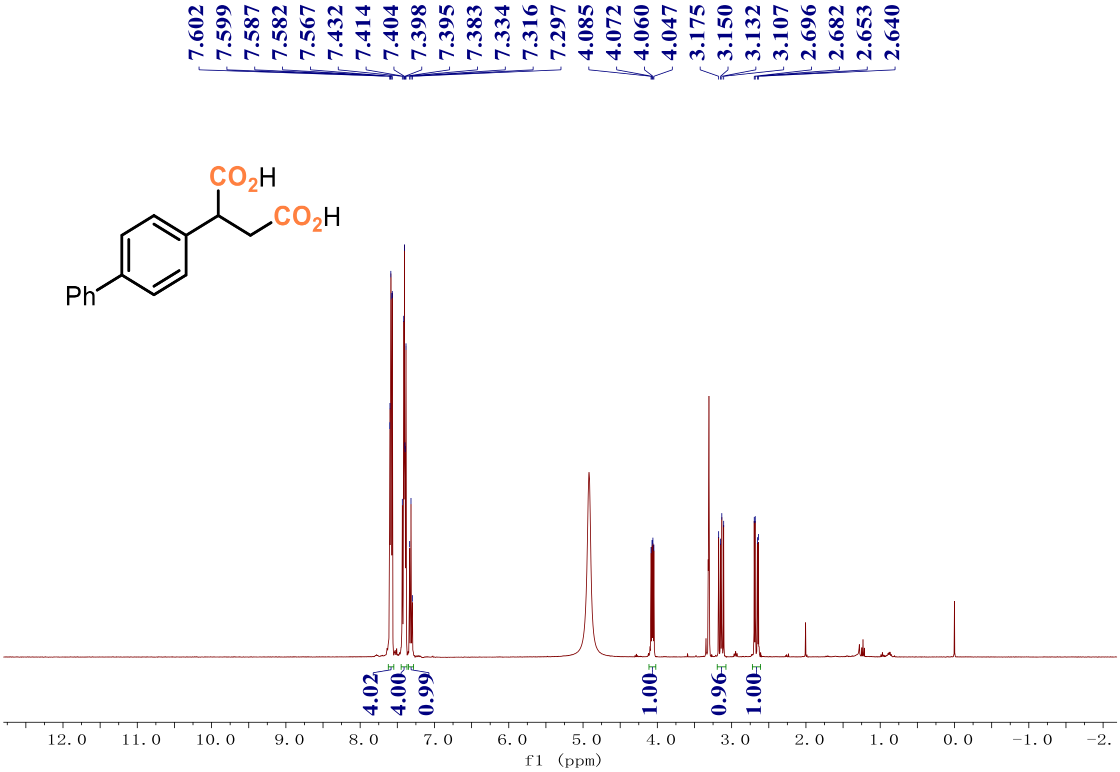

2-([1,1'-biphenyl]-4-yl)succinic acid (2a)

^{13}C NMR (100 MHz, CD_3OD)

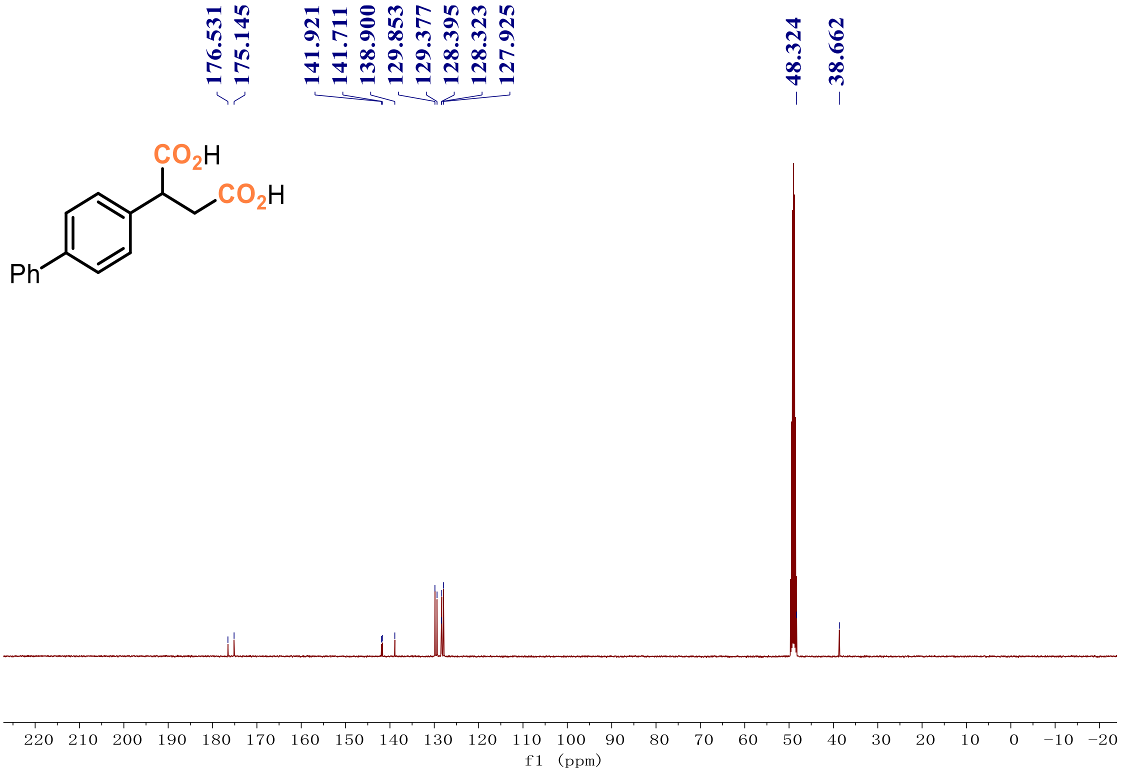

2-cyclohexyl-2-phenylsuccinic acid (2b)

^{1}H NMR (400 MHz, CD_3OD)

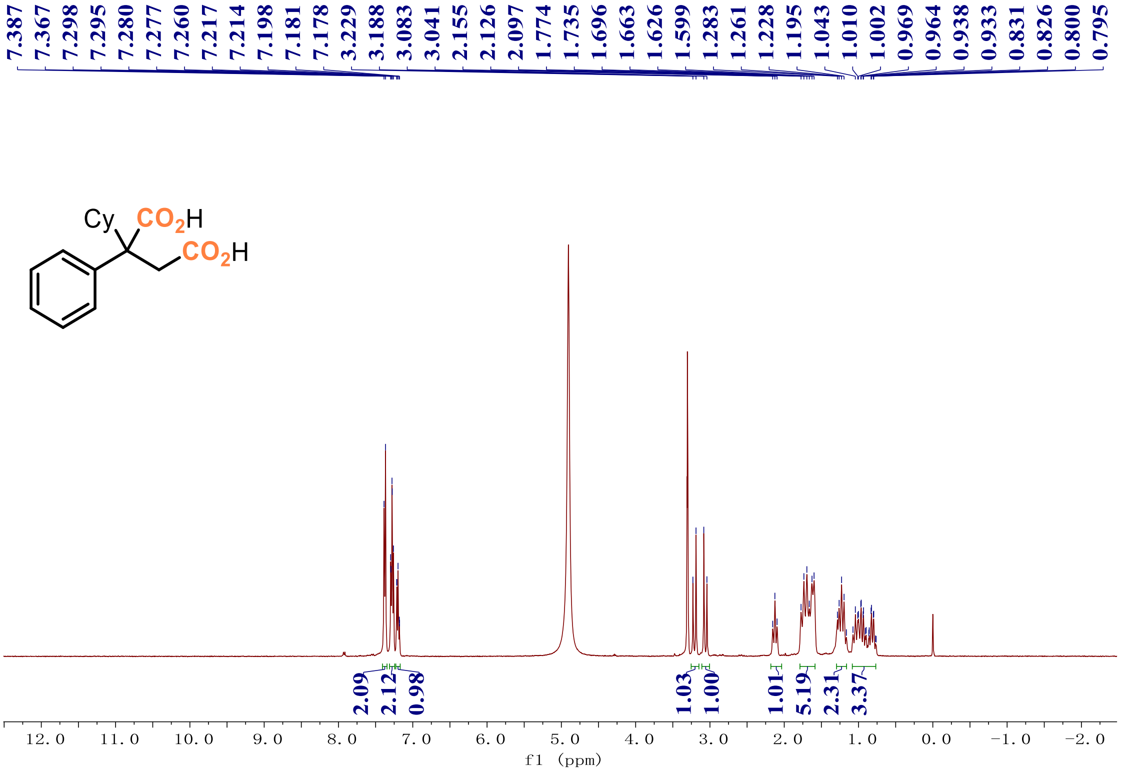

2-cyclohexyl-2-phenylsuccinic acid (2b)
^{13}C NMR (100 MHz, CD_3OD)

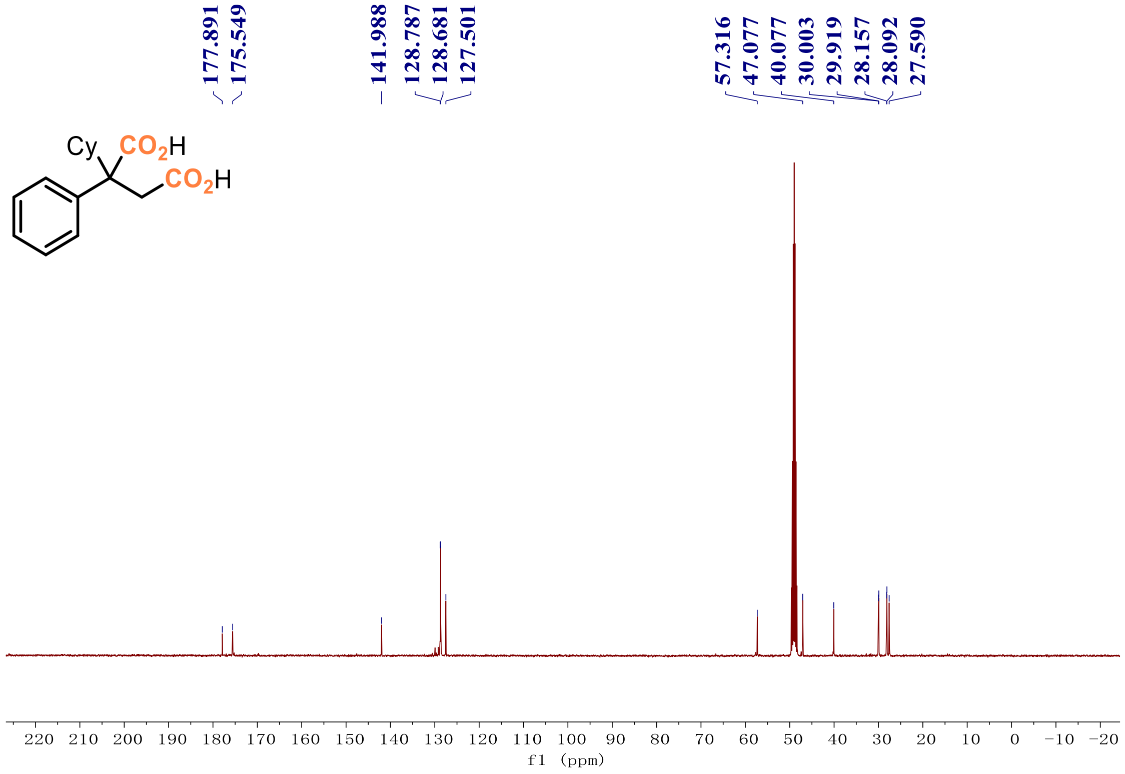

2-phenylsuccinic acid (2c)
^{1}H NMR (400 MHz, CD_3OD)

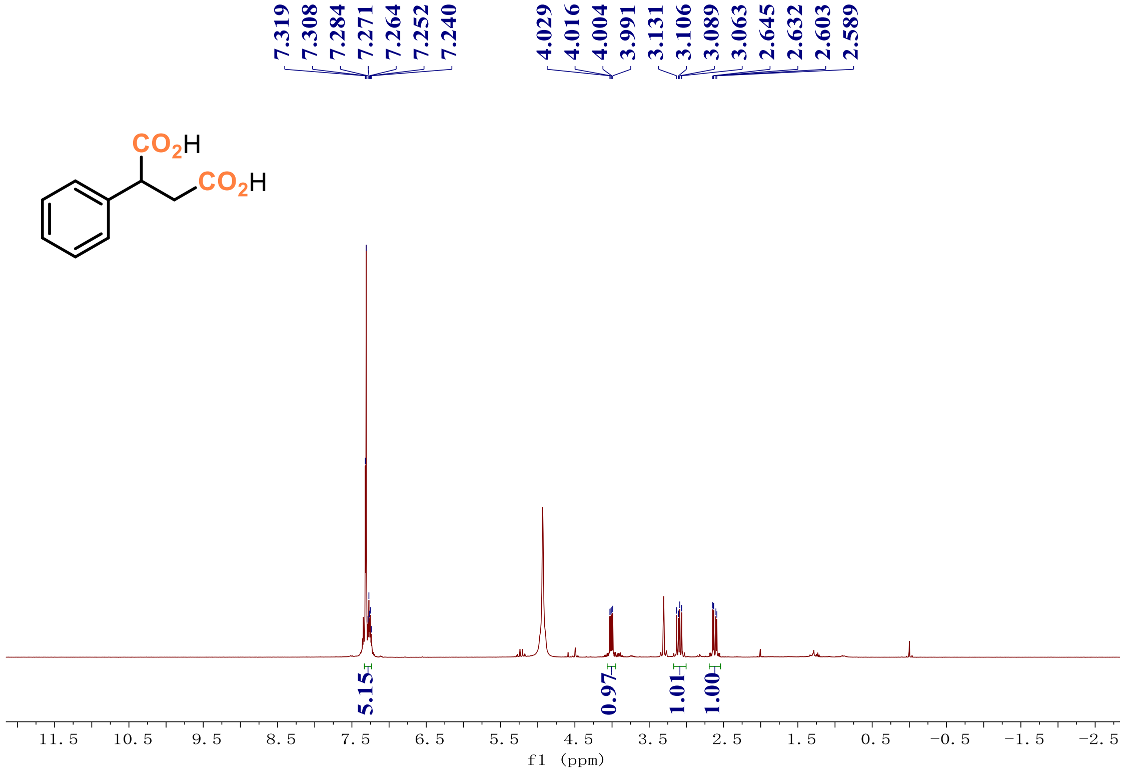

2-phenylsuccinic acid (2c)
^{13}C NMR (100 MHz, CD_3OD)

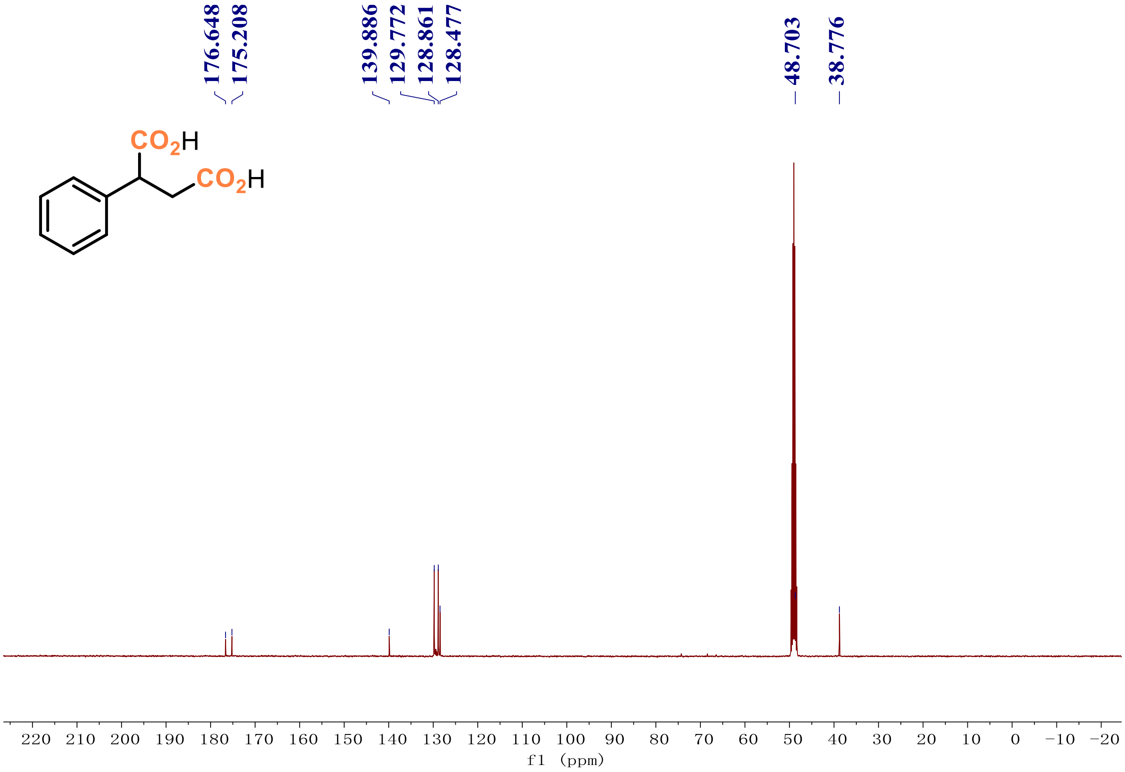

2-(4-cyanophenyl)succinic acid (2d)
^{1}H NMR (400 MHz, CD_3OD)

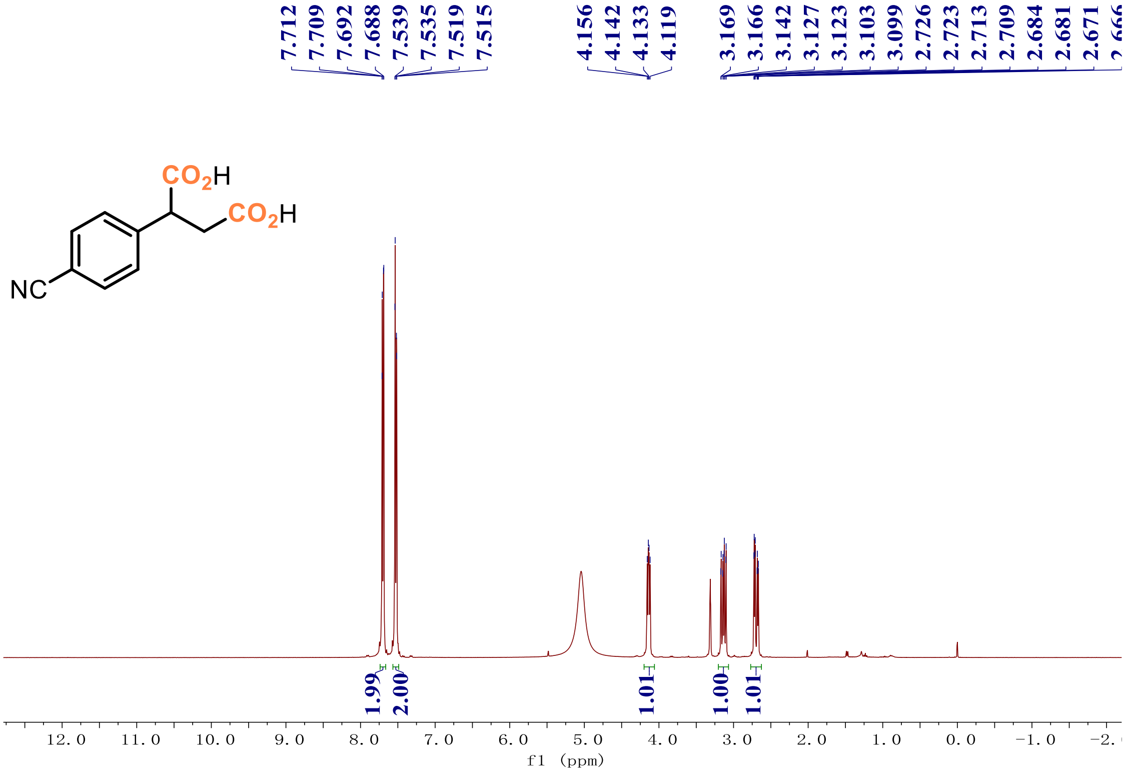

2-(4-cyanophenyl)succinic acid (2d)
^{13}C NMR (100 MHz, CD_3OD)

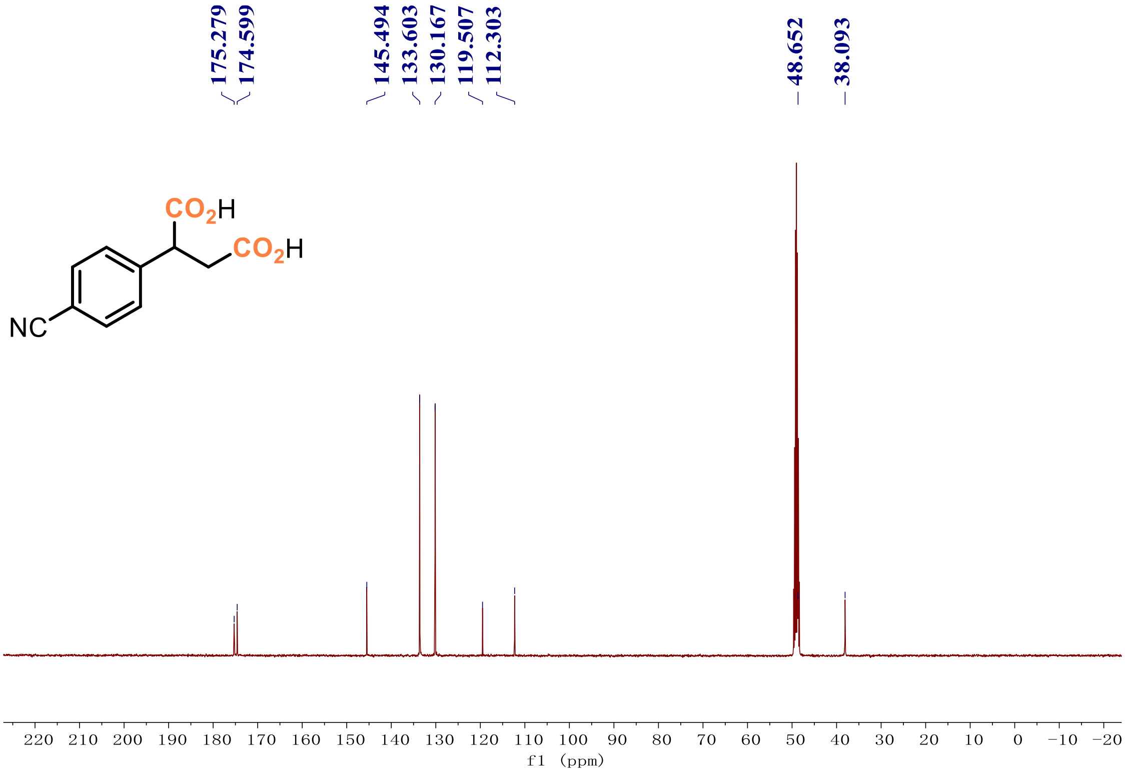

2-(4-methoxyphenyl)succinic acid (2e)
^{1}H NMR (400 MHz, CD_3OD)

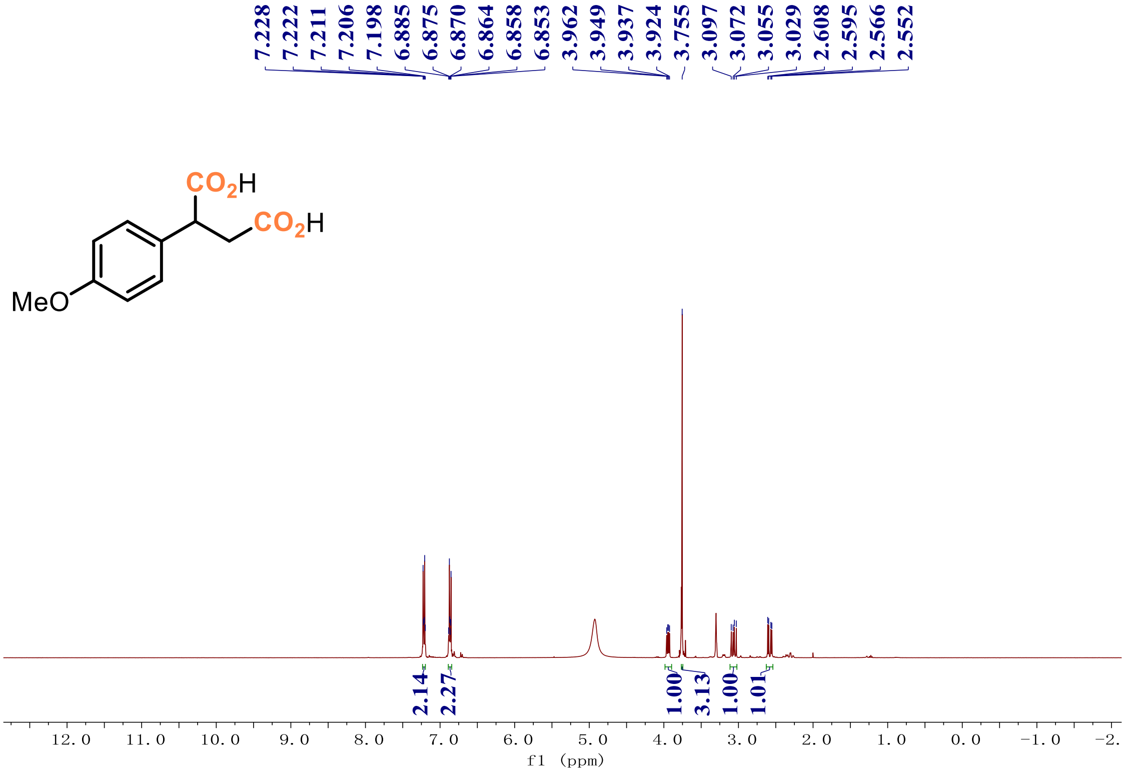

2-(4-methoxyphenyl)succinic acid (2e)
^{13}C NMR (100 MHz, CD_3OD)

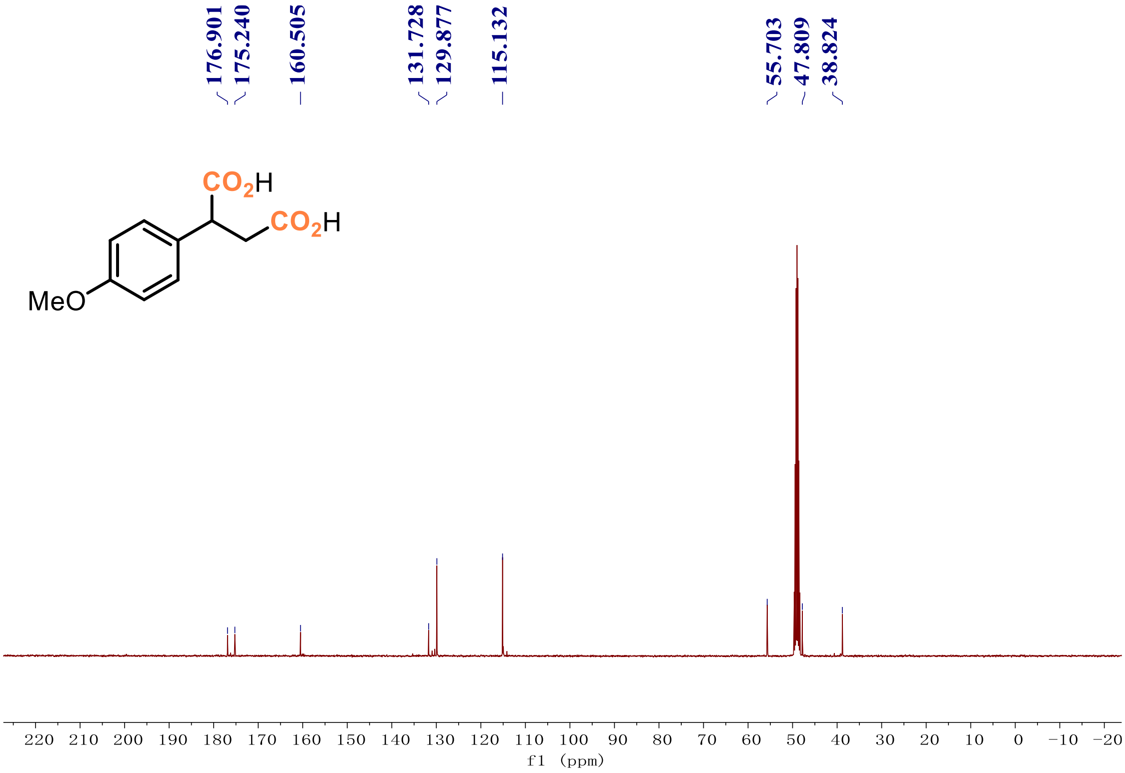

2-methyl-2-phenylsuccinic acid (2f)
^1H NMR (400 MHz, CD_3OD)

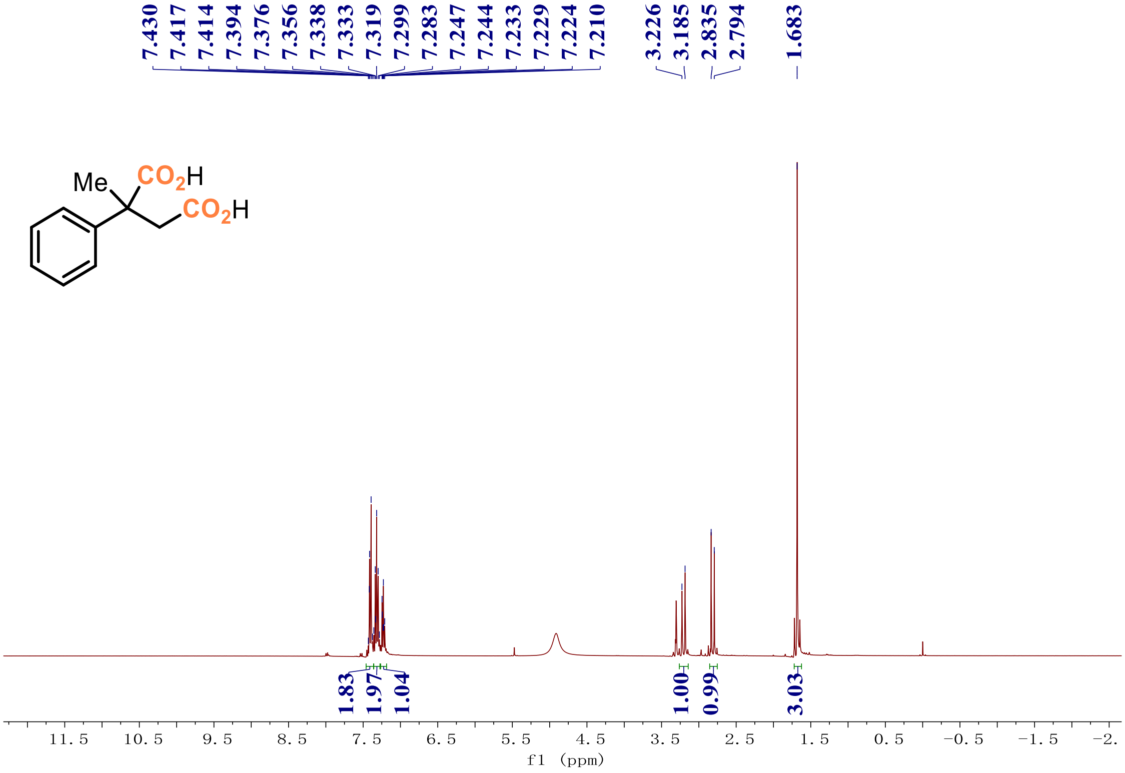

2-methyl-2-phenylsuccinic acid (2f)

^{13}C NMR (100 MHz, CD_3OD)

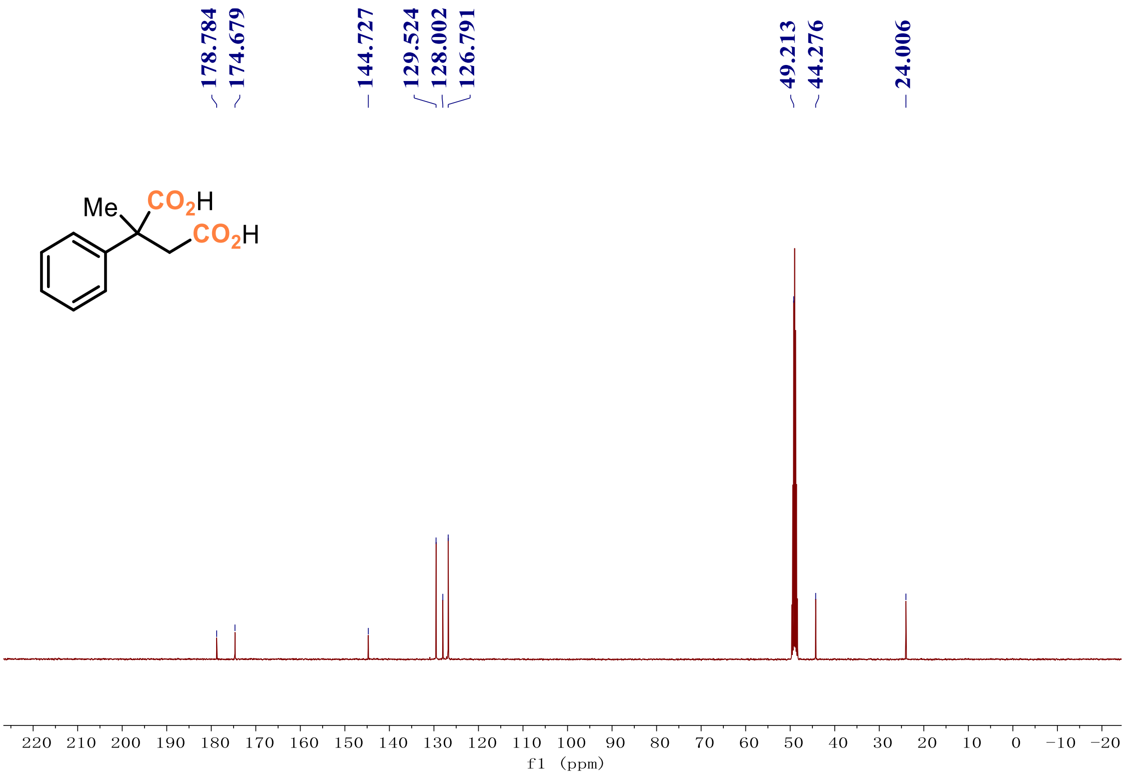

2-cyclopropyl-2-phenylsuccinic acid (2g)

^{1}H NMR (400 MHz, CD_3OD)

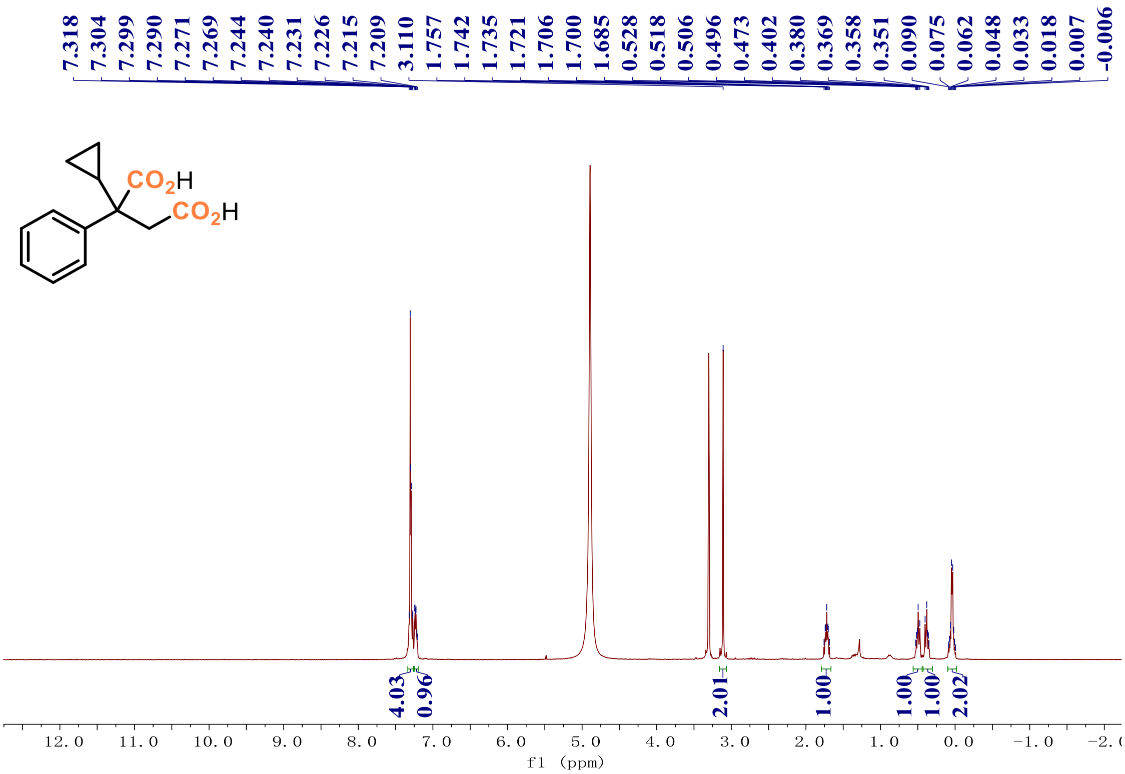

2-cyclopropyl-2-phenylsuccinic acid (2g)
^{13}C NMR (100 MHz, CD_3OD)

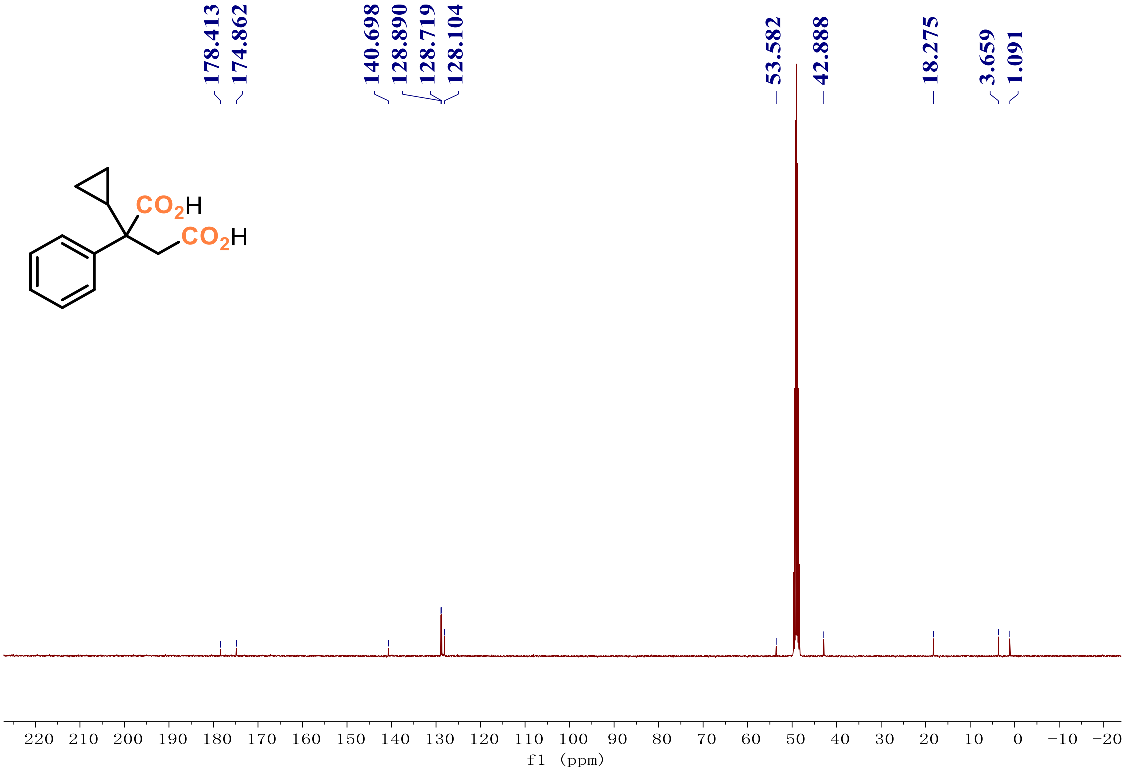

1-(carboxymethyl)-1,2,3,4-tetrahydronaphthalene-1-carboxylic acid (2h)
^{1}H NMR (400 MHz, CD_3OD)

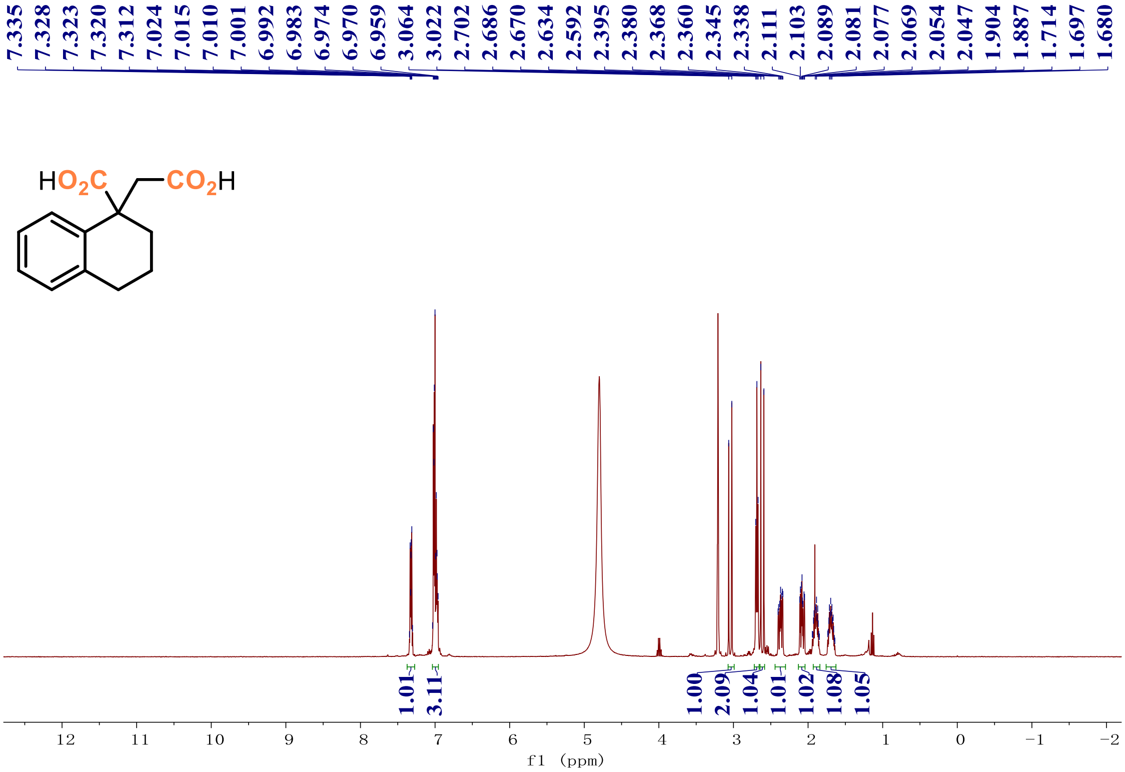

1-(carboxymethyl)-1,2,3,4-tetrahydronaphthalene-1-carboxylic acid (2h)
^{13}C NMR (100 MHz, CD_3OD)

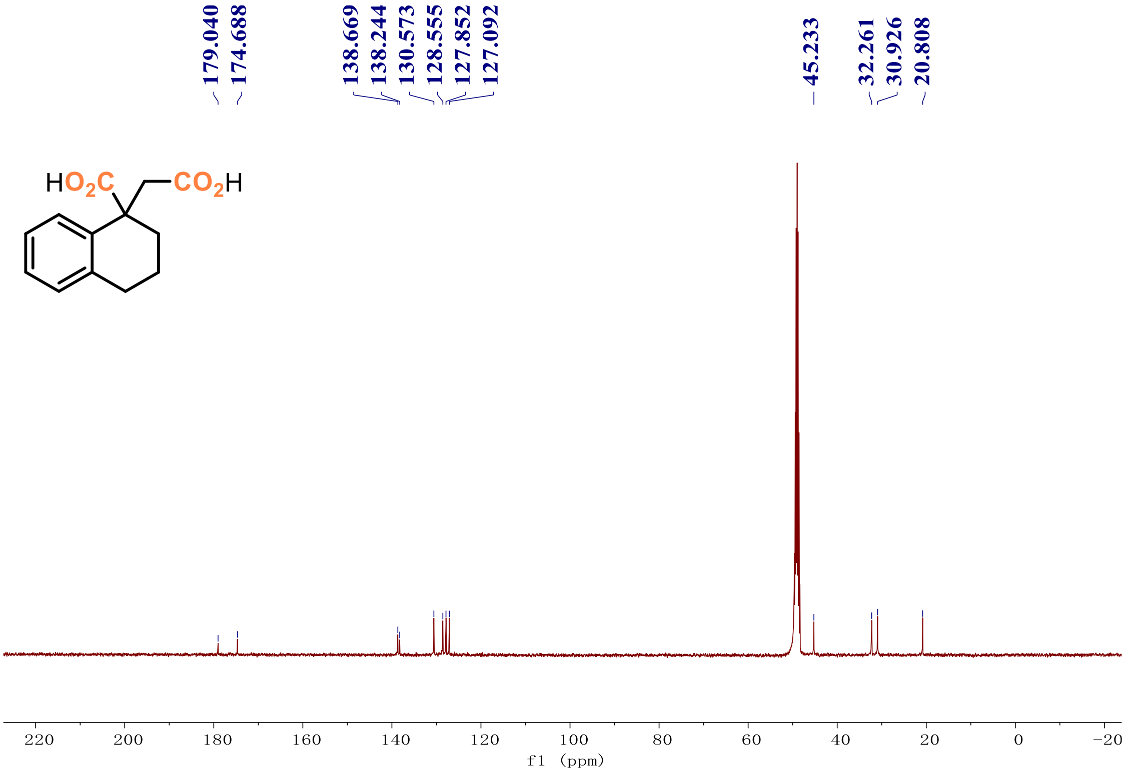

2-([1,1'-biphenyl]-2-yl)succinic acid (2i)
^{1}H NMR (400 MHz, CD_3OD)

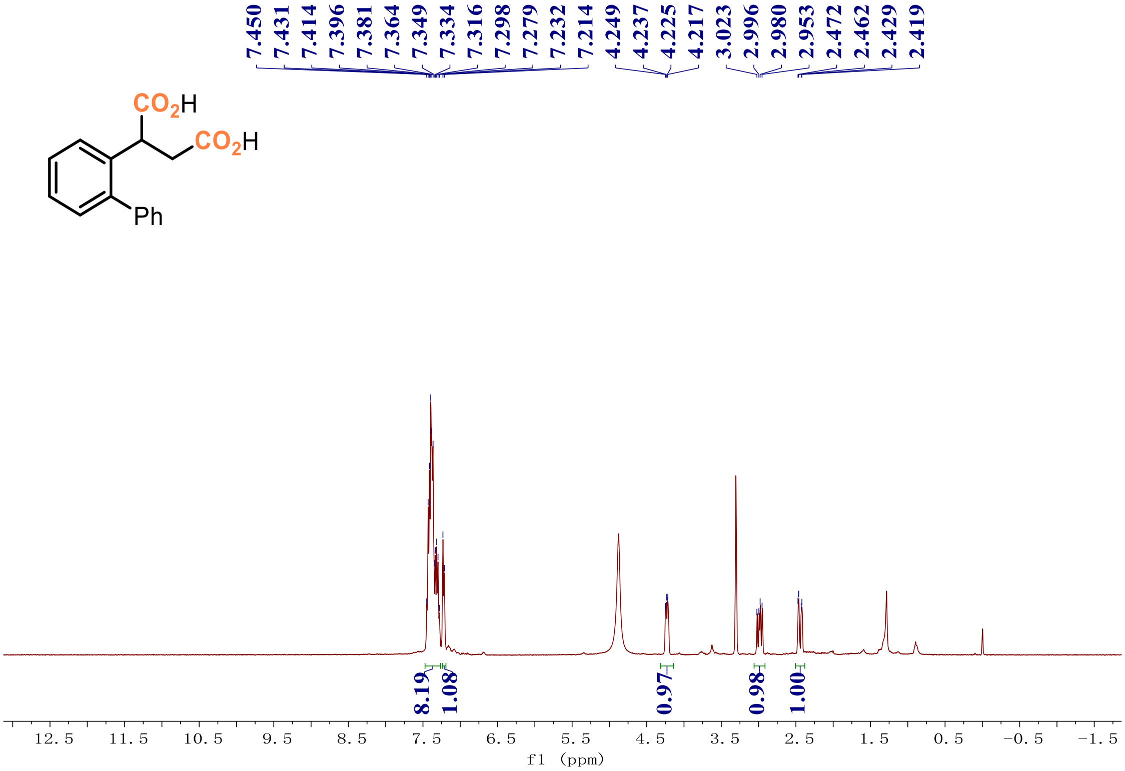

2-([1,1'-biphenyl]-2-yl)succinic acid (2i)

^{13}C NMR (100 MHz, CD_3OD)

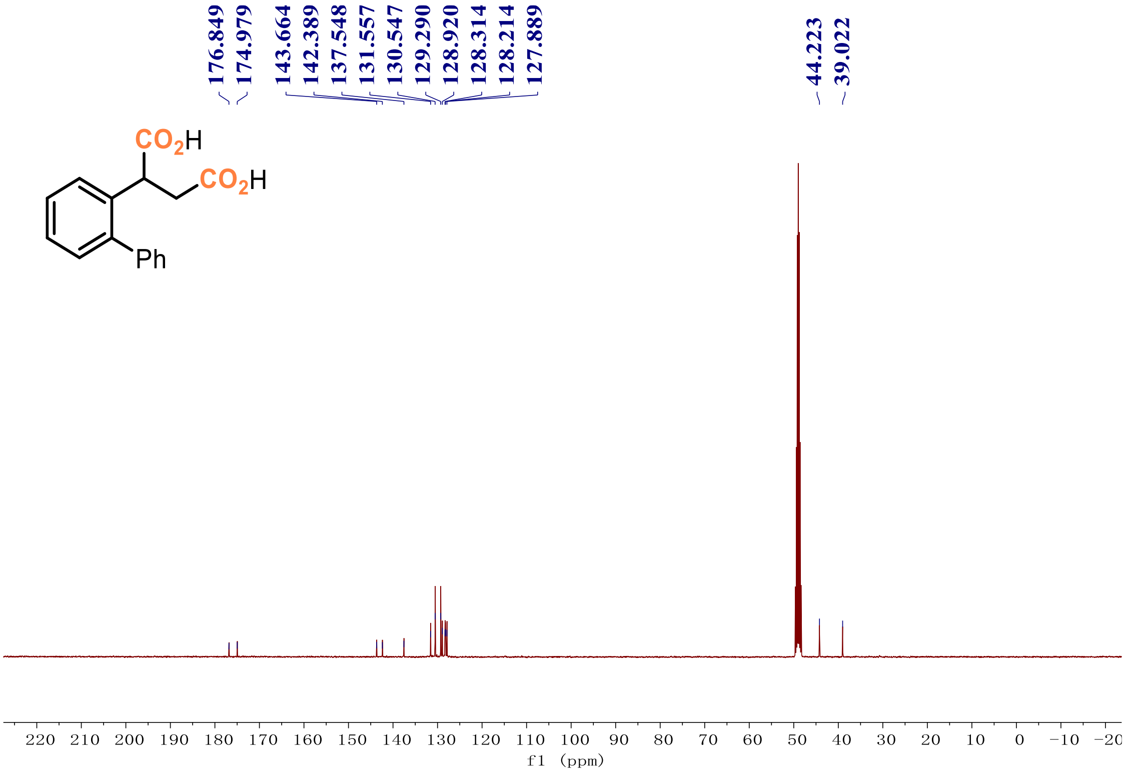

2-(3-phenoxyphenyl)succinic acid (2j)

^{1}H NMR (400 MHz, CD_3OD)

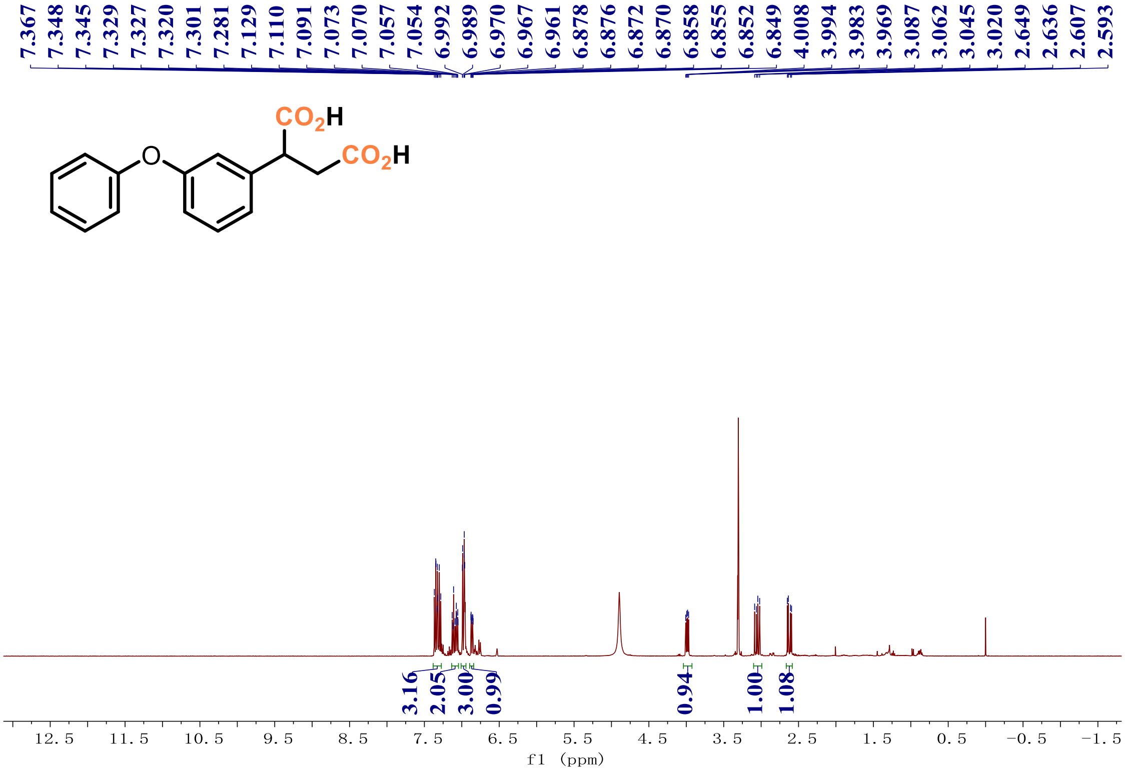

2-(3-phenoxyphenyl)succinic acid (2j)
^{13}C NMR (100 MHz, DMSO-d_6)

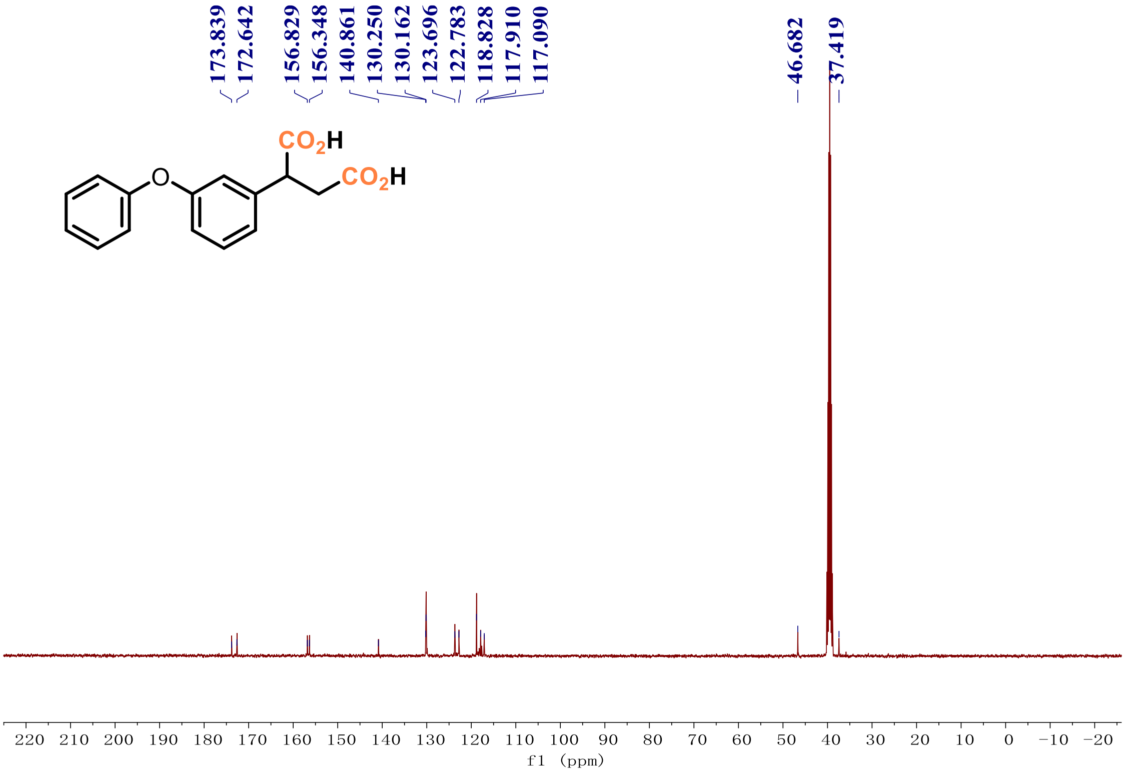

2-(4-benzylphenyl)succinic acid (2k)
^{1}H NMR (400 MHz, DMSO-d_6)

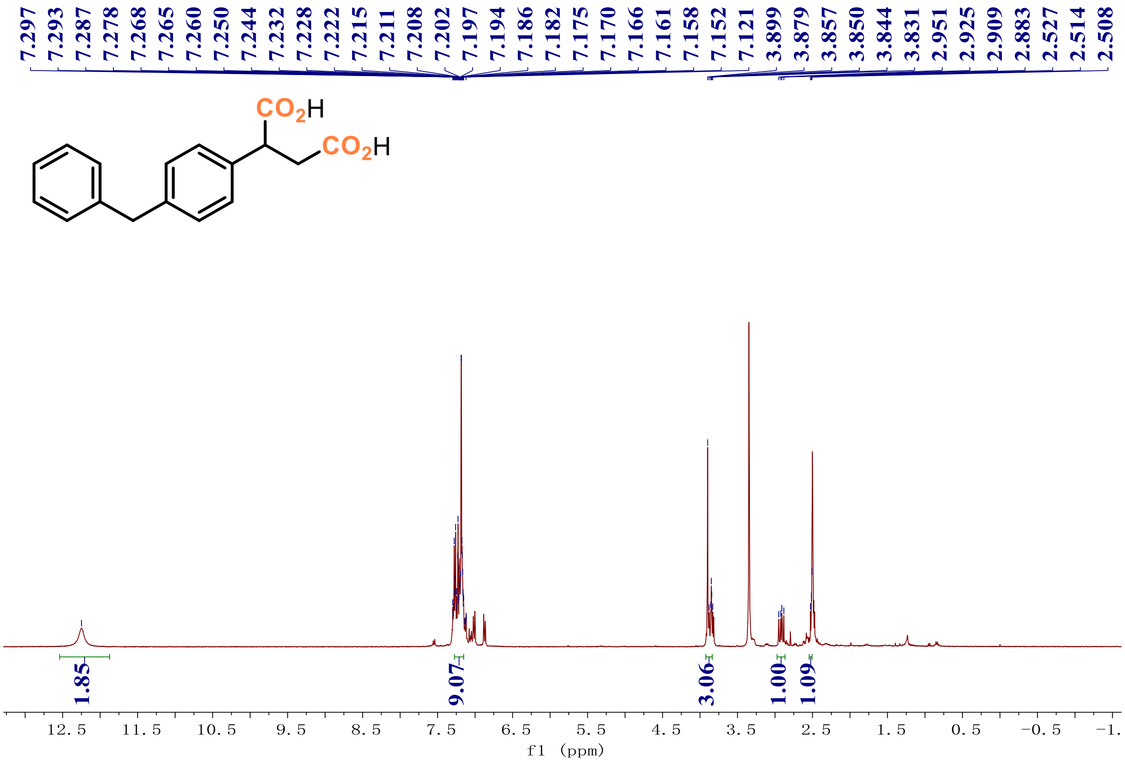

2-(4-benzylphenyl)succinic acid (2k)

^{13}C NMR (100 MHz, DMSO-d_6)

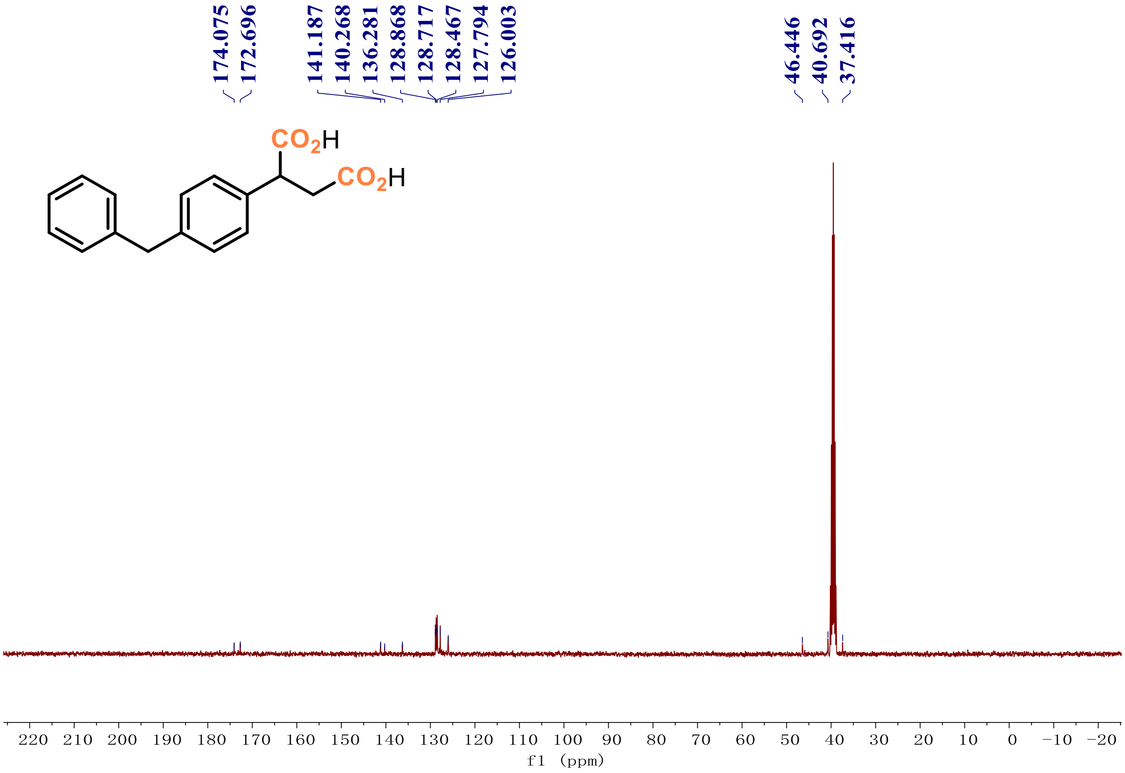

2-([1,1'-biphenyl]-2-yl)succinic acid (2l)

^{1}H NMR (400 MHz, CD_3OD)

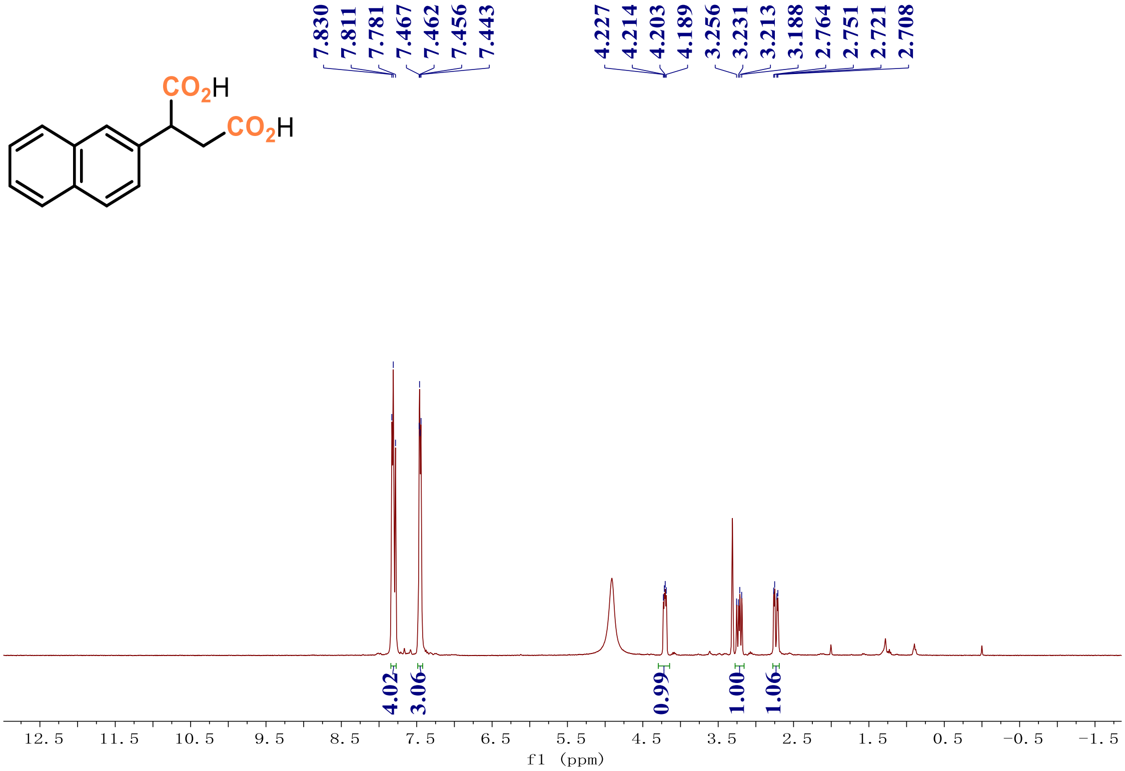

2-([1,1'-biphenyl]-2-yl)succinic acid (2l)

^{13}C NMR (100 MHz, CD_3OD)

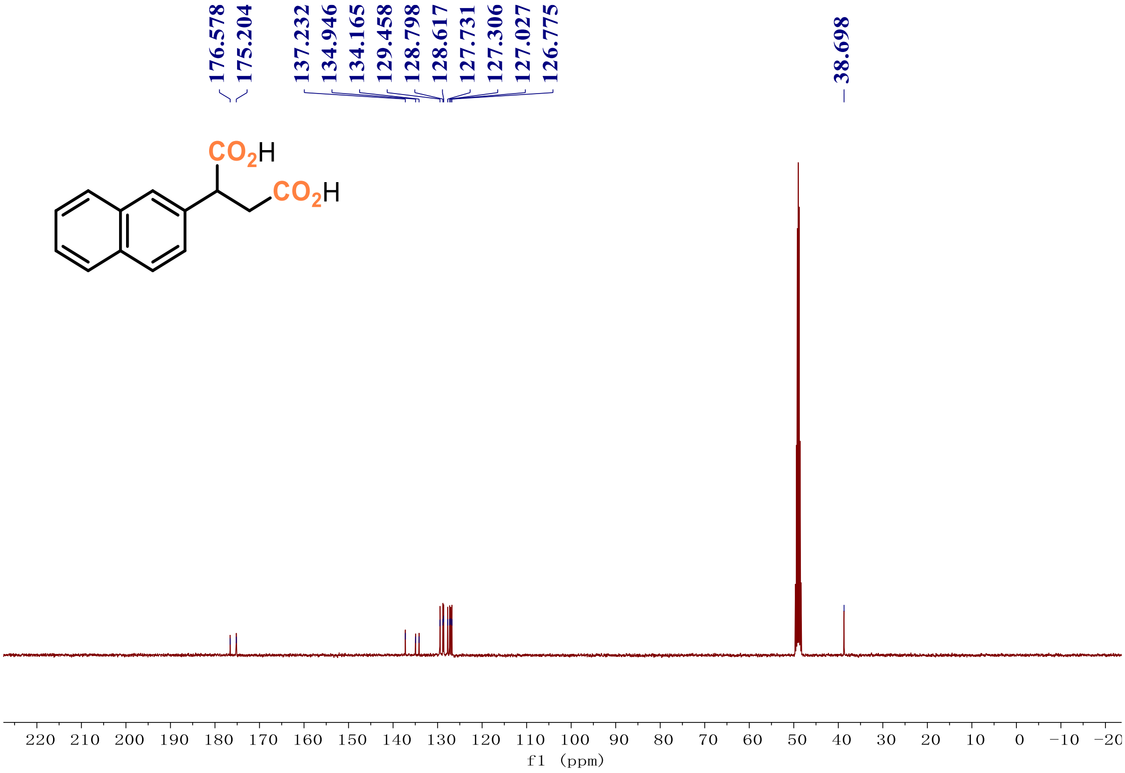

2-(6-methoxynaphthalen-2-yl)succinic acid (2m)

^{1}H NMR (400 MHz, CD_3OD)

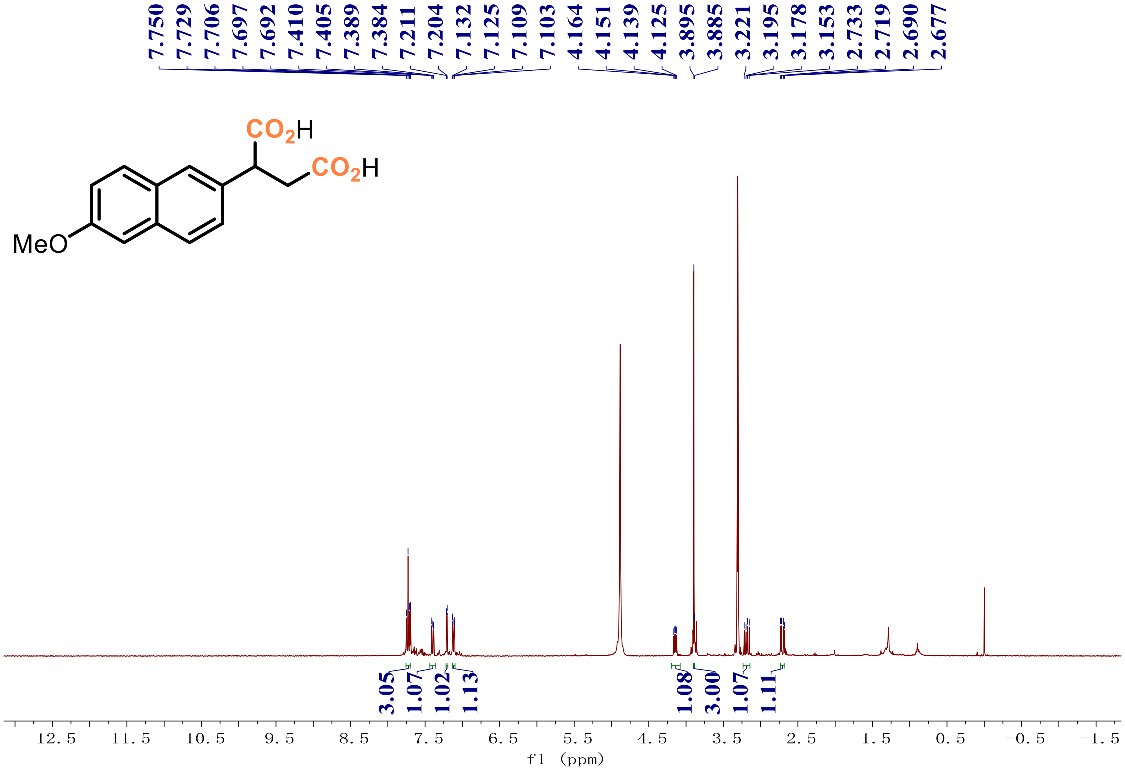

2-(6-methoxynaphthalen-2-yl)succinic acid (2m)
^{13}C NMR (100 MHz, CD_3OD)

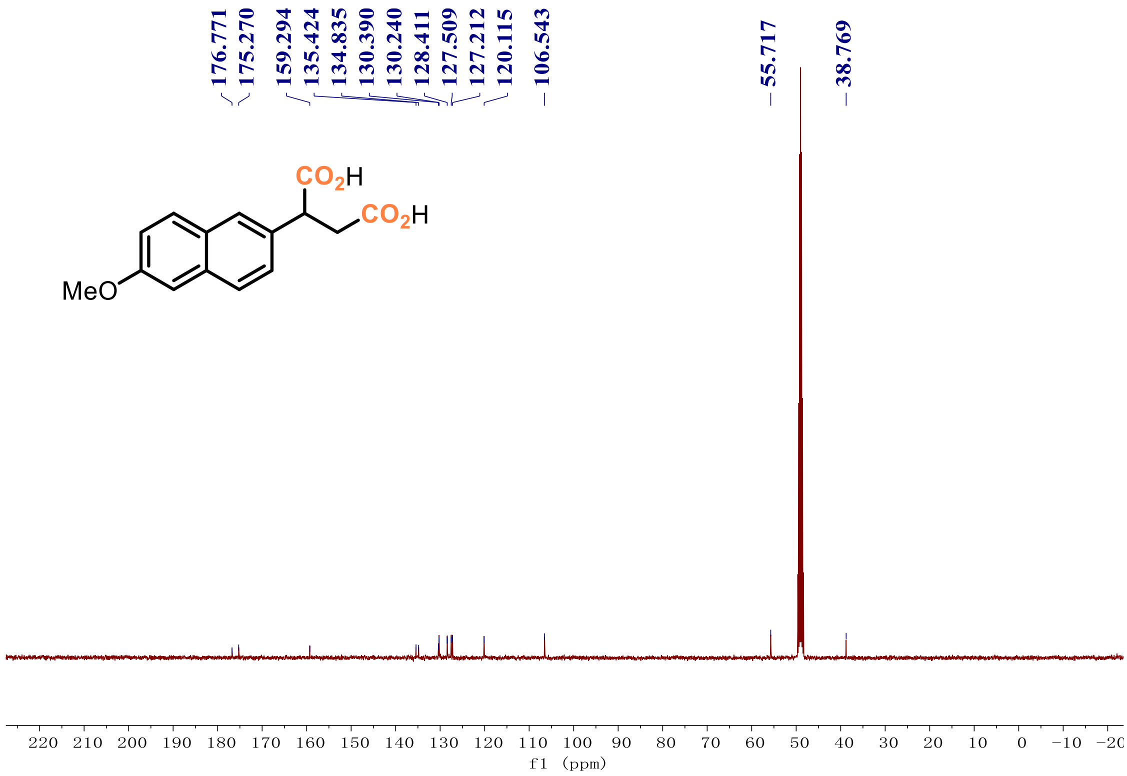

2-(4-(1*H*-pyrrol-1-yl)phenyl)succinic acid (2n)
^{1}H NMR (400 MHz, CD_3OD)

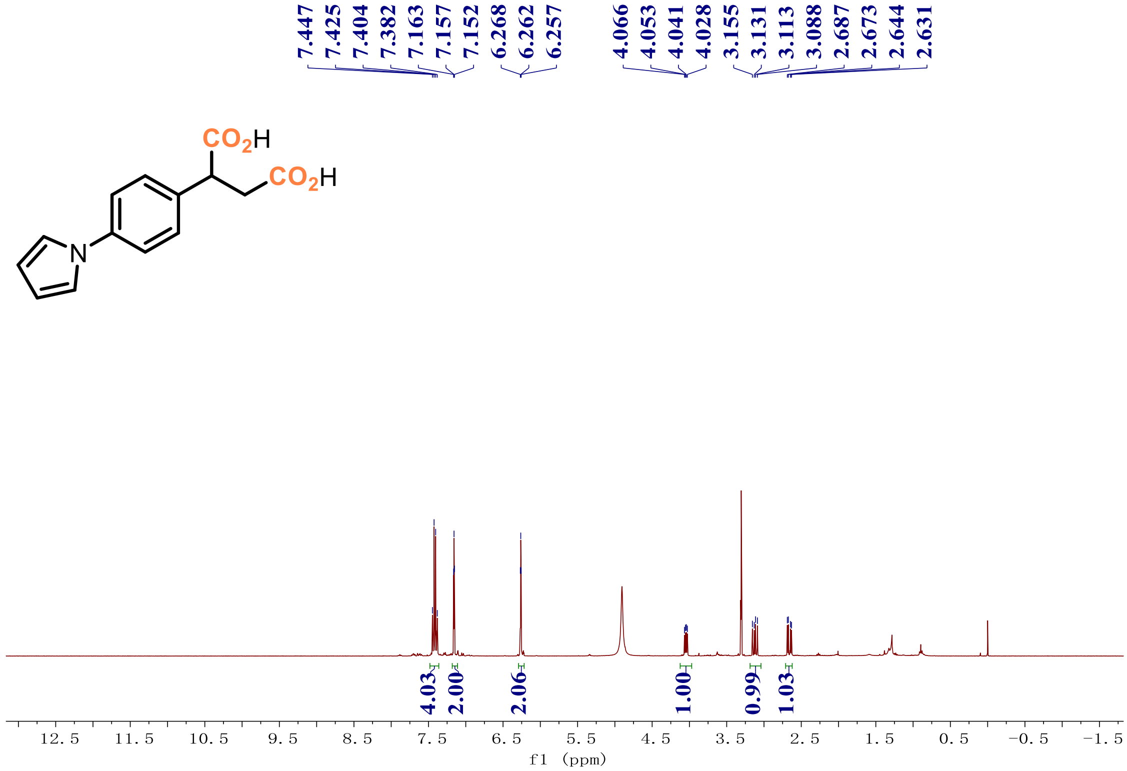

2-(4-(1*H*-pyrrol-1-yl)phenyl)succinic acid (2n)
^{13}C NMR (100 MHz, CD_3OD)

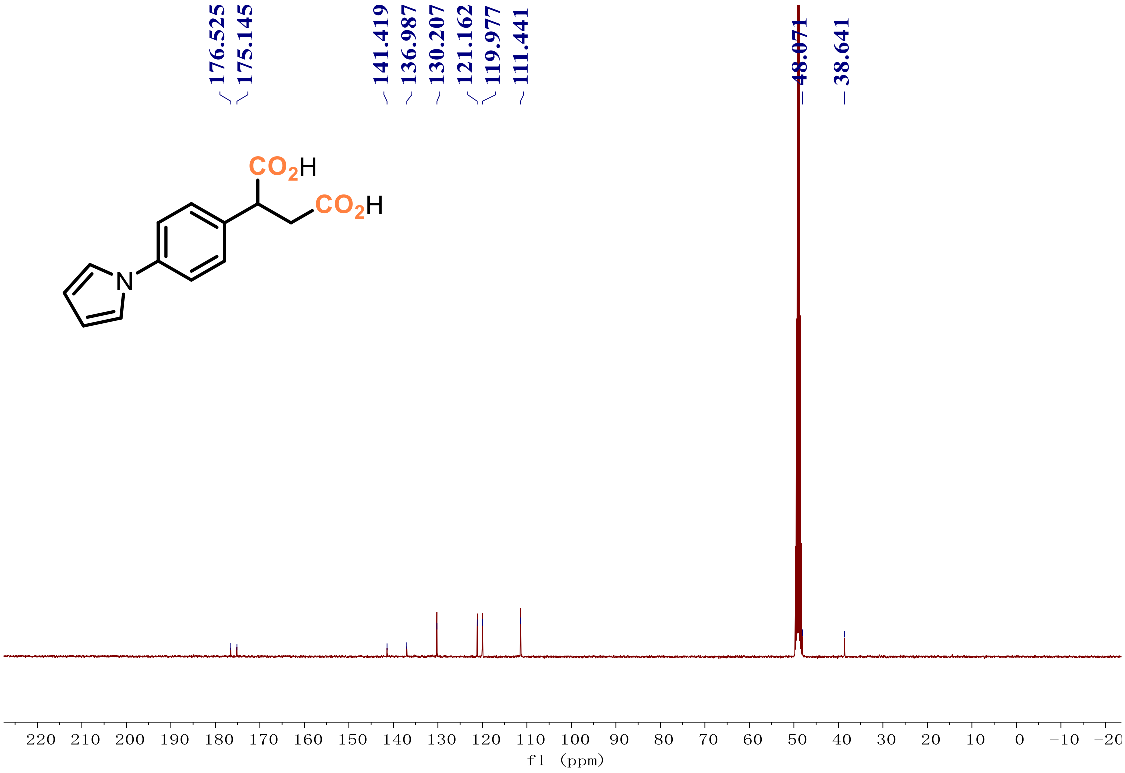

2-(4'-methoxy-[1,1'-biphenyl]-2-yl)succinic acid (2o)
^{1}H NMR (400 MHz, CD_3OD)

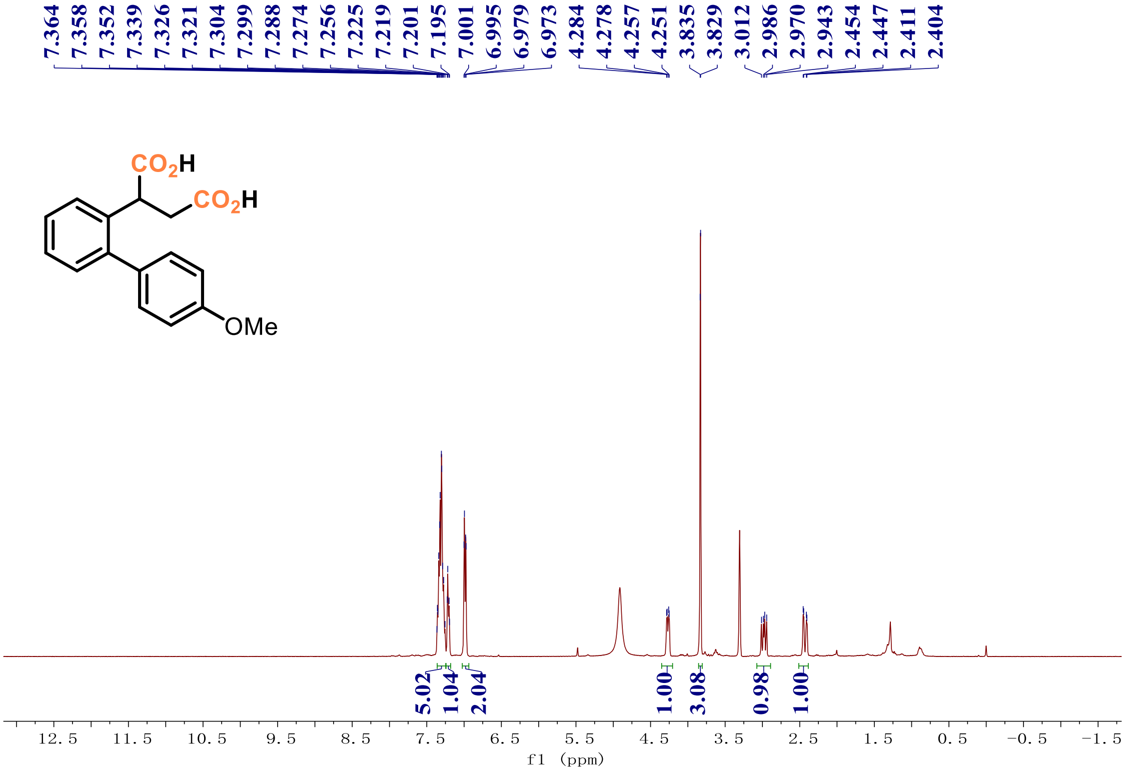

2-(4'-methoxy-[1,1'-biphenyl]-2-yl)succinic acid (2o)

^{13}C NMR (100 MHz, CD_3OD)

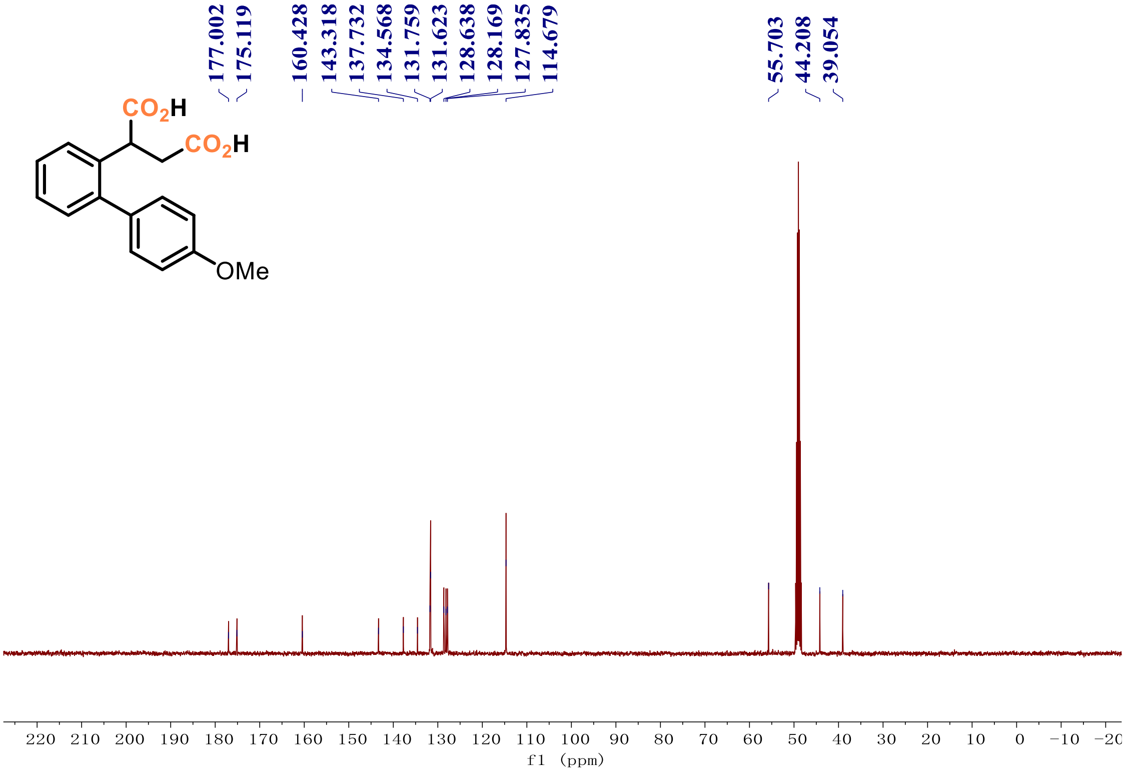

2-(5-methoxy-[1,1'-biphenyl]-2-yl)succinic acid (2p)

^{1}H NMR (400 MHz, CD_3OD)

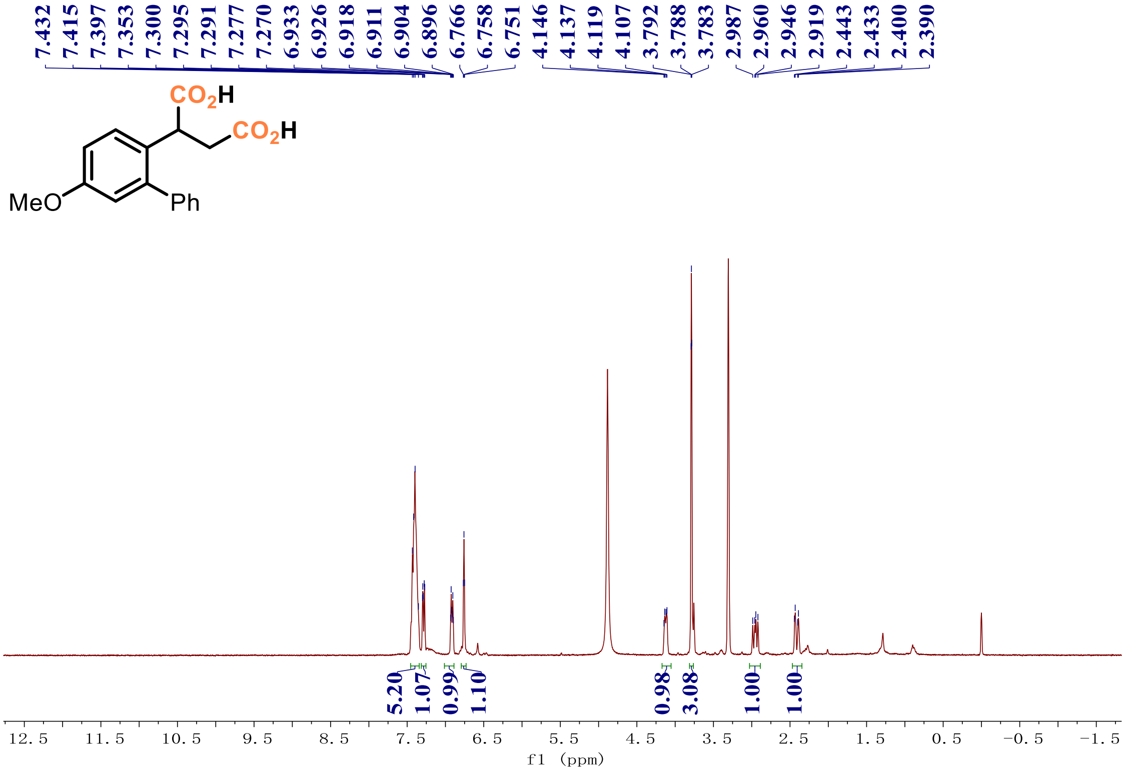

2-(5-methoxy-[1,1'-biphenyl]-2-yl)succinic acid (2p)

^{13}C NMR (100 MHz, CD_3OD)

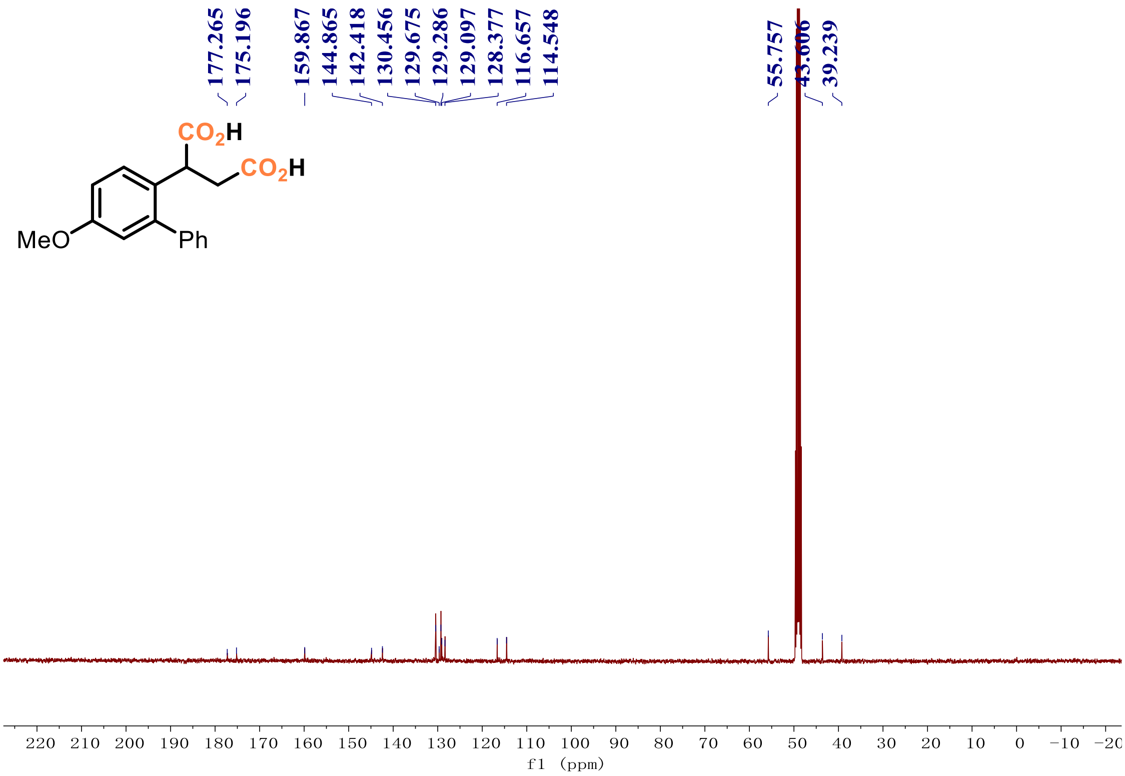

2-(4'-cyano-[1,1'-biphenyl]-2-yl)succinic acid (2q)

^{1}H NMR (400 MHz, CD_3OD)

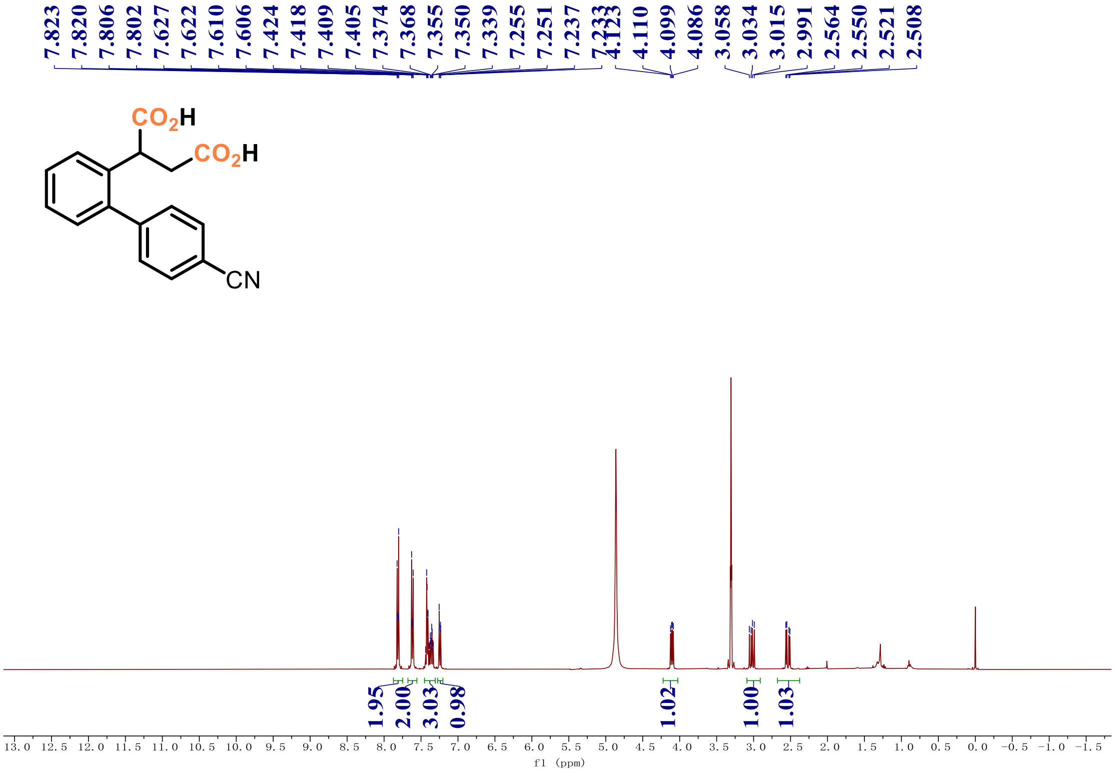

2-(4'-cyano-[1,1'-biphenyl]-2-yl)succinic acid (2q)
^{13}C NMR (100 MHz, CD_3OD)

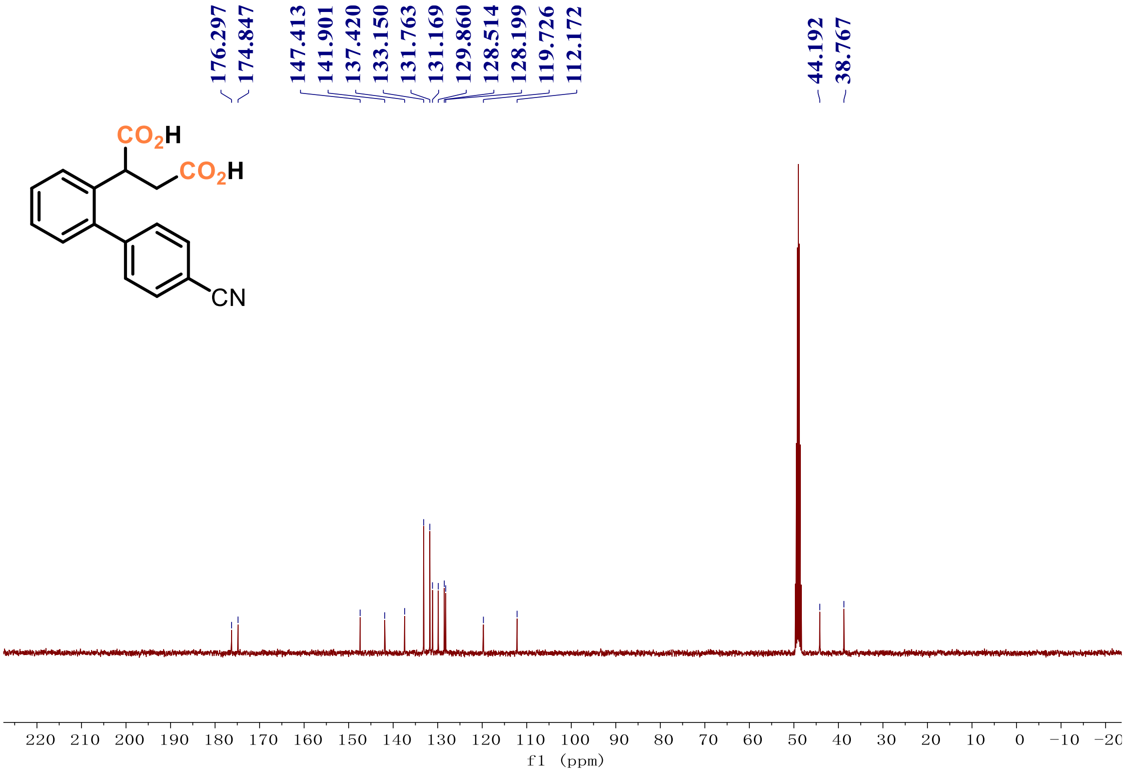

2-(4'-(methoxycarbonyl)-[1,1'-biphenyl]-4-yl)succinic acid (2r)
^{1}H NMR (400 MHz, DMSO-*d*$_6$)

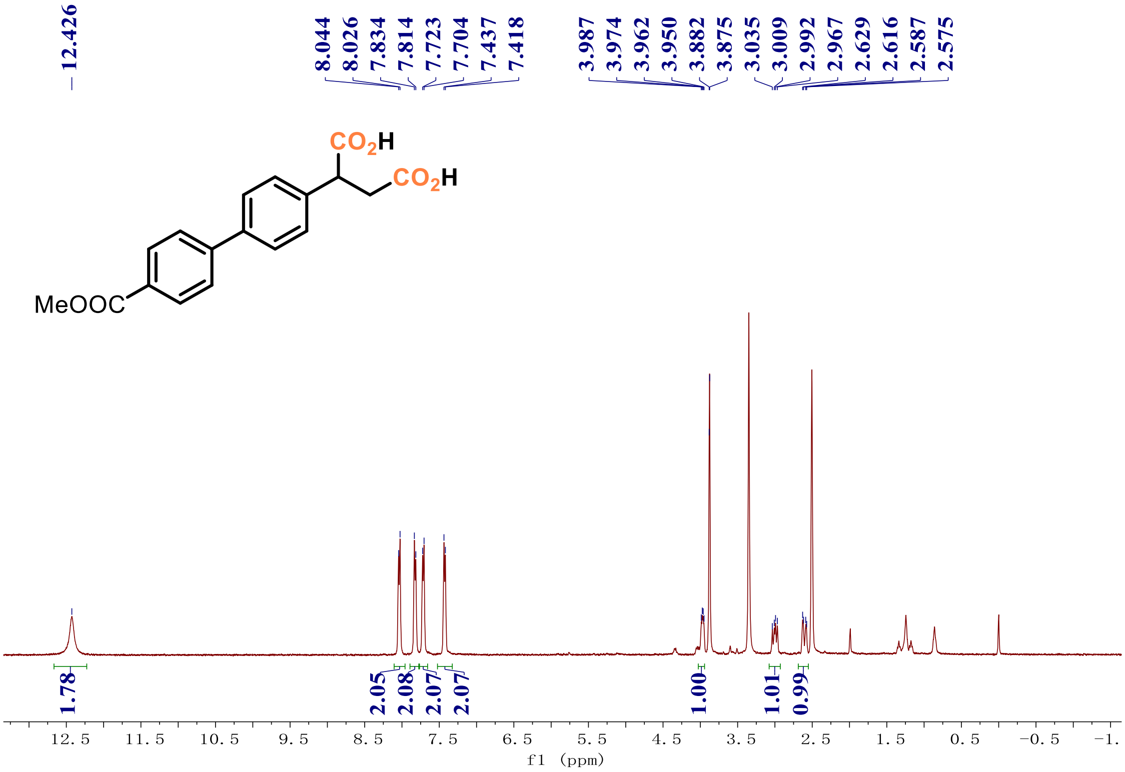

2-(4'-(methoxycarbonyl)-[1,1'-biphenyl]-4-yl)succinic acid (2r)

^{13}C NMR (100 MHz, DMSO-*d*$_6$)

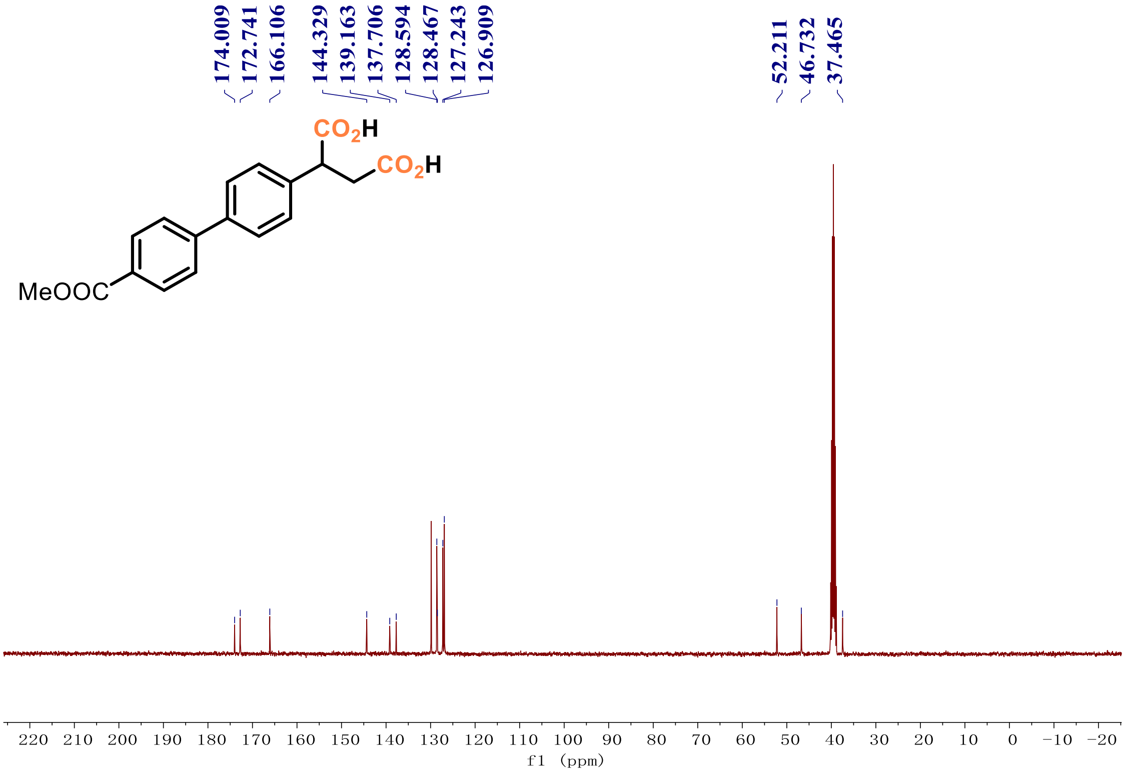

2-(3'-fluoro-[1,1'-biphenyl]-2-yl)succinic acid (2s)

^{1}H NMR (400 MHz, CD$_3$OD)

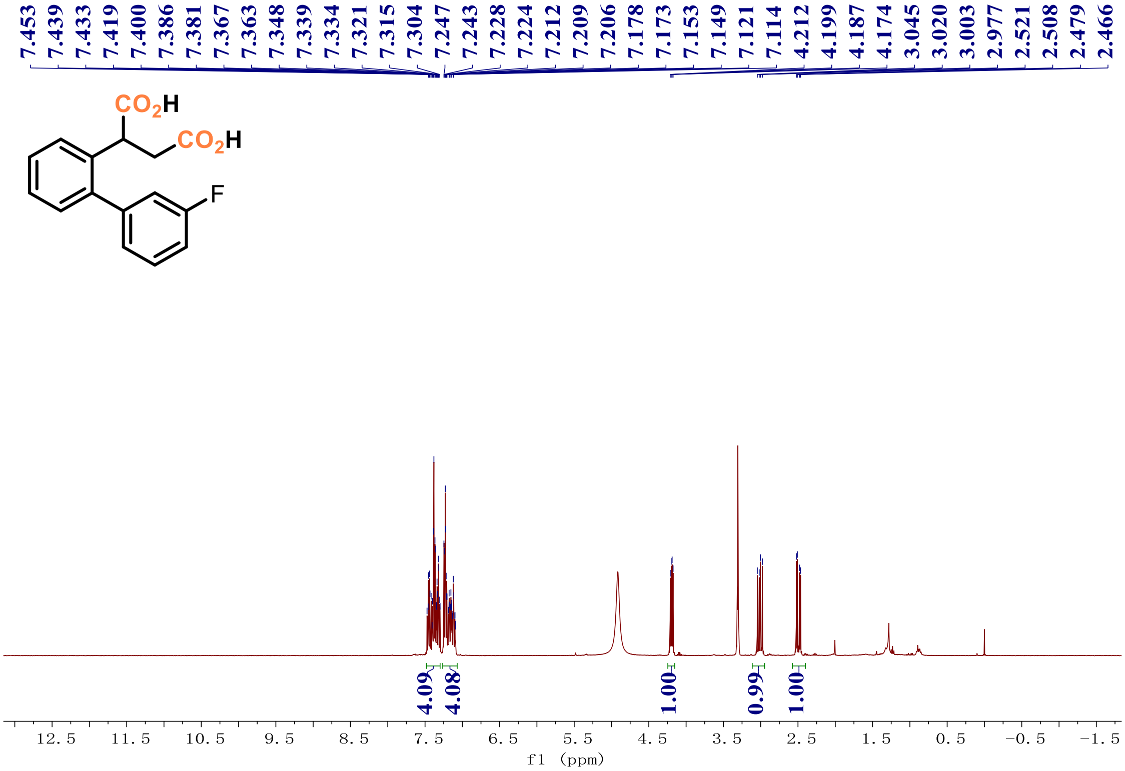

2-(3'-fluoro-[1,1'-biphenyl]-2-yl)succinic acid (2s)

^{13}C NMR (100 MHz, CD_3OD)

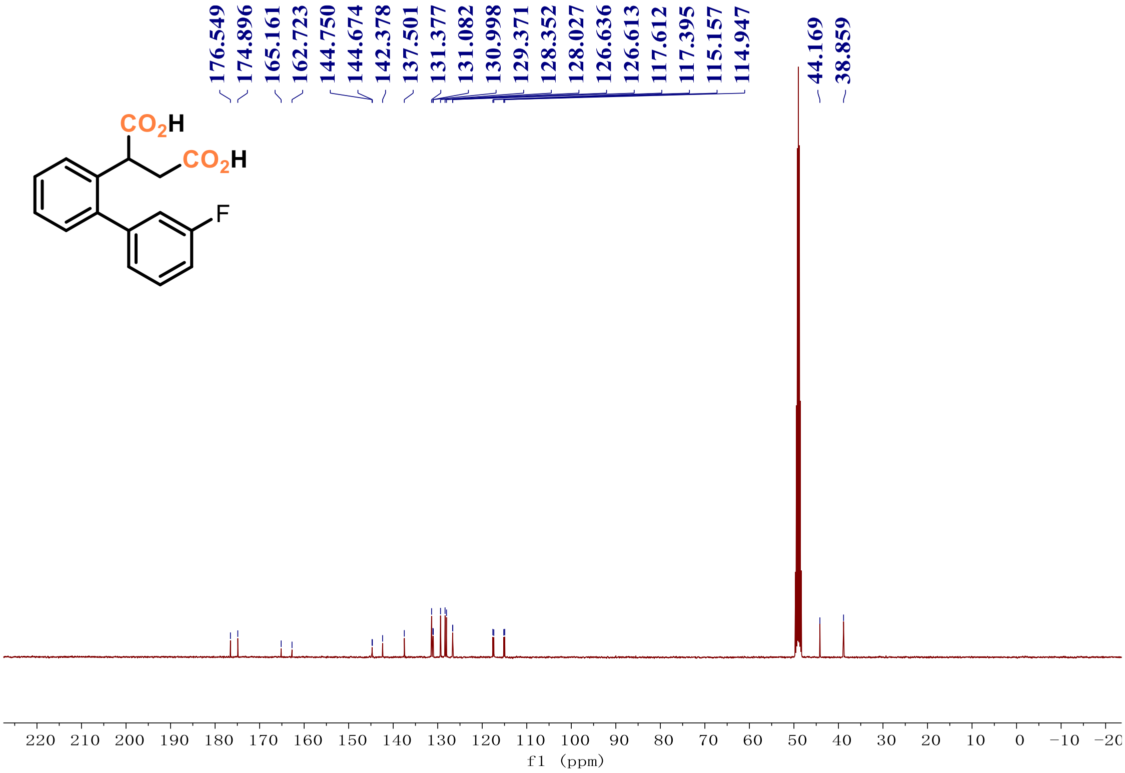

2-(3'-fluoro-[1,1'-biphenyl]-2-yl)succinic acid (2s)

^{19}F NMR (376 MHz, CD_3OD)

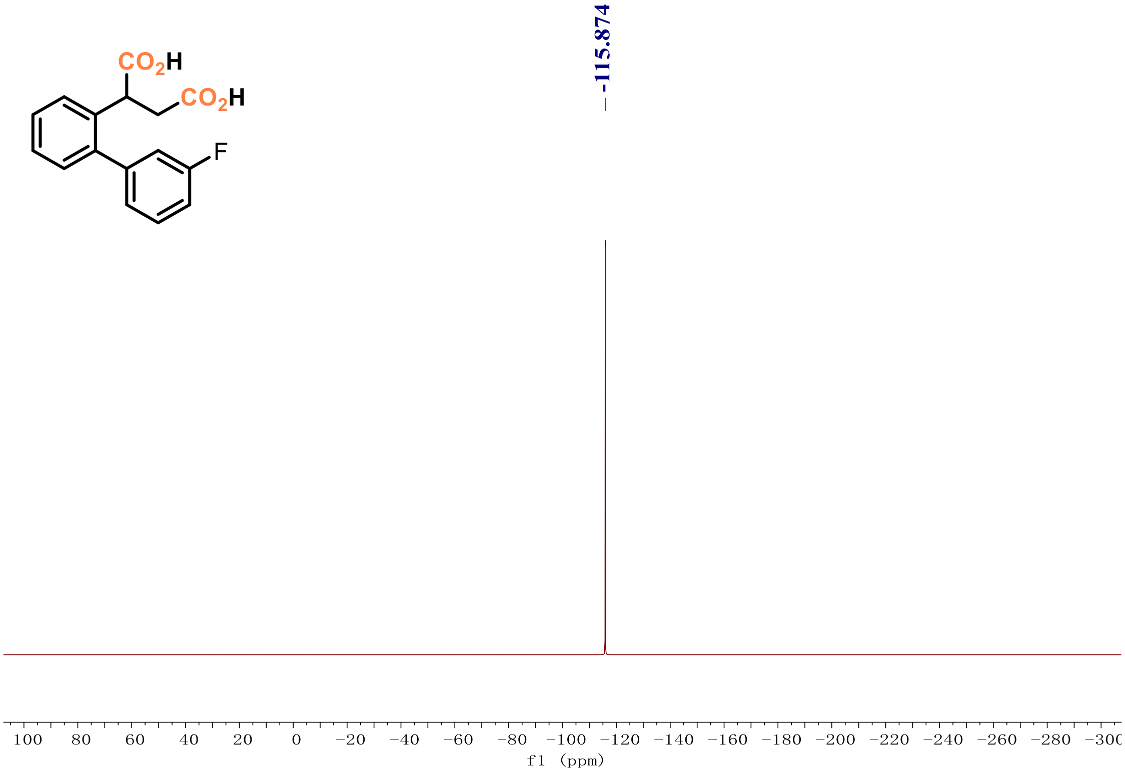

2-(4'-(trimethylsilyl)-[1,1'-biphenyl]-2-yl)succinic acid (2t)

^{1}H NMR (400 MHz, CD_3OD)

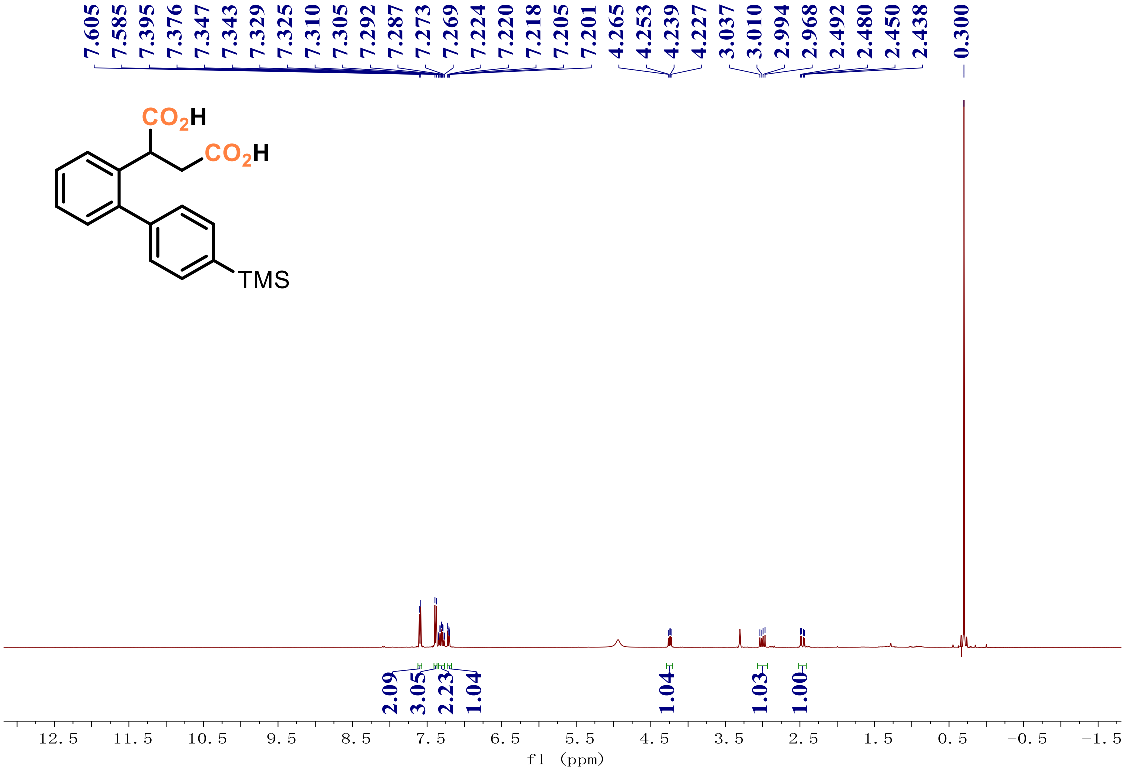

2-(4'-(trimethylsilyl)-[1,1'-biphenyl]-2-yl)succinic acid (2t)

^{13}C NMR (100 MHz, CD_3OD)

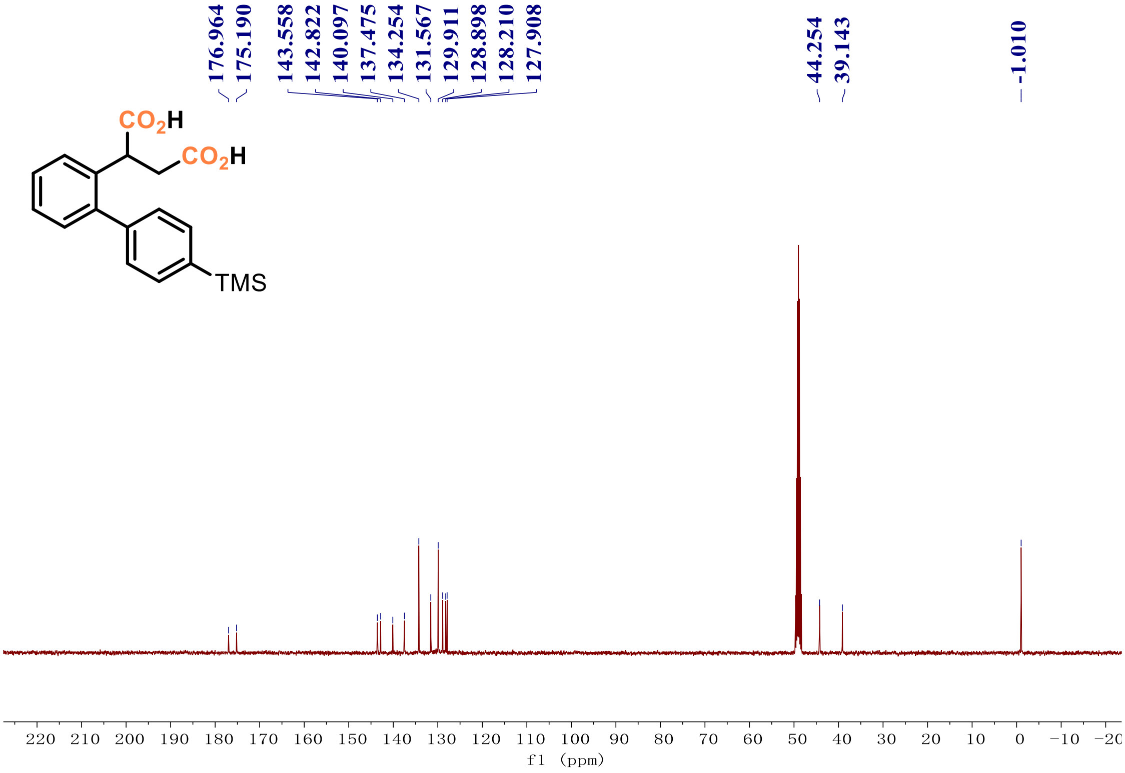

2-([1,1'-biphenyl]-4-yl)-2-methylsuccinic acid (2u)
^{1}H NMR (400 MHz, CD_3OD)

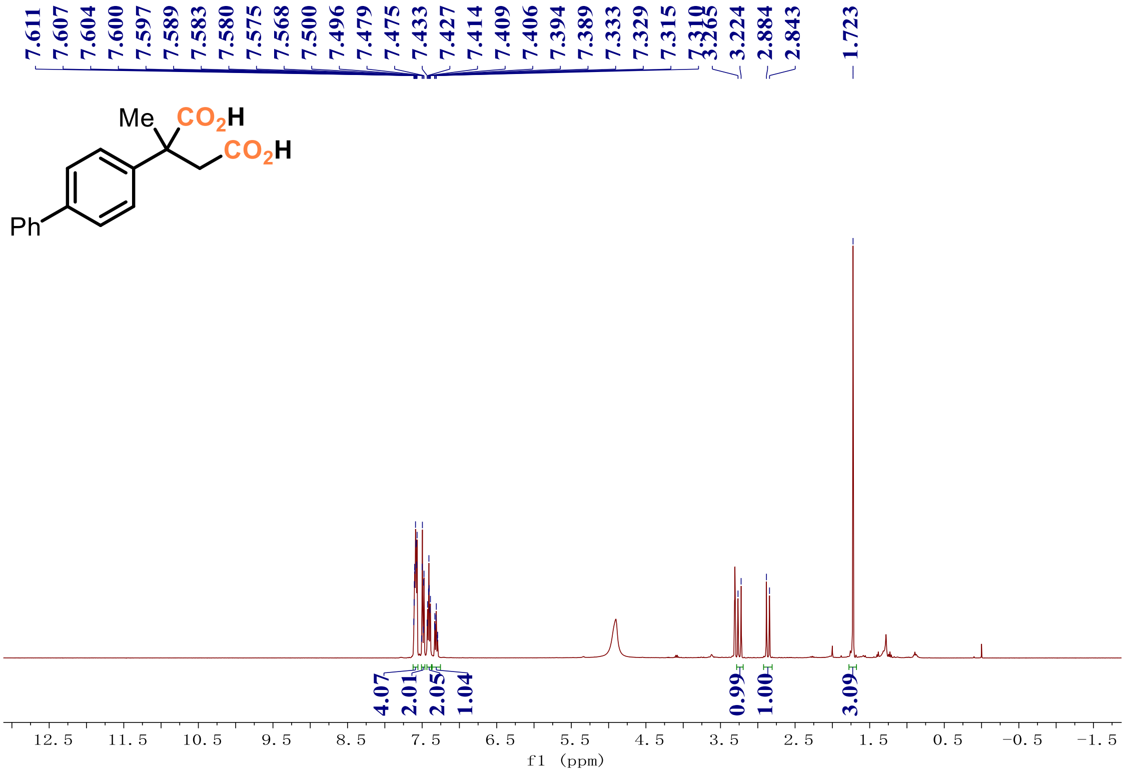

2-([1,1'-biphenyl]-4-yl)-2-methylsuccinic acid (2u)
^{13}C NMR (100 MHz, CD_3OD)

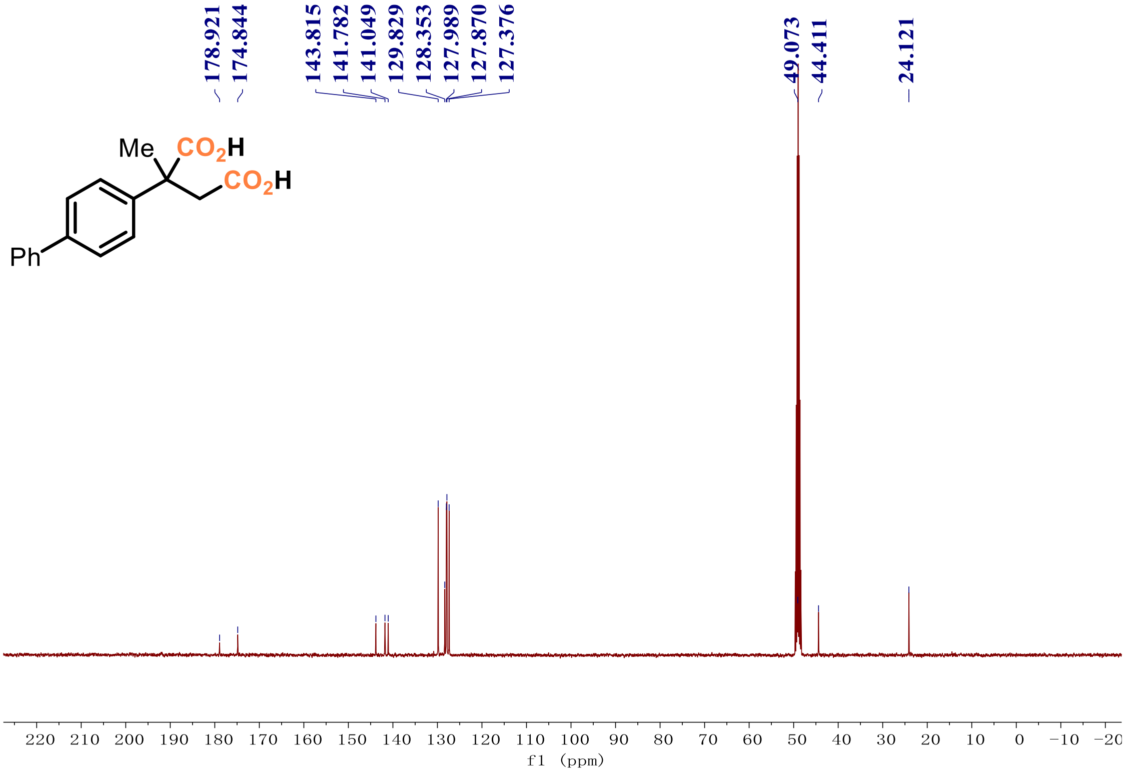

2,2-diphenylsuccinic acid (2aa)
^{1}H NMR (400 MHz, CD_3OD)

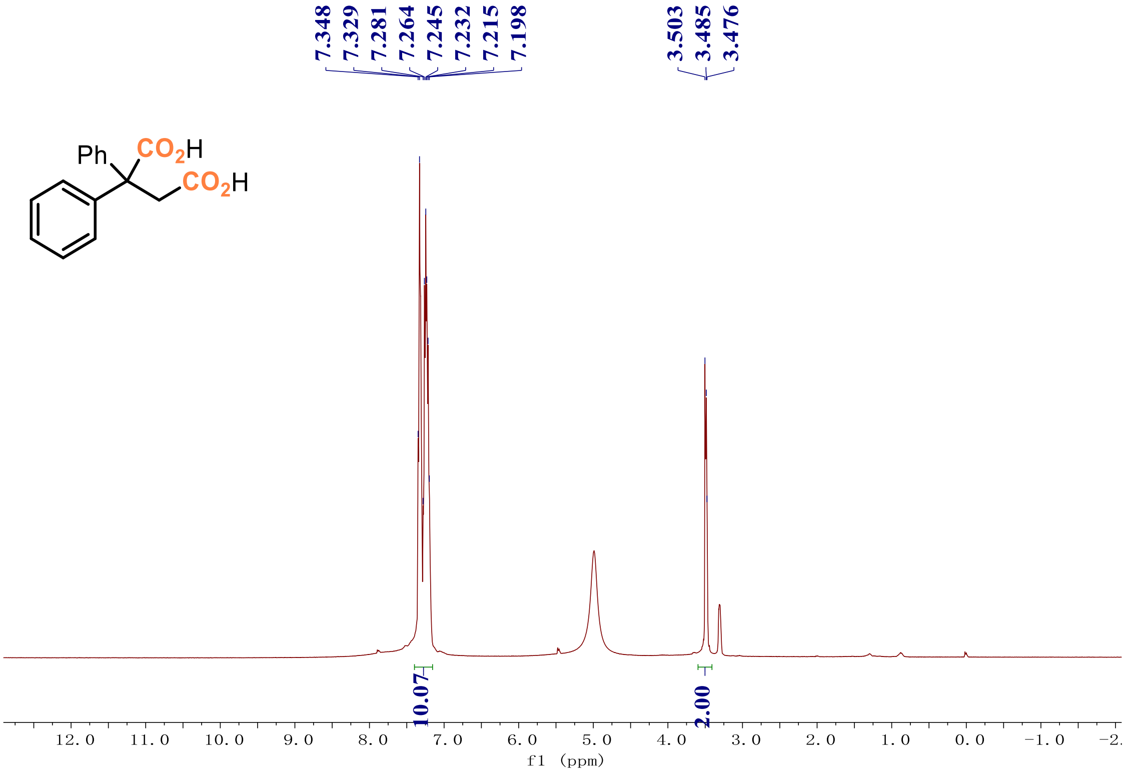

2,2-diphenylsuccinic acid (2aa)
^{13}C NMR (100 MHz, CD_3OD)

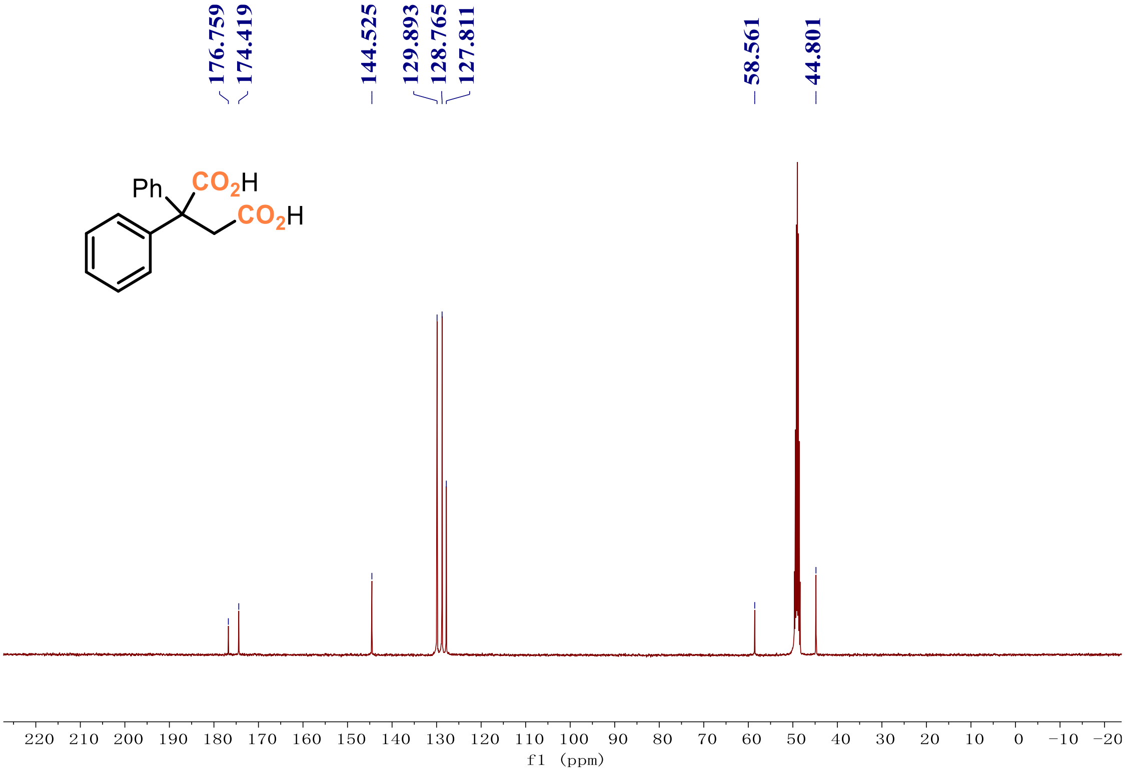

2-([1,1'-biphenyl]-4-yl)-2-phenylsuccinic acid (2ab)
^{1}H NMR (400 MHz, CD3OD)

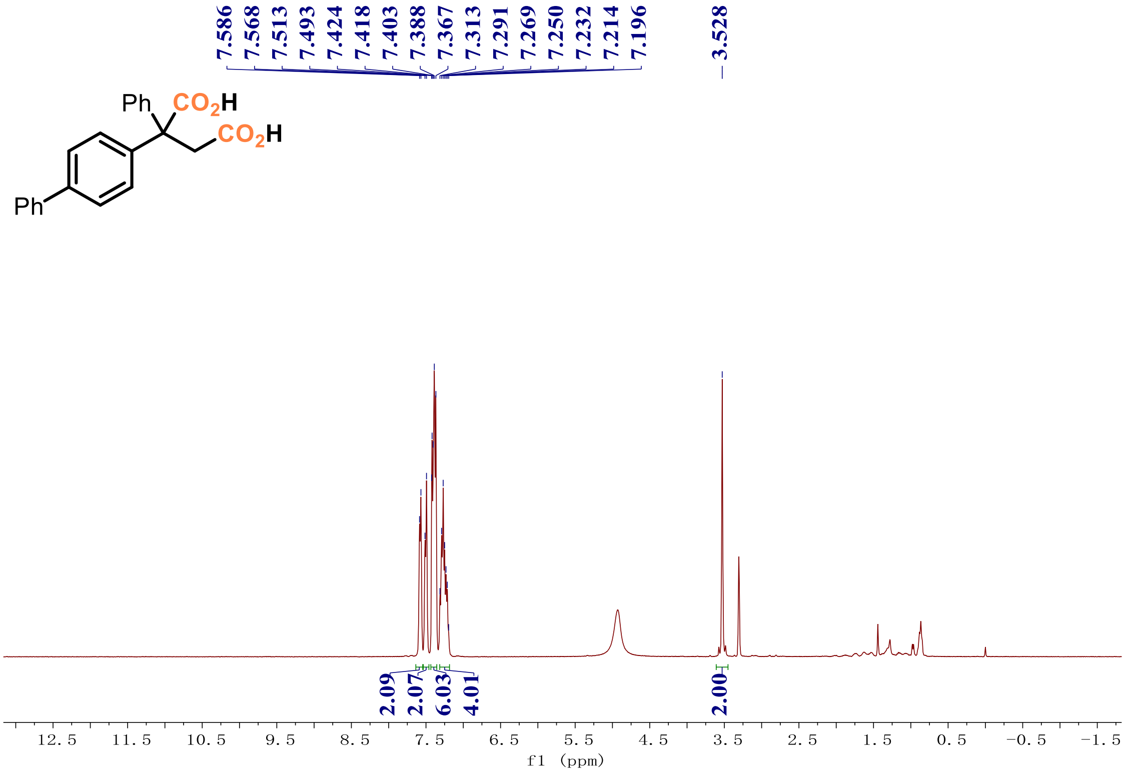

2-([1,1'-biphenyl]-4-yl)-2-phenylsuccinic acid (2ab)
^{13}C NMR (100 MHz, CD3OD)

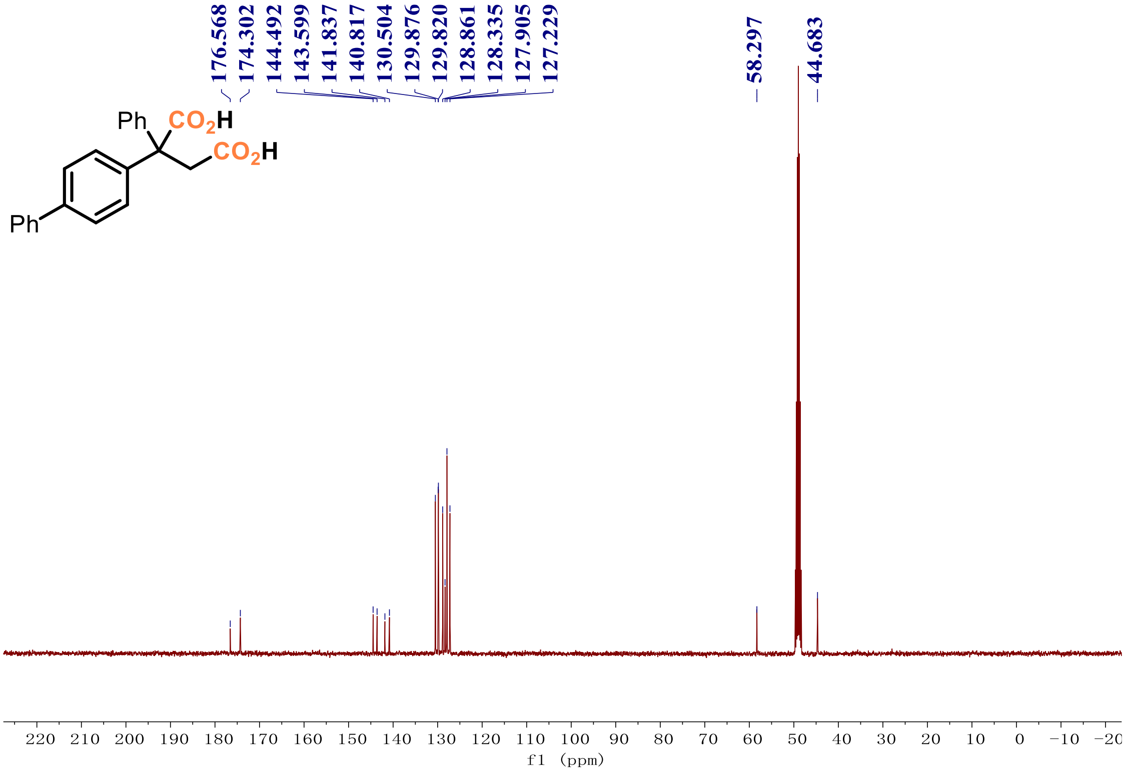

2-phenyl-2-(*o*-tolyl)succinic acid (2ac)
^{1}H NMR (400 MHz, CD_3OD)

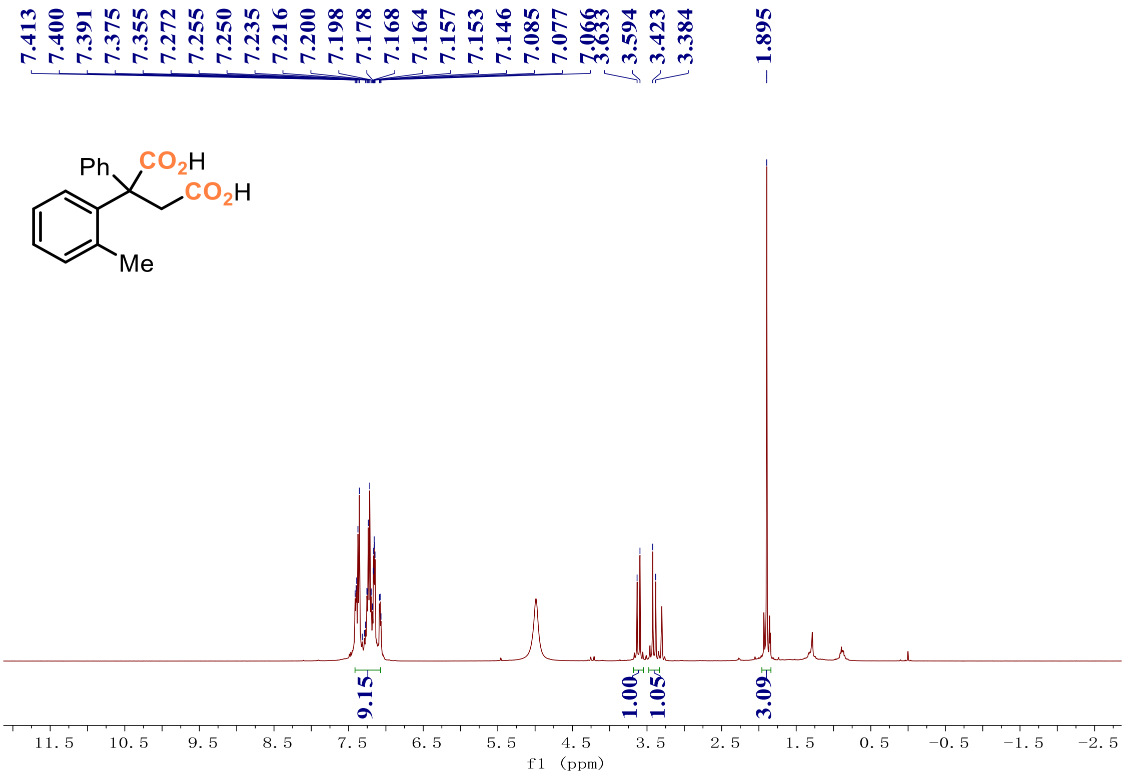

2-phenyl-2-(*o*-tolyl)succinic acid (2ac)
^{13}C NMR (100 MHz, CD_3OD)

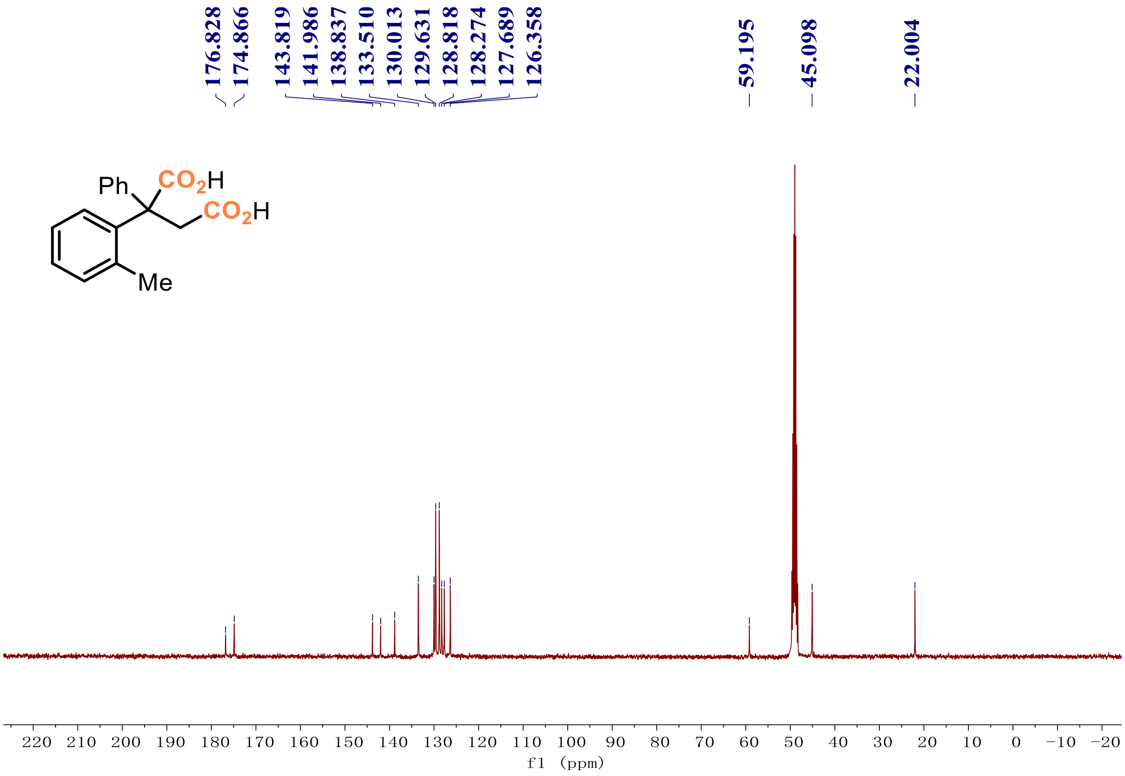

2-(4-fluorophenyl)-2-phenylsuccinic acid (2ad)
^{1}H NMR (400 MHz, CD_3OD)

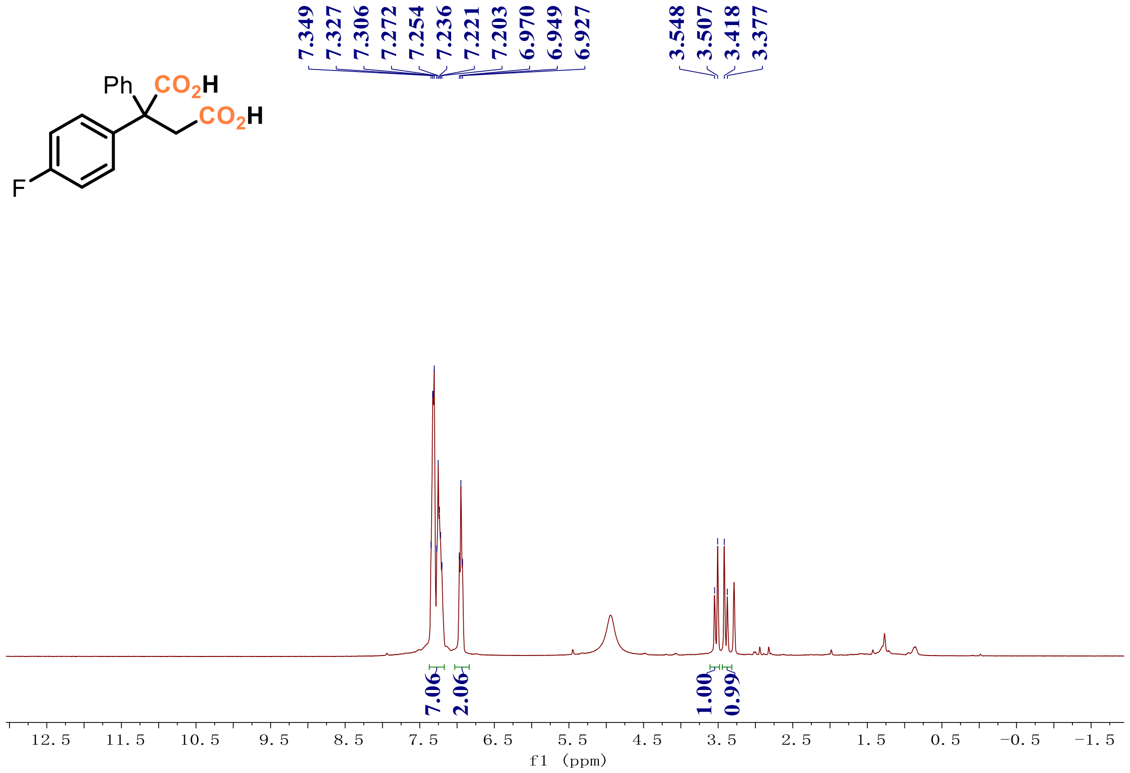

2-(4-fluorophenyl)-2-phenylsuccinic acid (2ad)
^{13}C NMR (100 MHz, CD_3OD)

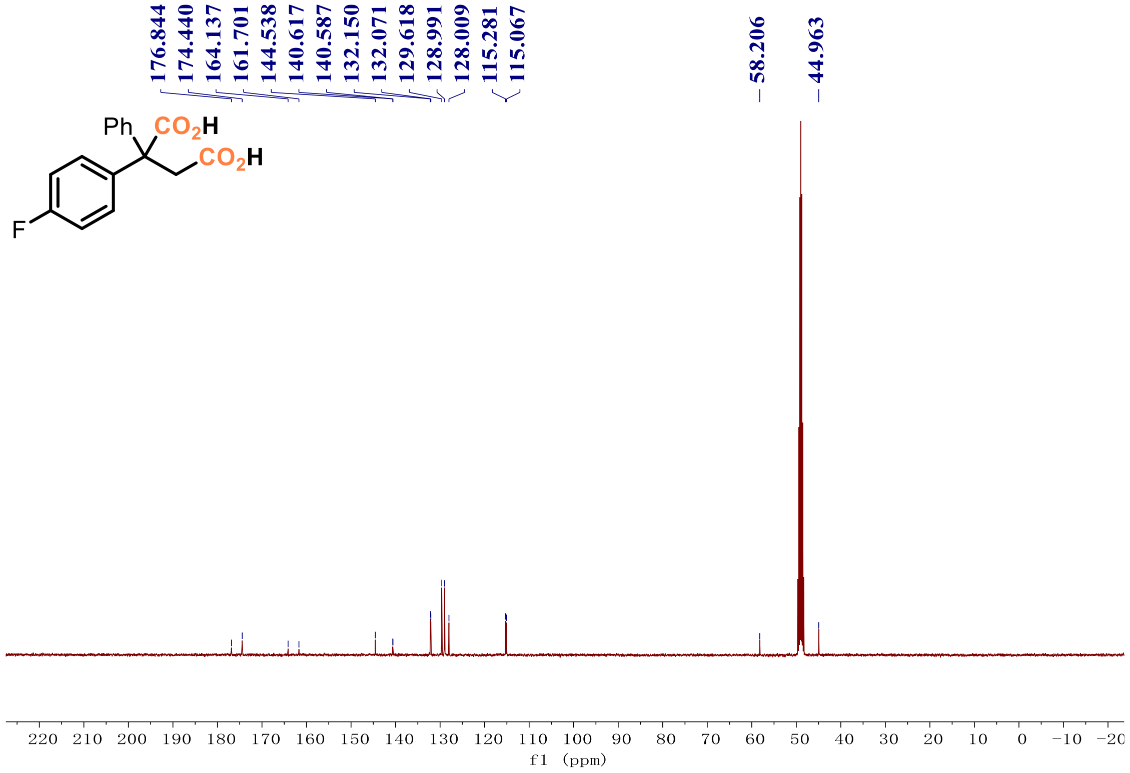

2-(4-fluorophenyl)-2-phenylsuccinic acid (2ad)
^{19}F NMR (376 MHz, CD_3OD)

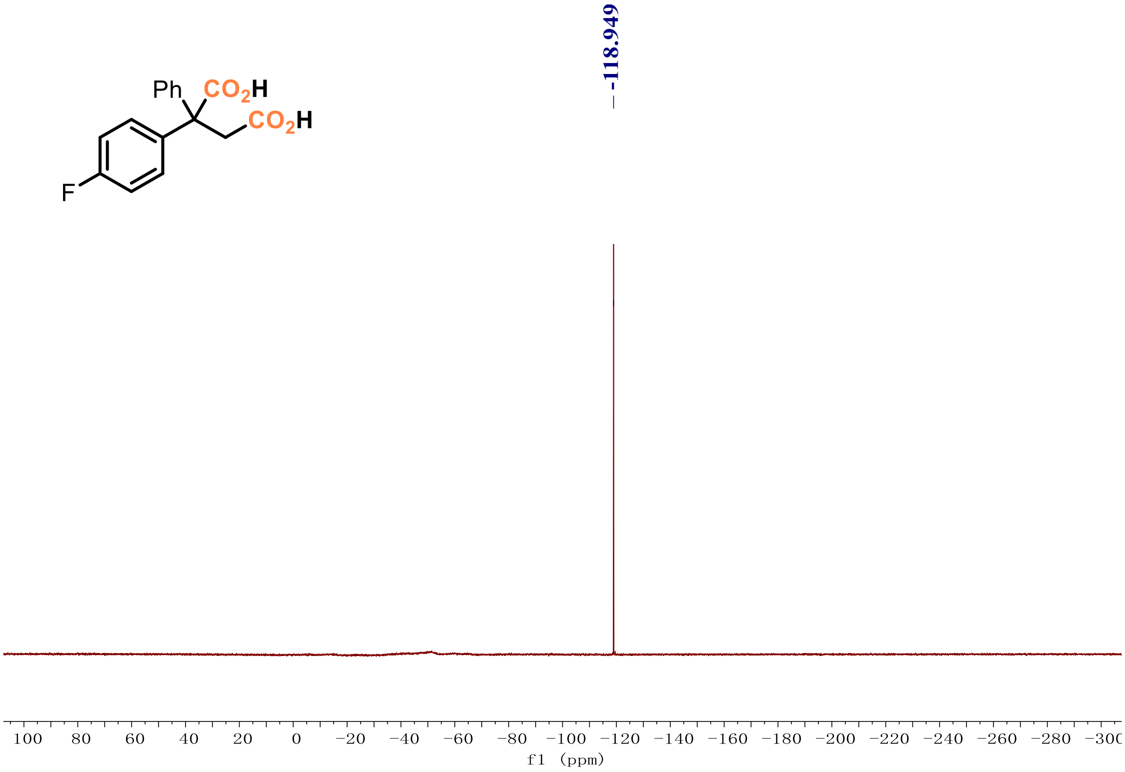

2-(3,4-difluorophenyl)-2-phenylsuccinic acid (2ae)
^{1}H NMR (400 MHz, CD_3OD)

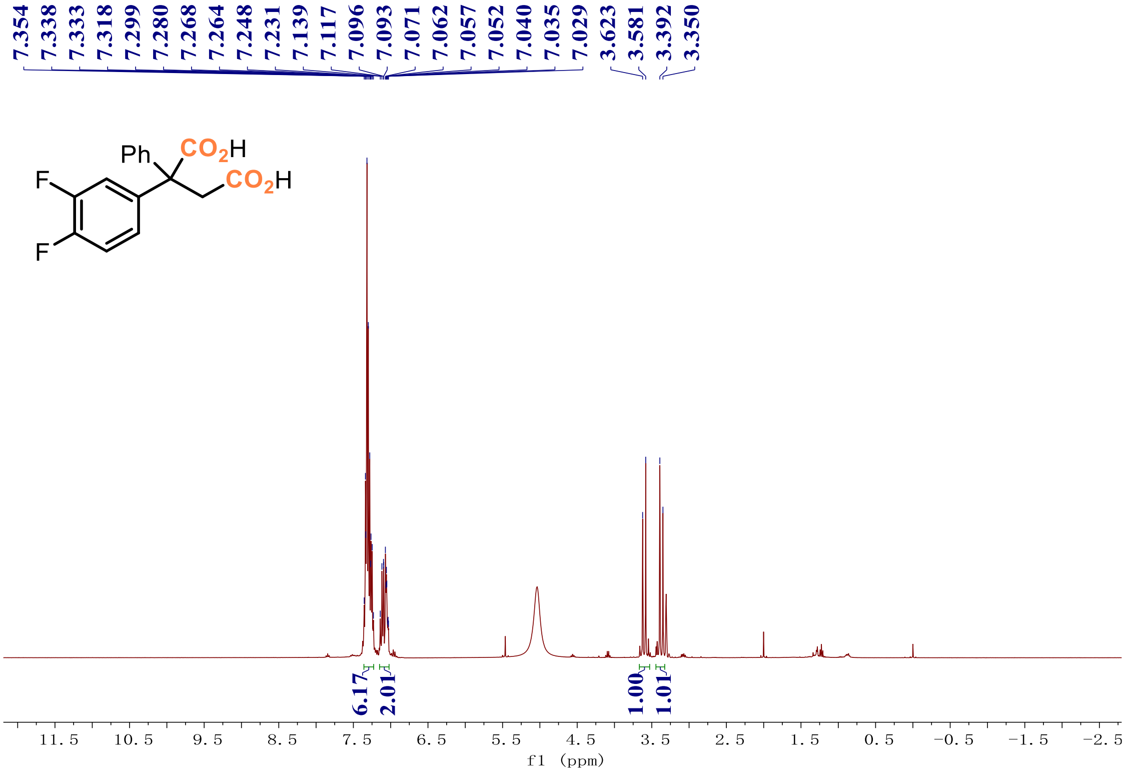

2-(3,4-difluorophenyl)-2-phenylsuccinic acid (2ae)
^{13}C NMR (100 MHz, CD3OD)

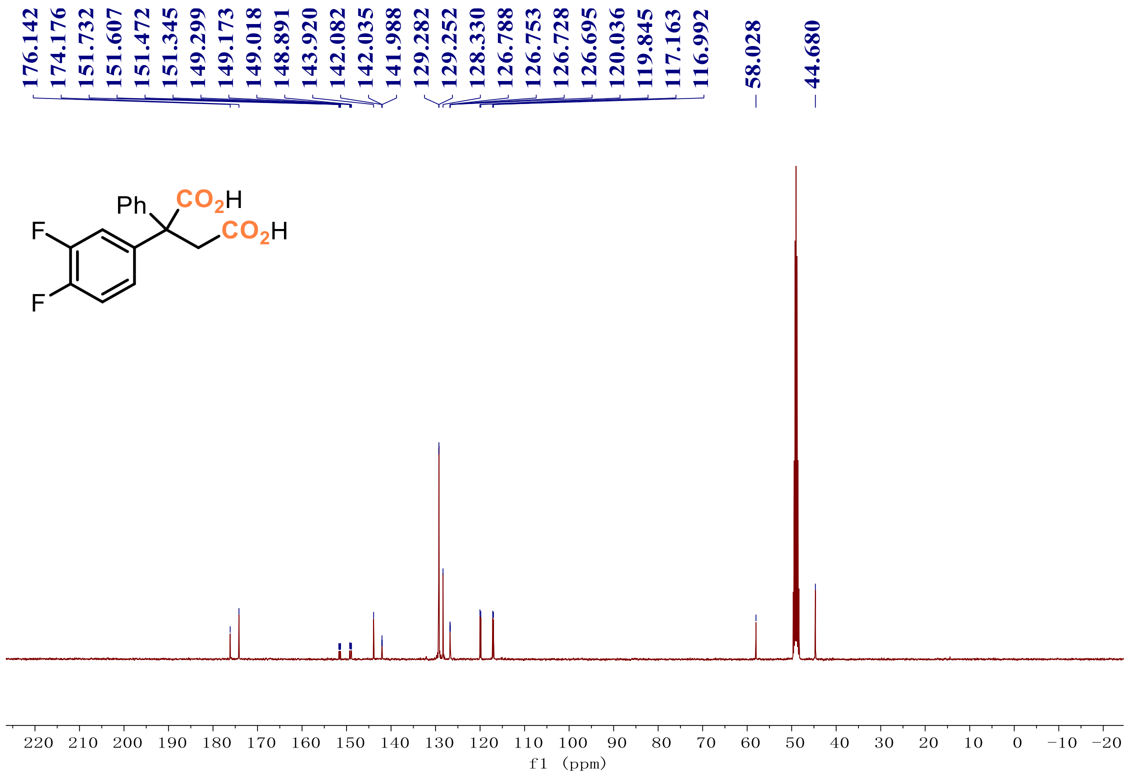

2-(3,4-difluorophenyl)-2-phenylsuccinic acid (2ae)
^{19}F NMR (376 MHz, CD3OD)

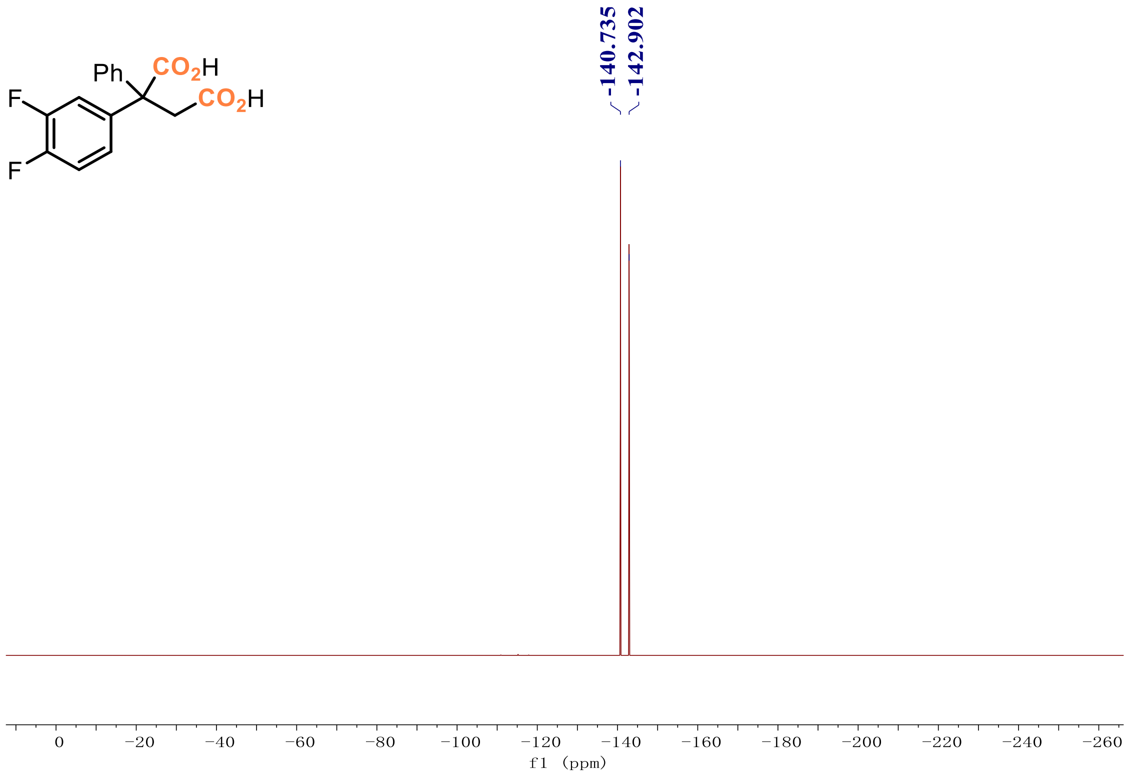

2-(naphthalen-2-yl)-2-phenylsuccinic acid (2af)
^{1}H NMR (400 MHz, CD3OD)

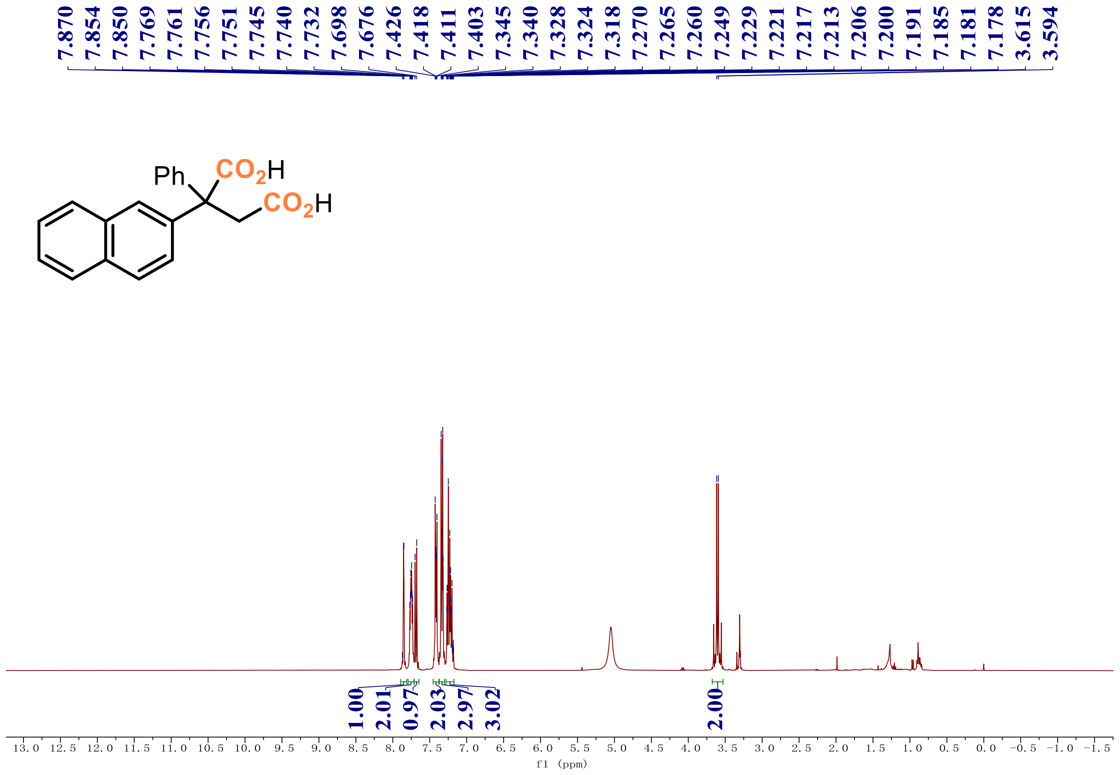

2-(naphthalen-2-yl)-2-phenylsuccinic acid (2af)
^{13}C NMR (100 MHz, CD3OD)

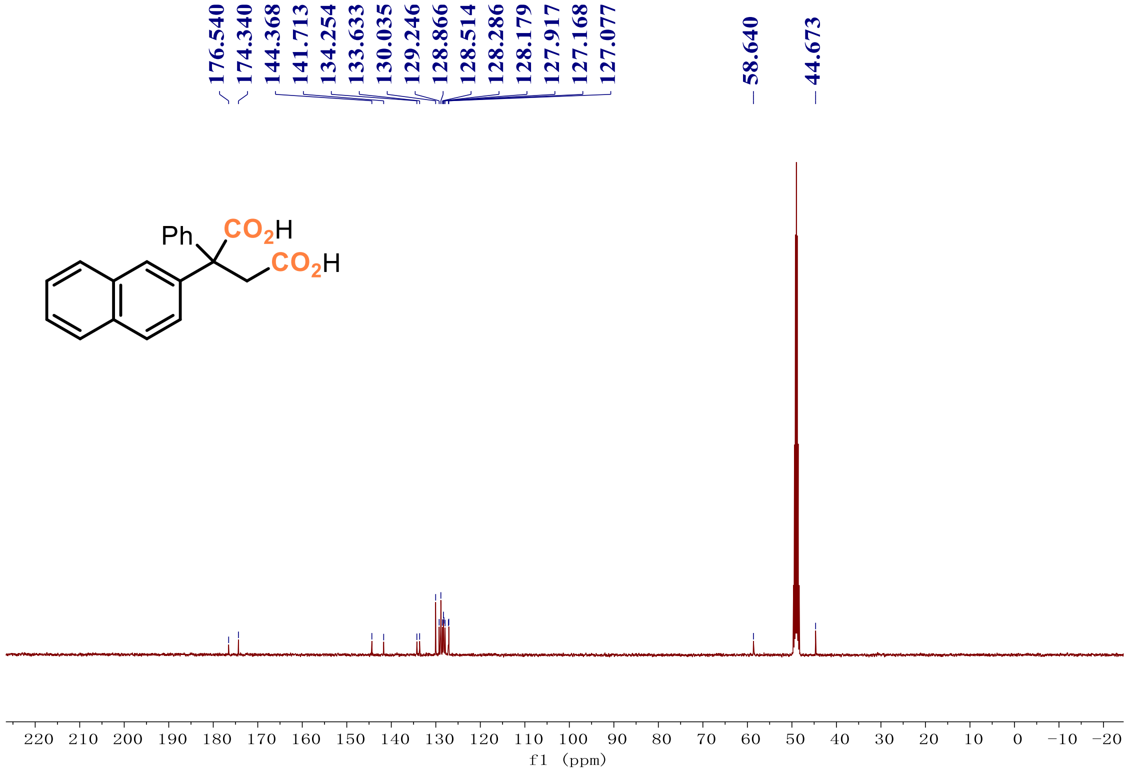

2-(dibenzo[*b*,*d*]furan-4-yl)-2-phenylsuccinic acid (2ag)

^{1}H NMR (400 MHz, CD_3OD)

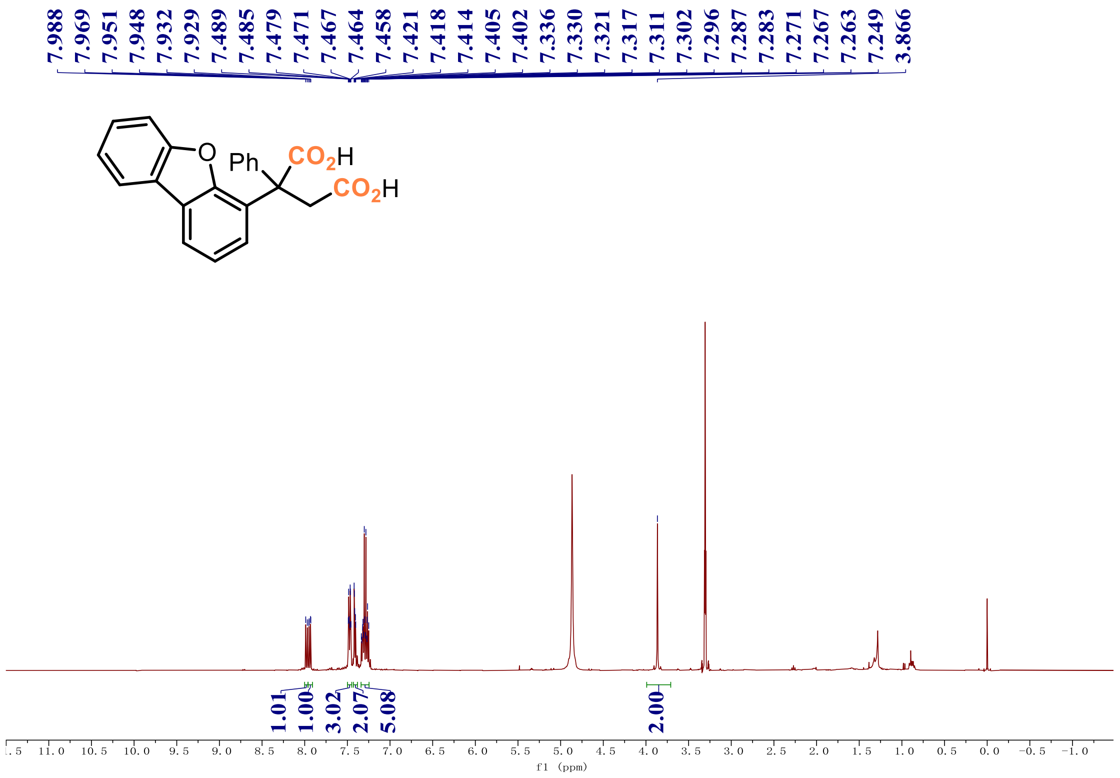

2-(dibenzo[*b*,*d*]furan-4-yl)-2-phenylsuccinic acid (2ag)

^{13}C NMR (100 MHz, CD_3OD)

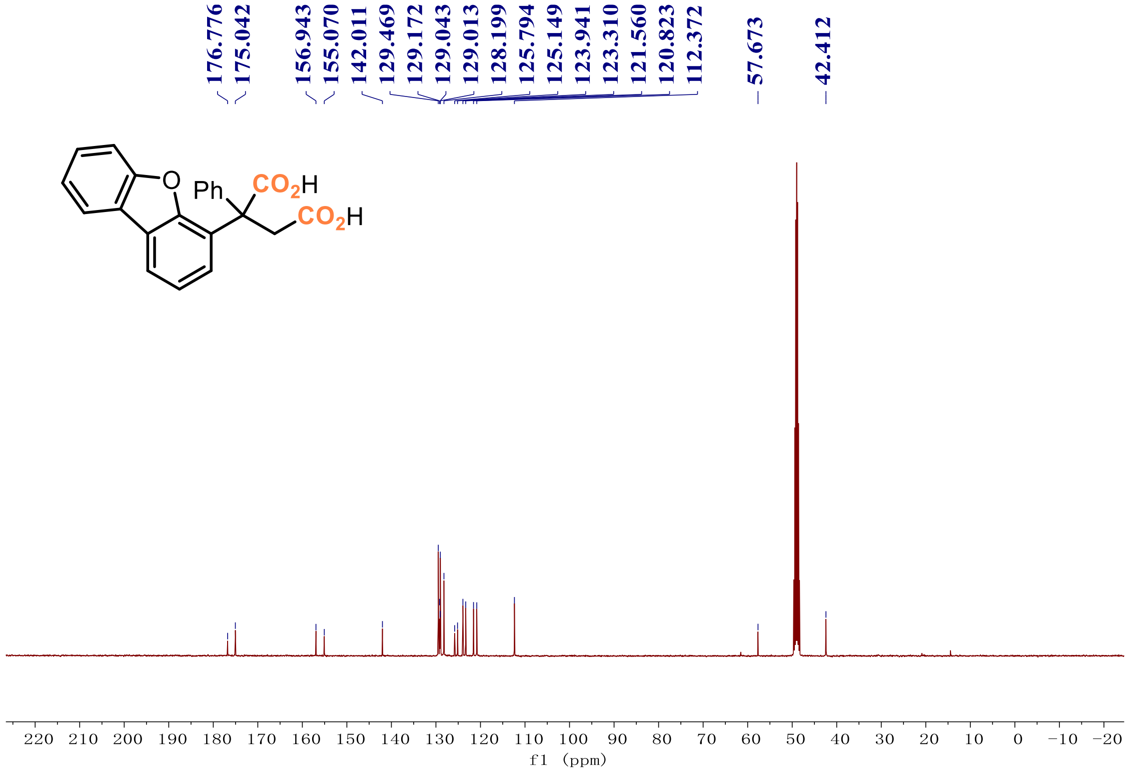

2,2-bis(4-fluorophenyl)succinic acid (2ah)

^{1}H NMR (400 MHz, CD_3OD)

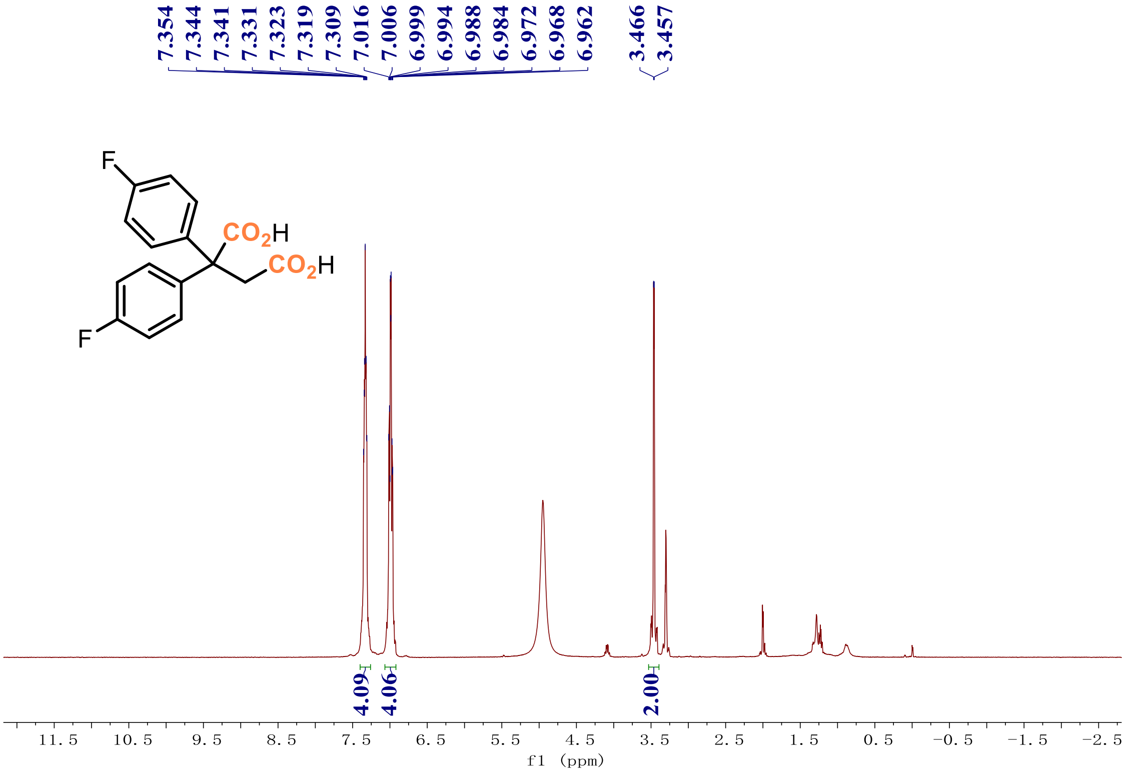

2,2-bis(4-fluorophenyl)succinic acid (2ah)

^{13}C NMR (100 MHz, CD_3OD)

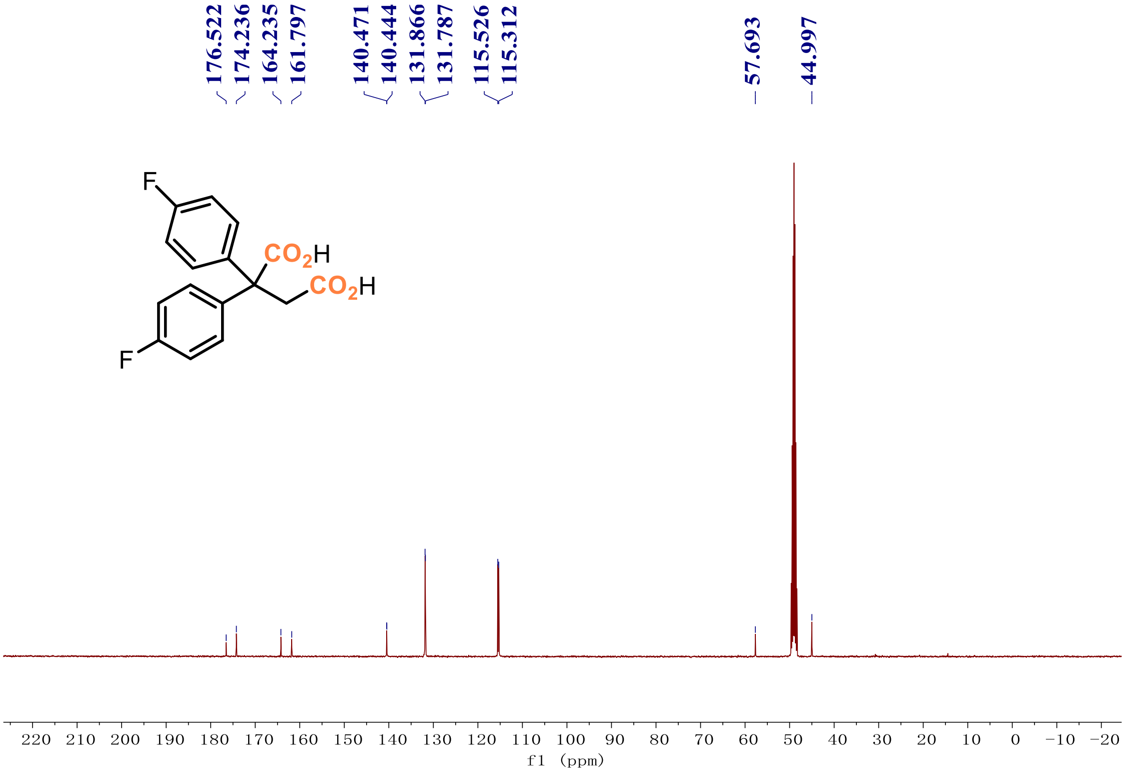

2,2-bis(4-fluorophenyl)succinic acid (2ah)
^{19}F NMR (376 MHz, CD_3OD)

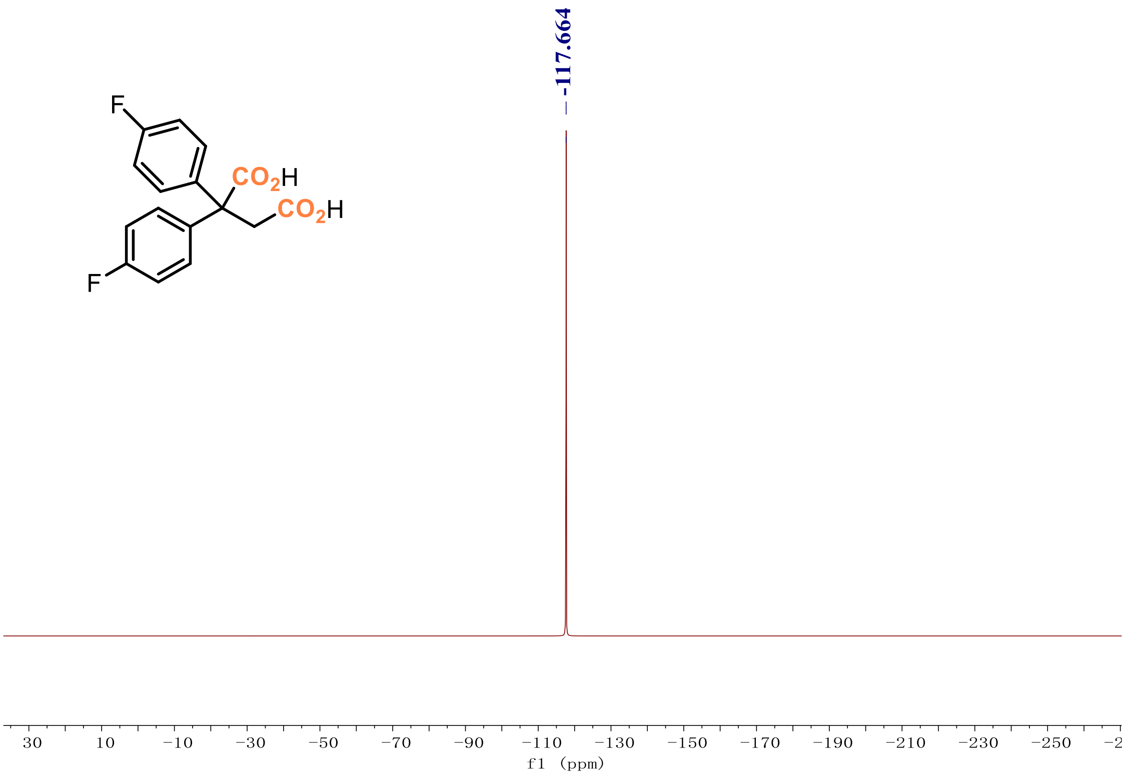

2-(4-fluorophenyl)-2-(4-methoxyphenyl)succinic acid (2ai)
^{1}H NMR (400 MHz, CD_3OD)

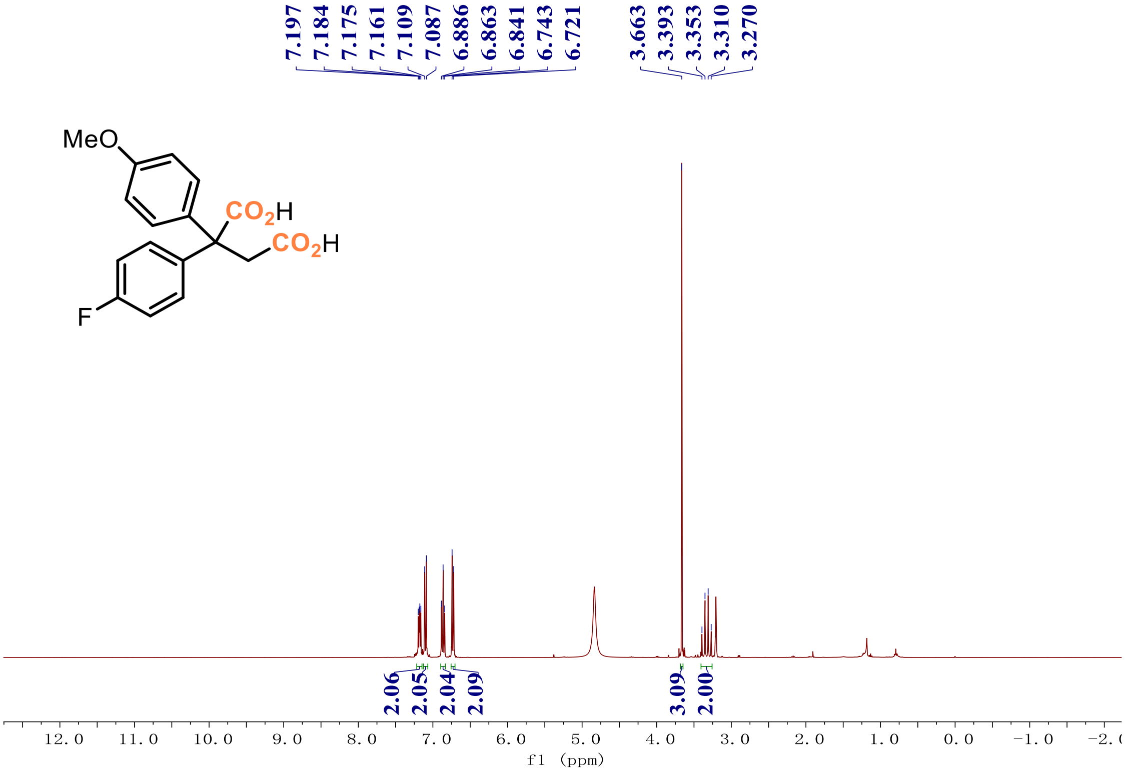

2-(4-fluorophenyl)-2-(4-methoxyphenyl)succinic acid (2ai)
^{13}C NMR (100 MHz, CD_3OD)

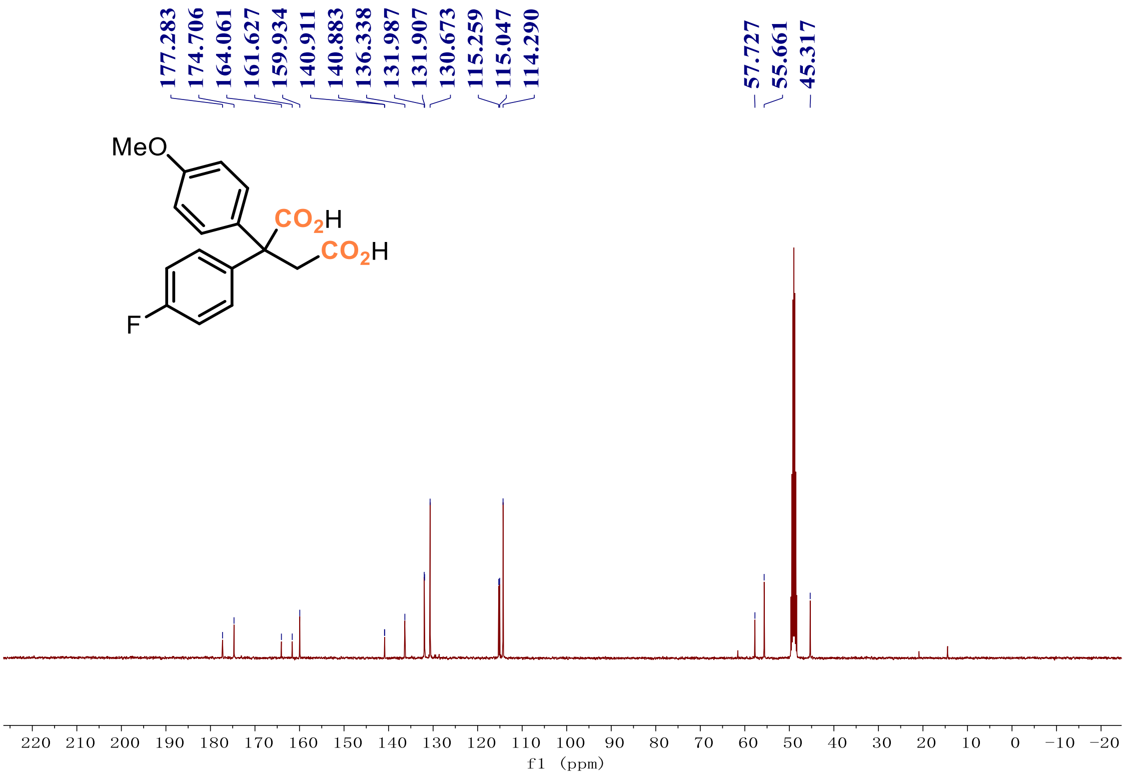

2-(4-fluorophenyl)-2-(4-methoxyphenyl)succinic acid (2ai)
^{19}F NMR (376 MHz, CD_3OD)

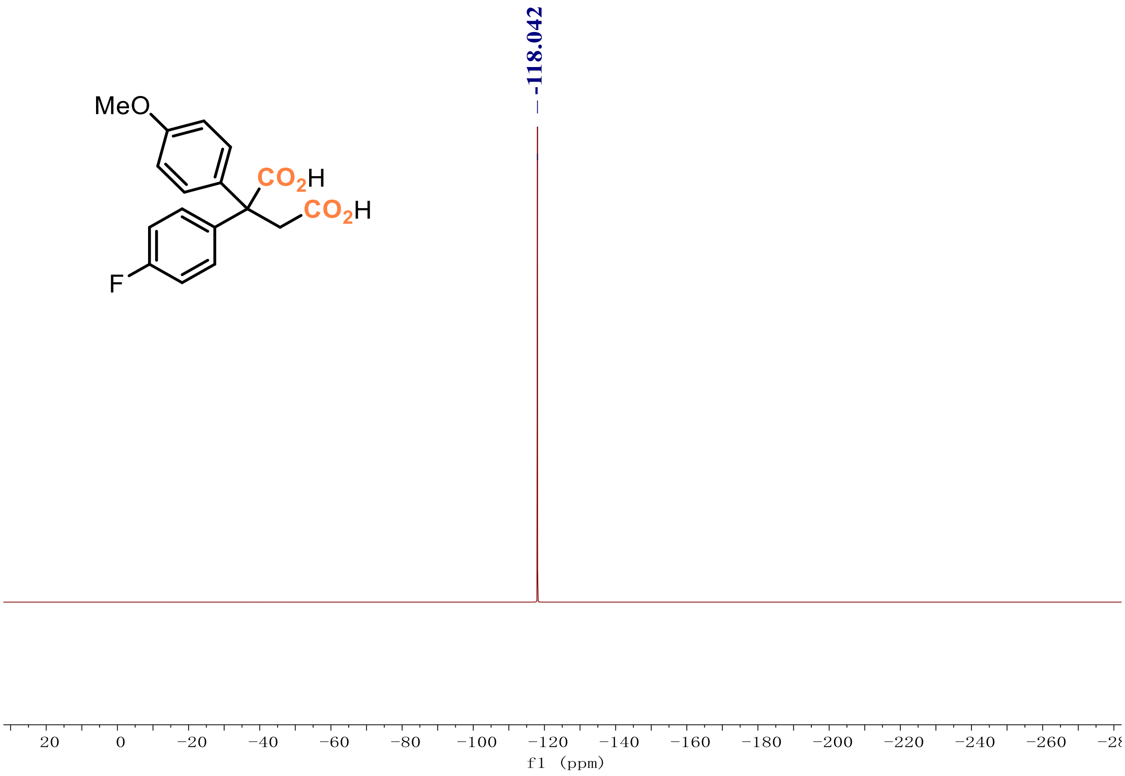

2-([1,1'-biphenyl]-4-yl)-3-methylsuccinic acid (2ba)
^{1}H NMR (400 MHz, CD_3OD)

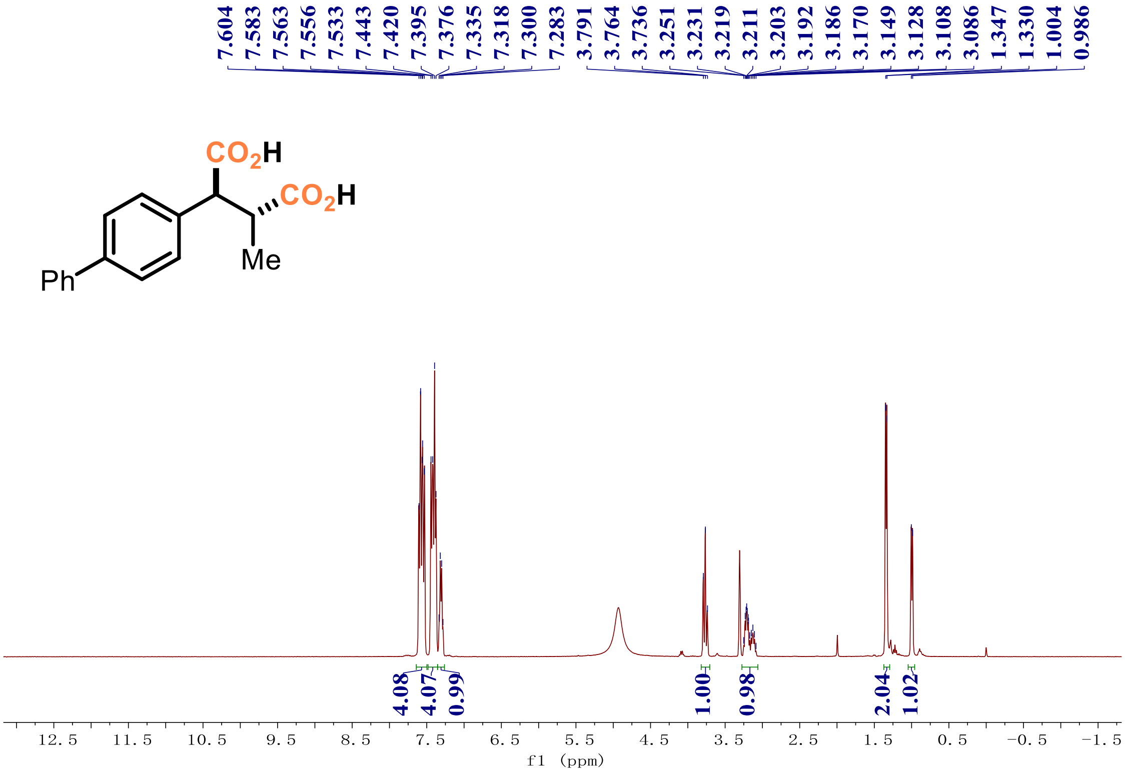

2-([1,1'-biphenyl]-4-yl)-3-methylsuccinic acid (2ba)
^{13}C NMR (100 MHz, CD_3OD)

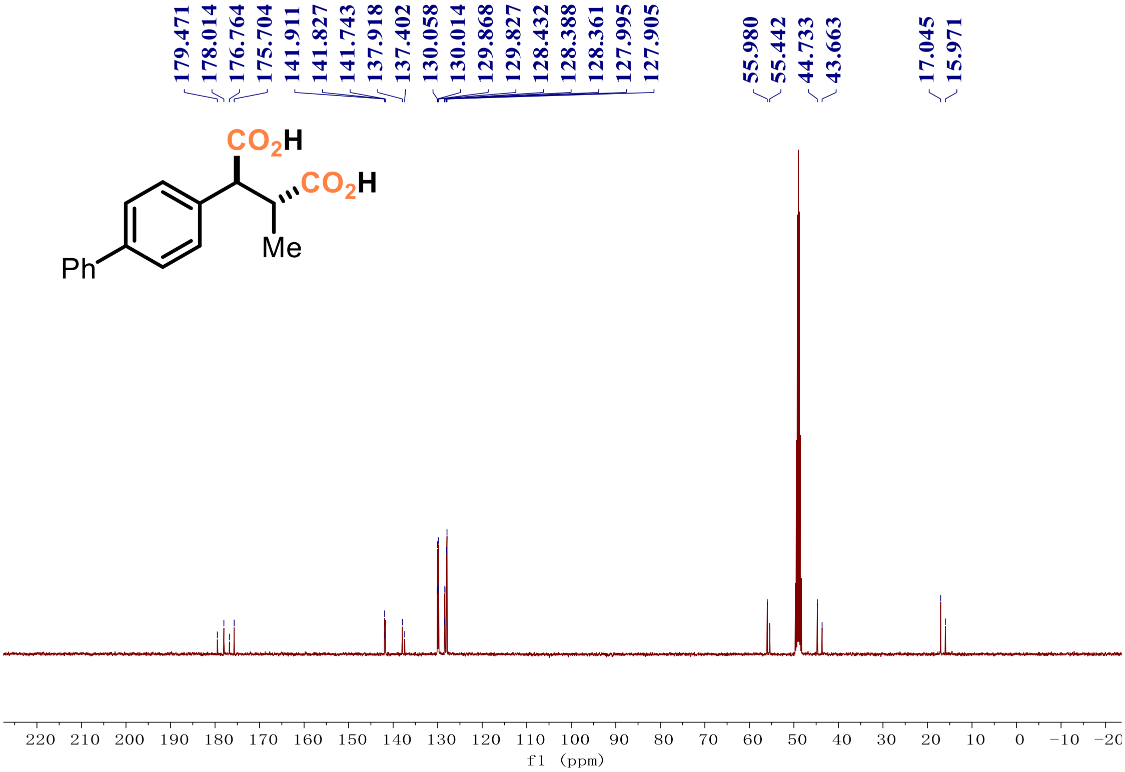

1,2,3,4-tetrahydronaphthalene-1,2-dicarboxylic acid (2bb)
^{1}H NMR (400 MHz, CD_3OD)

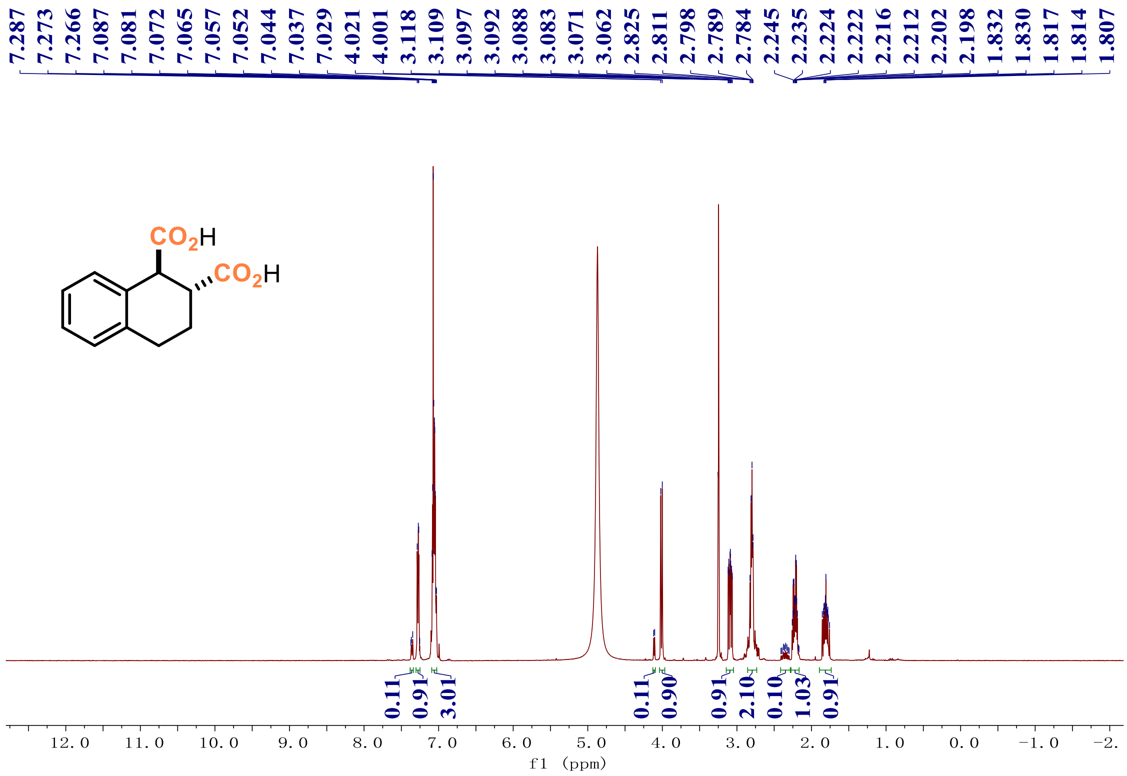

1,2,3,4-tetrahydronaphthalene-1,2-dicarboxylic acid (2bb)
^{13}C NMR (100 MHz, CD_3OD)

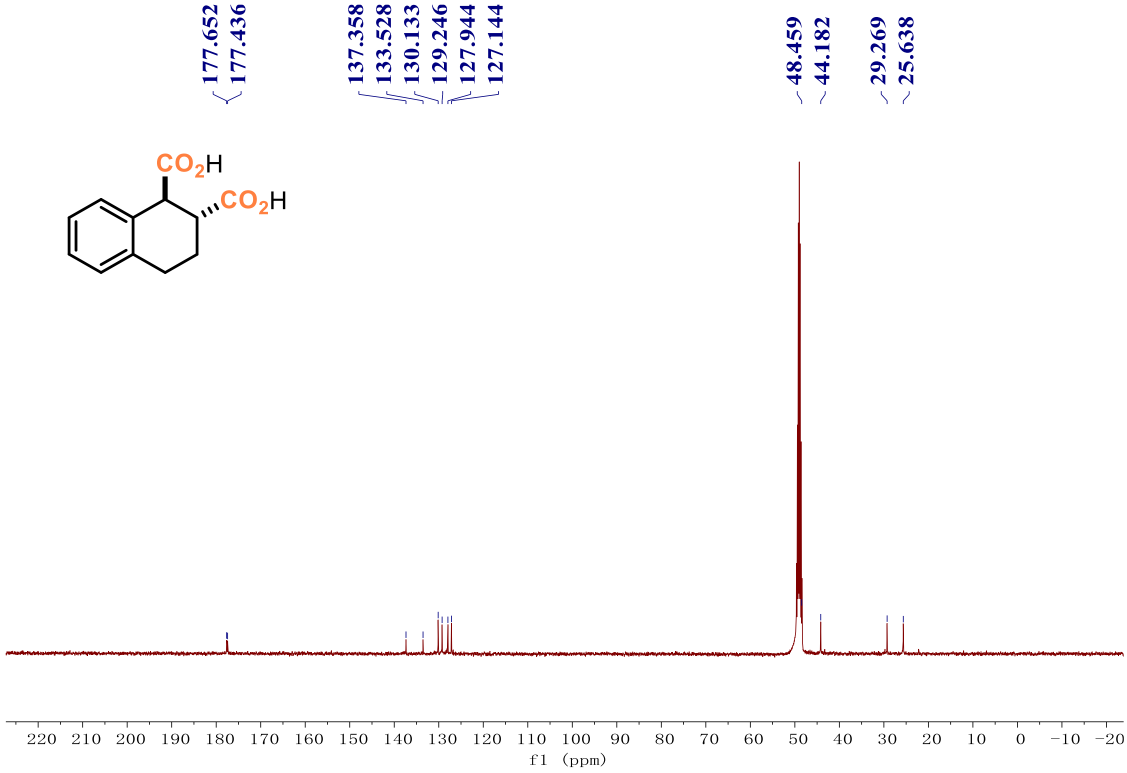

3-methyl-2,2-diphenylsuccinic acid (2bc)

^{1}H NMR (400 MHz, CD_3OD)

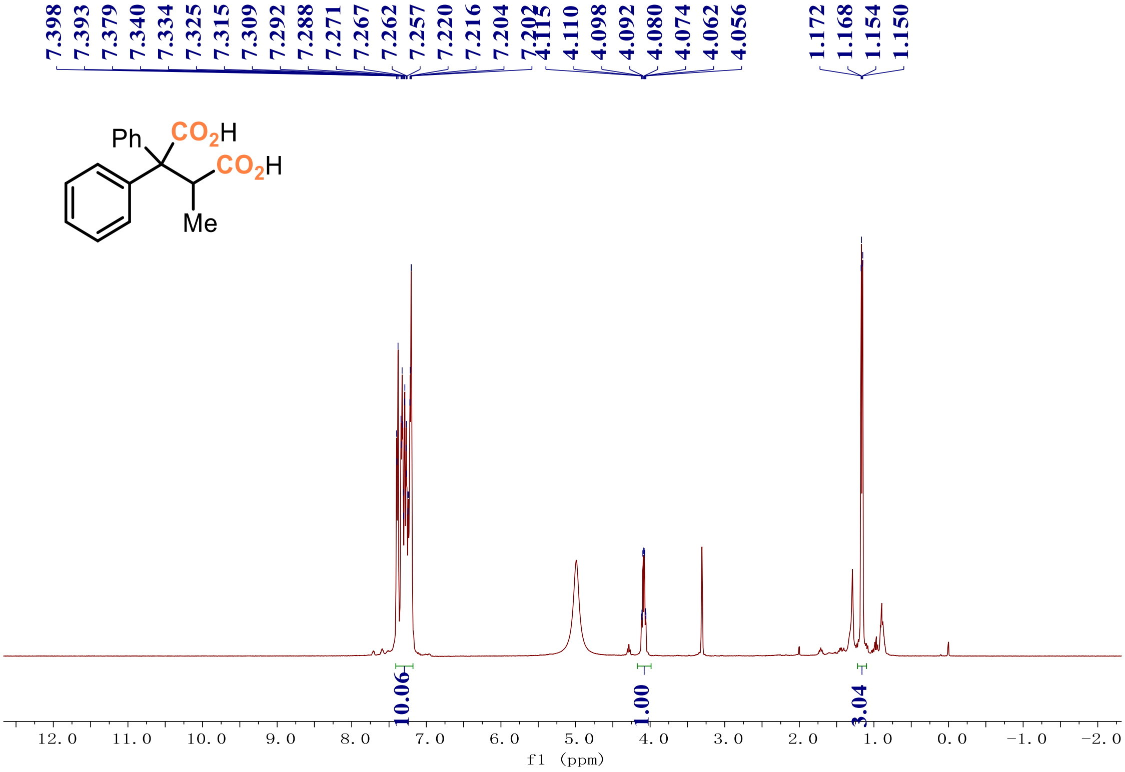

3-methyl-2,2-diphenylsuccinic acid (2bc)

^{13}C NMR (100 MHz, CD_3OD)

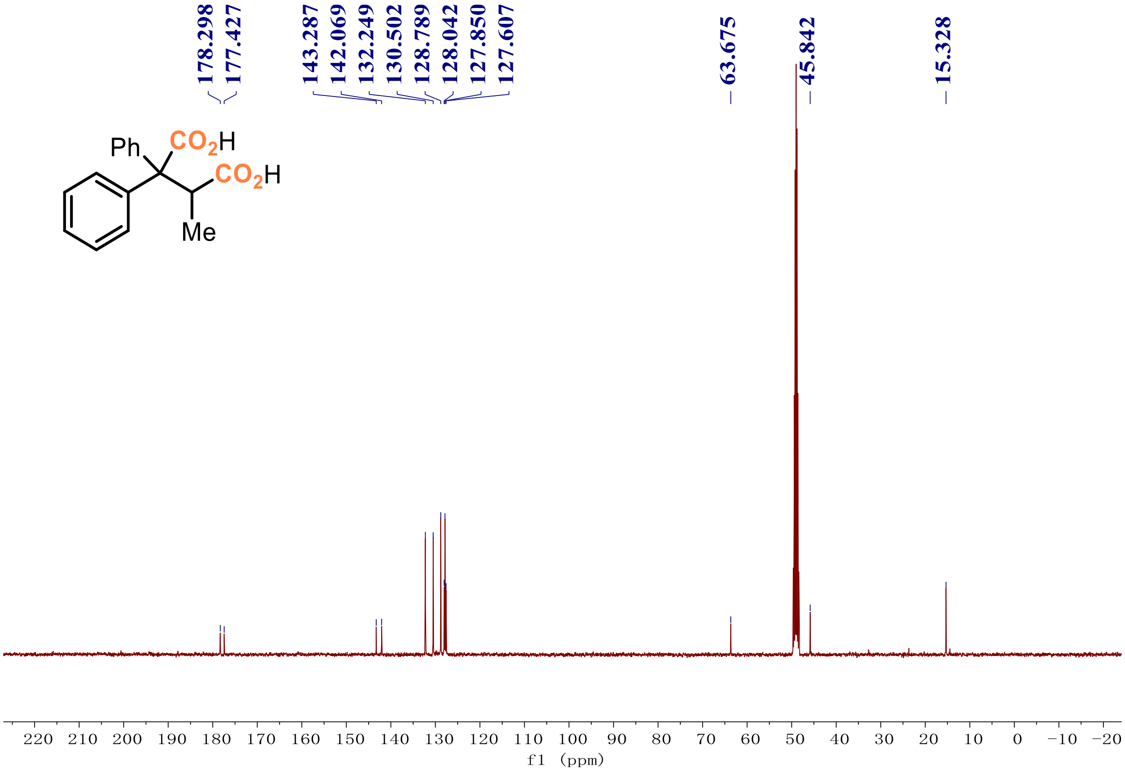

3-methyl-2-phenyl-2-(*m*-tolyl)succinic acid (2bd)
^{1}H NMR (400 MHz, CD_3OD)

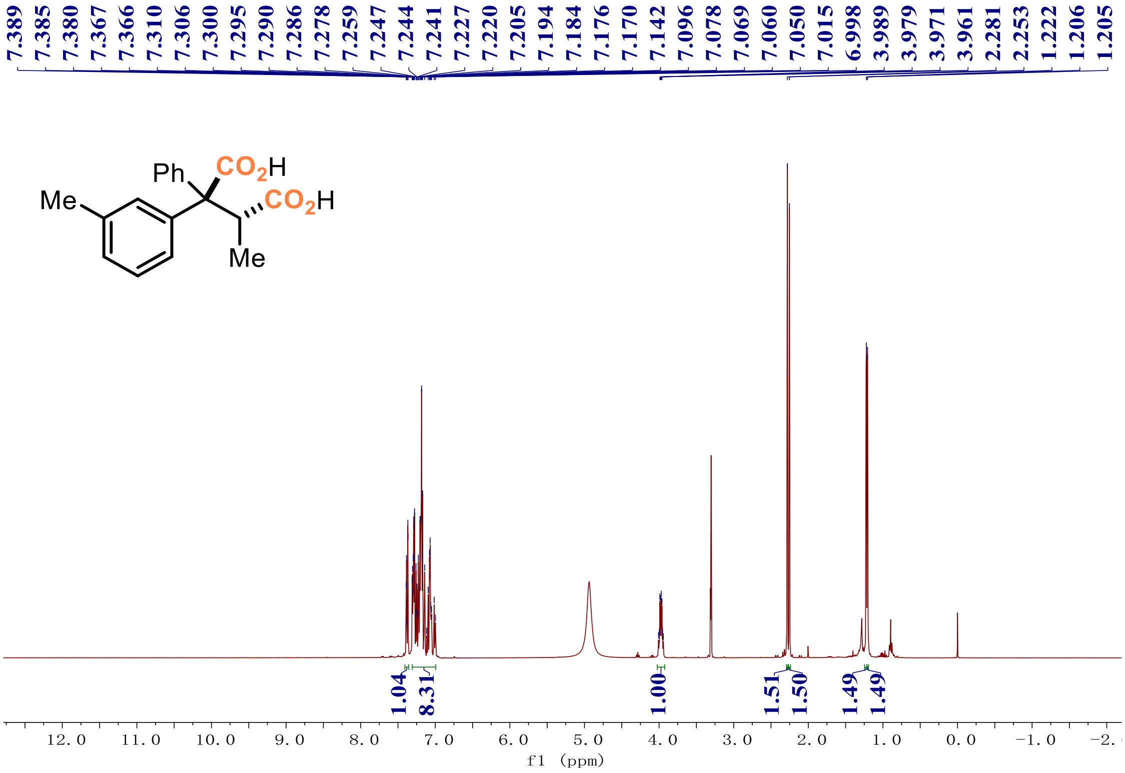

3-methyl-2-phenyl-2-(*m*-tolyl)succinic acid (2bd)
^{13}C NMR (100 MHz, CD_3OD)

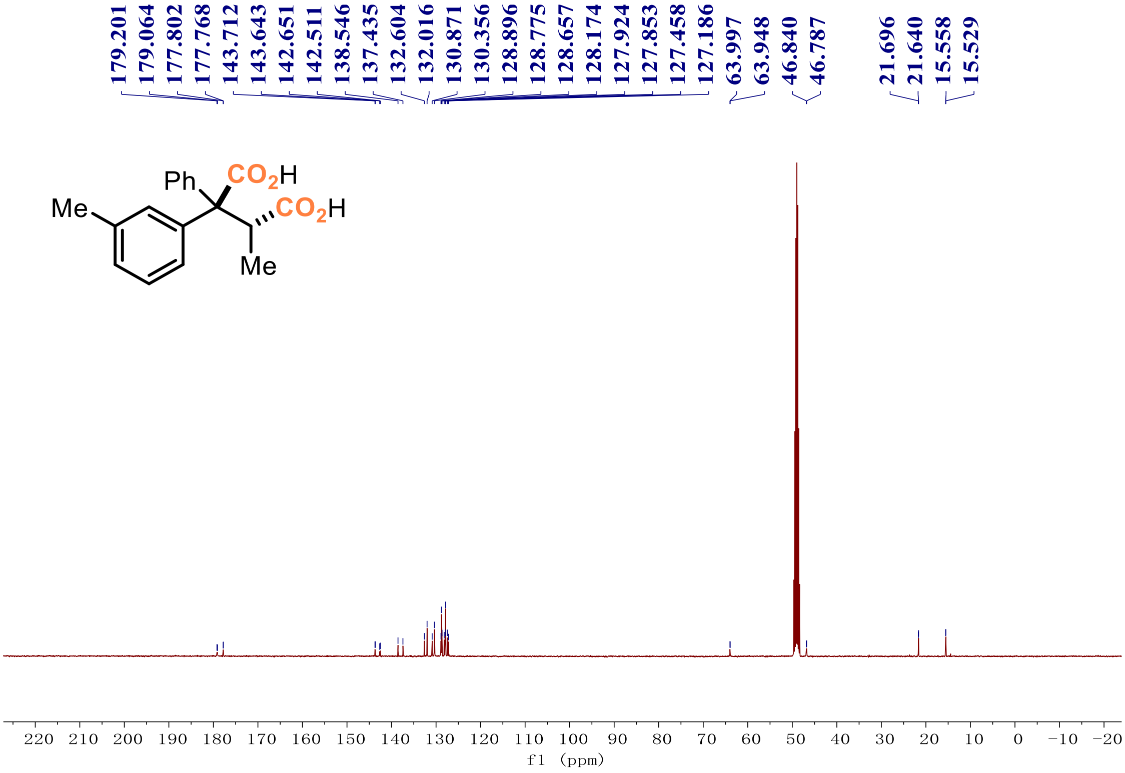

2,2,3-triphenylsuccinic acid (2be)
^{1}H NMR (400 MHz, CD_3OD)

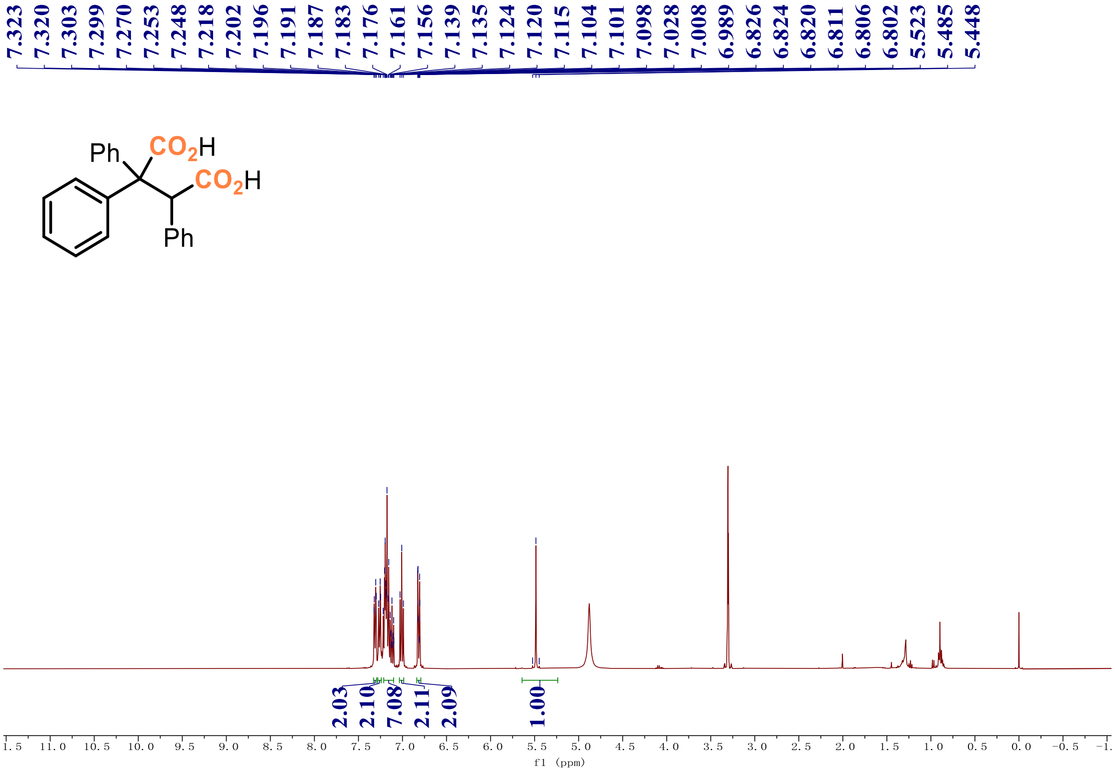

2,2,3-triphenylsuccinic acid (2be)
^{13}C NMR (100 MHz, CD_3OD)

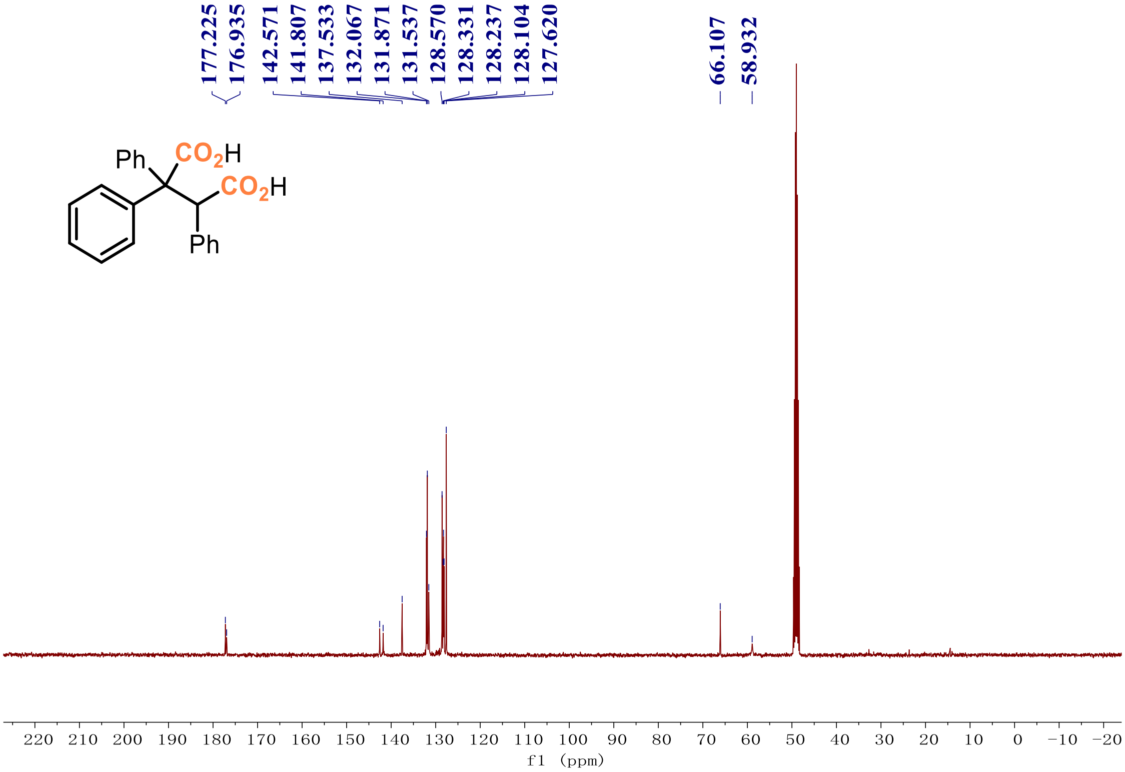

2,2-bis(4-fluorophenyl)-3-methylsuccinic acid (2bf)

^{1}H NMR (400 MHz, CD_3OD)

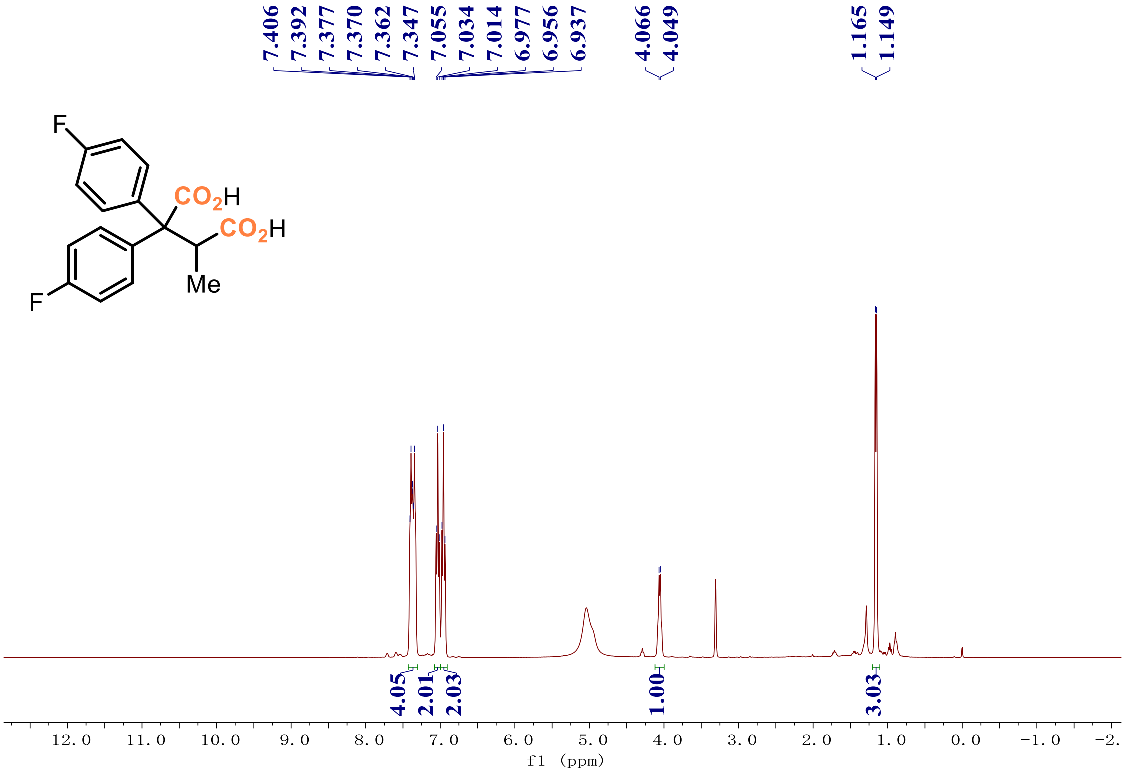

2,2-bis(4-fluorophenyl)-3-methylsuccinic acid (2bf)

^{13}C NMR (100 MHz, CD_3OD)

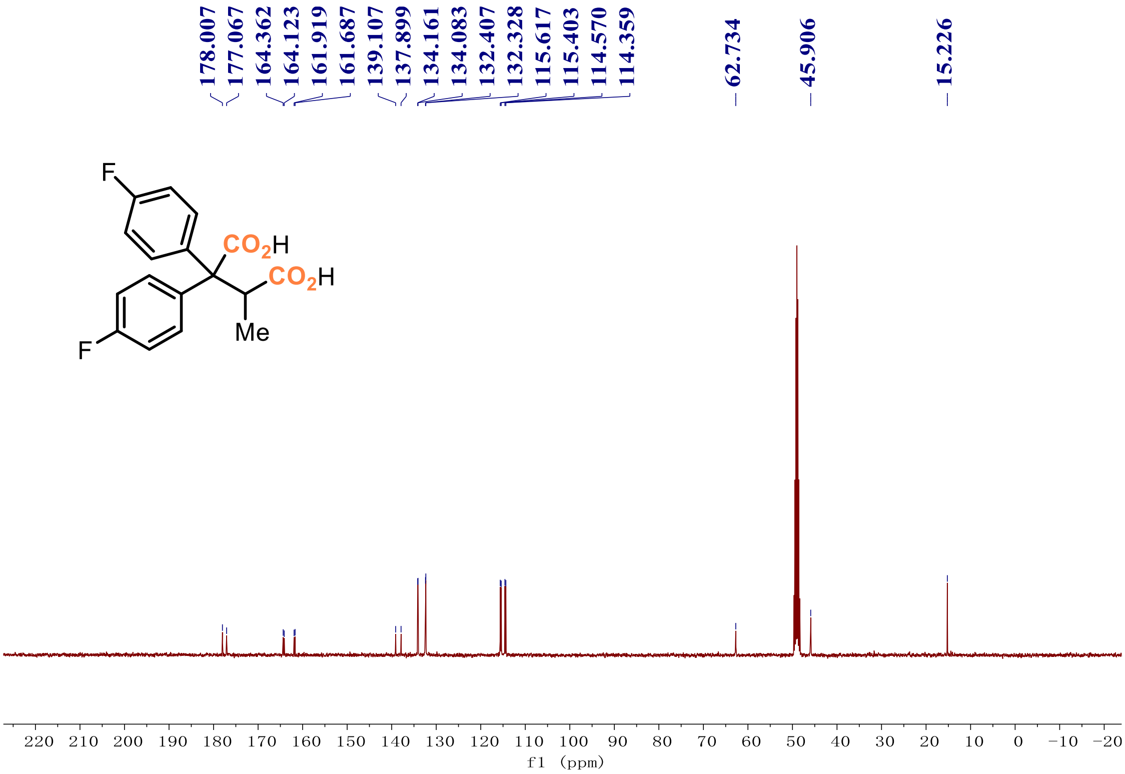

2,2-bis(4-fluorophenyl)-3-methylsuccinic acid (2bf)

^{19}F NMR (376 MHz, CD_3OD)

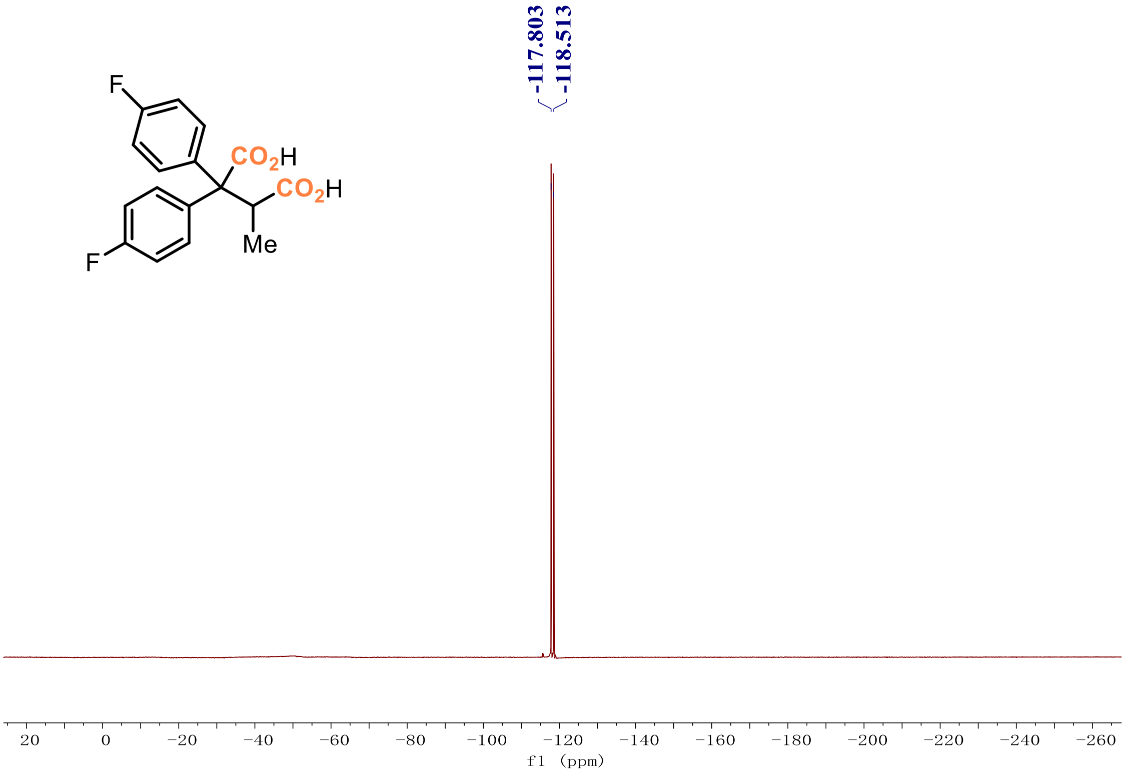

2-methyl-2-(phenethoxycarbonyl)succinic acid (2ca)

^{1}H NMR (400 MHz, CD_3OD)

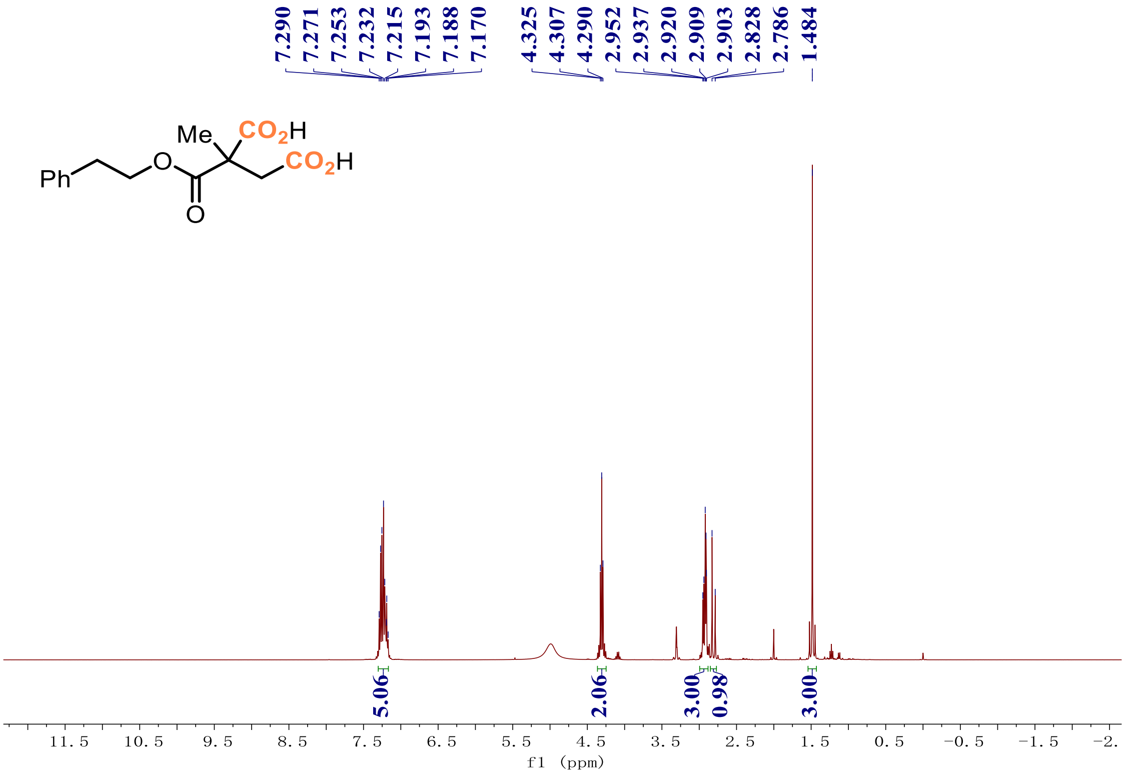

2-methyl-2-(phenethoxycarbonyl)succinic acid (2ca)

^{13}C NMR (100 MHz, CD_3OD)

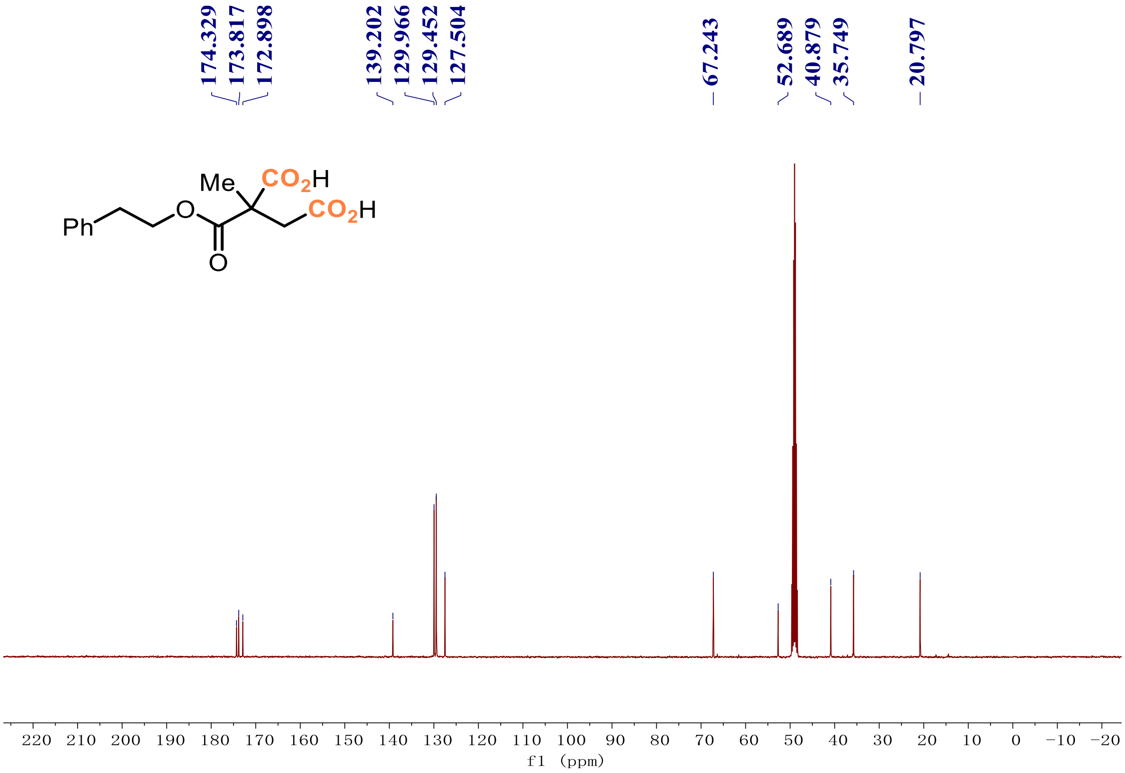

2-((((1*S*,2*R*,5*S*)-2-isopropyl-5-methylcyclohexyl)oxy)carbonyl)-2-methylsuccinic acid (2cb)

^{1}H NMR (400 MHz, CD_3OD)

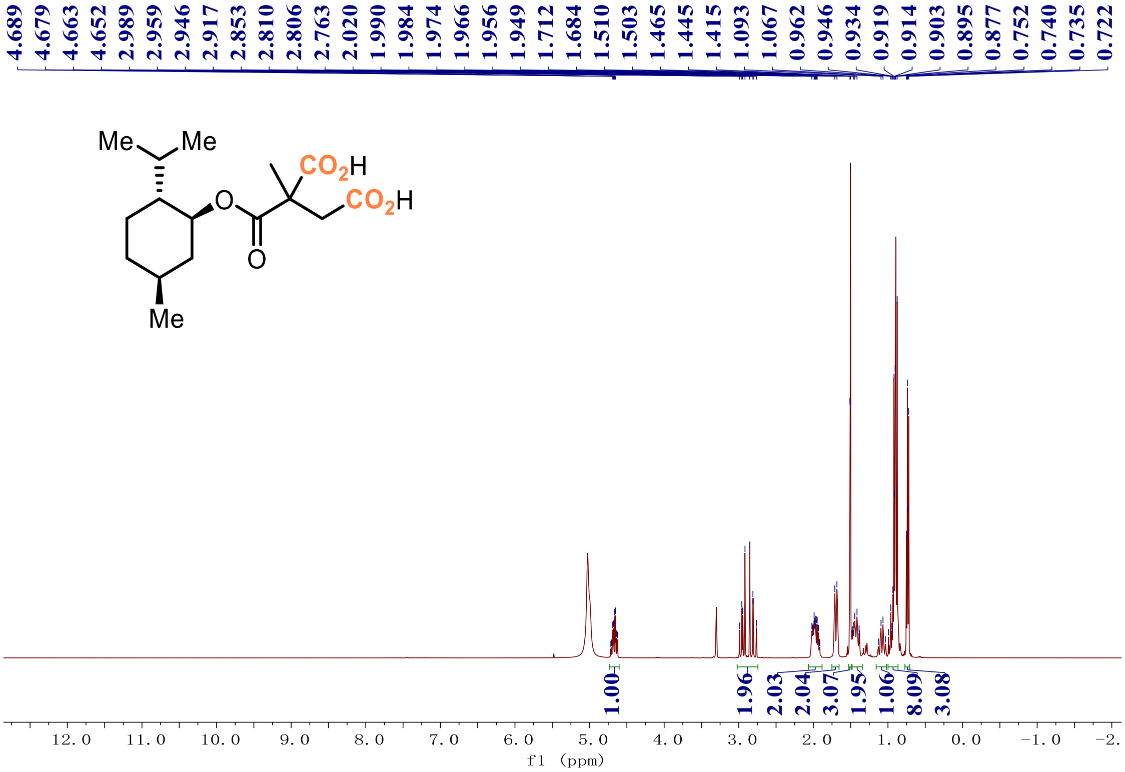

2-((((1*S*,2*R*,5*S*)-2-isopropyl-5-methylcyclohexyl)oxy)carbonyl)-2-methylsuccinic

acid (2cb)

^{13}C NMR (100 MHz, CD_3OD)

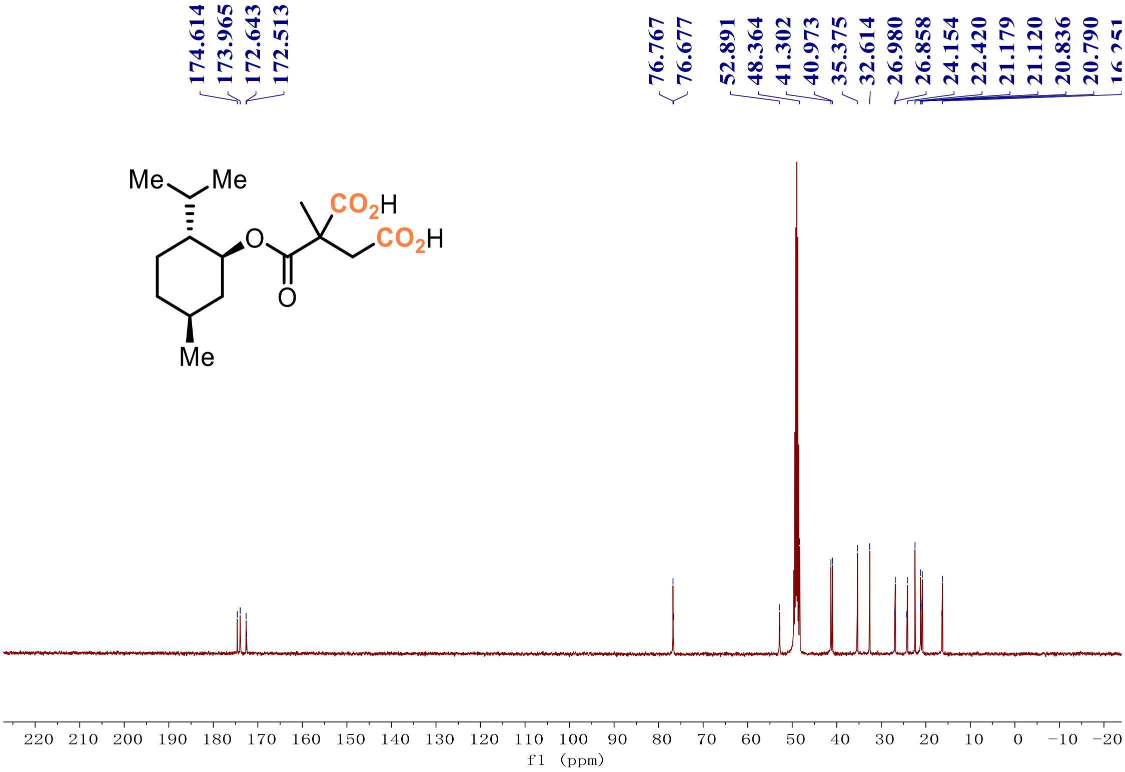

2-(4-((((1S,2R,5S)-2-isopropyl-5-methylcyclohexyl)oxy)carbonyl)phenyl)-2-phenylsuccinic acid (2cc)

^{1}H NMR (400 MHz, CD_3OD)

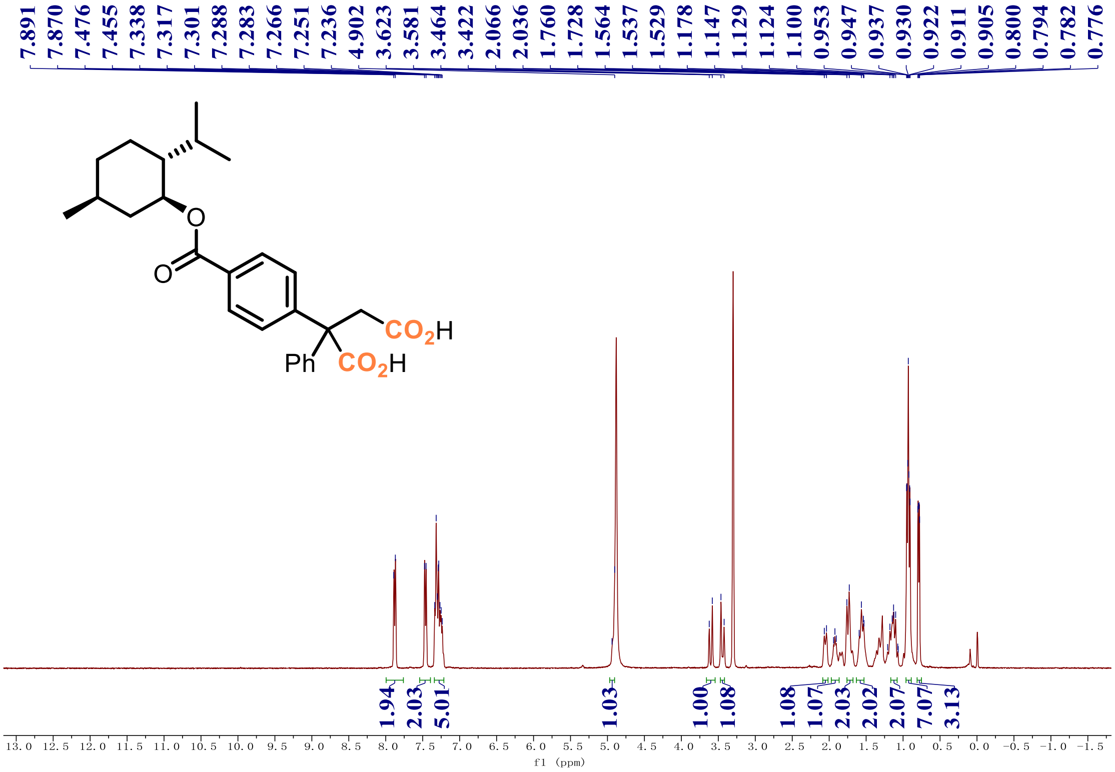

2-(4-((((1S,2R,5S)-2-isopropyl-5-methylcyclohexyl)oxy)carbonyl)phenyl)-2-

phenylsuccinic acid (2cc)

^{13}C NMR (100 MHz, CD_3OD)

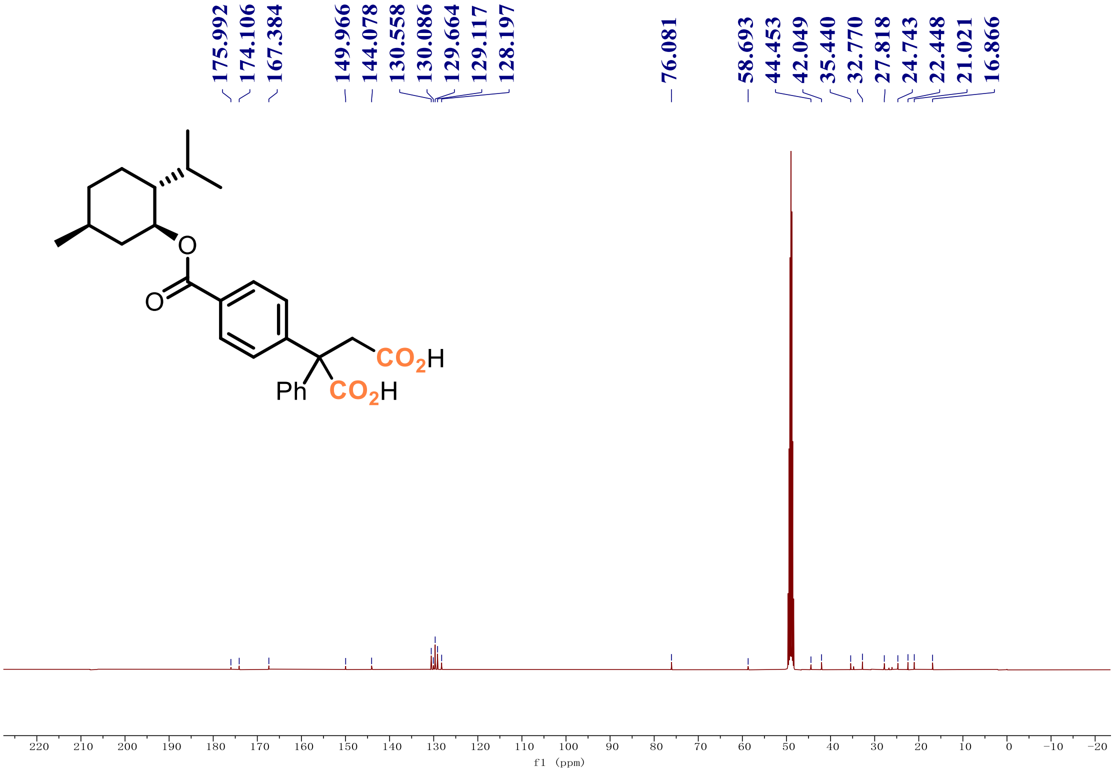

2-(4-(((4-(prop-1-en-2-yl)cyclohex-1-en-1-yl)methoxy)carbonyl)phenyl)succinic acid (2cd)

^{1}H NMR (400 MHz, CD_3OD)

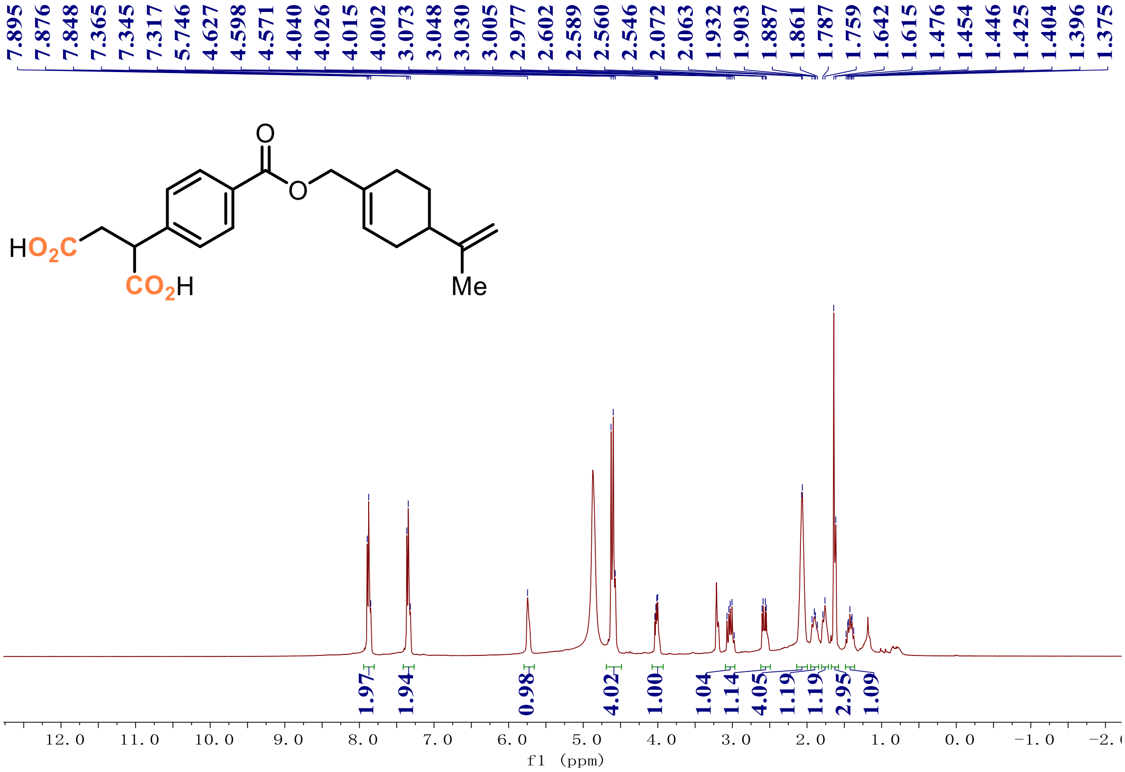

2-(4-(((4-(prop-1-en-2-yl)cyclohex-1-en-1-yl)methoxy)carbonyl)phenyl)succinic

acid (2cd)

^{13}C NMR (100 MHz, CD_3OD)

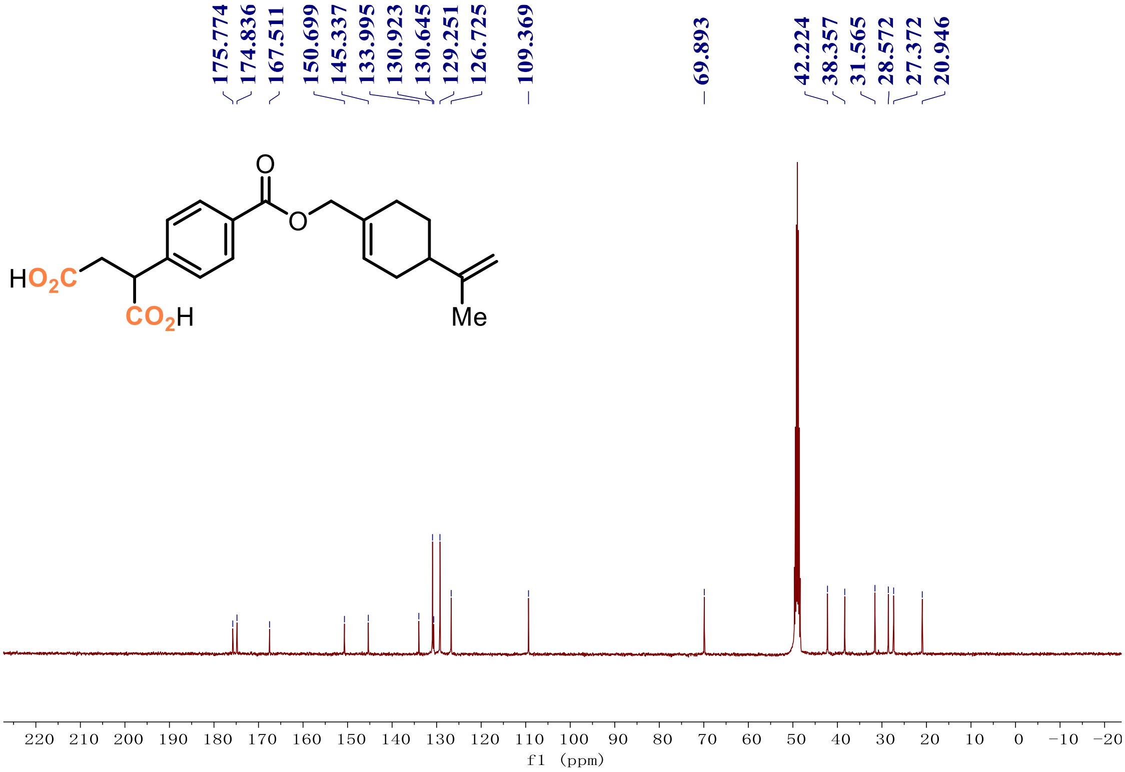

2-(2,2-diphenylvinyl)succinic acid (4a)

^{1}H NMR (400 MHz, CD_3OD)

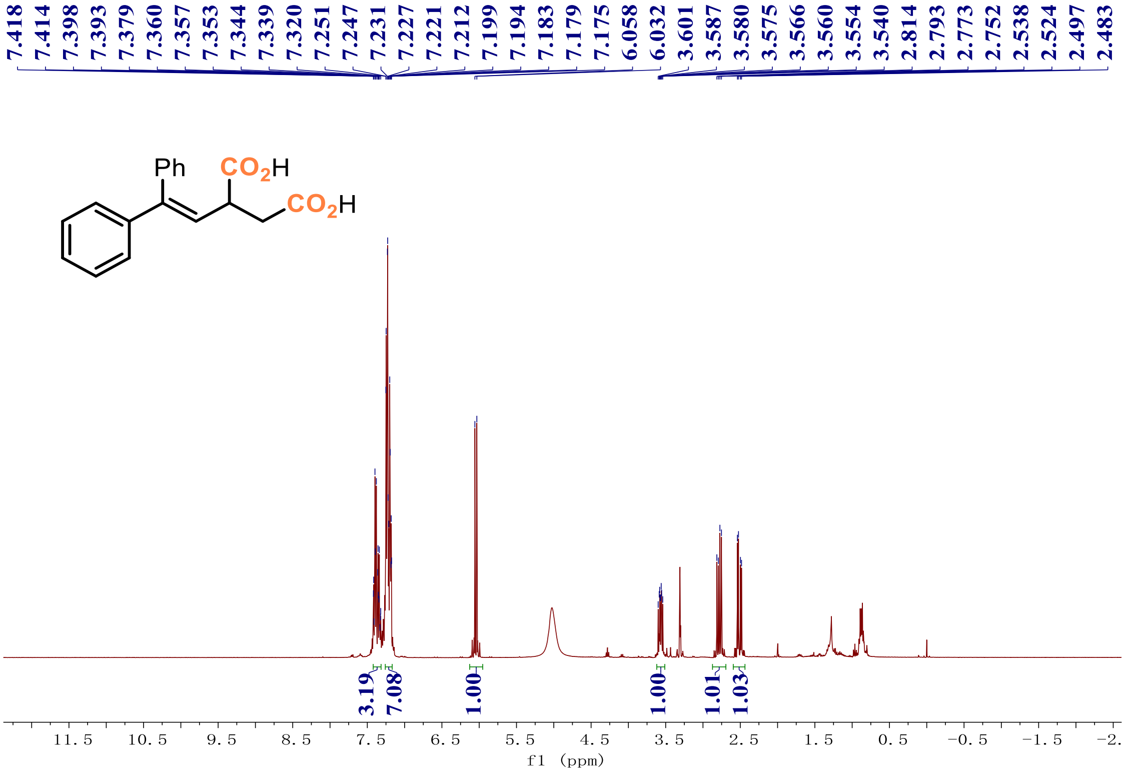

2-(2,2-diphenylvinyl)succinic acid (4a)

^{13}C NMR (100 MHz, CD$_3$OD)

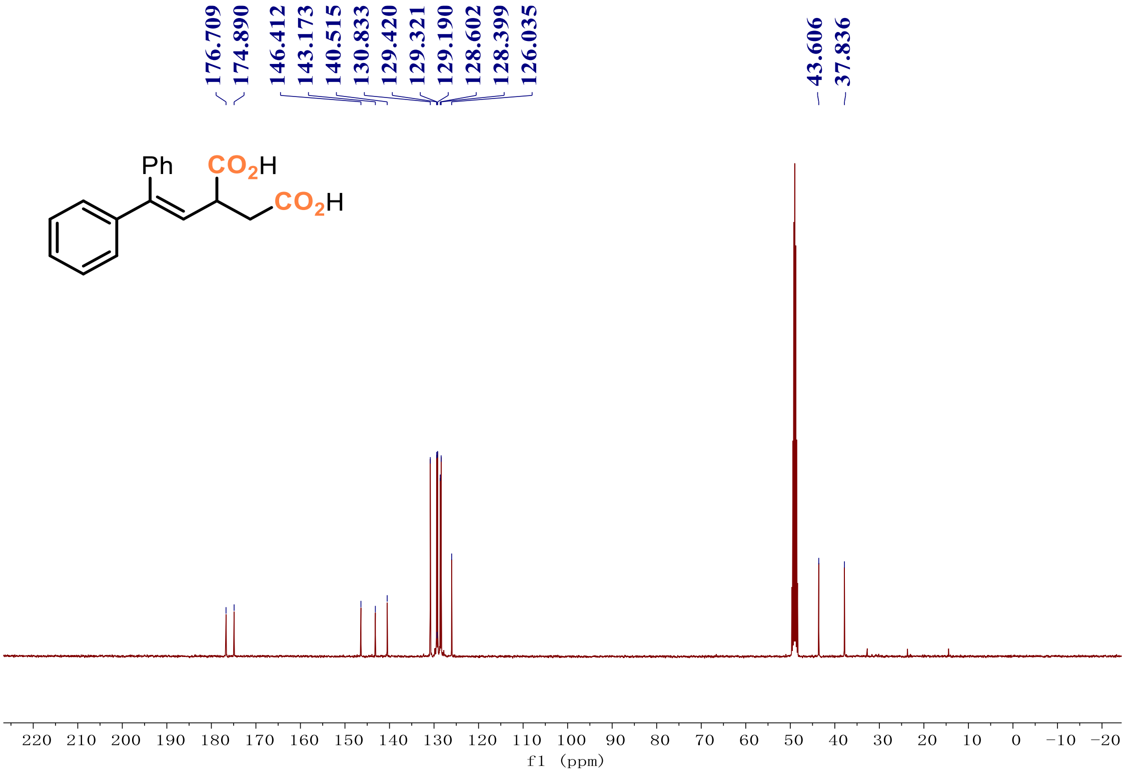

2-(2-phenyl-2-(*m*-tolyl)vinyl)succinic acid (4b)

^{1}H NMR (400 MHz, CD$_3$OD)

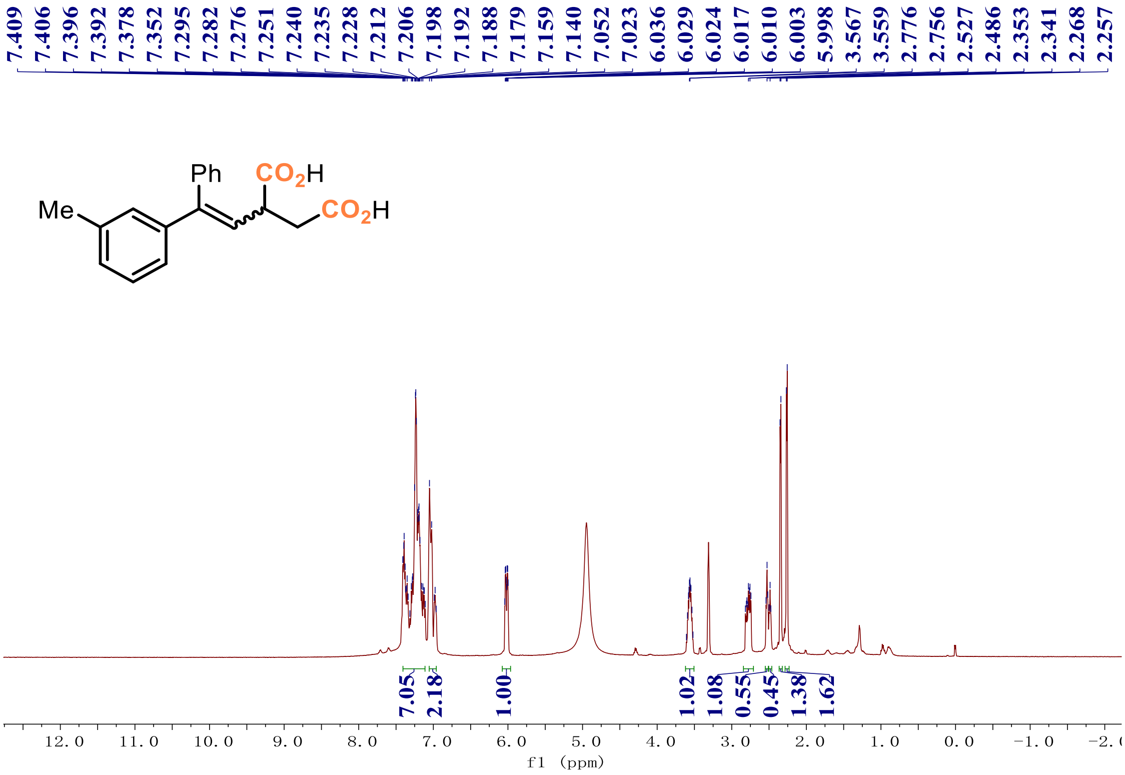

2-(2-phenyl-2-(*m*-tolyl)vinyl)succinic acid (4b)

^{13}C NMR (100 MHz, CD_3OD)

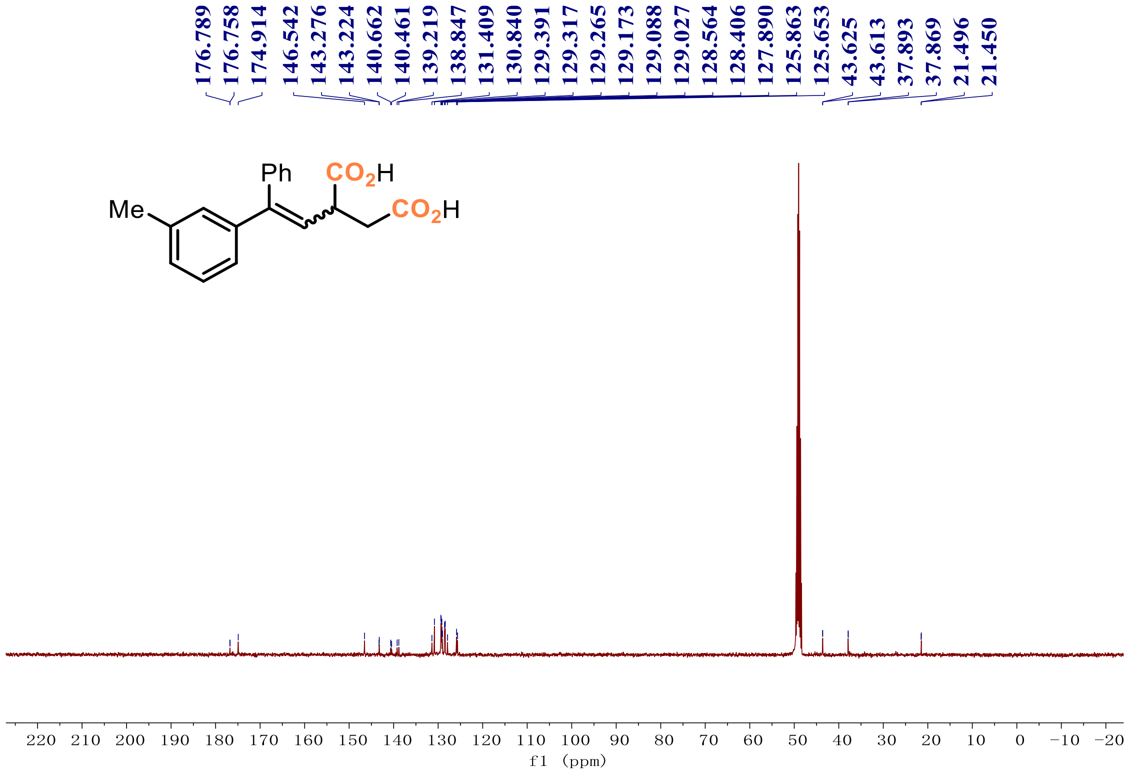

2-(2-phenyl-2-(*p*-tolyl)vinyl)succinic acid (4c)

^{1}H NMR (400 MHz, CD_3OD)

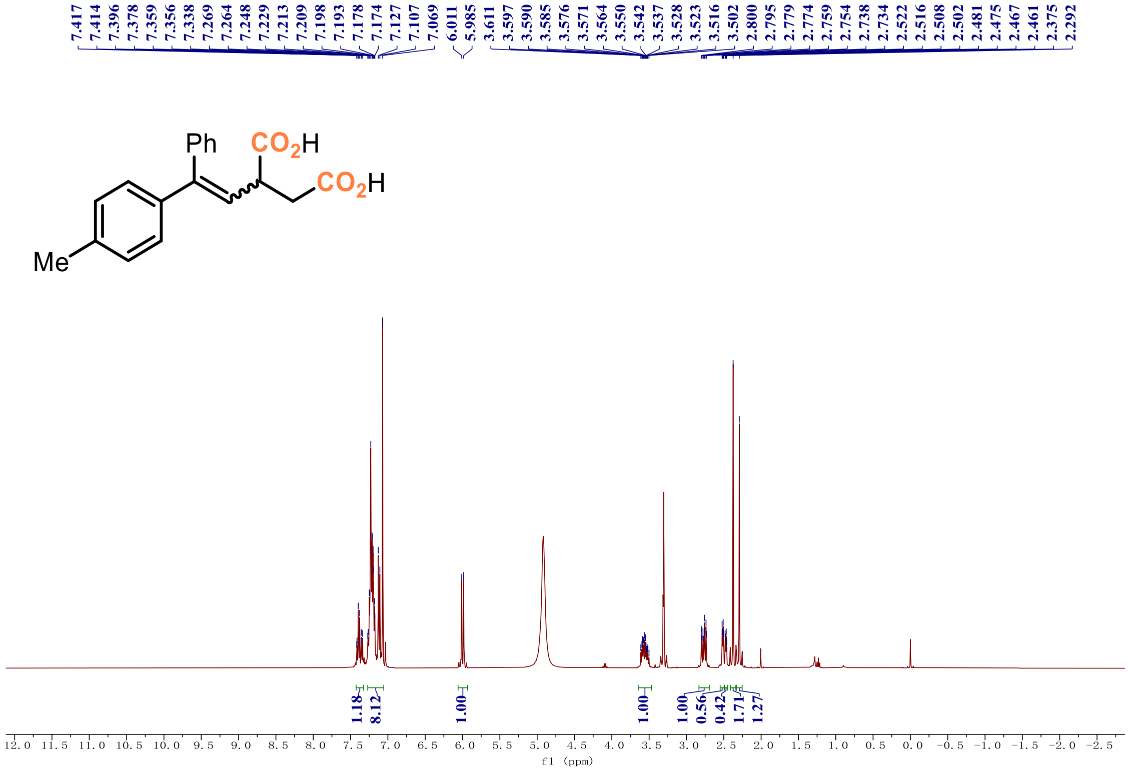

2-(2-phenyl-2-(*p*-tolyl)vinyl)succinic acid (4c)

^{13}C NMR (100 MHz, CD$_3$OD)

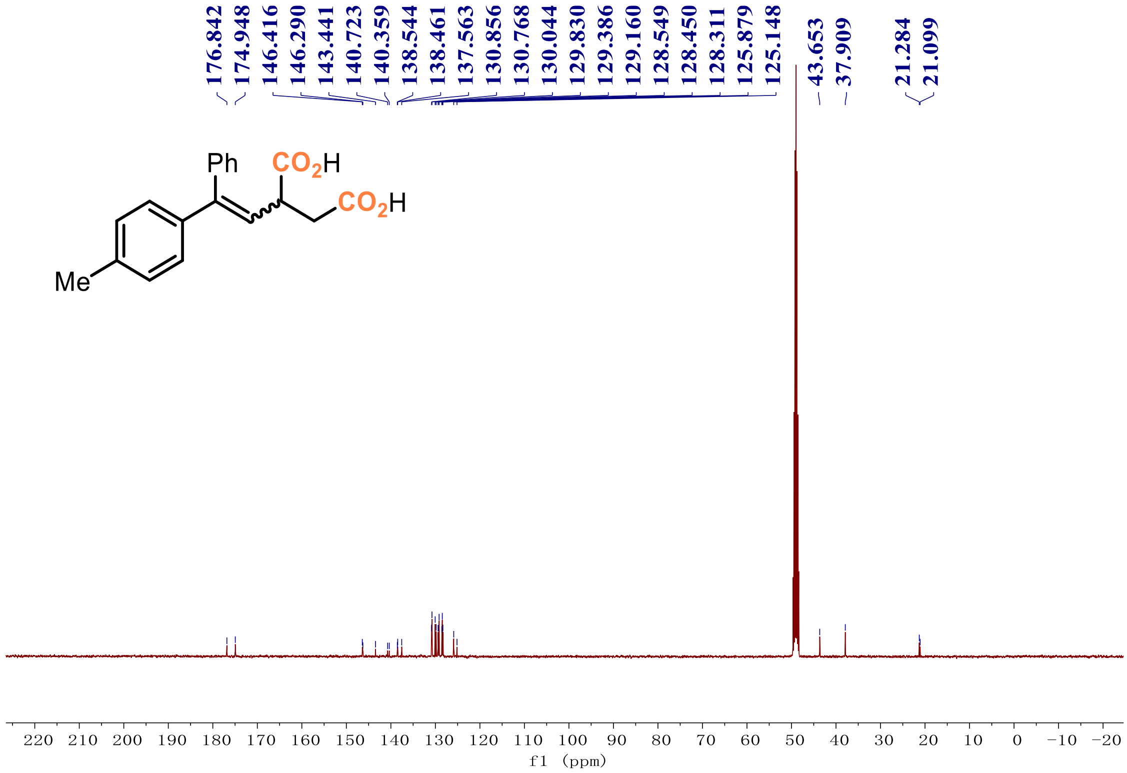

2-(2-([1,1'-biphenyl]-4-yl)-2-phenylvinyl)succinic acid (4d)

^{1}H NMR (400 MHz, CD$_3$OD)

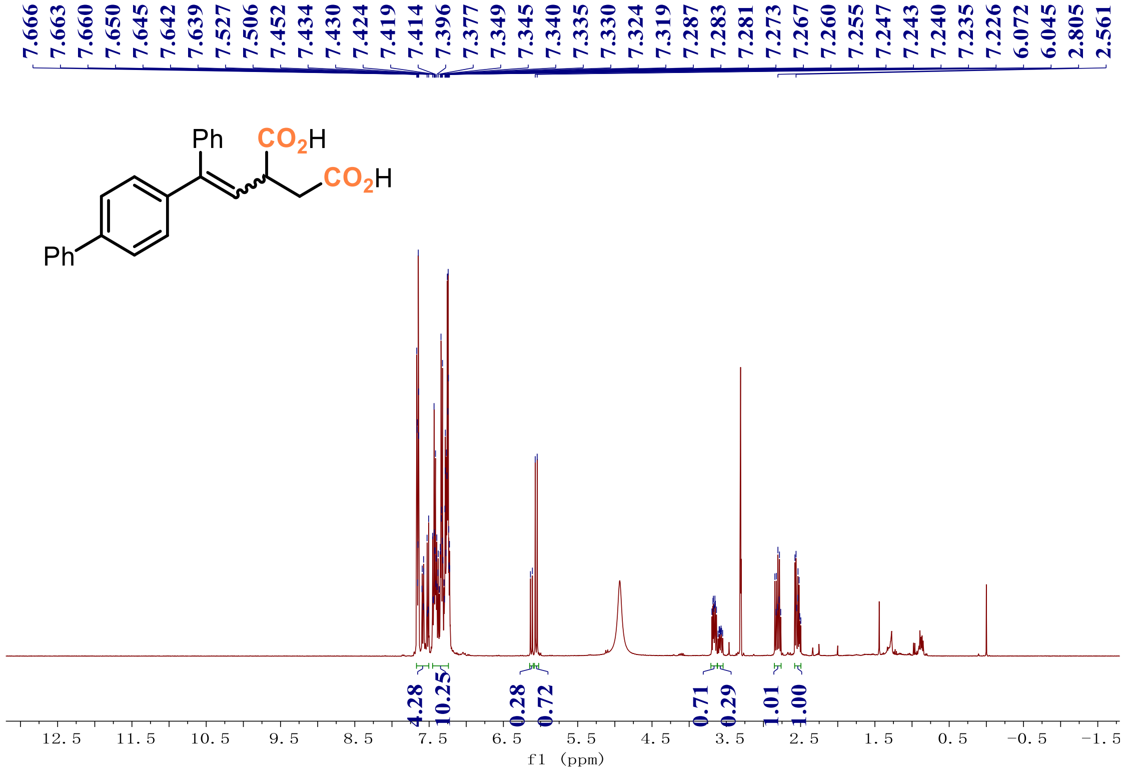

2-(2-([1,1'-biphenyl]-4-yl)-2-phenylvinyl)succinic acid (4d)

^{1}H NMR (400 MHz, CD_3OD)

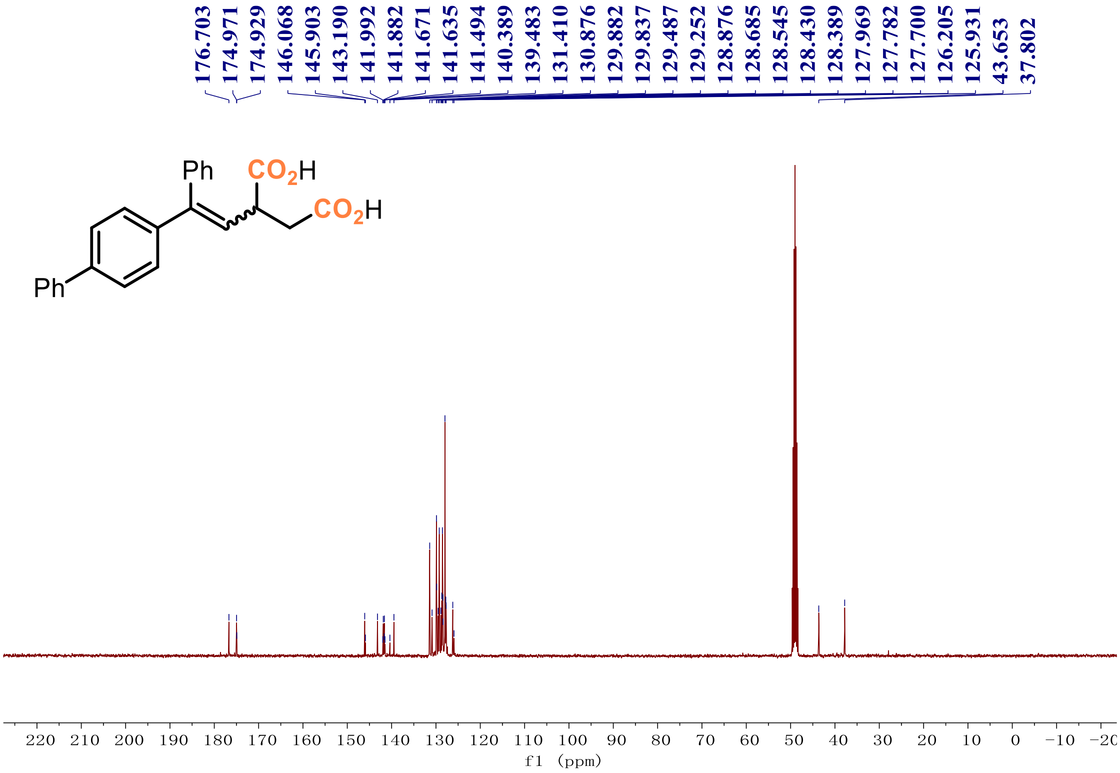

2-(2-(4-fluorophenyl)-2-phenylvinyl)succinic acid (4e)

^{1}H NMR (400 MHz, CD_3OD)

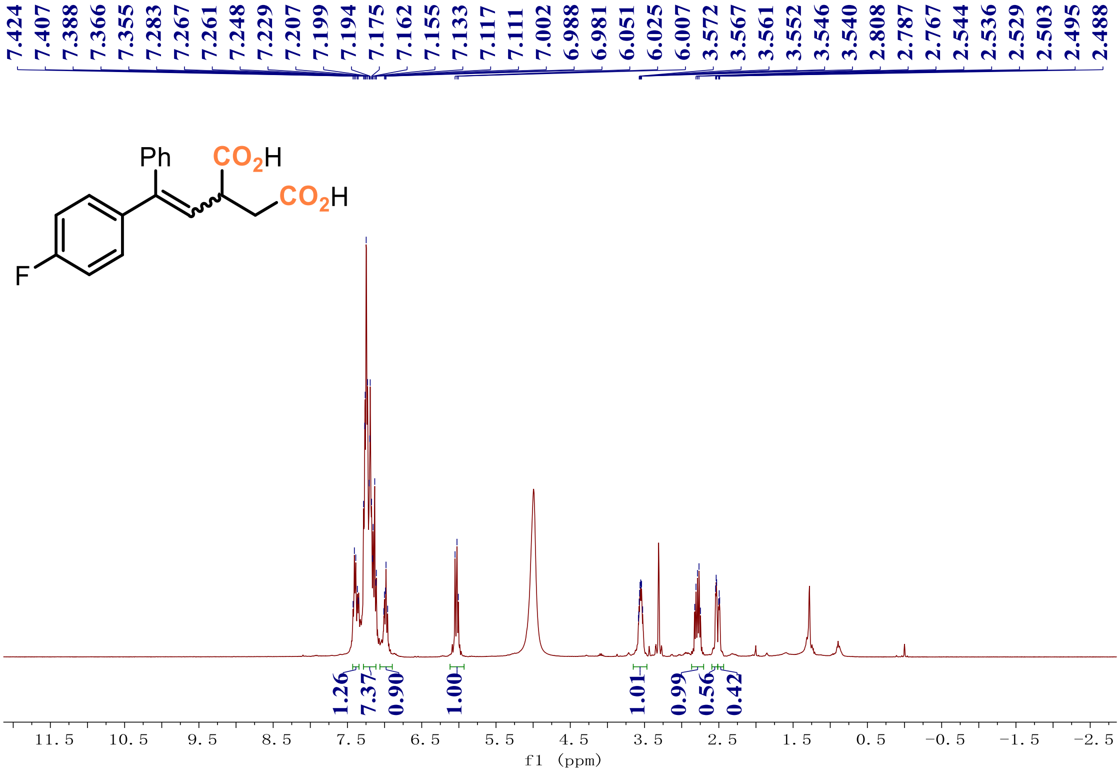

2-(2-(4-fluorophenyl)-2-phenylvinyl)succinic acid (4e)

^{13}C NMR (100 MHz, CD$_3$OD)

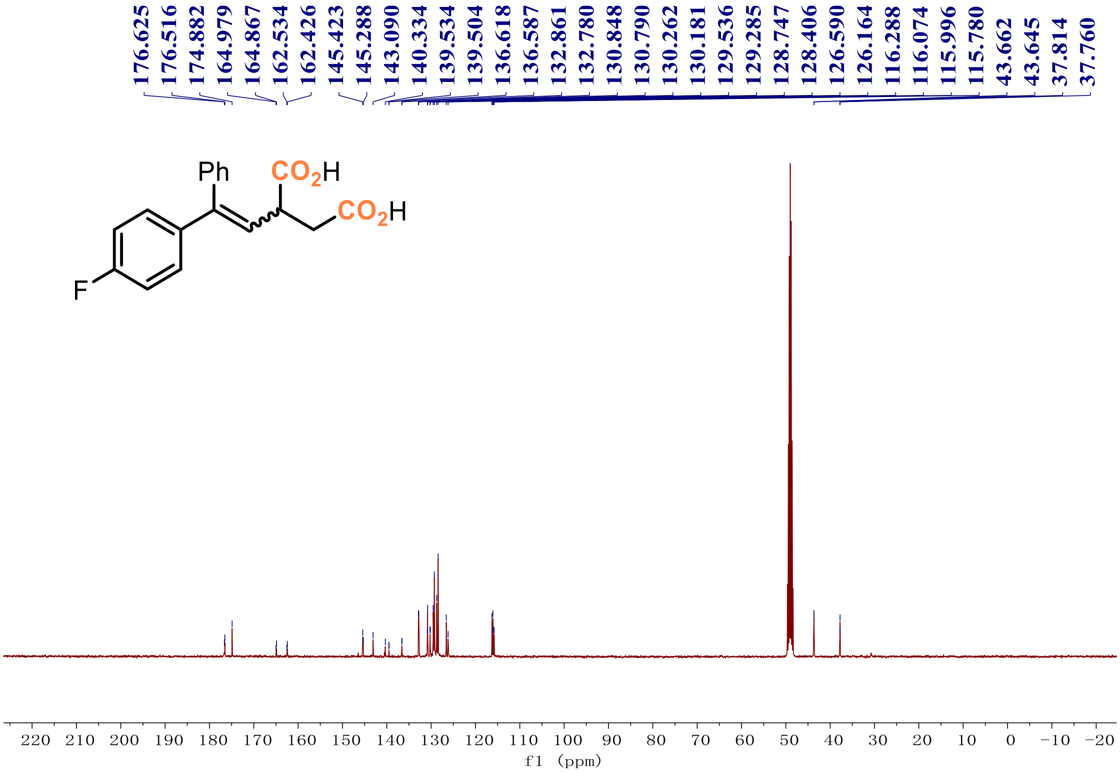

2-(2-(4-fluorophenyl)-2-phenylvinyl)succinic acid (4e)

^{19}F NMR (376 MHz, CD$_3$OD)

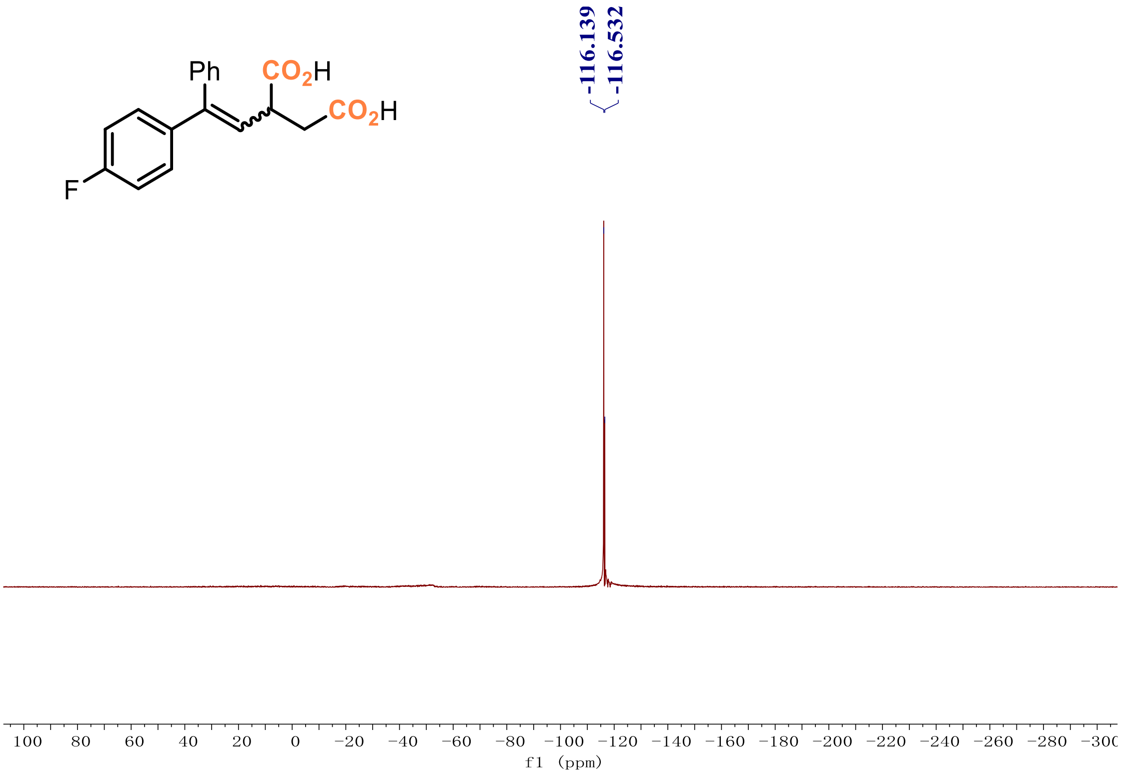

2-(2,2-bis(4-fluorophenyl)vinyl)succinic acid (4f)

^{1}H NMR (400 MHz, CD_3OD)

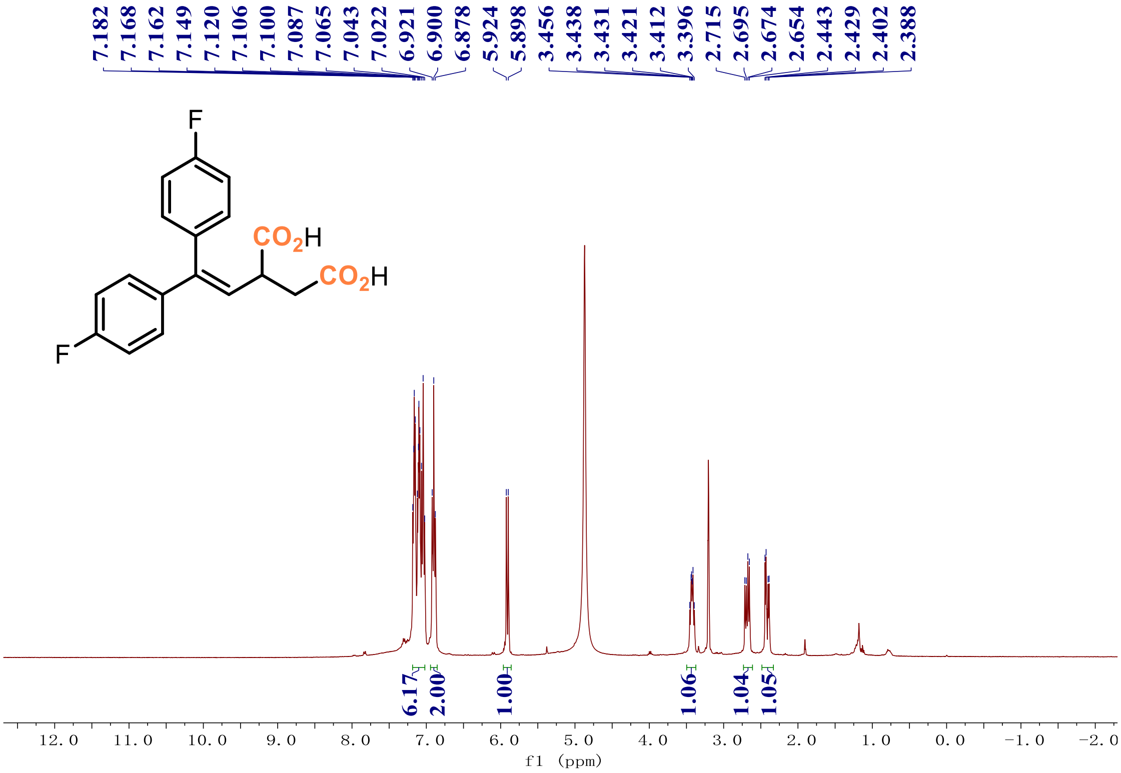

2-(2,2-bis(4-fluorophenyl)vinyl)succinic acid (4f)

^{13}C NMR (100 MHz, CD_3OD)

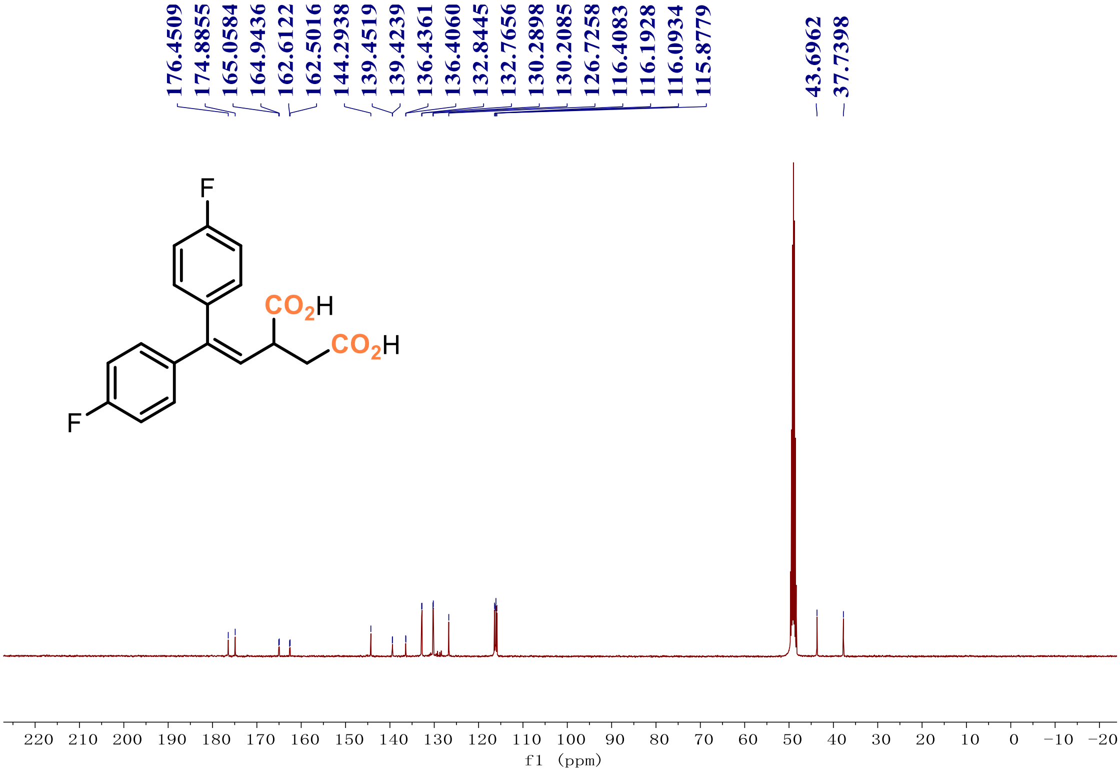

2-(2,2-bis(4-fluorophenyl)vinyl)succinic acid (4f)

^{19}F NMR (376 MHz, CD_3OD)

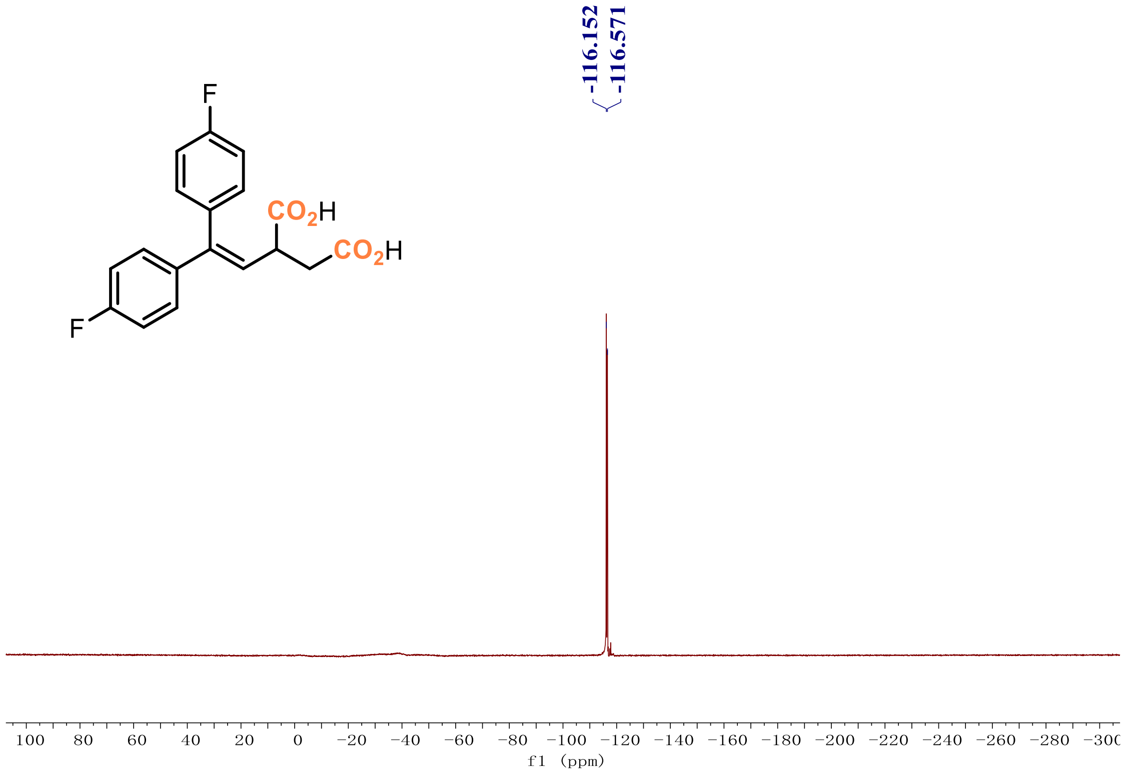

2-phenylhex-3-enedioic acid (5g)

^{1}H NMR (400 MHz, CD_3OD)

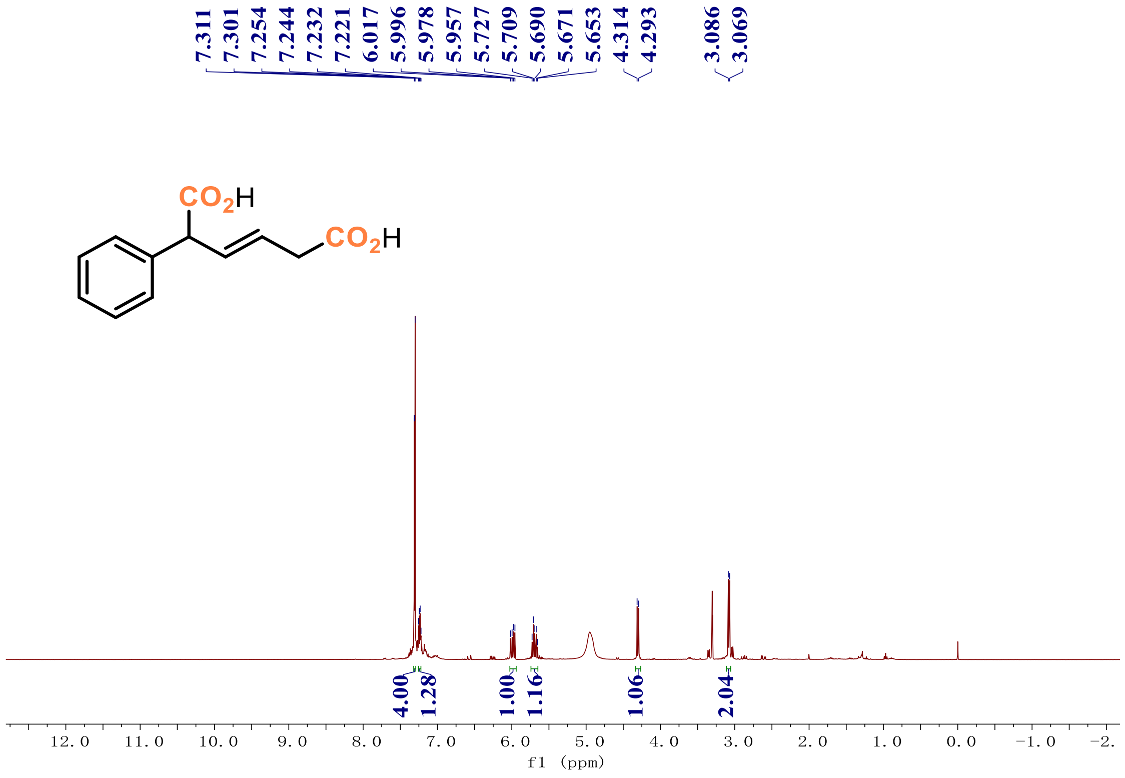

2-phenylhex-3-enedioic acid (5g)

^{13}C NMR (100 MHz, CD_3OD)

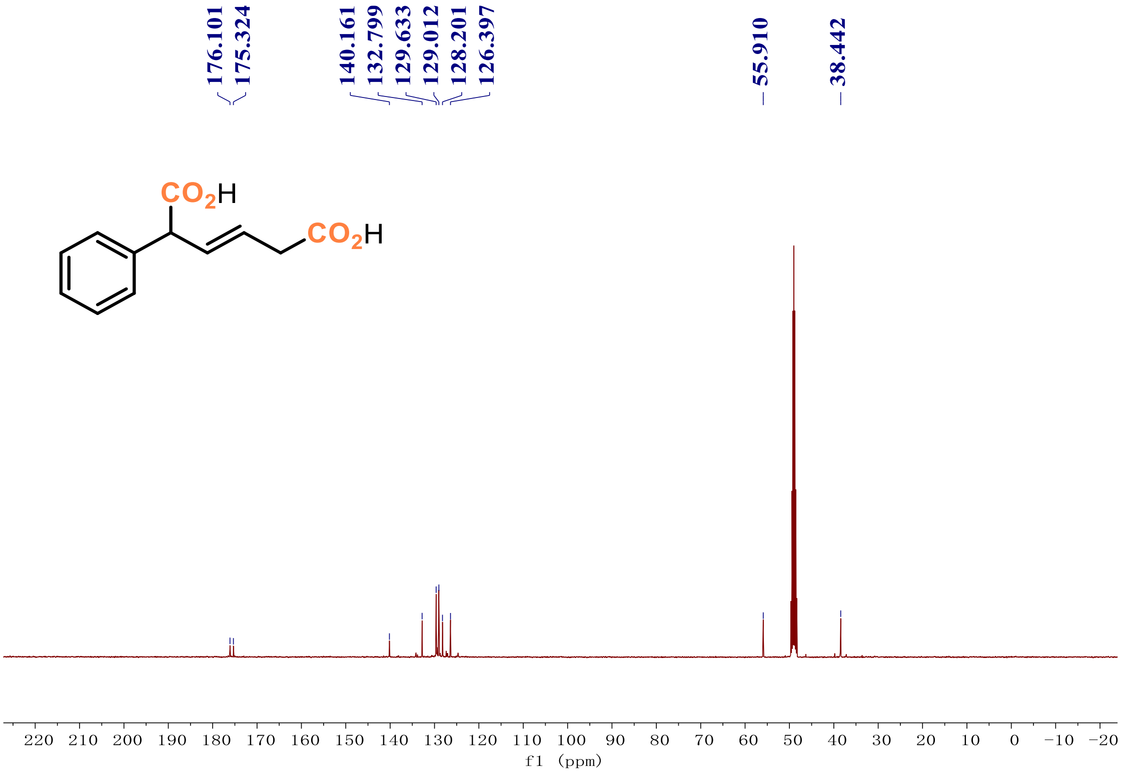

2-([1,1'-biphenyl]-4-yl)hex-3-enedioic acid (5h)

^{1}H NMR (400 MHz, CD_3OD)

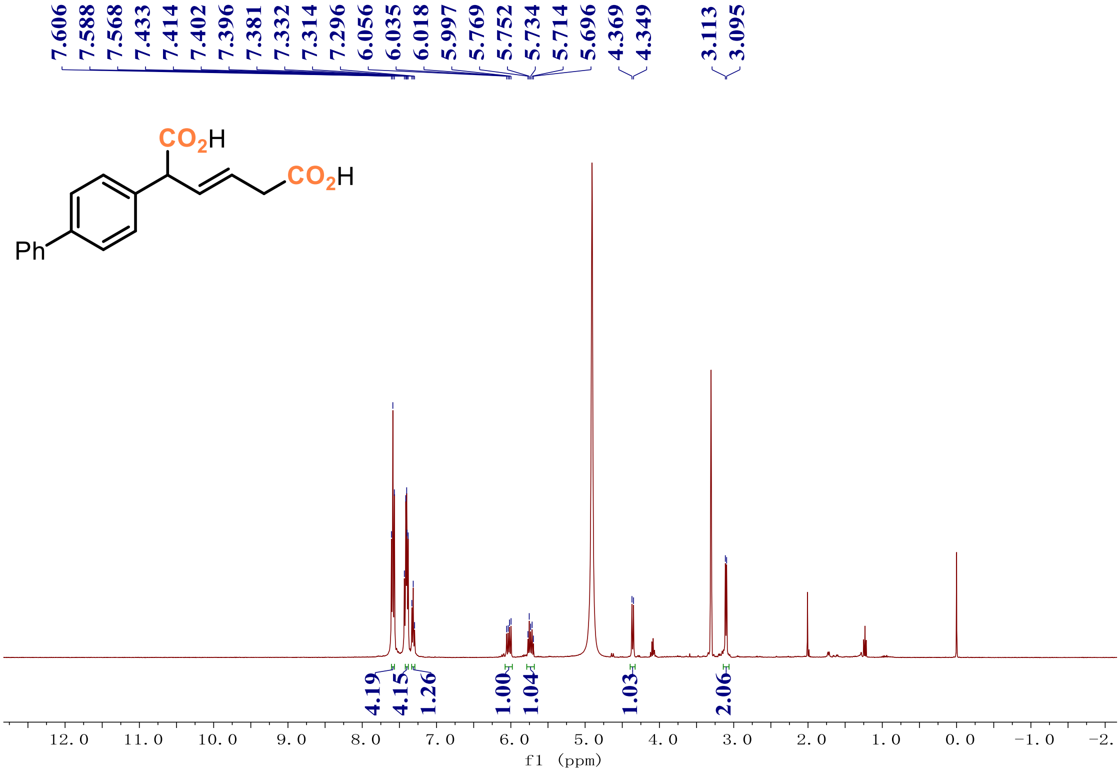

2-([1,1'-biphenyl]-4-yl)hex-3-enedioic acid (5h)

^{13}C NMR (100 MHz, CD_3OD)

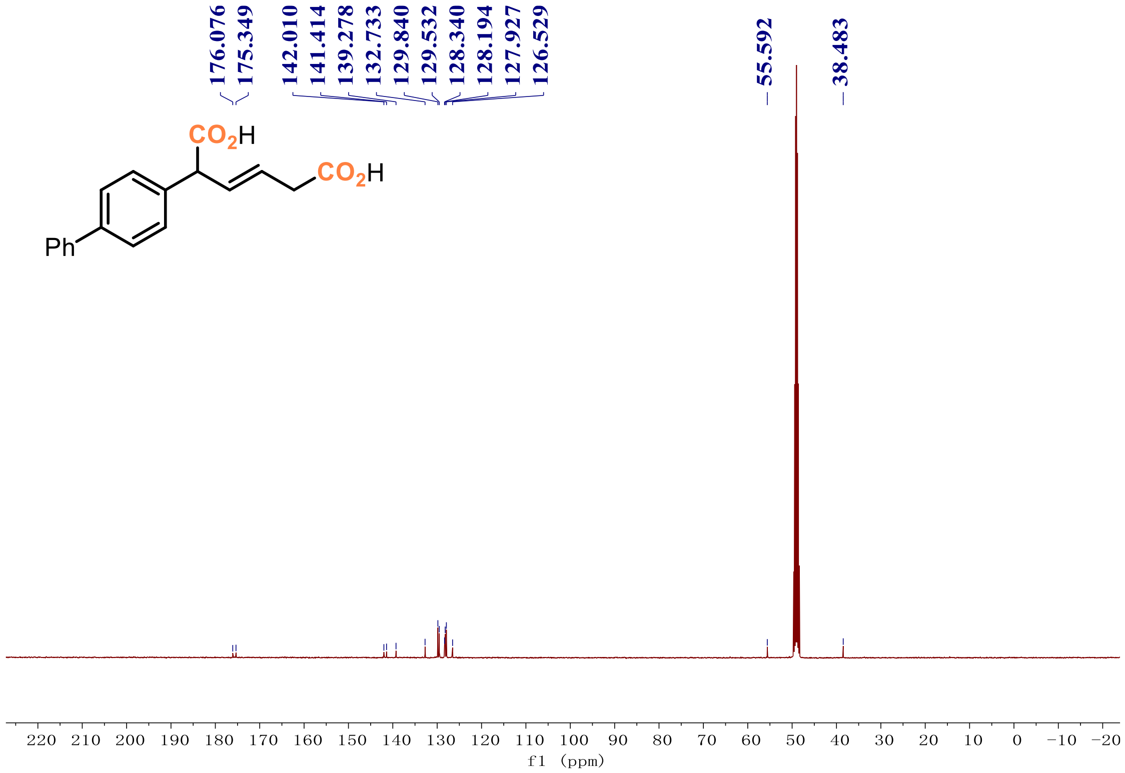

2-(naphthalen-2-yl)hex-3-enedioic acid (5i)

^{1}H NMR (400 MHz, CD_3OD)

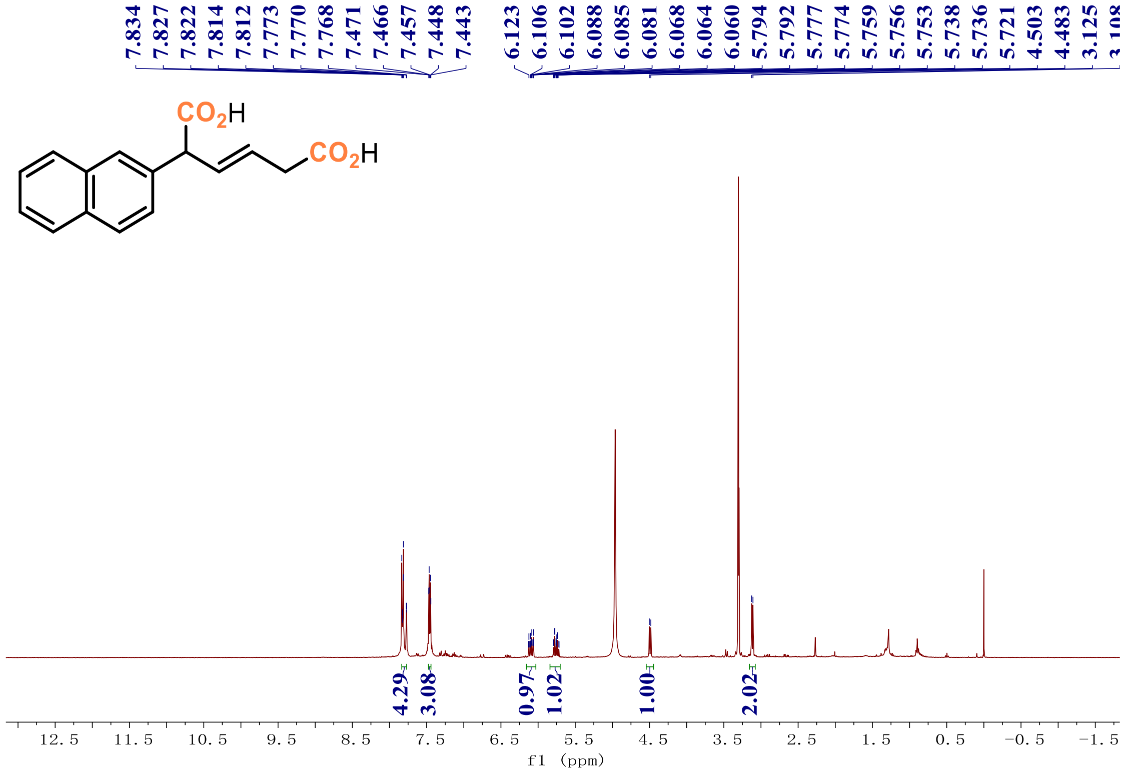

2-(naphthalen-2-yl)hex-3-enedioic acid (5i)

^{13}C NMR (100 MHz, CD_3OD)

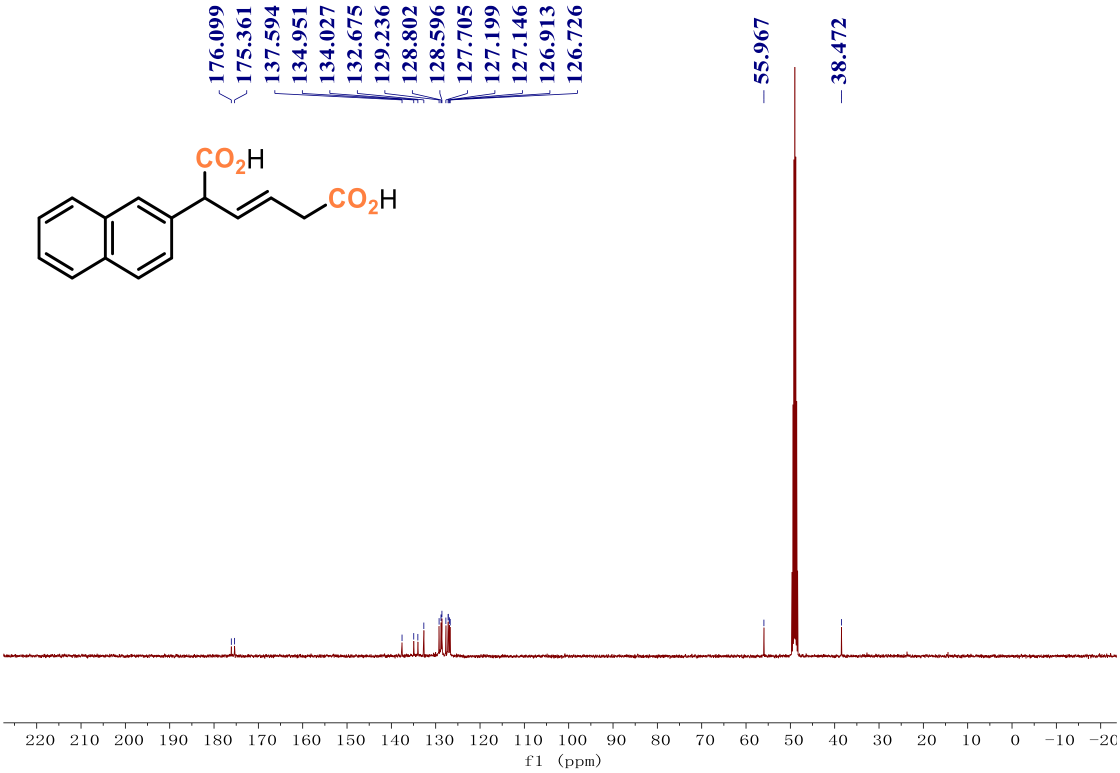

2-phenyl-2-propylhex-3-enedioic acid (5j)

^{1}H NMR (400 MHz, CD_3OD)

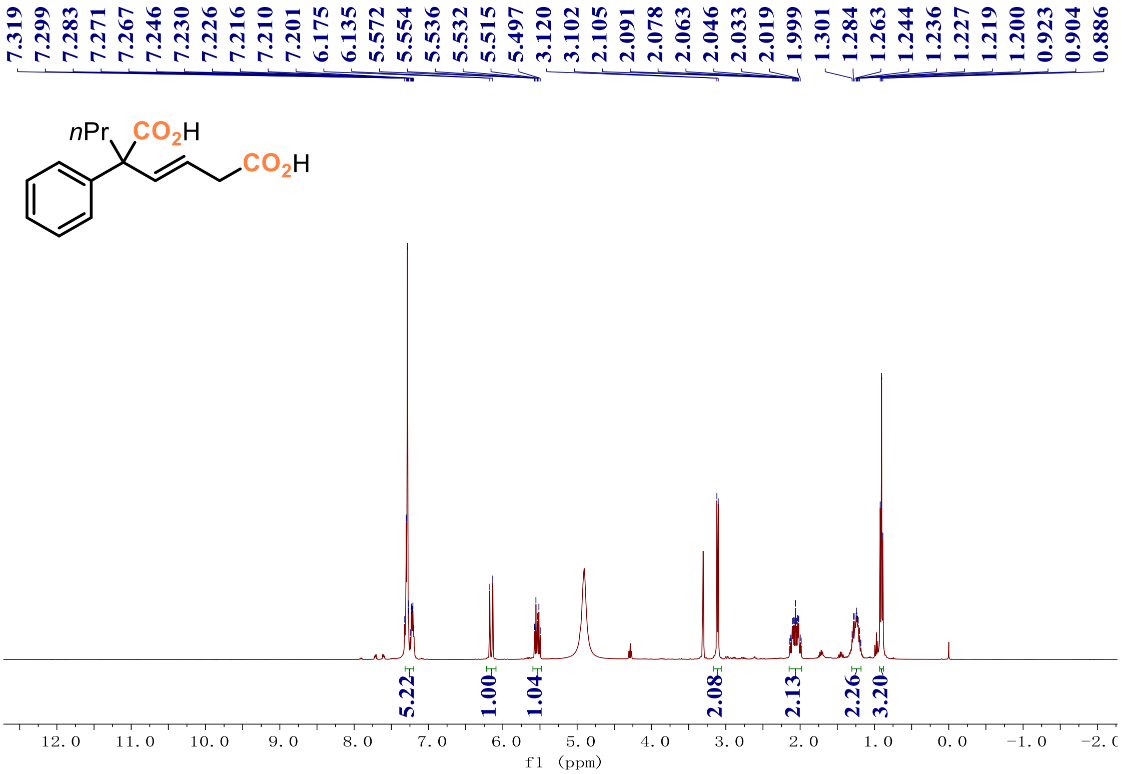

2-phenyl-2-propylhex-3-enedioic acid (5j)

^{13}C NMR (100 MHz, CD3OD)

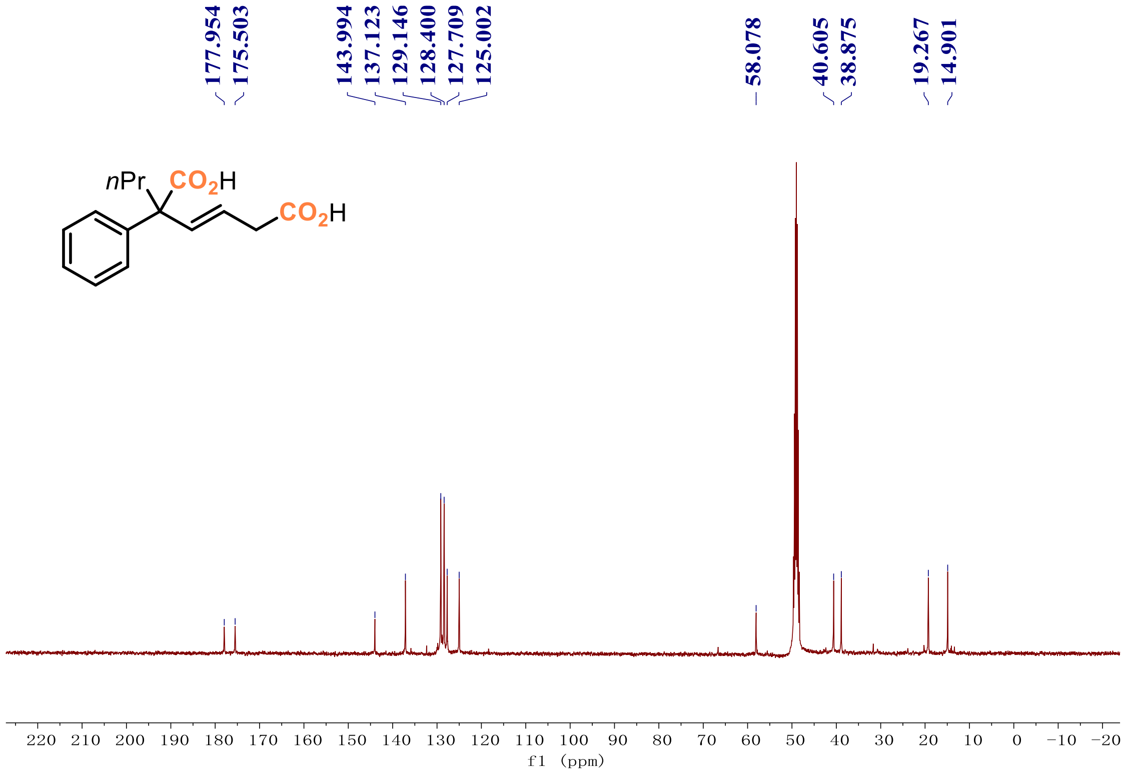

2-methyl-5-phenylhex-3-enedioic acid (5k)

^{1}H NMR (400 MHz, CD3OD)

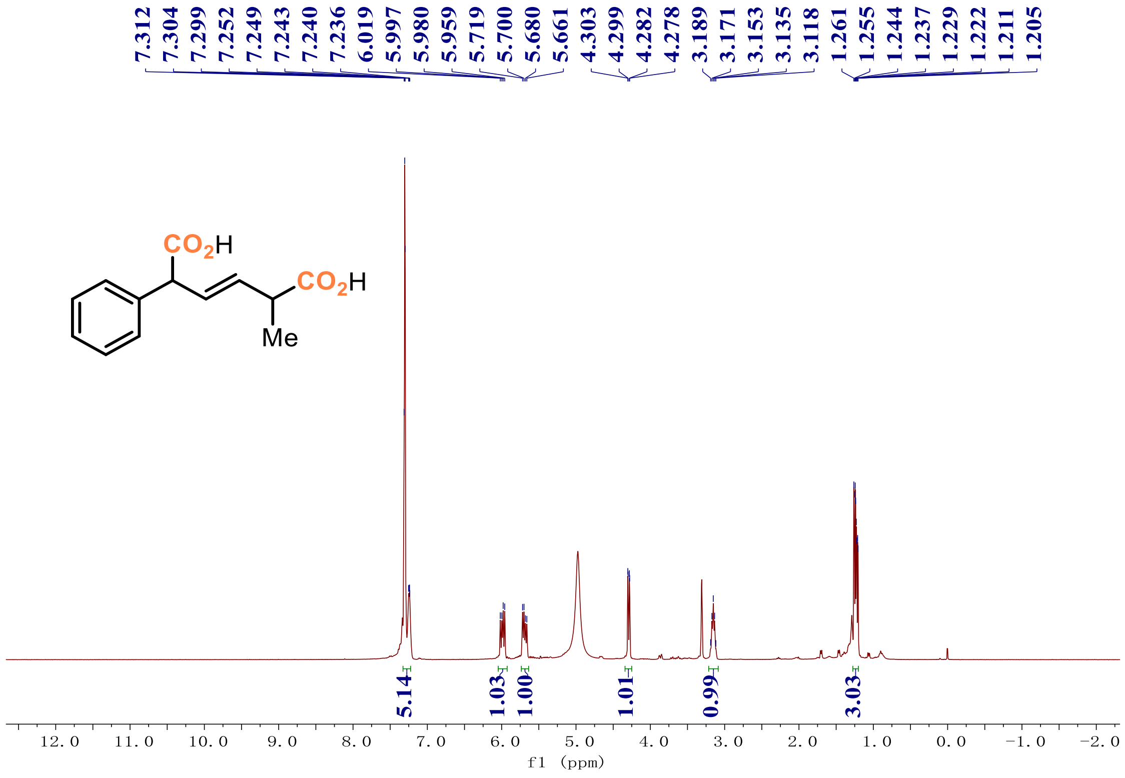

2-methyl-5-phenylhex-3-enedioic acid (5k)

^{13}C NMR (100 MHz, CD_3OD)

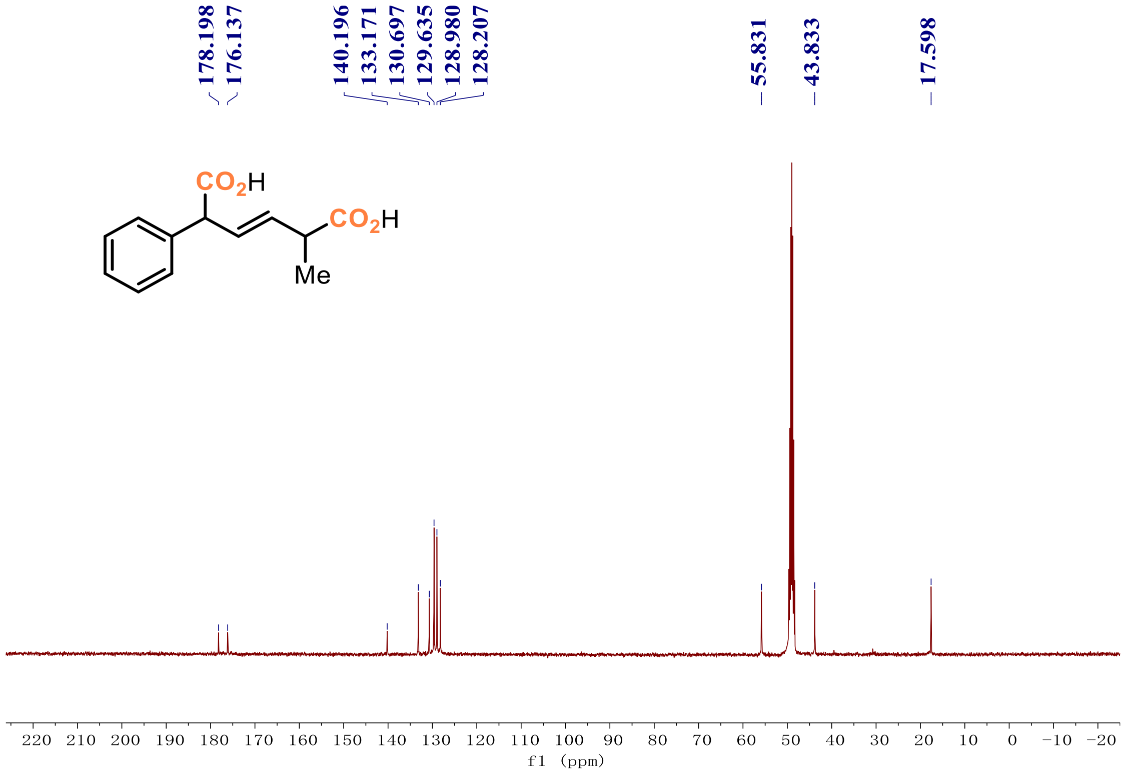

4-methyl-2-phenylhex-3-enedioic acid (5l)

^{1}H NMR (400 MHz, CD_3OD)

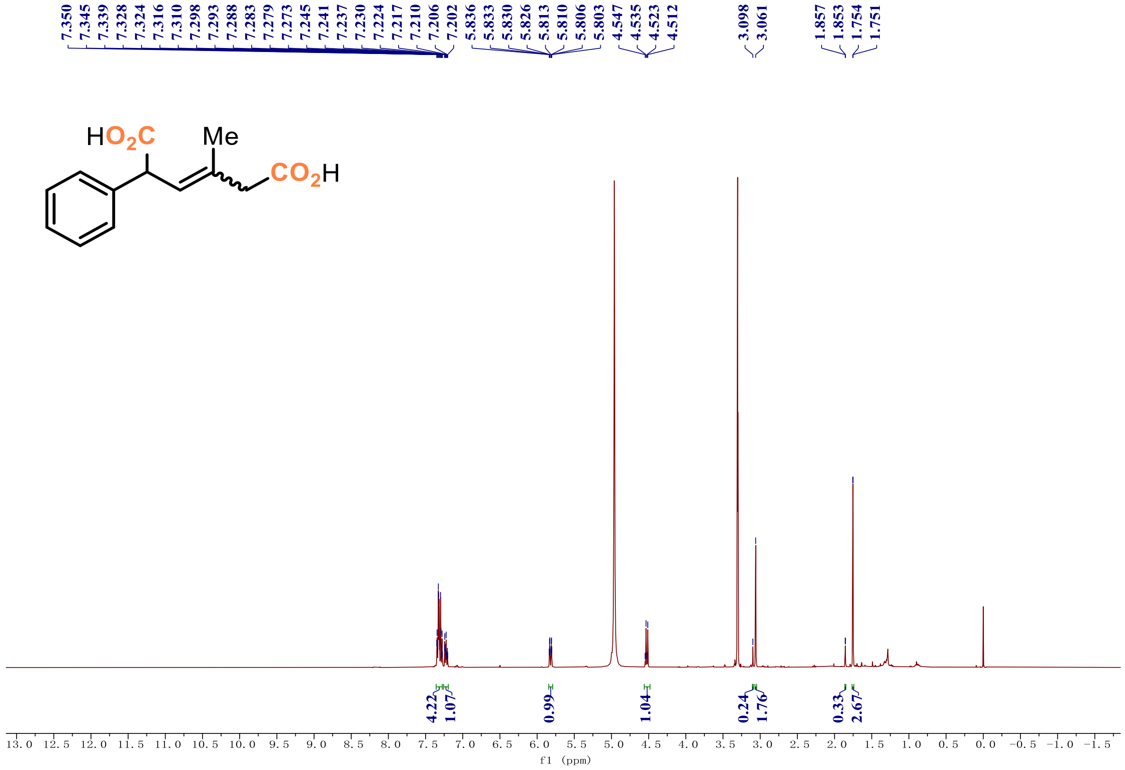

4-methyl-2-phenylhex-3-enedioic acid (5l)

^{13}C NMR (100 MHz, CD_3OD)

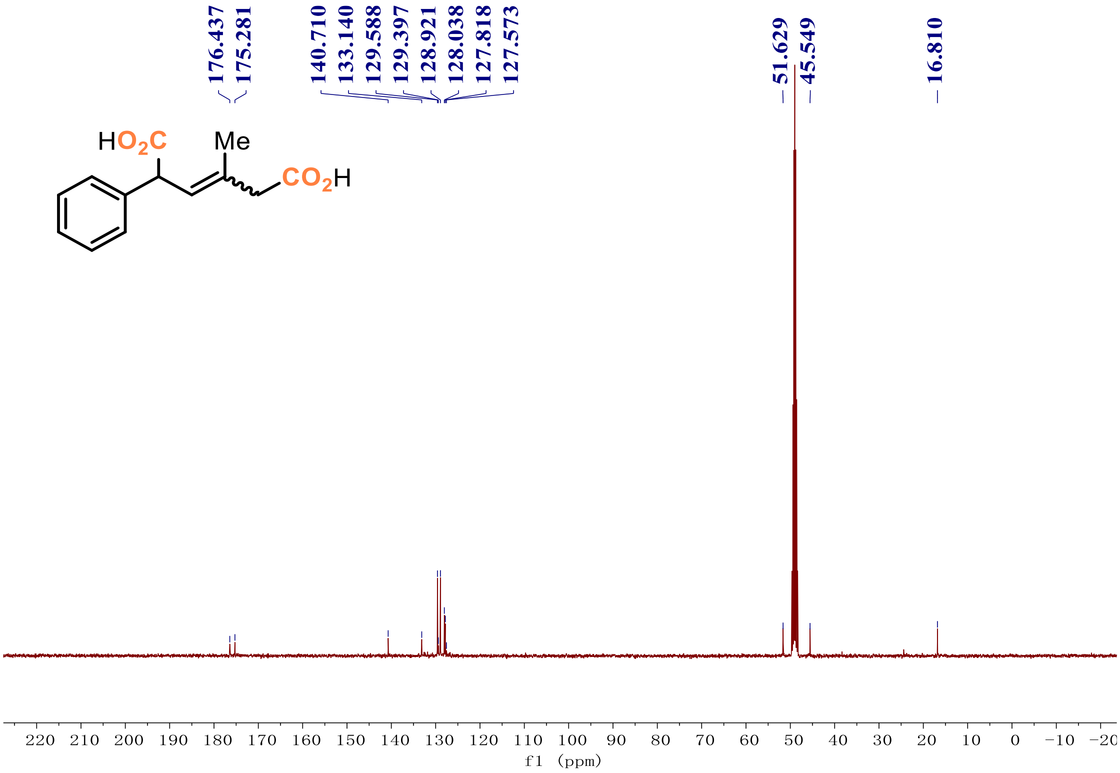

2-phenylocta-3,5-dienedioic acid (5m)

^{1}H NMR (400 MHz, CD_3OD)

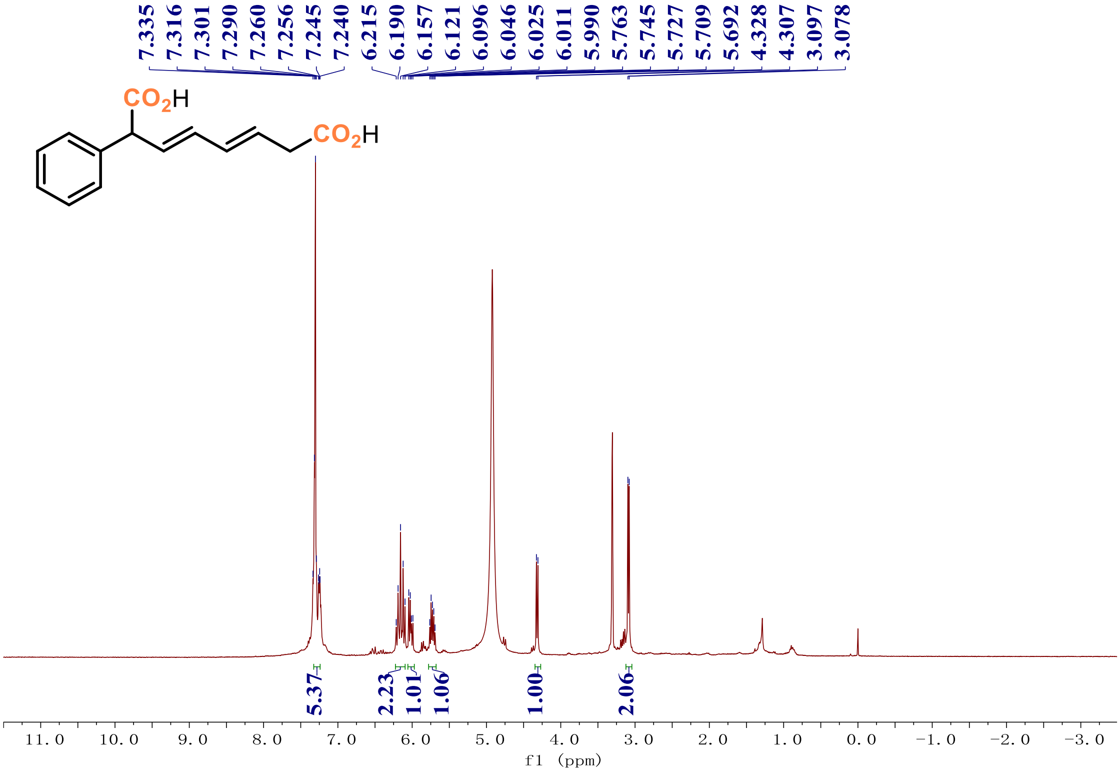

2-phenylocta-3,5-dienedioic acid (5m)

^{13}C NMR (100 MHz, CD_3OD)

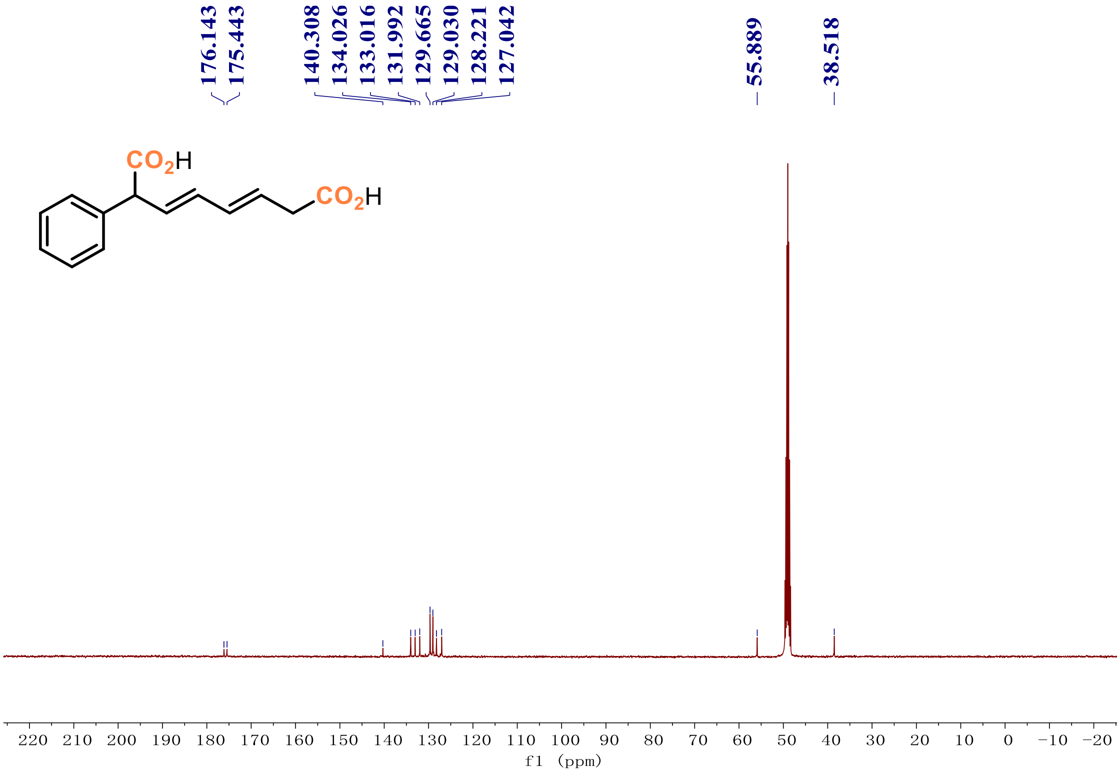

1-(*tert*-butoxycarbonyl)indoline-2,3-dicarboxylic acid (7a)

^{1}H NMR (400 MHz, CD_3OD)

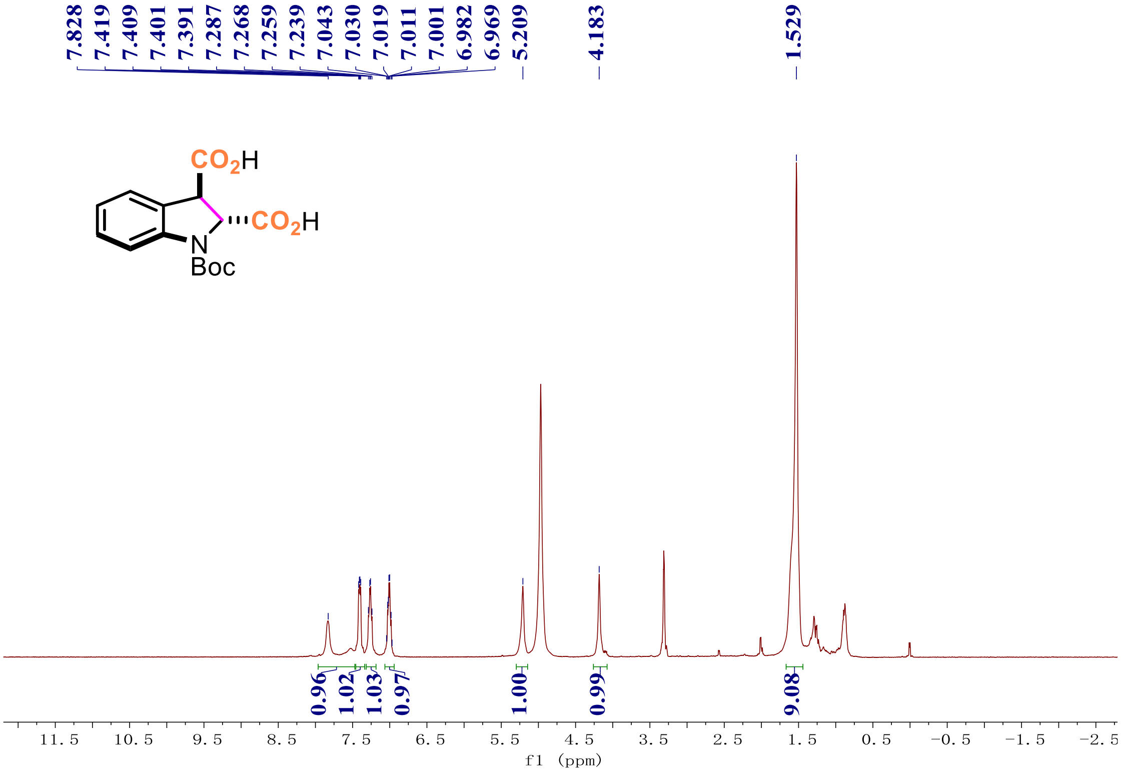

1-(*tert*-butoxycarbonyl)indoline-2,3-dicarboxylic acid (7a)

^{13}C NMR (100 MHz, CD_3OD)

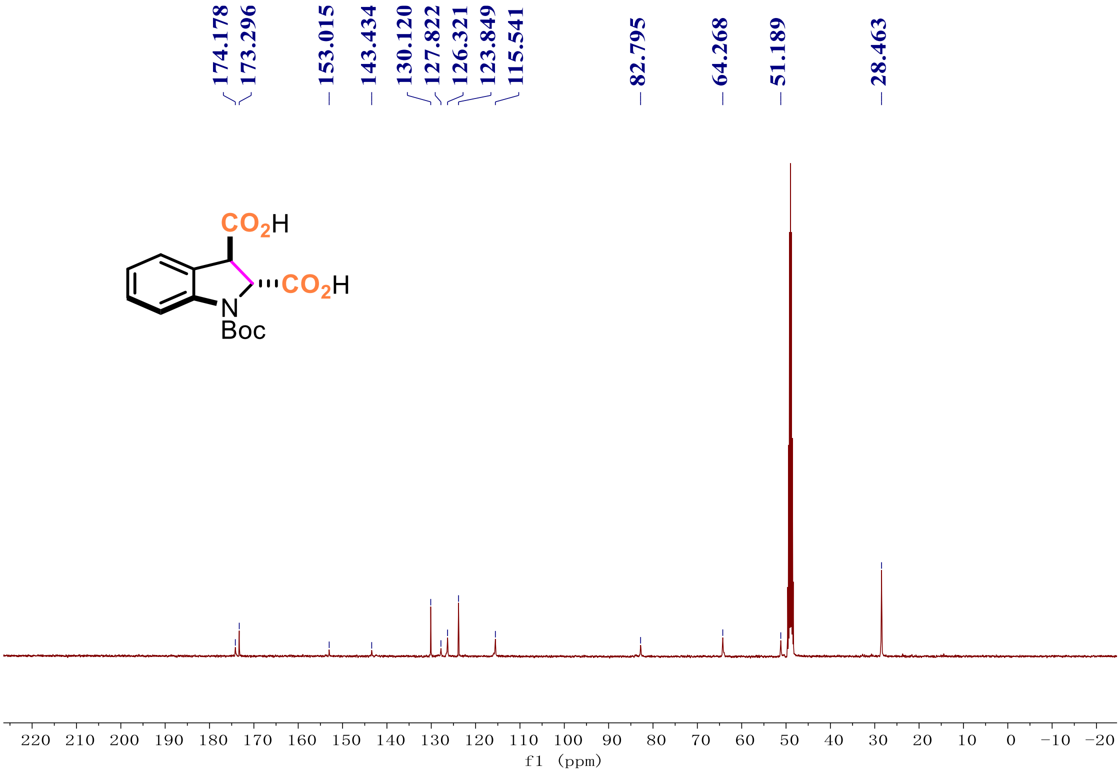

1-(*tert*-butoxycarbonyl)-4-fluoroindoline-2,3-dicarboxylic acid (7b)

^{1}H NMR (400 MHz, DMSO-d_6)

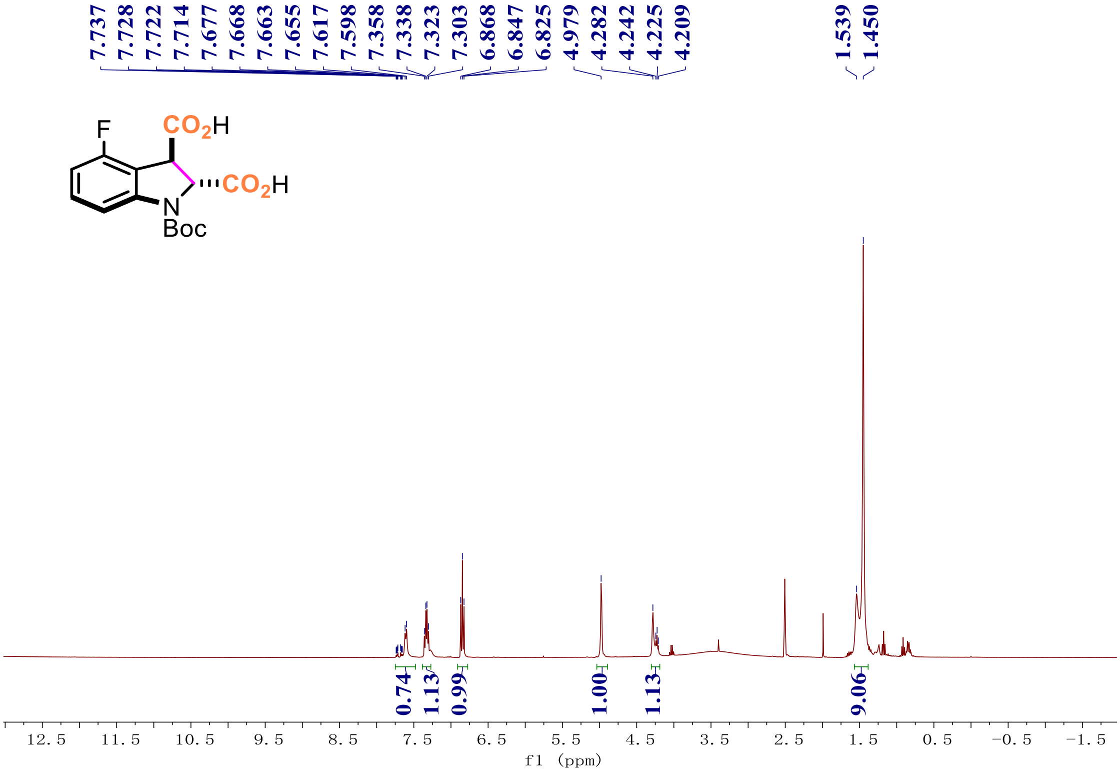

1-(*tert*-butoxycarbonyl)-4-fluoroindoline-2,3-dicarboxylic acid (7b)

^{13}C NMR (100 MHz, DMSO-*d*$_6$)

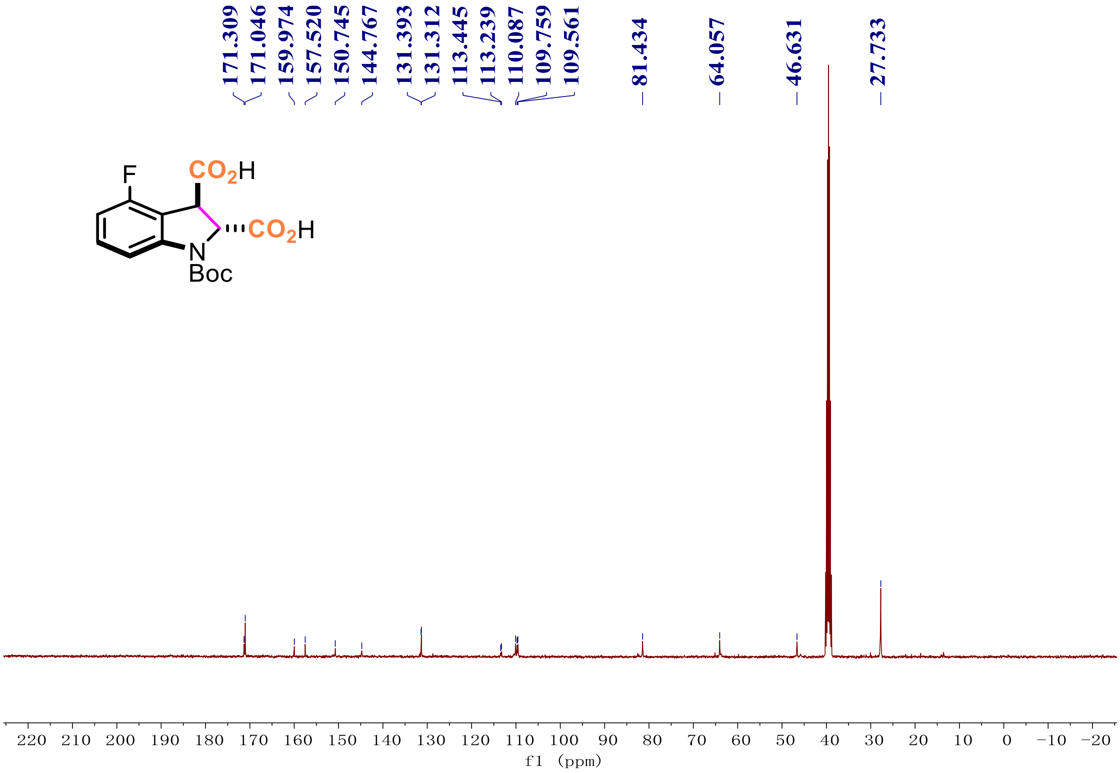

1-(*tert*-butoxycarbonyl)-4-fluoroindoline-2,3-dicarboxylic acid (7b)
^{19}F NMR (376 MHz, DMSO-*d*$_6$)

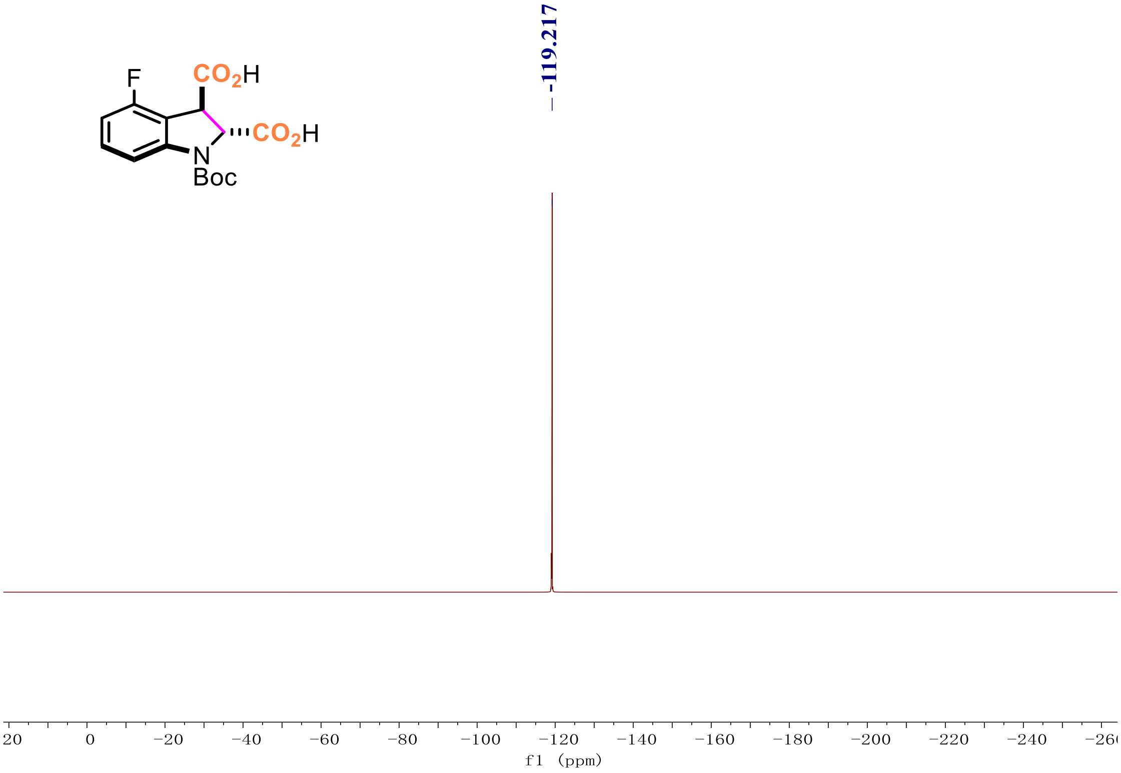

1-(*tert*-butoxycarbonyl)-5-fluoroindoline-2,3-dicarboxylic acid (7c)

^{1}H NMR (400 MHz, CD_3OD)

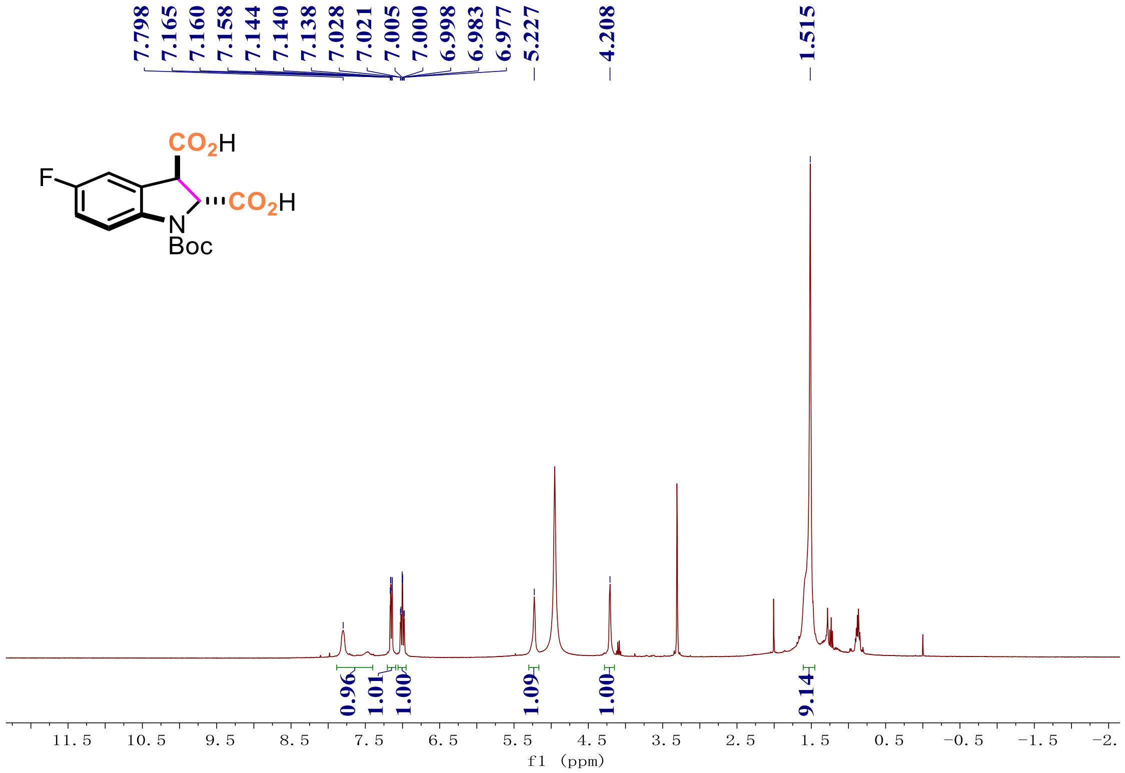

1-(*tert*-butoxycarbonyl)-5-fluoroindoline-2,3-dicarboxylic acid (7c)

^{13}C NMR (100 MHz, CD_3OD)

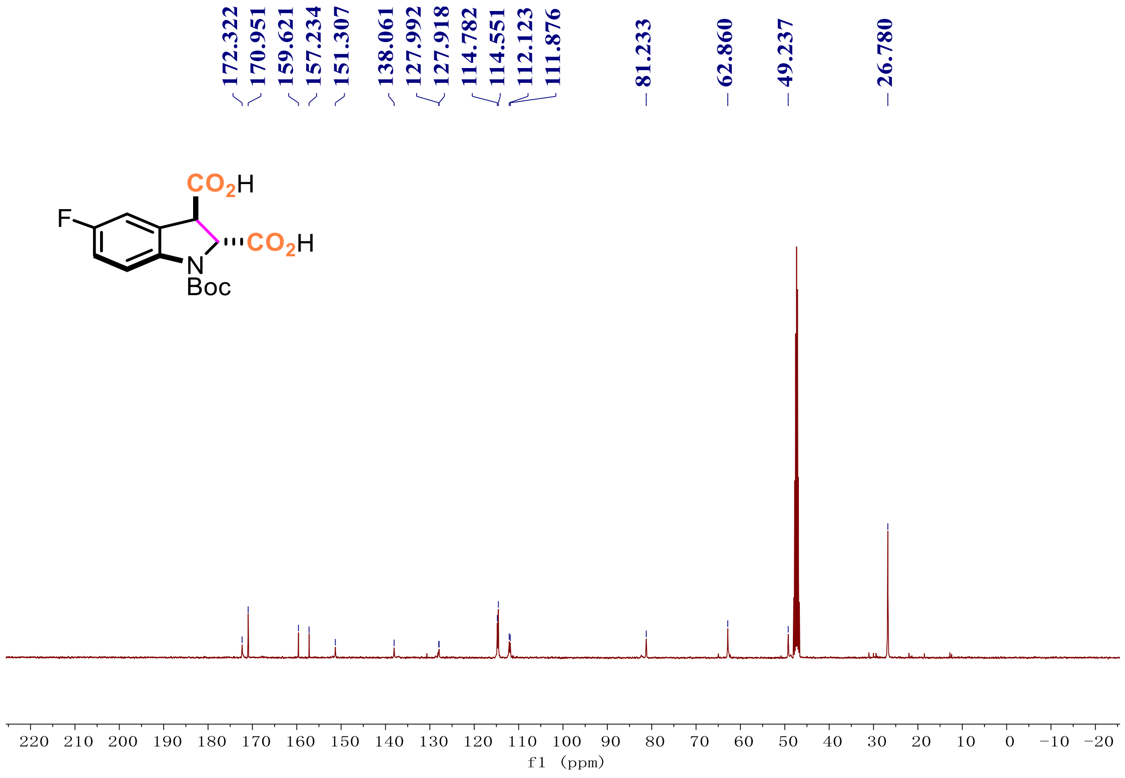

1-(*tert*-butoxycarbonyl)-5-fluoroindoline-2,3-dicarboxylic acid (7c)

^{19}F NMR (376 MHz, CD_3OD)

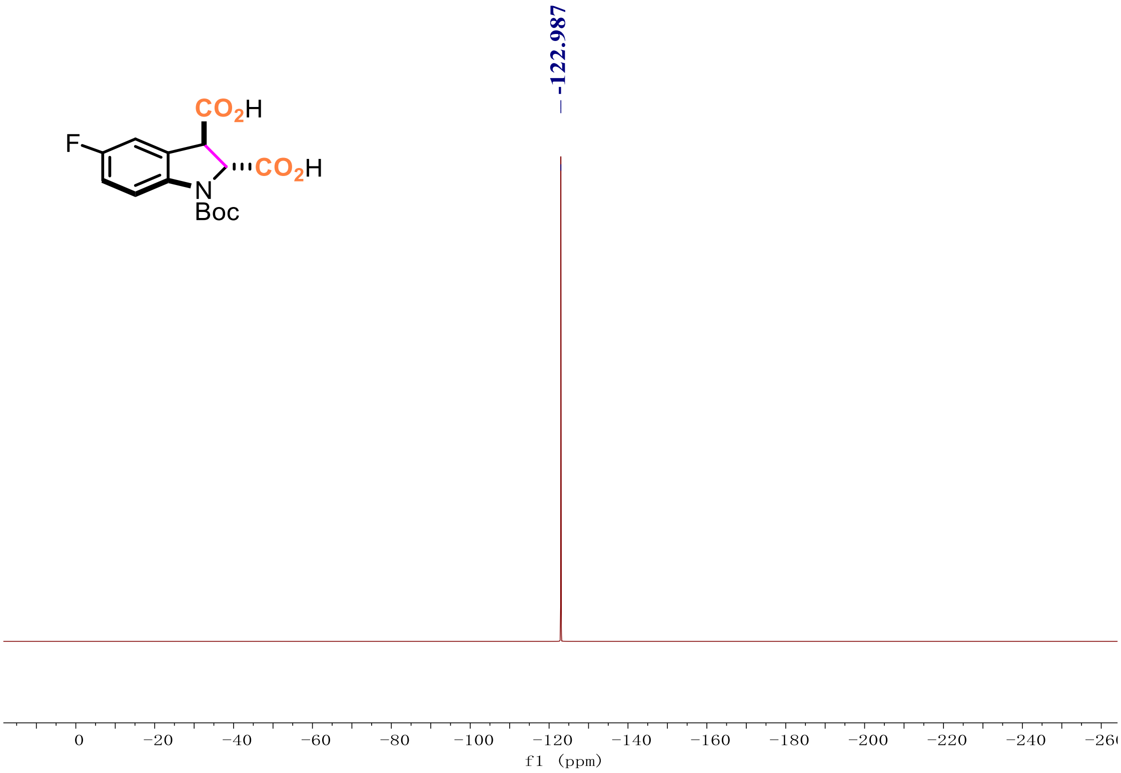

1-(*tert*-butoxycarbonyl)-7-fluoroindoline-2,3-dicarboxylic acid (7d)

^{1}H NMR (400 MHz, CD_3OD)

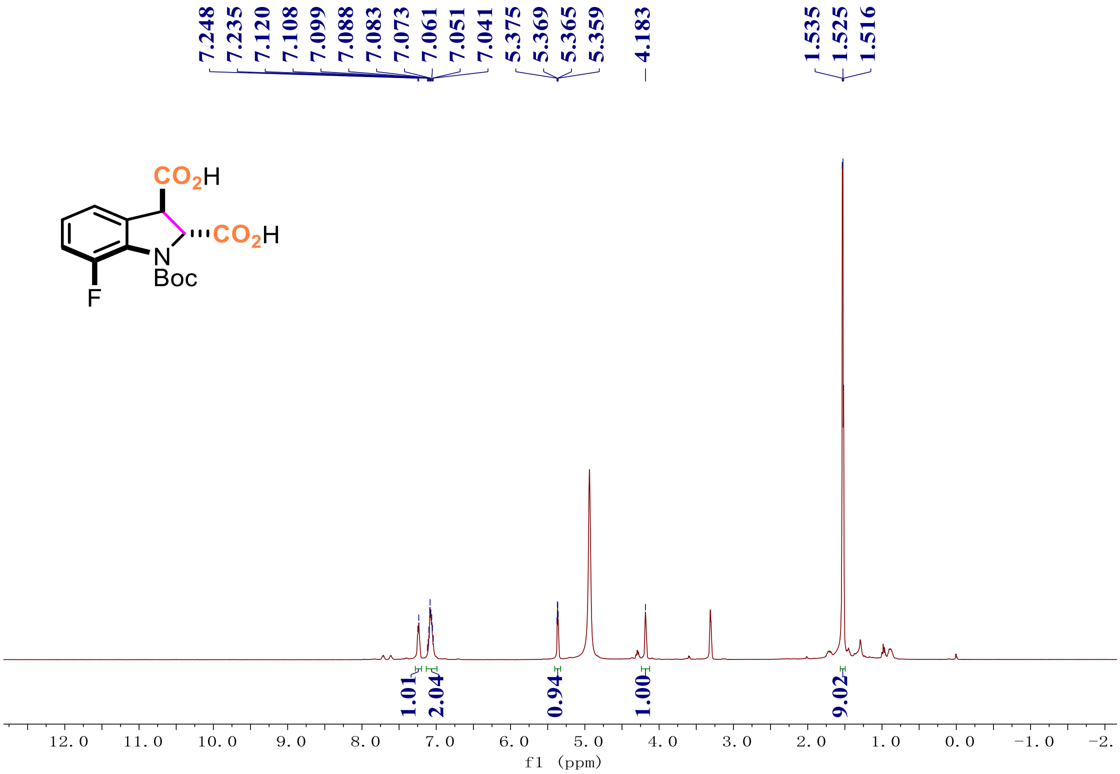

1-(*tert*-butoxycarbonyl)-7-fluoroindoline-2,3-dicarboxylic acid (7d)

^{13}C NMR (100 MHz, CD_3OD)

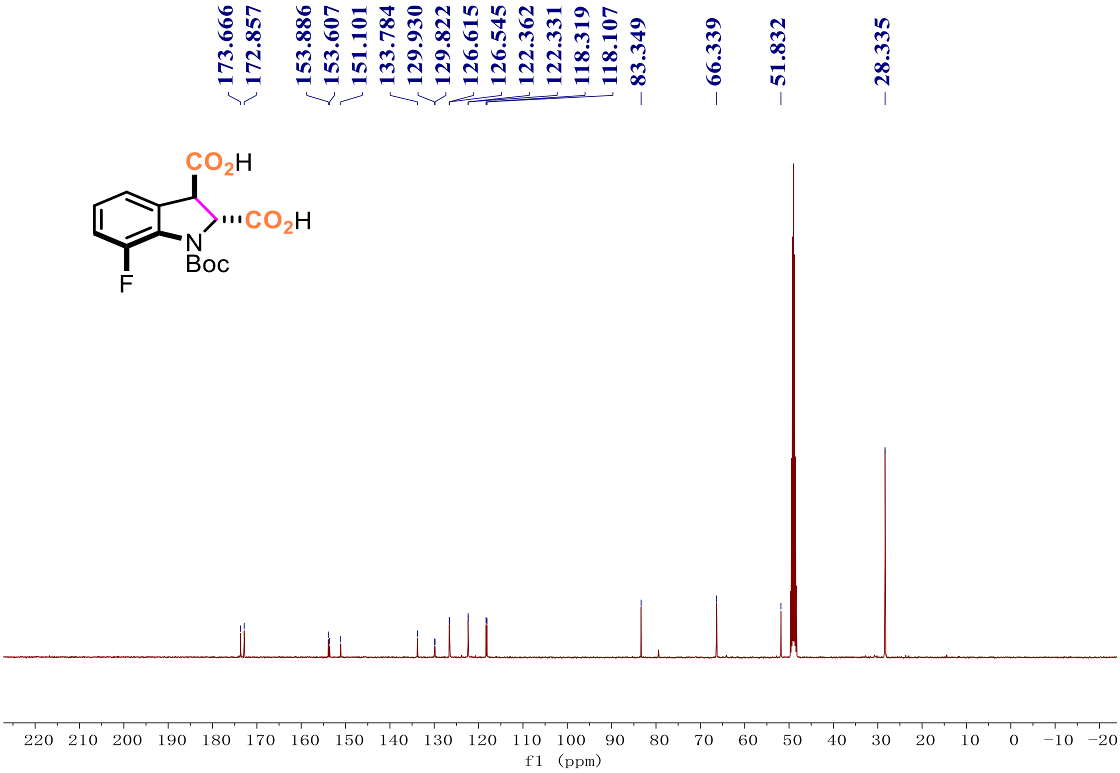

1-(*tert*-butoxycarbonyl)-7-fluoroindoline-2,3-dicarboxylic acid (7d)
^{19}F NMR (376 MHz, CD_3OD)

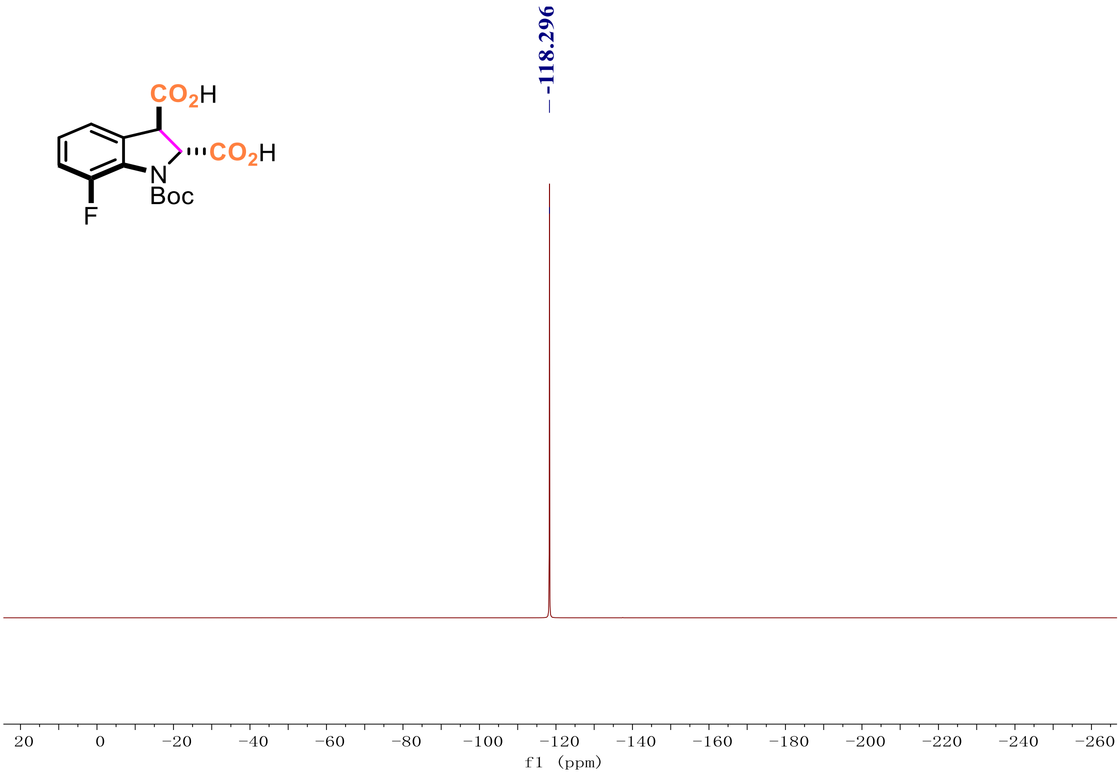

1-(tert-butoxycarbonyl)-6-methylindoline-2,3-dicarboxylic acid (7e)

^{1}H NMR (400 MHz, CD$_3$OD)

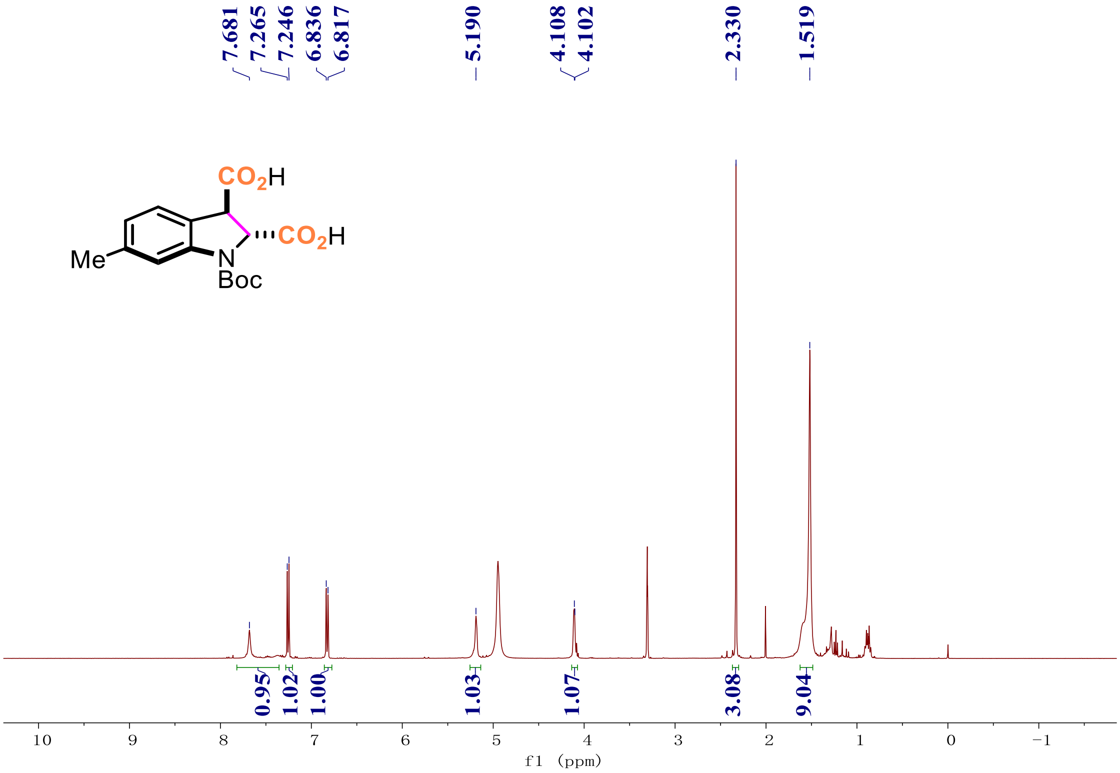

1-(tert-butoxycarbonyl)-6-methylindoline-2,3-dicarboxylic acid (7e)
^{13}C NMR (100 MHz, CD$_3$OD)

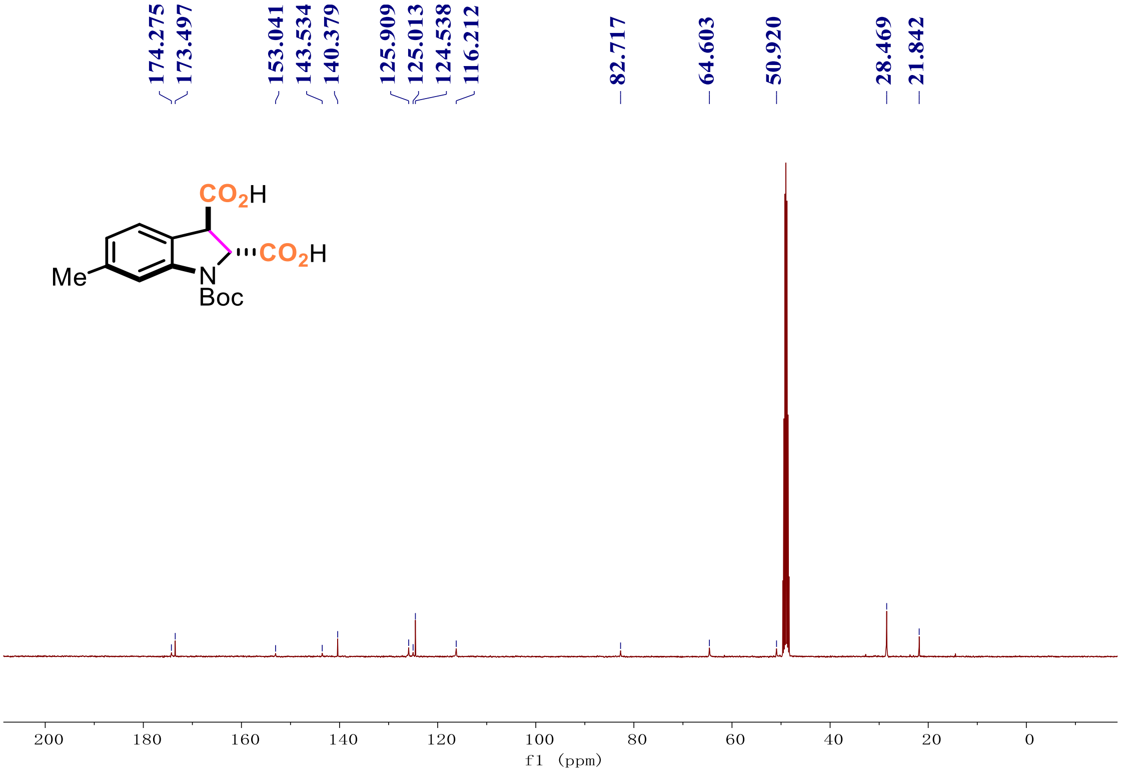

1-(*tert*-butoxycarbonyl)-6-(methoxycarbonyl)indoline-2,3-dicarboxylic acid (7f)

^{1}H NMR (400 MHz, CD_3OD)

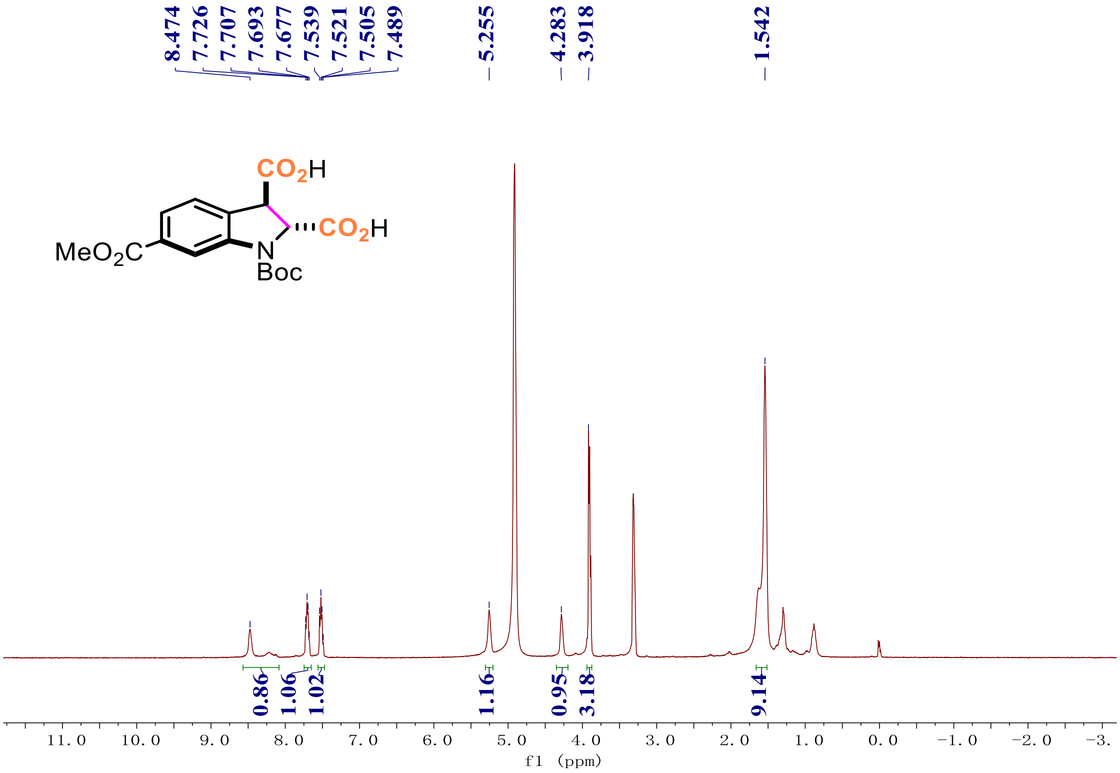

1-(*tert*-butoxycarbonyl)-6-(methoxycarbonyl)indoline-2,3-dicarboxylic acid (7f)

^{13}C NMR (100 MHz, CD_3OD)

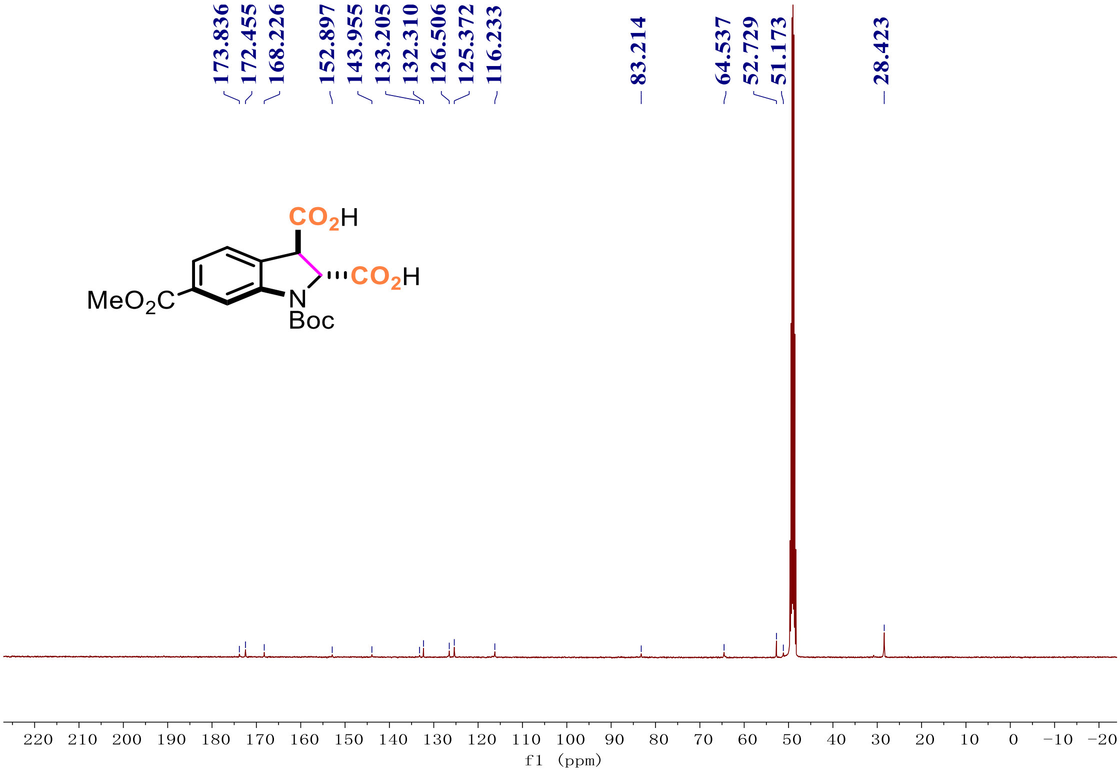

1-(*tert*-butoxycarbonyl)-3-methylindoline-2,3-dicarboxylic acid (7g)

^{1}H NMR (400 MHz, CD_3OD)

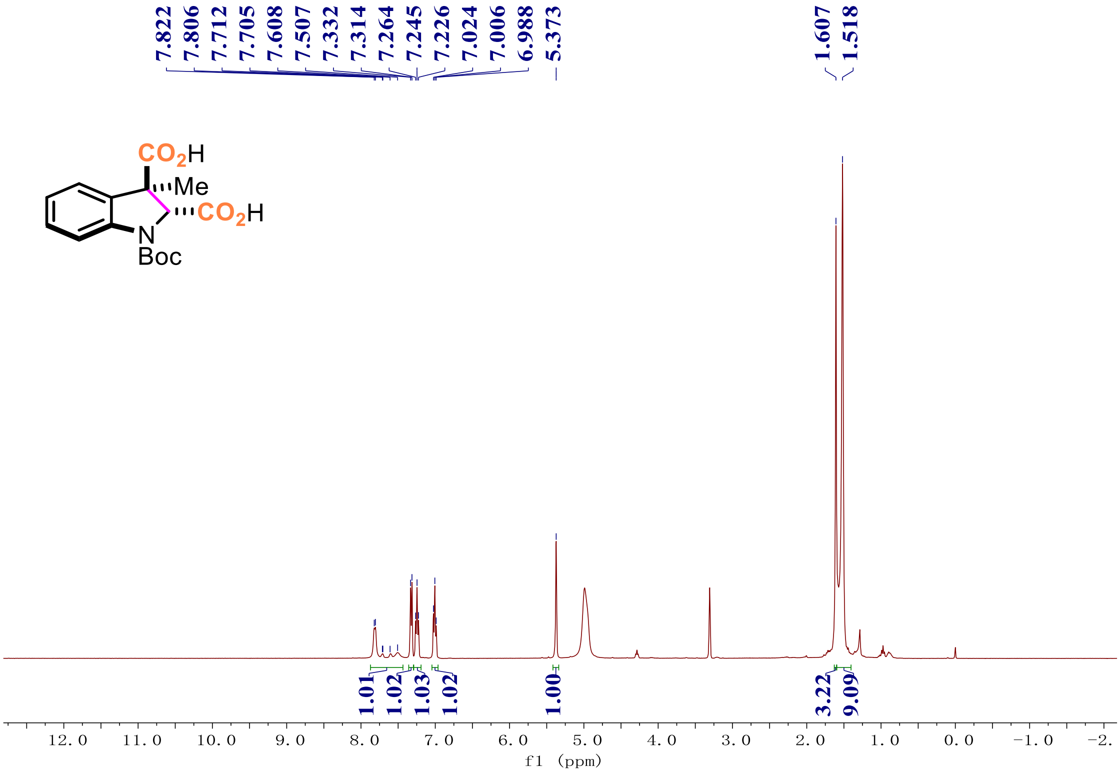

1-(*tert*-butoxycarbonyl)-3-methylindoline-2,3-dicarboxylic acid (7g)

^{13}C NMR (100 MHz, CD_3OD)

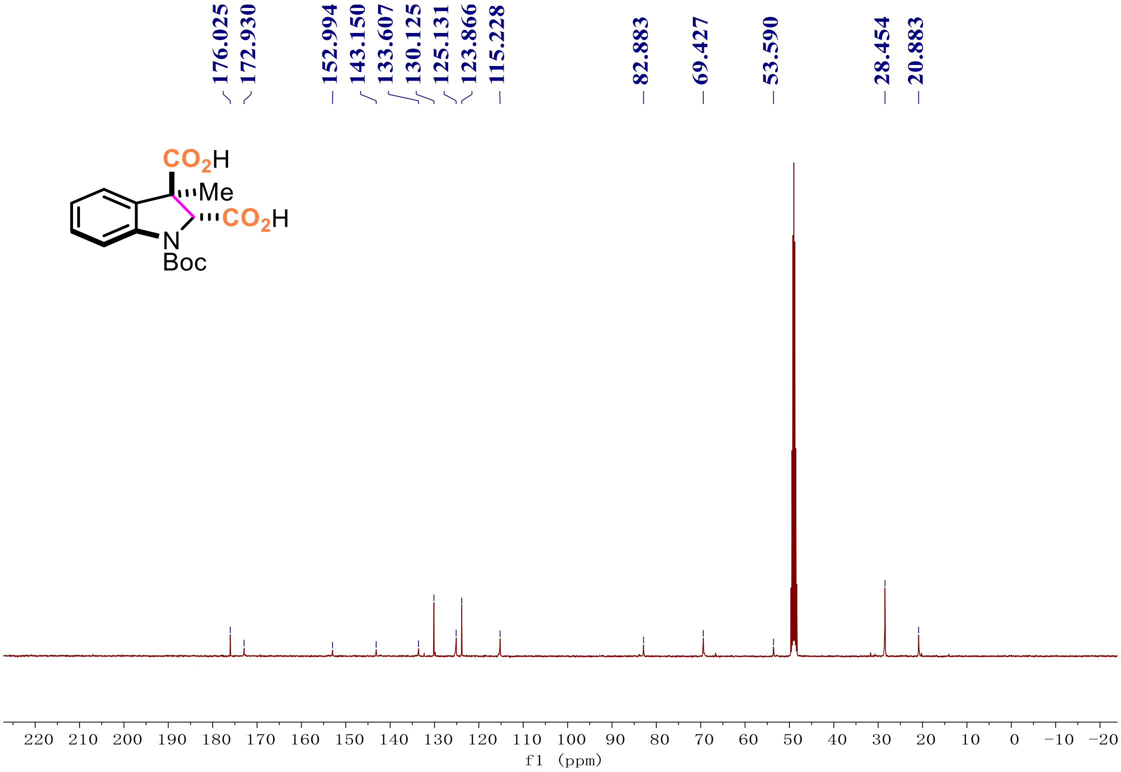

1-(*tert*-butoxycarbonyl)-2-phenylindoline-2,3-dicarboxylic acid (7h)

^{1}H NMR (400 MHz, CD_3OD)

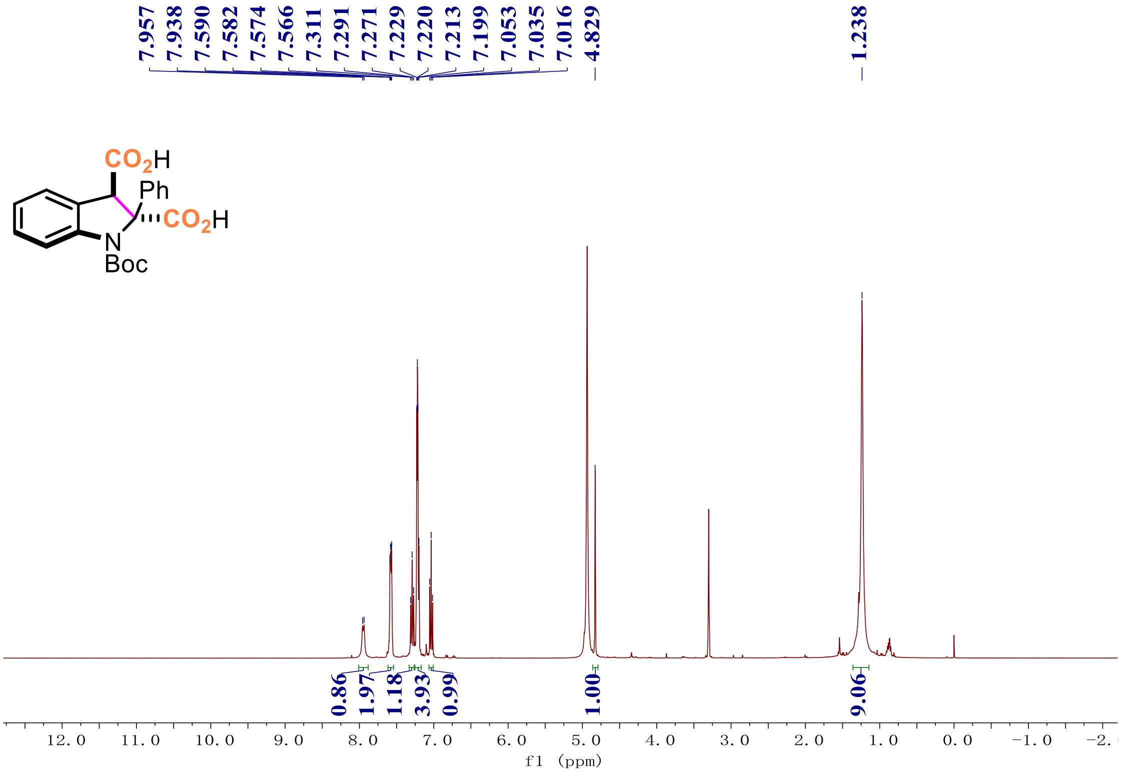

1-(*tert*-butoxycarbonyl)-2-phenylindoline-2,3-dicarboxylic acid (7h)
^{13}C NMR (100 MHz, CD_3OD)

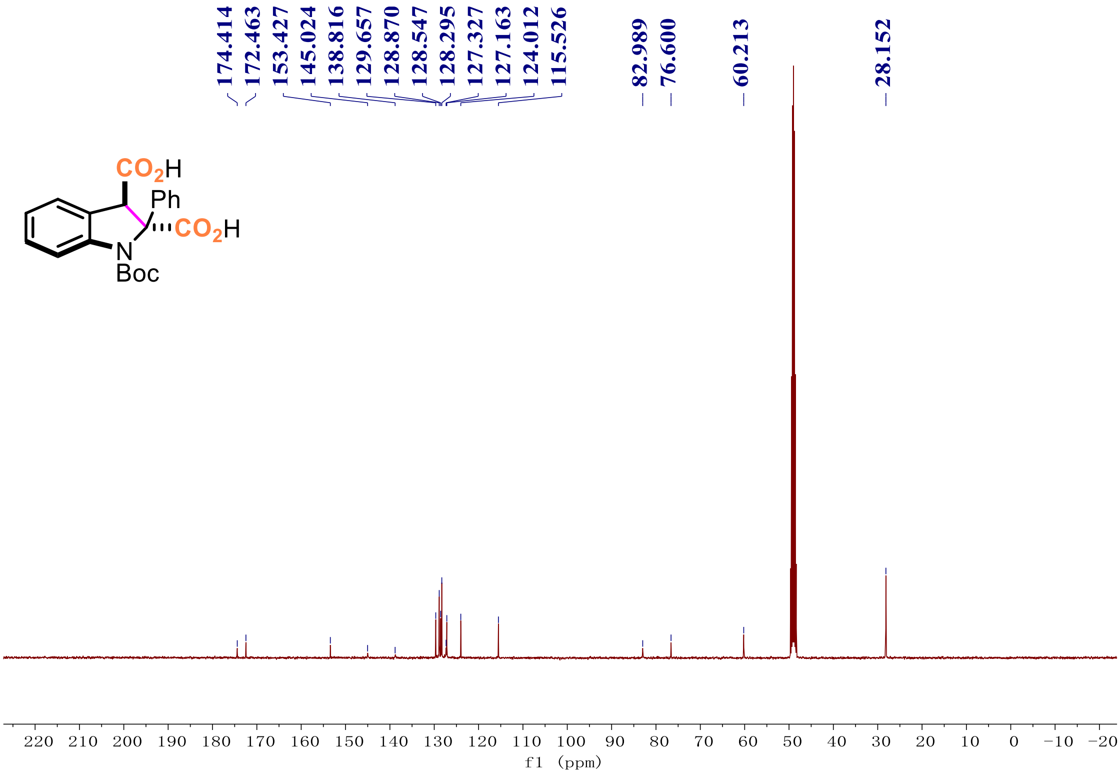

3,3-diphenylpropanoic-3-*d* acid (8a)

^{1}H NMR (400 MHz, $CDCl_3$)

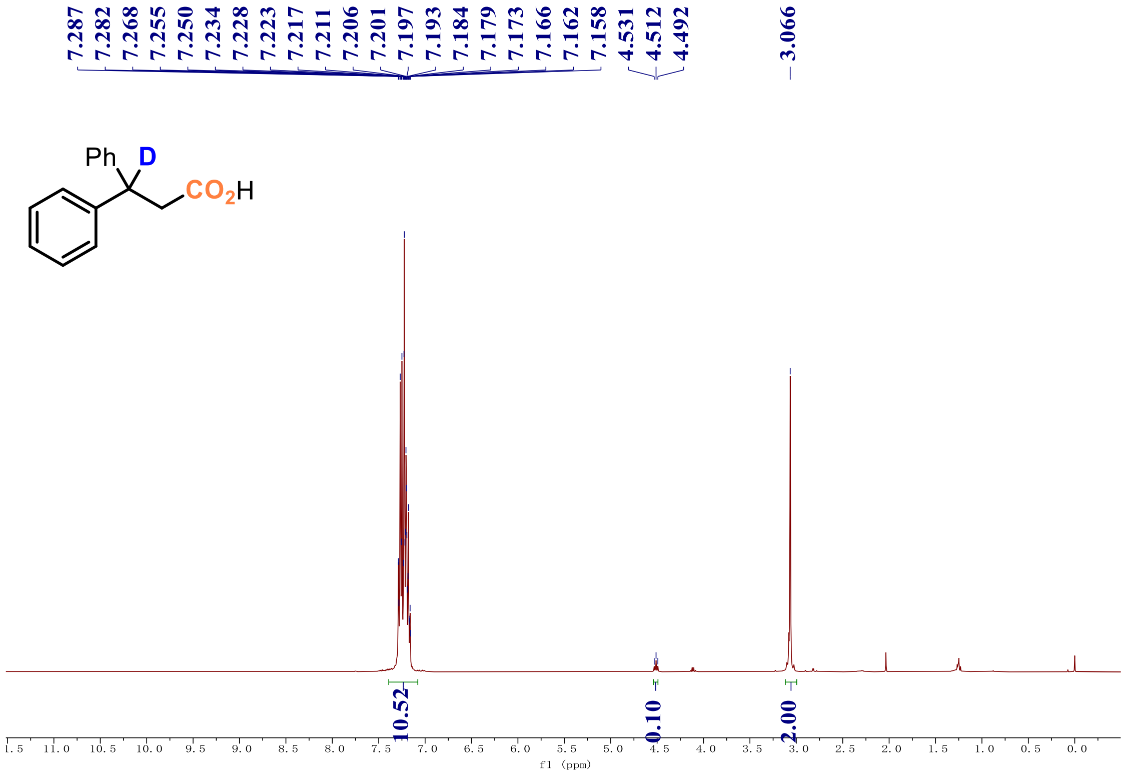

3,3-diphenylpropanoic-3-*d* acid (8a)

^{13}C NMR (100 MHz, $CDCl_3$)

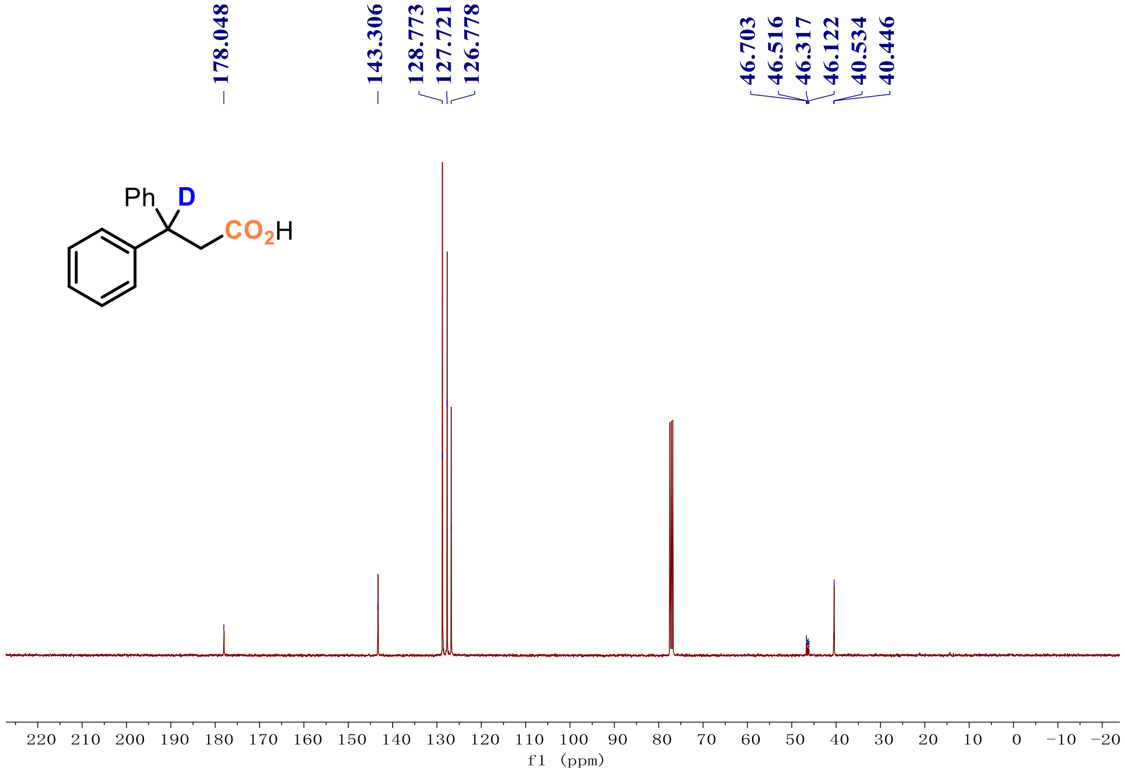

2-methyl-3,3-diphenylpropanoic-3-*d* acid (8b)

^{1}H NMR (400 MHz, $CDCl_3$)

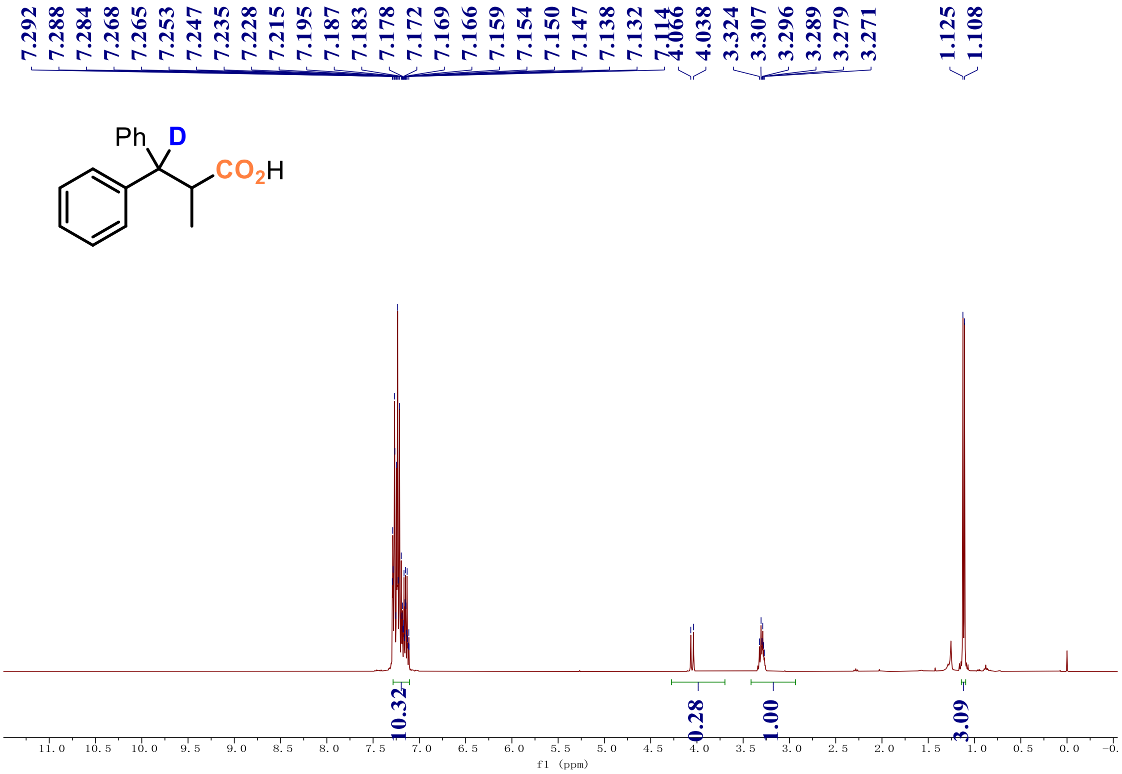

2-methyl-3,3-diphenylpropanoic-3-*d* acid (8b)

^{13}C NMR (100 MHz, $CDCl_3$)

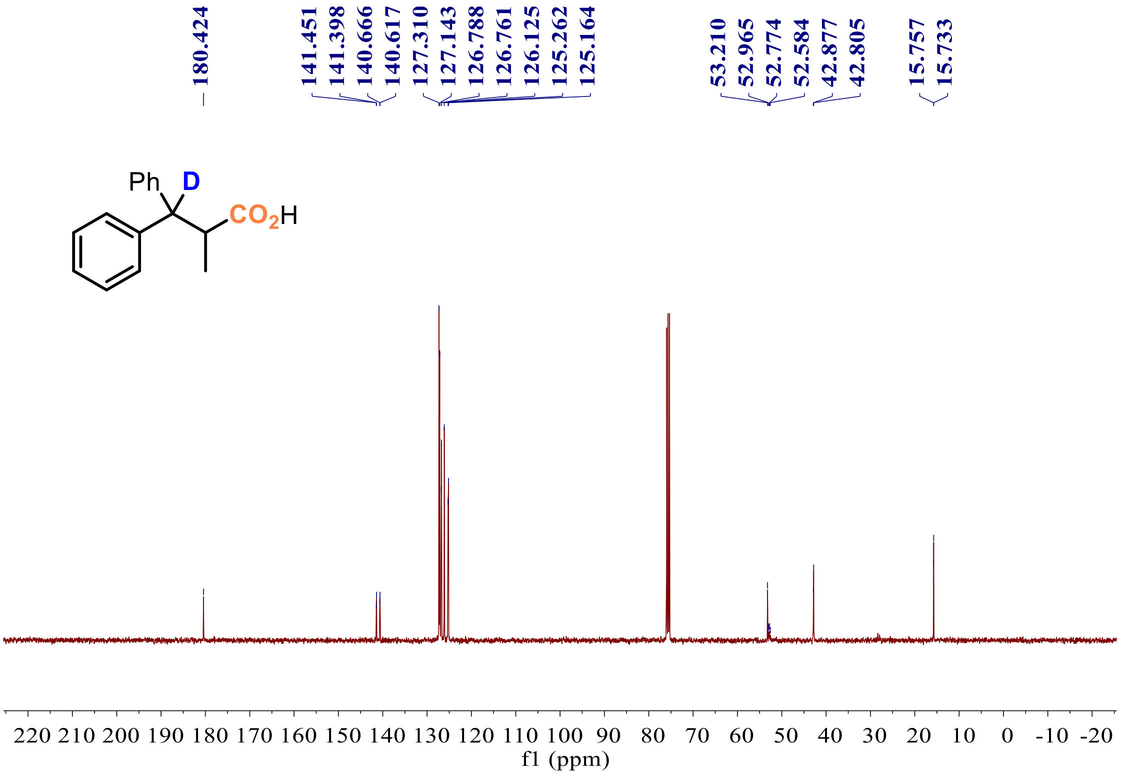

(*E*)-5-phenylpent-3-enoic-5-*d* acid (8c)

^{1}H NMR (400 MHz, $CDCl_3$)

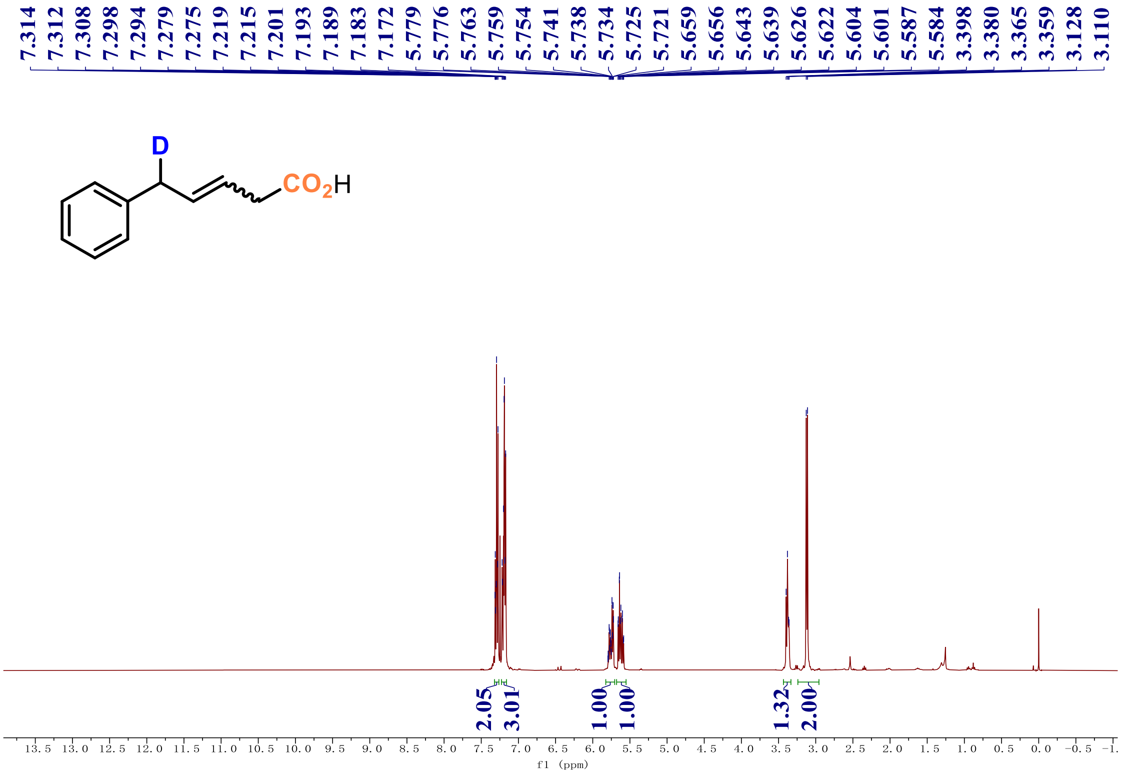

(*E*)-5-phenylpent-3-enoic-5-*d* acid (8c)

^{13}C NMR (100 MHz, $CDCl_3$)

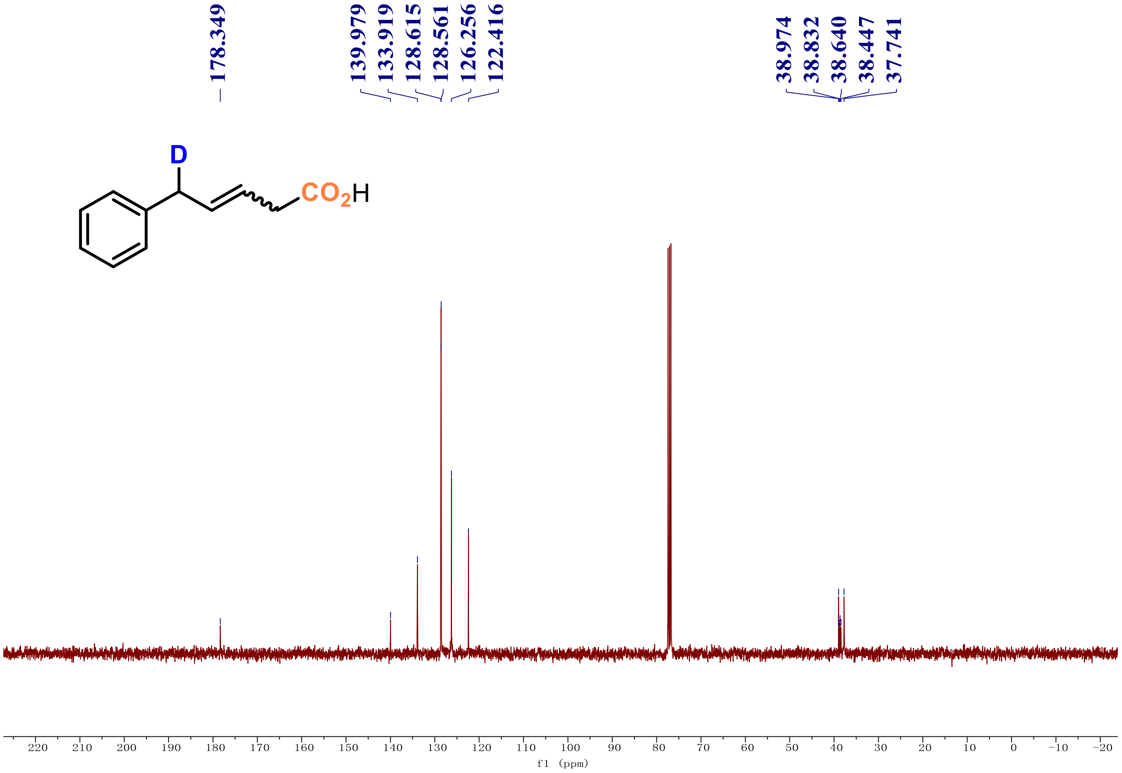

3-phenylpropanoic-3-*d* acid (8d)

^{1}H NMR (400 MHz, $CDCl_3$)

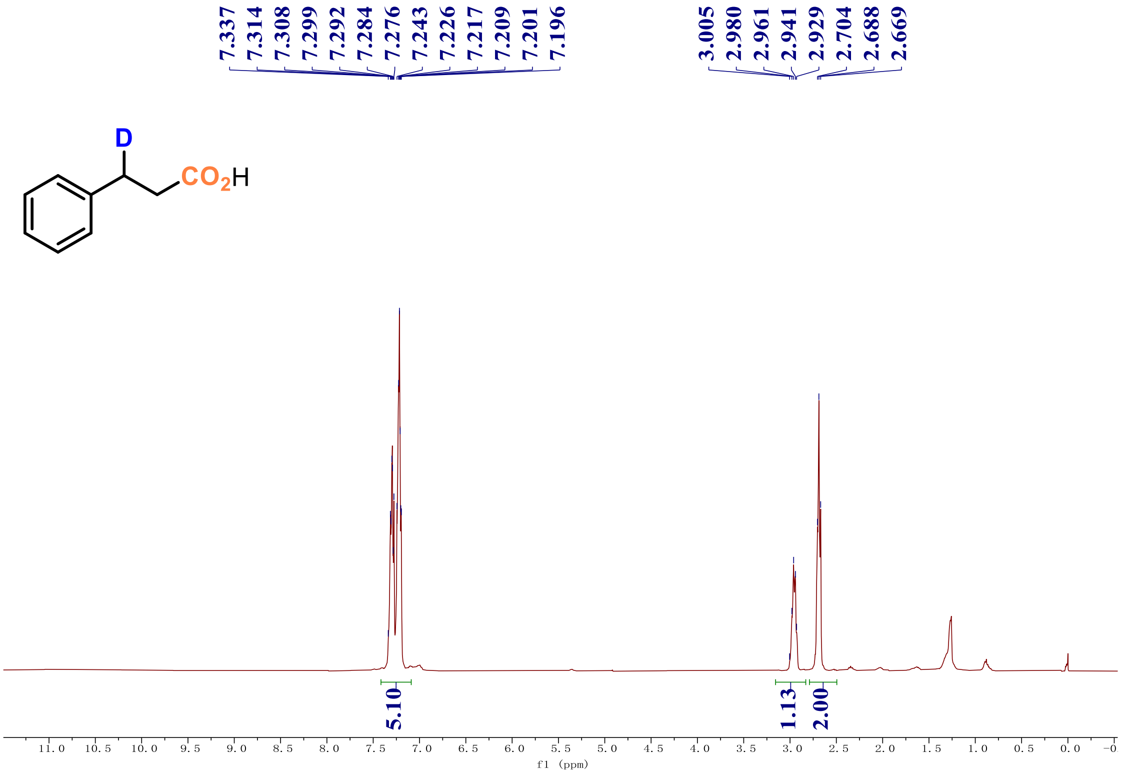

3-phenylpropanoic-3-*d* acid (8d)

^{13}C NMR (100 MHz, $CDCl_3$)

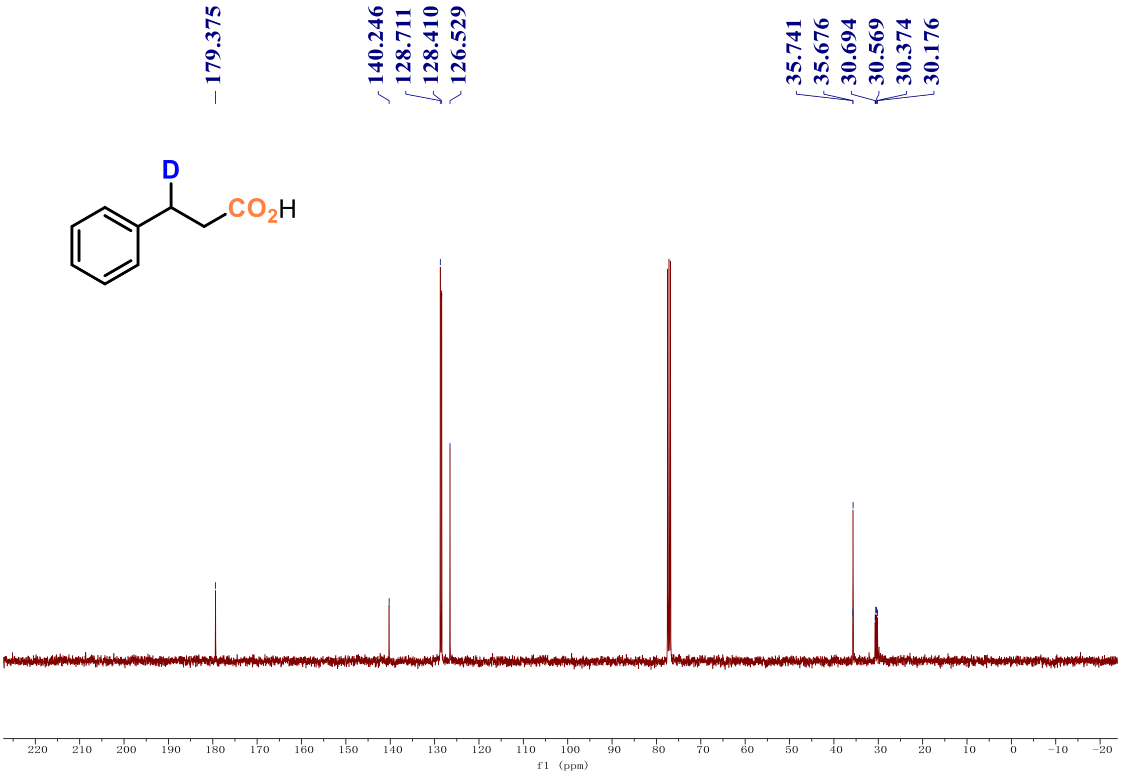

3-phenylbutanoic-3-*d* acid (8e)

^{1}H NMR (400 MHz, $CDCl_3$)

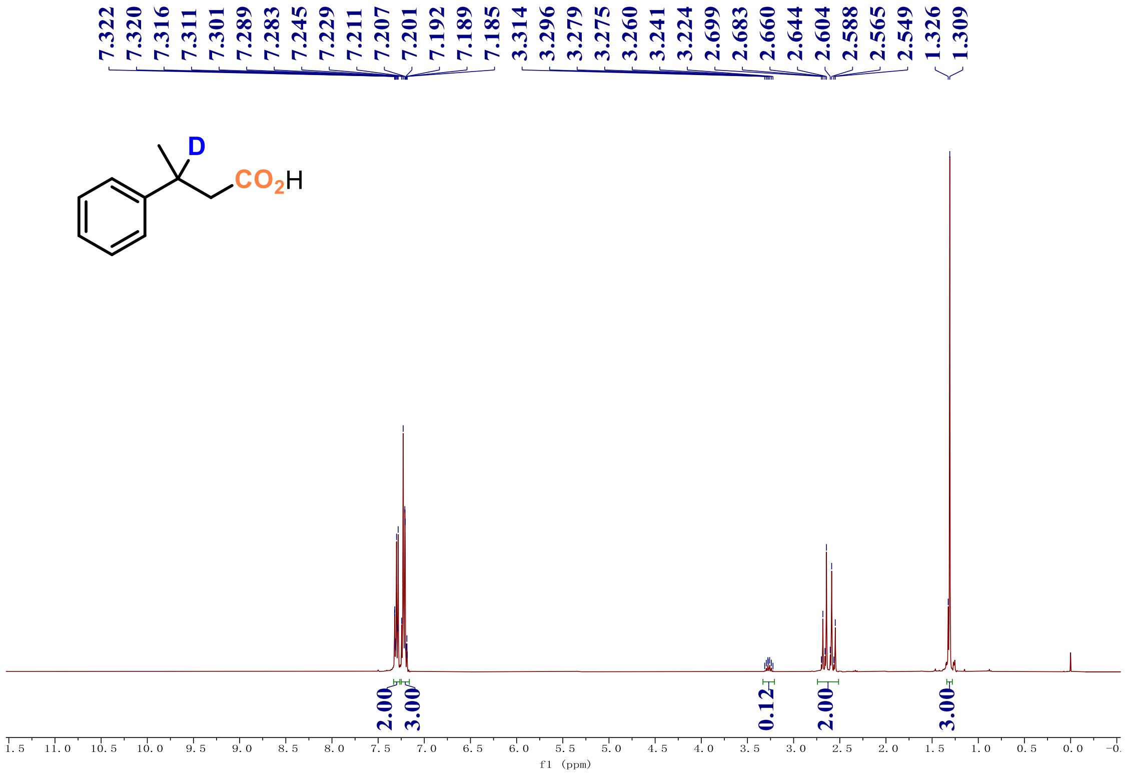

3-phenylbutanoic-3-*d* acid (8e)

^{13}C NMR (100 MHz, $CDCl_3$)

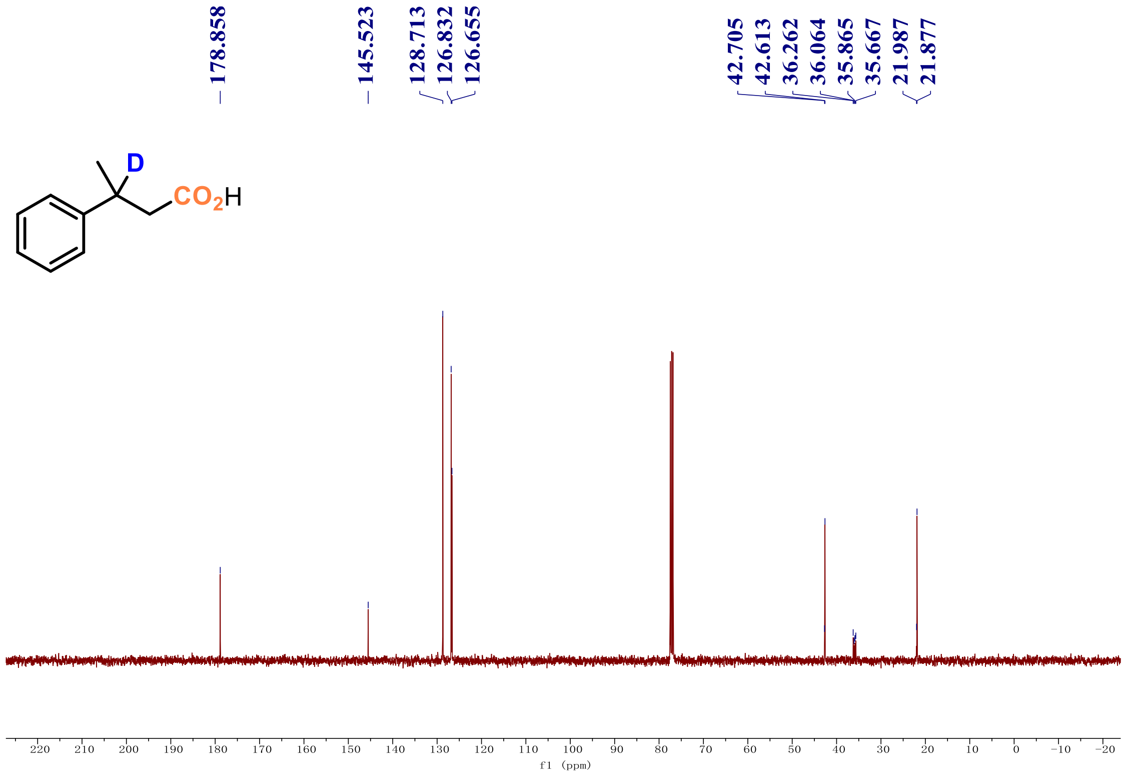

3,3-diphenylpyrrolidine-2,5-dione (9)

^{1}H NMR (400 MHz, CDCl$_3$)

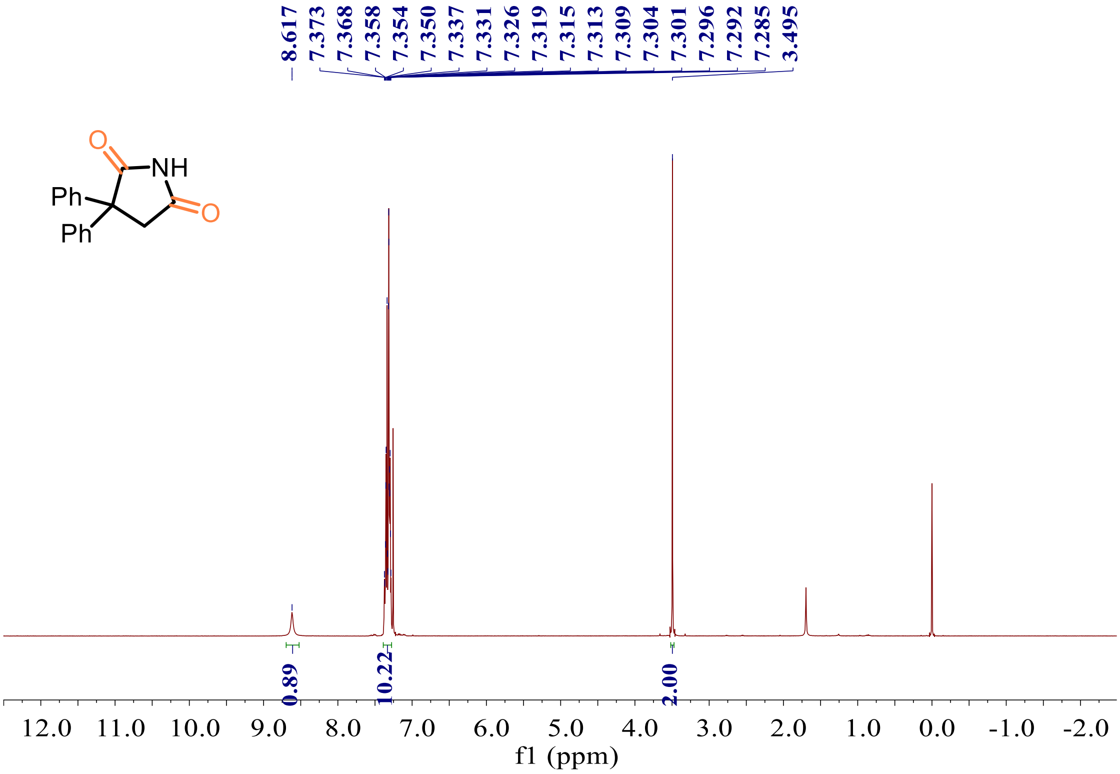

3,3-diphenylpyrrolidine-2,5-dione (9)

^{13}C NMR (100 MHz, CDCl$_3$)

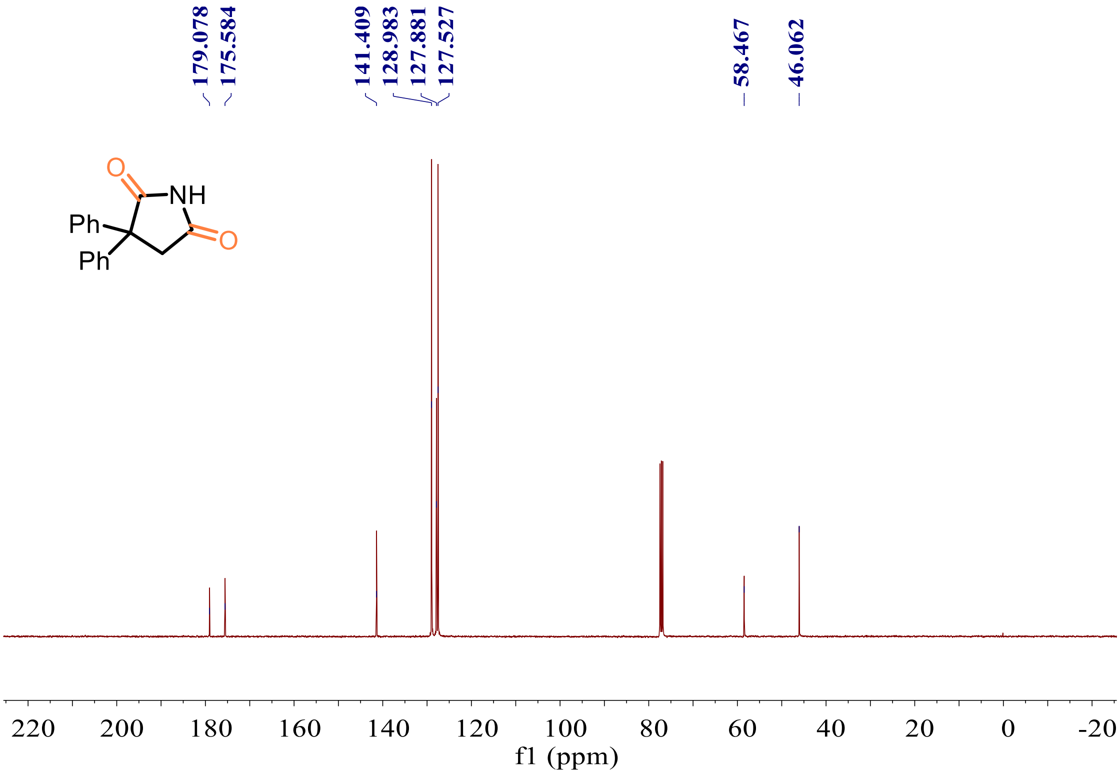

dimethyl 2-(2,2-diphenylvinyl)succinate (10)

^{1}H NMR (400 MHz, $CDCl_3$)

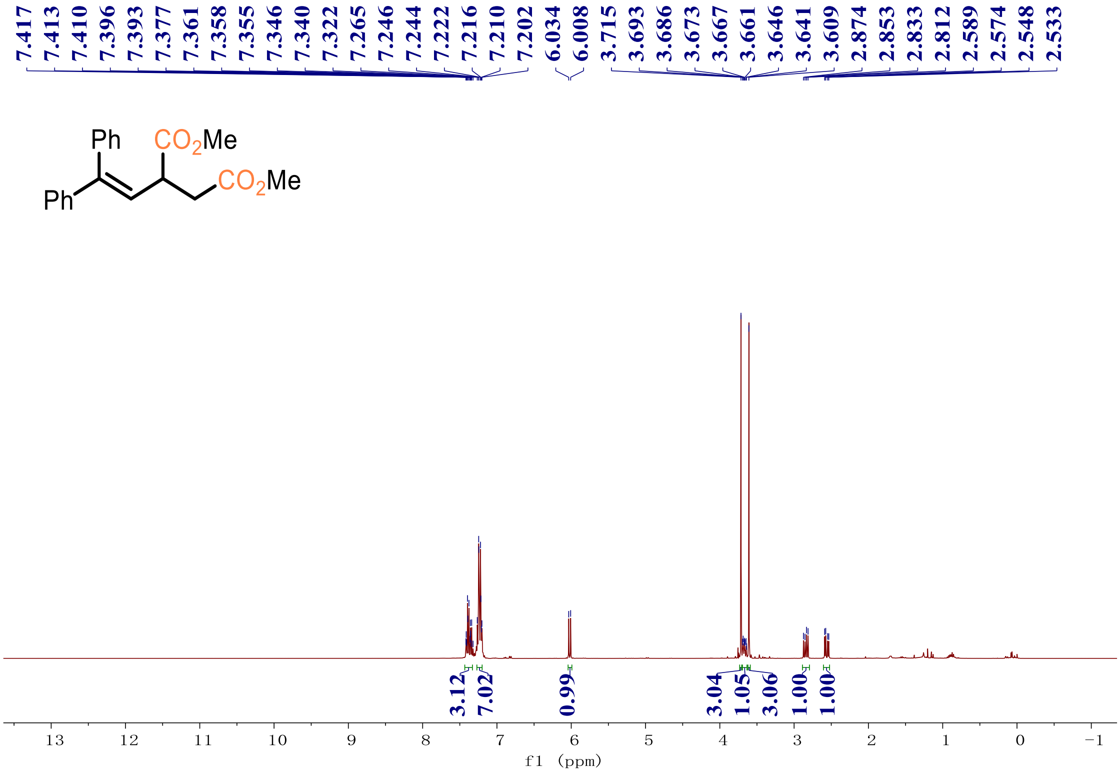

dimethyl 2-(2,2-diphenylvinyl)succinate (10)

^{13}C NMR (100 MHz, $CDCl_3$)

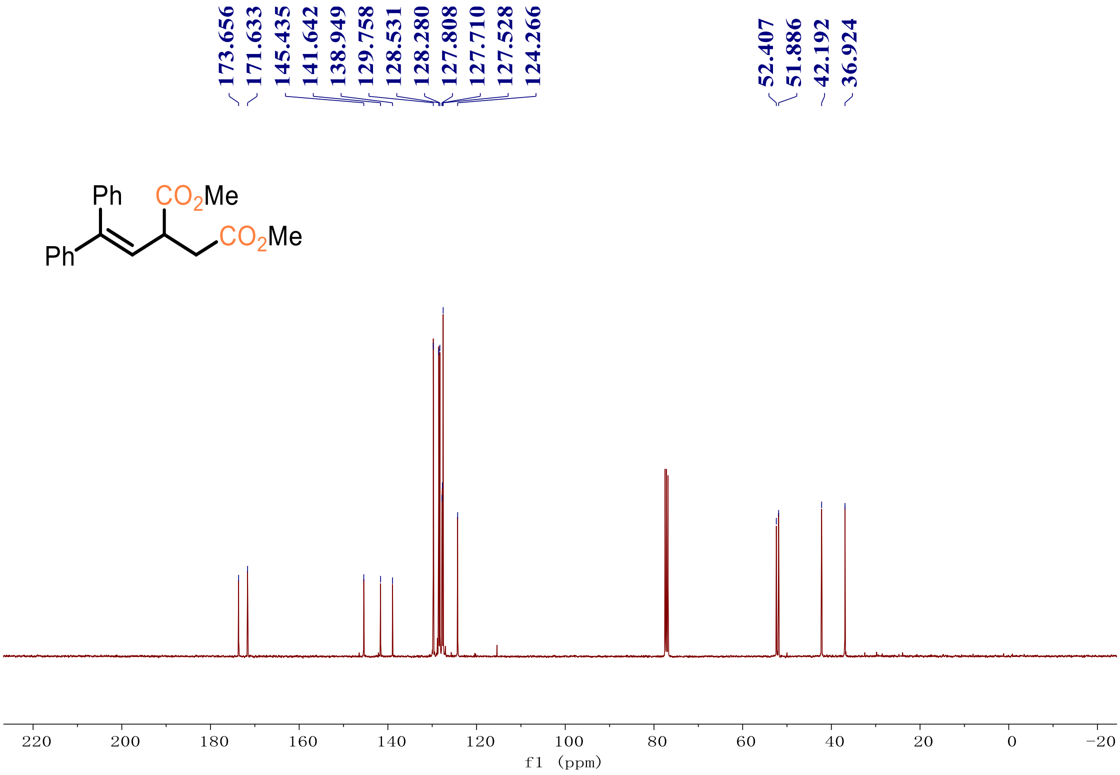

methyl 2-oxo-6,6-diphenyl-3,6-dihydro-2*H*-pyran-4-carboxylate (11)

^{1}H NMR (400 MHz, $CDCl_3$)

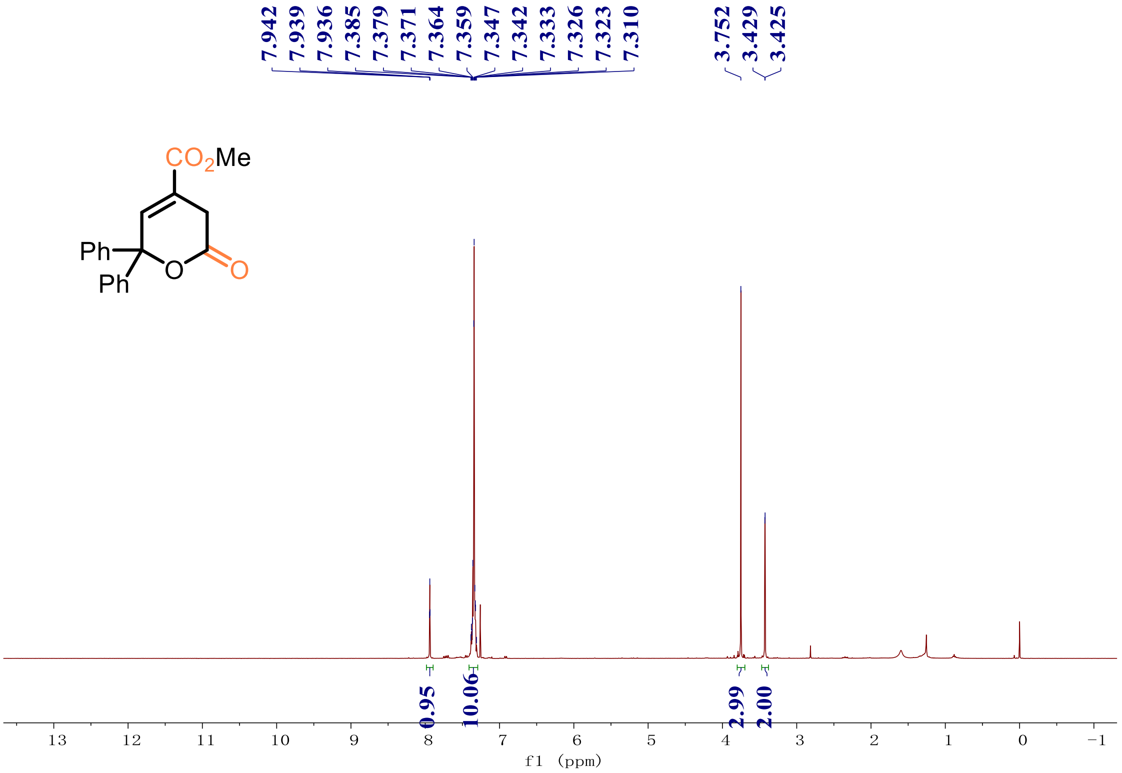

methyl 2-oxo-6,6-diphenyl-3,6-dihydro-2*H*-pyran-4-carboxylate (11)

^{13}C NMR (100 MHz, $CDCl_3$)

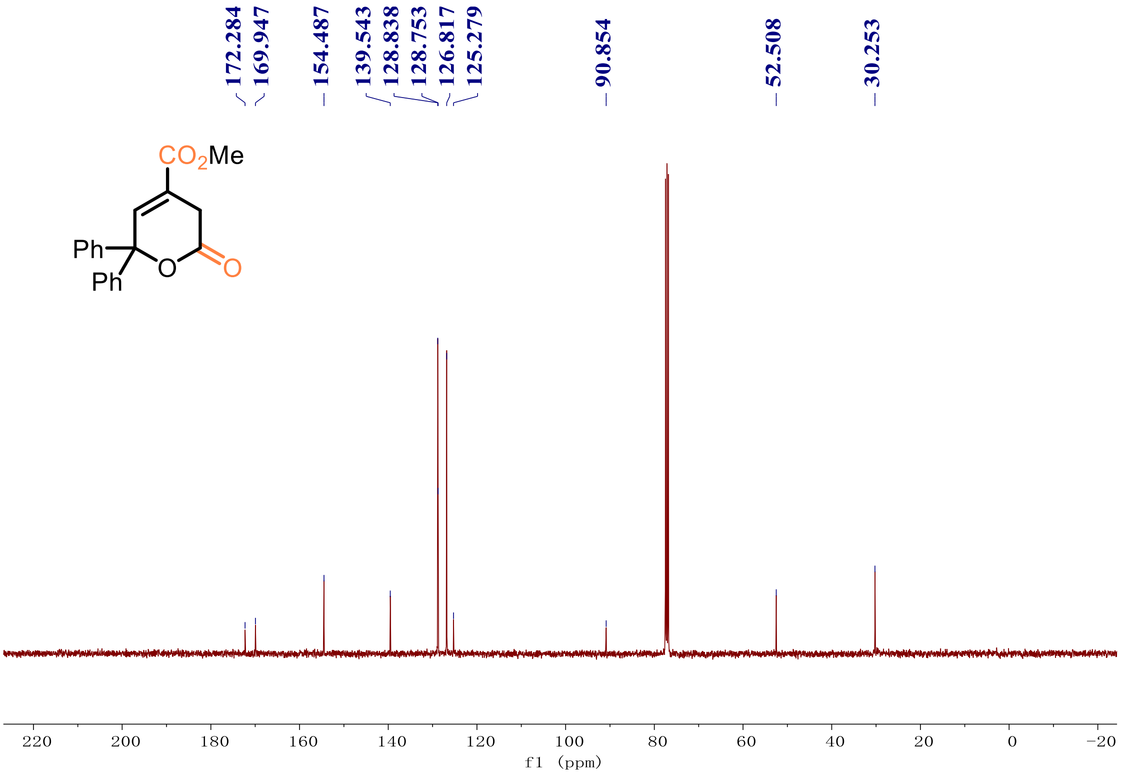

1-(*tert*-butyl) 2,3-dimethylindoline-1,2,3-tricarboxylate (12)

^{1}H NMR (400 MHz, CD_3OD)

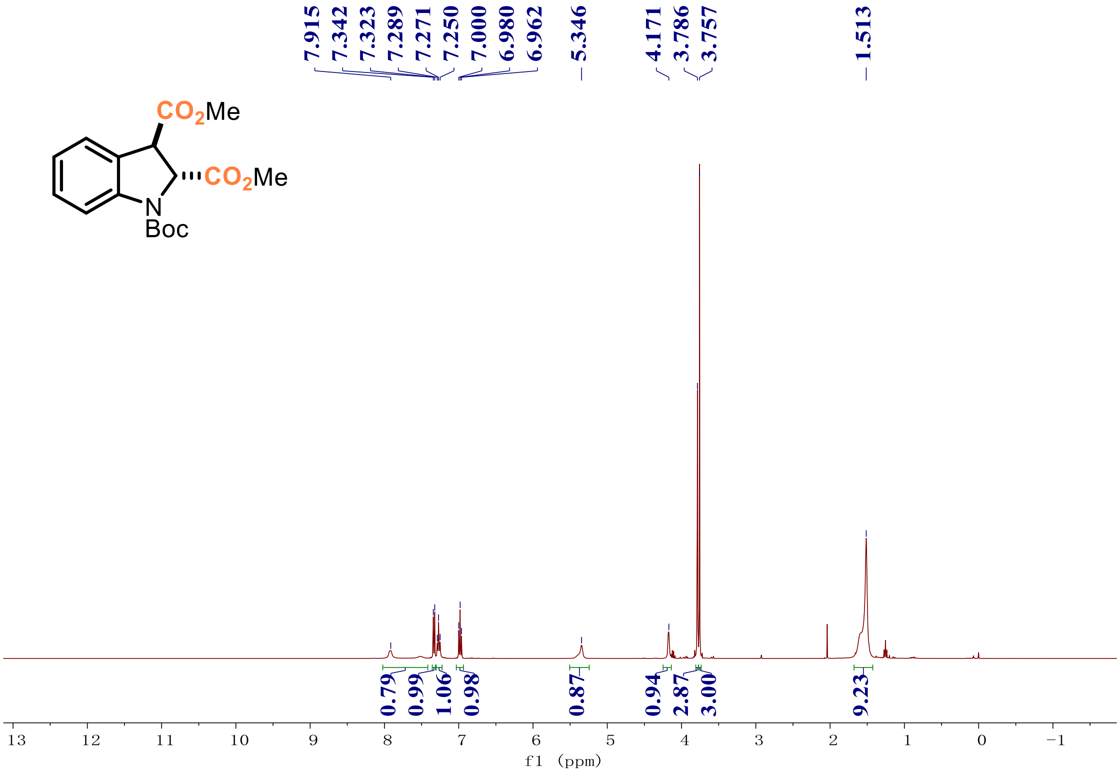

1-(*tert*-butyl) 2,3-dimethylindoline-1,2,3-tricarboxylate (12)
^{13}C NMR (100 MHz, CD_3OD)

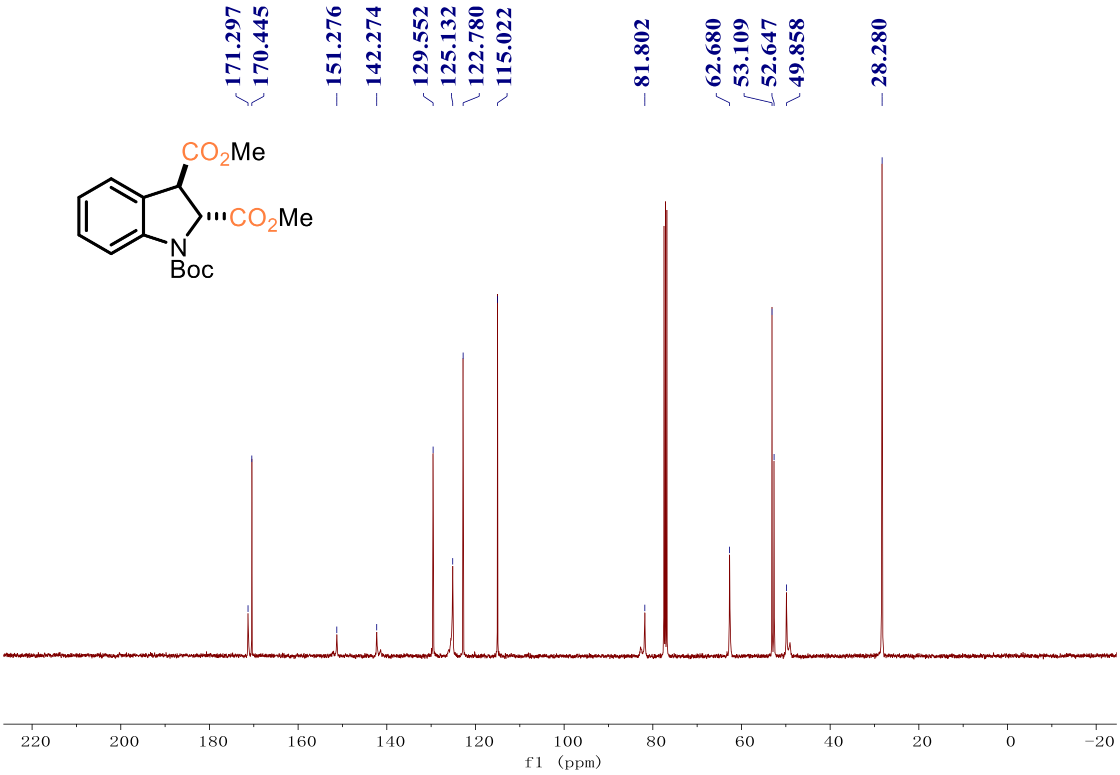

dimethylindoline-2,3-dicarboxylate (13)

^{1}H NMR (400 MHz, CDCl$_3$)

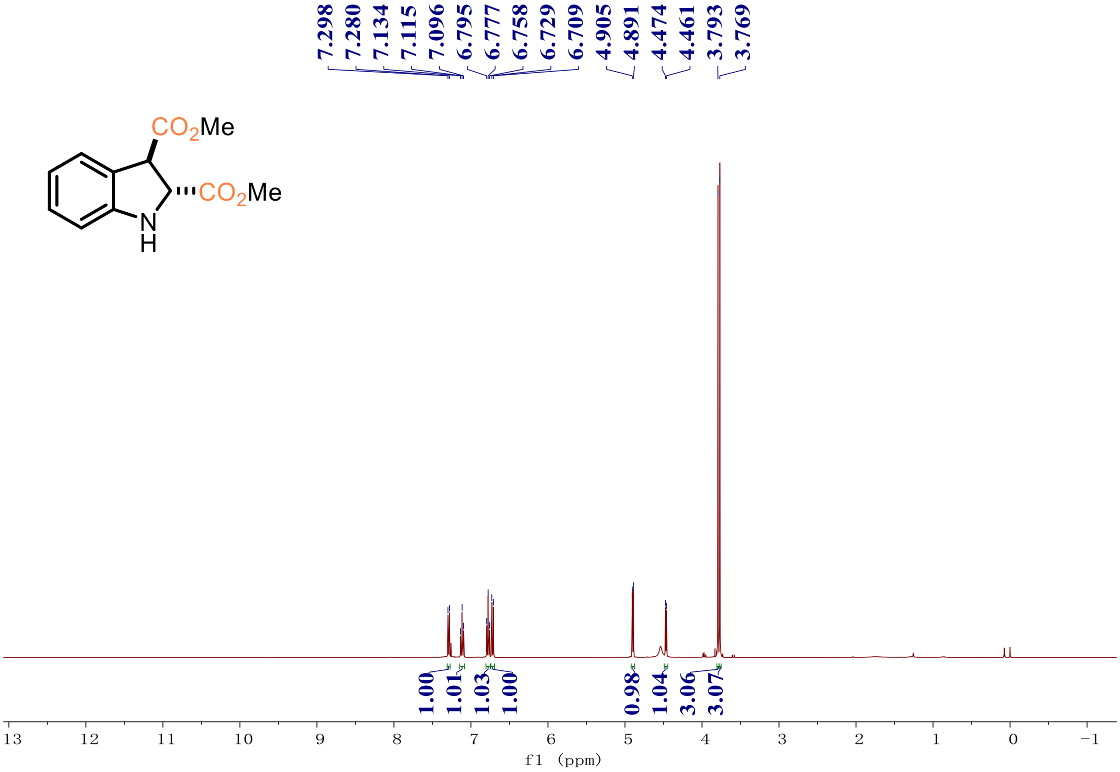

dimethylindoline-2,3-dicarboxylate (13)

^{13}C NMR (100 MHz, CDCl$_3$)

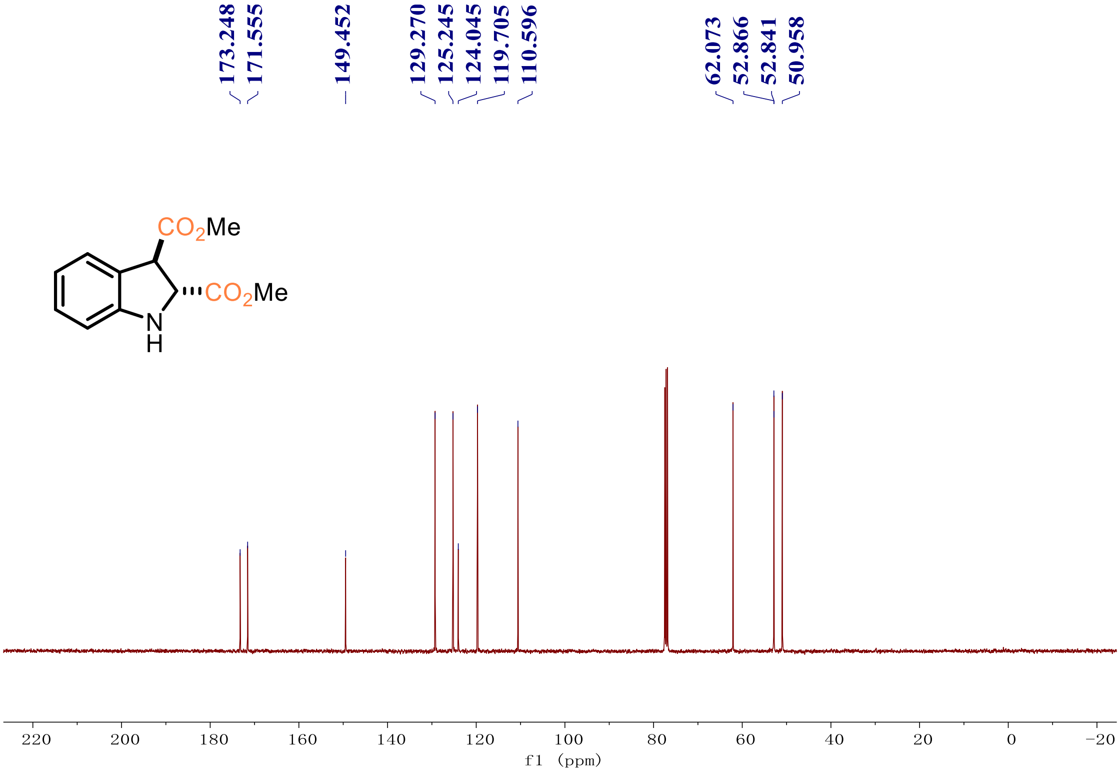

dimethyl 1*H*-indole-2,3-dicarboxylate (14)

^{1}H NMR (400 MHz, CD3OD)

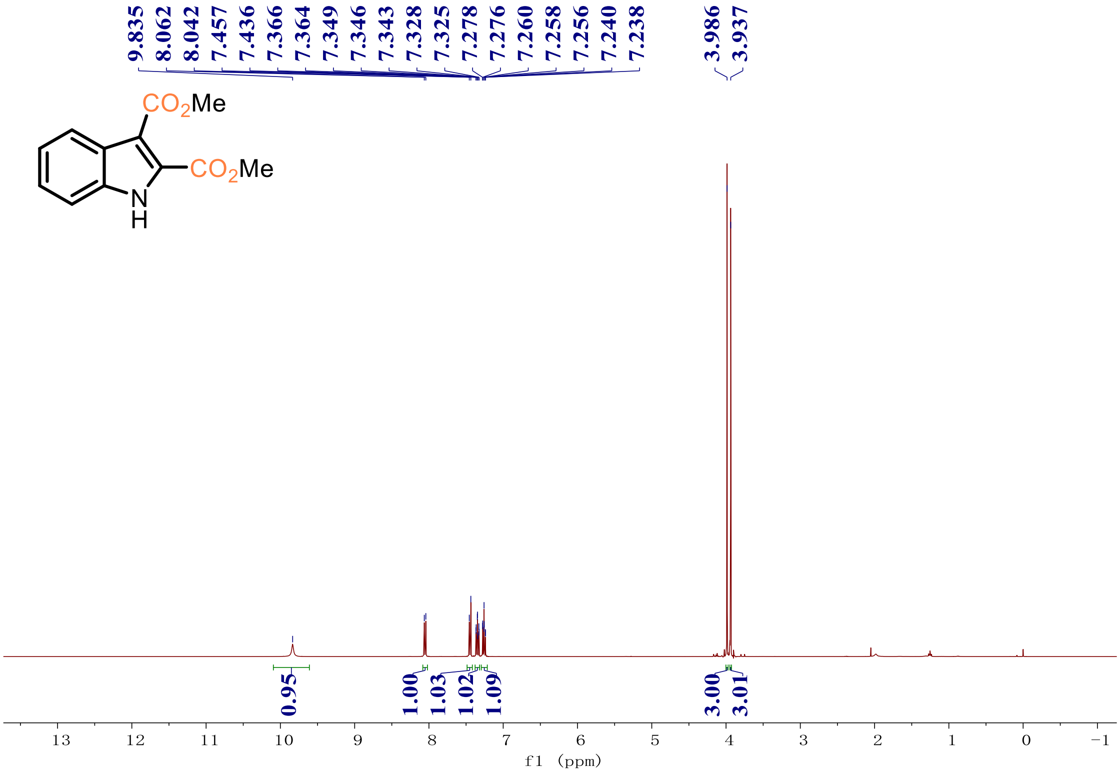

dimethyl 1*H*-indole-2,3-dicarboxylate (14)

^{13}C NMR (100 MHz, CD3OD)

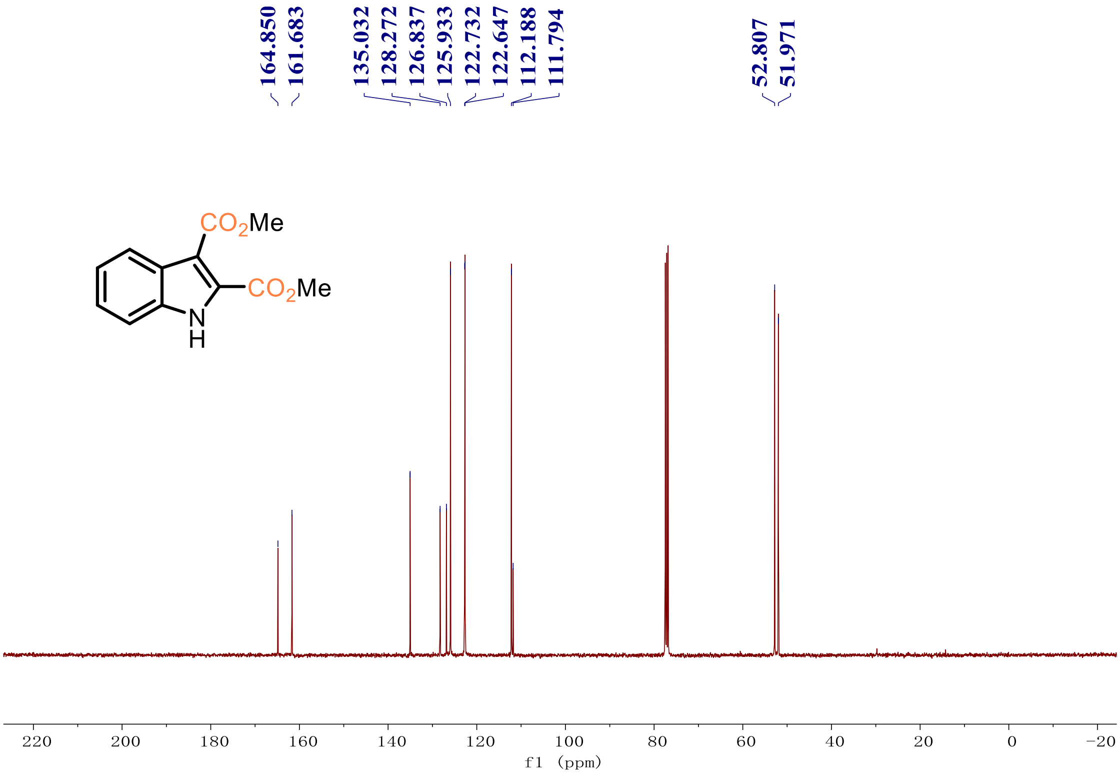

Supplement: Supplementary file 1 — oc4c01464_si_001.pdf [file oc4c01464_si_001.pdf]
